# Nonpeptidic Oxazole-Based Prolyl Oligopeptidase Ligands with Disease-Modifying Effects on α-Synuclein Mouse Models of Parkinson’s Disease

**DOI:** 10.1021/acs.jmedchem.3c00235

**Published:** 2023-05-29

**Authors:** Tommi P. Kilpeläinen, Henri T. Pätsi, Reinis Svarcbahs, Ulrika H. Julku, Tony S. Eteläinen, Hengjing Cui, Samuli Auno, Nina Sipari, Susanna Norrbacka, Teppo O. Leino, Maria Jäntti, Timo T. Myöhänen, Erik A. A. Wallén

**Affiliations:** ^†^Division of Pharmacology and Pharmacotherapy, and ^‡^Division of Pharmaceutical Chemistry and Technology, Drug Research Program, Faculty of Pharmacy, University of Helsinki, P.O. Box 56, 00014 Helsinki, Finland; § School of Pharmacy, Faculty of Health Sciences, University of Eastern Finland, Yliopistonranta 1C, 70211 Kuopio, Finland; ∥ Viikki Metabolomics Unit, Department of Biosciences, University of Helsinki, Viikinkaari 5 E, 00014 Helsinki, Finland

## Abstract

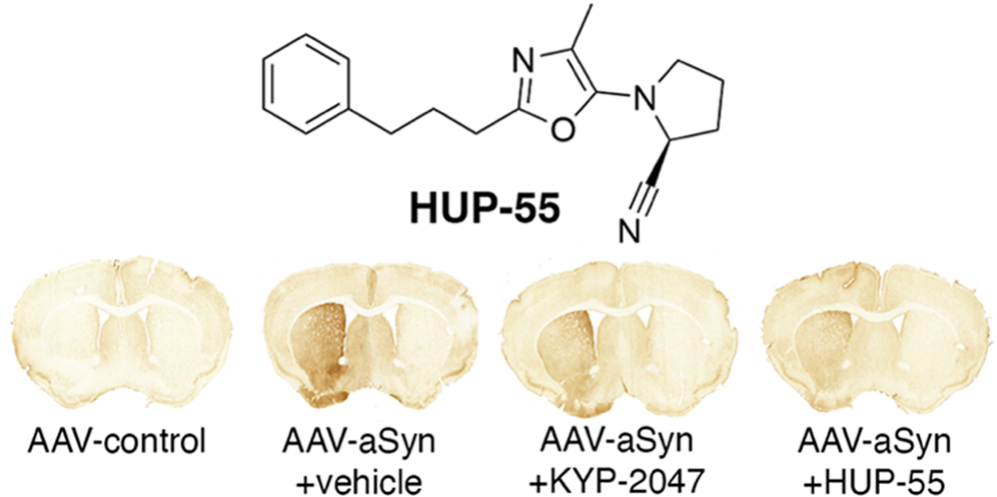

Prolyl oligopeptidase (PREP) is a widely distributed serine protease in the human body cleaving proline-containing peptides; however, recent studies suggest that its effects on pathogenic processes underlying neurodegeneration are derived from direct protein–protein interactions (PPIs) and not from its regulation of certain neuropeptide levels. We discovered novel nonpeptidic oxazole-based PREP inhibitors, which deviate from the known structure–activity relationship for PREP inhibitors. These new compounds are effective modulators of the PPIs of PREP, reducing α-synuclein (αSyn) dimerization and enhancing protein phosphatase 2A activity in a concentration–response manner, as well as reducing reactive oxygen species production. From the best performing oxazoles, **HUP-55** was selected for *in vivo* studies. Its brain penetration was evaluated, and it was tested in αSyn virus vector-based and αSyn transgenic mouse models of Parkinson’s disease, where it restored motor impairment and reduced levels of oligomerized αSyn in the striatum and *substantia nigra*.

## 1 Introduction

Prolyl oligopeptidase (PREP, EC 3.4.21.26, also POP or PEP) is a serine protease with endopeptidase activity, cleaving peptides up to ca 30 amino acids after a proline residue.^1,2^ PREP is linked to several diseases and pathological processes such as neurodegenerative diseases, cancer, and inflammation (for a review, see Svarcbahs et al.^3^). Initially, it was suggested that PREP could regulate the degradation of proline-containing neuropeptides such as substance P, arginine-vasopressin, angiotensins, and thyrotropin-releasing hormone due to its ability to cleave them *in vitro* (for a review, see García-Horsman et al.^4^). However, the physiological effects of the mainly cytosolic PREP are more likely a result of direct protein–protein interactions (PPIs) with other proteins, such as α-synuclein (αSyn),^5^ Tau,^6^ protein phosphatase 2A (PP2A),^7^ and growth-associated protein 43,^8^ than of the cleavage of certain neuropeptides. PREP has been shown to induce αSyn aggregation *in vitro*,^9^ and we have previously reported that PREP accelerates αSyn and Tau aggregation via PPIs.^5,6^ PREP also forms a complex with PP2A,^7^ which inhibits PP2A activity leading to decreased autophagy and increased reactive oxygen species (ROS) production.^10^ Together, increased ROS production and αSyn aggregation accompanied by impaired autophagy create a vicious cycle that leads to neuronal cell death in Parkinson’s disease (PD). Moreover, decreased PP2A levels and activity have been implicated in the pathophysiology of Alzheimer’s disease and PD,^11,12^ and a recent study identified point mutations in an endogenous PP2A activator, leading to reduced PP2A activity, proposed to contribute to early onset PD.^13^ Therefore, PP2A activating compounds could tackle several pathophysiological mechanisms in neurodegenerative diseases and eventually lead to a disease-modifying effect.

**Figure 1:**
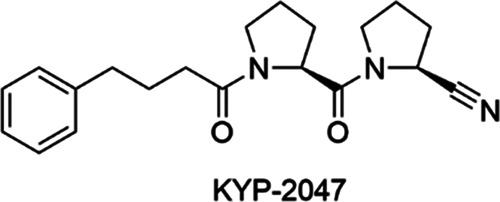
Peptide-like PREP inhibitor KYP-2047.

The development of PREP ligands was originally focused on inhibiting proteolytic activity, and a few potent inhibitors entered clinical trials.^14−16^ These trials did not advance, to our understanding, due to a lack of efficacy, but they showed that at least short-term PREP inhibition is safe in humans. Interestingly, the most recent compound that entered clinical trials, S17092, had no effect on the PPI-mediated functions of PREP when it was later evaluated in our assays.^17^ The well-known peptide-like PREP inhibitor KYP-2047 (Figure 1) differs from S17092 in that it is also able to modulate the PPIs of PREP.^17^ KYP-2047 has been shown to reduce αSyn aggregation and enhance autophagy to degrade αSyn aggregates in several *in vitro* and *in vivo* PD models,^18−22^ and most recently, to reduce Tau aggregates *in vitro* and *in vivo*.^6^ In addition, it is able to decrease ROS production in cells and PD and frontotemporal dementia mouse models.^6,10,23^

By studying different PREP inhibitors, we have demonstrated that the structure–activity relationships (SARs) for inhibition of the proteolytic activity and modulation of PPI-mediated functions, αSyn dimerization and autophagy, are disconnected in the way that some weak PREP inhibitors are highly effective modulators of the PPIs and some highly potent inhibitors do not affect the PPIs at all.^17,24,25^ In an αSyn-dimerization assay performed with PREP knock-out (PREP-KO) cells, the effect of all PREP inhibitors on αSyn dimerization was lost, demonstrating that their effect is the result of an interaction with PREP.^17^ As PREP is a highly dynamic protein and inhibitor binding has been reported to restrict its conformational freedom,^26,27^ our hypothesis is that the functions of PREP are dependent on what conformations PREP can adopt, and these dynamic features can be regulated differently by different ligands.

**Scheme 1:**
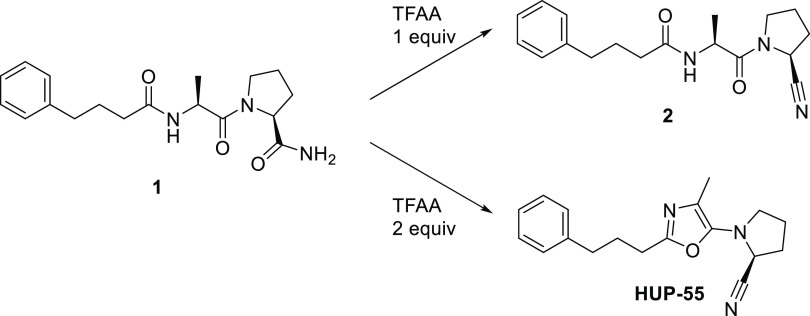
Dehydration of Compound **1**^,^ The major product is determined by the amount of trifluoroacetic anhydride (TFAA). Reagents and conditions: TFAA, Et_3_N, tetrahydrofuran (THF), 0 °C, 2 h.

We recently reported a series of peptide-like PREP inhibitors with a tetrazole ring in the position of the typical electrophilic group.^24^ We now report that the dehydration of compound **1** to the corresponding nitrile **2** in that paper also gave a minor side product where the peptide backbone had dehydrated to the nonpeptidic oxazole **HUP-55** (Scheme 1). Although some 2,4-dialkyl-substituted 5-aminooxazoles display decreased stability,^28^ this compound with a 2-cyanopyrrolidine group as the 5-amino group was readily isolated. **HUP-55** was tested for its inhibition of the proteolytic activity of PREP, and to our surprise, it was a nanomolar inhibitor, although it lacked the two important carbonyl groups described as critical for inhibitors of PREP.^29−31^ To our knowledge, this is the first low nanomolar PREP inhibitor lacking both important carbonyl groups, as the only previous successful attempt has only one of the important carbonyl groups replaced by a carbonyl-mimicking pyridine ring.^32^ Herein, we report the discovery of **HUP-55** and other new oxazole-based PREP inhibitors together with their biological activities in three cellular assays measuring pathophysiological mechanisms present in neurodegenerative diseases and *in vivo* in mouse models of PD.

## 2 Results and Discussion

### 2.1 Chemistry

Synthesis of the peptidic starting materials (Table 1) for oxazole formation by dehydration with trifluoroacetic anhydride (TFAA) was performed similarly to earlier reported procedures,^24^ applying typical amide bond formation reactions for peptides. **HUP-55** was synthesized from compound **1** according to Scheme 1 and Table 1. The dehydration reaction was optimized by increasing the amount of TFAA to a minimum of 2 equiv to obtain the oxazole as the main product (Scheme 1).

**Table 1:**
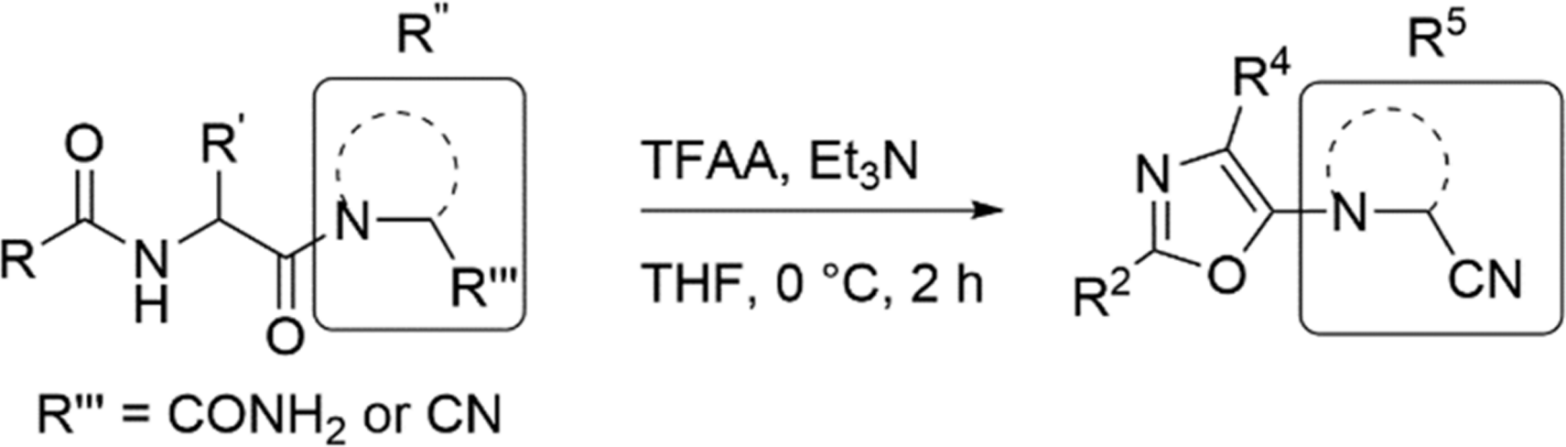
Peptidic Starting Materials and the General Dehydration Reaction Used for Oxazole Synthesis

starting material	R	R′	R″	product^a^
**1**	Ph(CH_2_)_3_-	CH_3_	l-prolinamide	**HUP-55**
**34**	Ph(CH_2_)_3_-	CH_3_	d-prolinamide	**3**
**35**	Ph(CH_2_)_3_-	CH_3_	(*S*)-piperidine-2-carboxamide	**4**
**36**	Ph(CH_2_)_3_-	CH_3_	NH(CH_2_CN)	**5**
**37**	Ph(CH_2_)_3_-	CH_3_	N(CH_2_CN)(CH_3_)	**6**
**38**	Ph(CH_2_)_3_-	-CH(CH_3_)_2_	l-prolinamide	**7**
**39**	Ph(CH_2_)_3_-	-CH_2_CH(CH_3_)_2_	l-prolinamide	**8**
**40**	Ph(CH_2_)_3_-	-C(CH_3_)_3_	l-prolinamide	**9**
**41**	Ph(CH_2_)_3_-	Ph	l-prolinamide	**10**
**42**	Ph(CH_2_)_3_-	-(CH_2_)_2_SCH_3_	l-prolinamide	**11**
**43**	Ph(CH_2_)_3_-	H	l-prolinamide	**12**
**44**	Ph	CH_3_	l-prolinamide	**13**
**45**	PhCH_2_-	CH_3_	l-prolinamide	**14**
**46**	Ph(CH_2_)_2_-	CH_3_	l-prolinamide	**15**
**47**	Ph(CH_2_)_4_-	CH_3_	l-prolinamide	**16**
**48**	PhO(CH_2_)_2_-	CH_3_	l-prolinamide	**17**
**49**	4-methoxyphenyl-(CH_2_)_3_-	CH_3_	l-prolinamide	**18**
**50**	3,4-methoxyphenyl-(CH_2_)_3_-	CH_3_	l-prolinamide	**19**
**51**	3,5-methoxyphenyl-(CH_2_)_2_-	CH_3_	l-prolinamide	**20**
**52**	thien-2-yl-(CH_2_)_3_-	CH_3_	l-prolinamide	**21**
**53**	indol-3-yl-(CH_2_)_2_-	CH_3_	l-prolinamide	**22, 23**
**54**	azepan-1-yl-CO(CH_2_)_2_-	CH_3_	l-prolinamide	**24**

a Product substituents R^2^, R^4^, and R^5^ are reported in Table 2.

The analogue of **HUP-55** without the nitrile group could not be obtained by the TFAA dehydration reaction, and it was instead synthesized using another dehydration reaction^33^ (full experimental procedure in the Supporting Information) and successfully isolated, but exposure to mildly acidic conditions such as silica gel in flash chromatography significantly increased the degradation of the compound. Noticeable degradation also occurred in CDCl_3_ at room temperature overnight. We concluded that it was not stable enough for reliable results in biological assays.

Although we did not observe any stability problems for **HUP-55**, we needed to verify its stability. An NMR sample with **HUP-55** dissolved in DMSO-*d*_6_ was observed over 2 months, and a sample of **HUP-55** exposed to the conditions of the PREP inhibition assay was analyzed by MS. In theory, **HUP-55** could be hydrolyzed back to **2** (Scheme 1), which is a potent PREP inhibitor. Hydrolysis or any other decomposition of the oxazole ring was not observed in either of these stability studies (Figures S63 and S64). From this, we concluded that the nitrile group in the 2-position of the pyrrolidine ring has a strong stabilizing effect on the 5-aminooxazole structure. **HUP-55** is also configurationally stable, as no racemization was observed over time (Table S1).

As **HUP-55** was stable, a series of analogues **3**–**24** (Table 2) were synthesized according to Table 1. The dehydration reaction proceeded analogously with all amino acids except for glycine, where compound **12**, having a trifluoroacetyl group attached to the 4-position of the oxazole ring, was obtained. Furthermore, even weakly basic NH groups, such as that of indole in compound **23**, were trifluoroacetylated during the dehydration reaction. We routinely examined compounds after isolation for their long-term stability and excluded any compounds with even slight stability issues from biological assays (Figure S62).

**Table 2:**
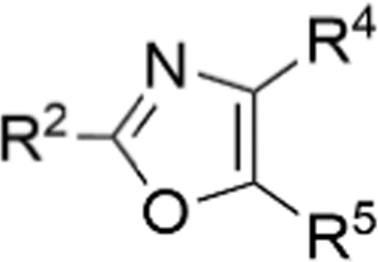
Structures and Biological Activities for the New Oxazoles^g^

compound	R^2^	R^4^	R^5^	IC_50_ (nM)^a^ (95% CI)	αSyn dimer. at 10 μM (%)^b^	autophagy at 10 μM (%)^c^	ROS at 10 μM (%)^d^
KYP-2047	-	-	-	<1^e^	87 ± 1	89 ± 3	88 ± 3
**2**	-	-	-	3.3 (2.0–5.3)	105 ± 14	86 ± 6	92 ± 7
**HUP-55**	Ph(CH_2_)_3_-	CH_3_	(*S*)-2-cyanopyrrolidin-1-yl	5.0 (3.2–7.6)	85 ± 2	87 ± 3	85 ± 3
**3**	Ph(CH_2_)_3_-	CH_3_	(*R*)-2-cyanopyrrolidin-1-yl	1660 (620–4500)	94 ± 8	85 ± 4	88 ±5
**4**	Ph(CH_2_)_3_-	CH_3_	(*S*)-2-cyanopiperidin-1-yl	n.a.	86 ± 19	97 ± 2	n.d.
**5**	Ph(CH_2_)_3_-	CH_3_	-N(CH_2_CN)(COCF_3_)	108 000 (33 000–433 000)	112 ± 6	77 ± 17	n.d.
**6**	Ph(CH_2_)_3_-	CH_3_	-N(CH_2_CN)(CH_3_)	12 400 (7600–20 000)	109 ± 6	96 ± 3	94 ± 7
**7**	Ph(CH_2_)_3_-	-CH(CH_3_)_2_	(*S*)-2-cyanopyrrolidin-1-yl	156 (100–240)	82 ± 13	82 ± 8	81 ± 5
**8**	Ph(CH_2_)_3_-	-CH_2_CH(CH_3_)_2_	(*S*)-2-cyanopyrrolidin-1-yl	4580 (2900–7300)	80 ± 6	81 ± 11	83 ± 7
**9**	Ph(CH_2_)_3_-	-C(CH_3_)_3_	(*S*)-2-cyanopyrrolidin-1-yl	415 000 (99 000–1 700 000)	92 ± 6	103 ± 3	86 ± 10
**10**	Ph(CH_2_)_3_-	Ph	(*S*)-2-cyanopyrrolidin-1-yl	17 400 (11 000–29 000)	97 ± 10	104 ± 6	77 ±8
**11**	Ph(CH_2_)_3_-	-(CH_2_)_2_SCH_3_	(*S*)-2-cyanopyrrolidin-1-yl	445 (330–590)	90 ± 8	92 ± 5	101 ± 8
**12**	Ph(CH_2_)_3_-	-COCF_3_	(*S*)-2-cyanopyrrolidin-1-yl	209 000 (150 000–310 000)	104 ± 5	98 ± 2	81 ± 10
**13**	Ph	CH_3_	(*S*)-2-cyanopyrrolidin-1-yl	7940 (4800–13 000)	80 ± 9	92 ± 1	86 ± 13
**14**	PhCH_2_-	CH_3_	(*S*)-2-cyanopyrrolidin-1-yl	288 (210–390)	99 ± 8	97 ± 6	84 ± 4
**15**	Ph(CH_2_)_2_-	CH_3_	(*S*)-2-cyanopyrrolidin-1-yl	692 (470–1000)	75 ± 4	88 ± 4	89 ± 1
**16**	Ph(CH_2_)_4_-	CH_3_	(*S*)-2-cyanopyrrolidin-1-yl	n.d.	108 ± 2	89 ± 6	n.d.
**17**	PhO(CH_2_)_2_-	CH_3_	(*S*)-2-cyanopyrrolidin-1-yl	1160 (570–2400)	120 ± 8	94 ± 6	70 ± 8
**18**	4-methoxyphenyl-(CH_2_)_3_-	CH_3_	(*S*)-2-cyanopyrrolidin-1-yl	65 (24–180)	100 ±5	98 ± 1	86 ± 6
**19**	3,4-methoxyphenyl-(CH_2_)_3_-	CH_3_	(*S*)-2-cyanopyrrolidin-1-yl	115 (80–170)	98 ± 17	93 ± 2	70 ± 9
**20**	3,5-methoxyphenyl-(CH_2_)_2_-	CH_3_	(*S*)-2-cyanopyrrolidin-1-yl	1293 (830–2000)	100 ± 9	89 ± 6	76 ± 8
**21**	thien-2-yl-(CH_2_)_3_-	CH_3_	(*S*)-2-cyanopyrrolidin-1-yl	18 (12–27)	101 ± 8	72 ±3	91 ± 6
**22**	indol-3-yl-(CH_2_)_2_-	CH_3_	(*S*)-2-cyanopyrrolidin-1-yl	890 (520–1500)	72 ± 2	97 ± 3	82 ± 11
**23**	*N*-COCF_3_-indol-3-yl-(CH_2_)_2_-	CH_3_	(*S*)-2-cyanopyrrolidin-1-yl	120 (57–250)	87 ± 11	94 ± 3	84 ± 7
**24**	azepan-1-yl-CO-(CH_2_)_2_-	CH_3_	(*S*)-2-cyanopyrrolidin-1-yl	654 (500–860)	117 ± 9	84 ± 5	90 ± 5
**25**	Ph(CH_2_)_2_CO-	CH_3_	pyrrolidin-1-yl	90 000^f^ (35 000–230 000)	110 ± 6	95 ± 10	105 ± 8
**26**	Ph(CH_2_)_3_-	CN	NH_2_	n.a.	73 ± 15	96 ± 4	n.d.
**27**	Ph(CH_2_)_3_-	CN	pyrrolidin-1-yl	75 400 (38 000–150 000)	88 ± 6	96 ± 5	n.d.
**28**	Ph(CH_2_)_3_-	CN	(*S*)-2-cyanopyrrolidin-1-yl	76 600 (23 000–250 000)	109 ± 5	97 ± 5	93 ± 11
**29**	Ph(CH_2_)_3_-	H	Ph	91 600 (21 000–400 000)	90 ± 6	90 ± 4	93 ± 11
**30**	Ph(CH_2_)_3_-	H	thien-2-yl	37 300 (17 000–81 000)	86 ± 8	98 ± 10	96 ± 9
**31**	Ph(CH_2_)_3_-	pyrrolidine-1-carbonyl	Me	138 000 (97 000–200 000)	117 ± 11	97 ± 4	n.d.
**32**	Ph(CH_2_)_3_-	(*S*)-2-carbamoylpyrrolidine-1-carbonyl	Me	n.a.	n.d.	98 ± 3	n.d.
**33**	Ph(CH_2_)_3_-	(*S*)-2-cyanopyrrolidine-1-carbonyl	Me	12 910 (8600–13 000)	109 ± 6	97 ± 5	98 ± 6

a Assessed using recombinant porcine PREP with Suc-Gly-Pro-AMC as the substrate.

b Luminescence signal percentage of DMSO control with SEM, assessed with a split *Gaussia* luciferase-based method using Neuro2A cells.

c GFP signal percentage of DMSO control with SEM, assessed using human embryonic kidney 293 cells stably expressing green fluorescent protein-tagged microtubule-associated proteins 1A/1B light chain 3B.

d Fluorescence signal percentage of DMSO control with SEM, assessed using a fluorogenic reactive oxygen species assay.

e The assay is limited by the enzyme concentration of 2 nM for IC_50_ values under this concentration; KYP-2047 is a slow, tight-binding inhibitor with a *K_i_*-value of 0.02 nM.^39,40^

f Measured with mouse brain homogenate.

g n.a.: no activity (>1 mM). n.d.: not determined.

**Scheme 2:**
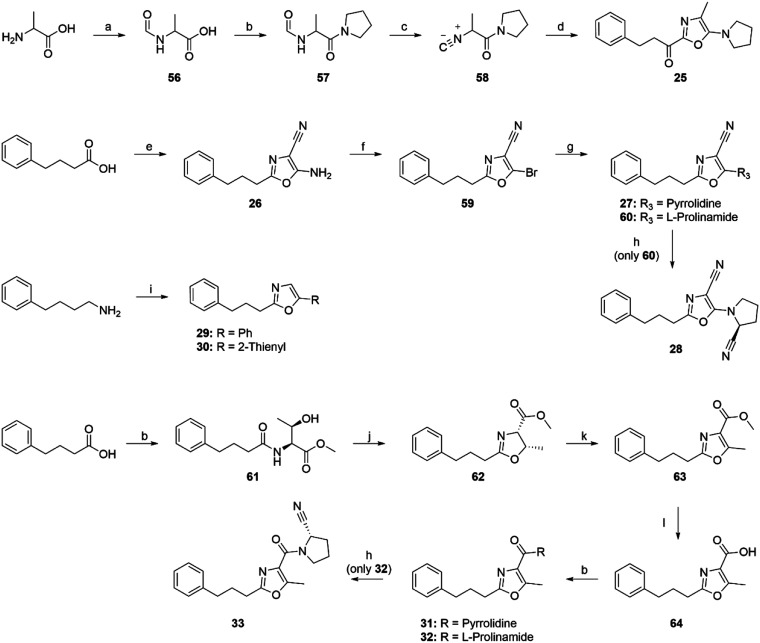
Synthetic Routes for Oxazoles that could not be Synthesized with the Main Synthetic Route Reagents and conditions: (a) Ac_2_O, HCO_2_H, 0 °C, 4 days; (b) (1) pivaloyl chloride, Et_3_N, dichloromethane (DCM), 0 °C, 1 h (2) amine, Et_3_N, rt, 1 day; (c) (1) POCl_3_, Et_3_N, DCM, −25 °C, 2 h (2) NaHCO_3_, rt, 16 h; (d) 3-phenylpropionyl chloride, Et_3_N, DCM, rt, 2 h; (e) aminomalonitrile *p*-toluenesulfonate, *N*,*N*-dimethylformamide (DMF), 120 °C, 30 min; (f) CuBr_2_, *t*-butyl nitrite, MeCN, rt, 19 h; (g) pyrrolidine, Pd(OAc)_2_, BINAP, KO-*t*-Bu, 1,4-dioxane, 170 °C, 15 min; (h) TFAA, Et_3_N, THF, 0 °C, 2 h; (i) aldehyde, CuBr_2_, K_2_CO_3_, pyridine, toluene, 80 °C, 18 h; (j) Burgess reagent, THF, rt, 16 h; (k) CCl_4_, DBU, pyridine, MeCN, rt, 16 h; and (l) LiOH, MeOH, H_2_O, rt, 16 h.

To further explore the scaffold, we also synthesized compounds having an electron-withdrawing substituent attached directly to the oxazole ring or replacing the 5-amino group with an aryl group. The electron-withdrawing group was introduced as an acyl group to the 2-position or a nitrile group to the 4-position of the oxazole ring, resulting in compounds **25** and **27**, respectively. These were synthesized according to previously reported methods for similar compounds (Scheme 2).^34−36^ Compounds where the 5-amino substituent on the oxazole ring was replaced by an aryl group, compounds **29** and **30**, were synthesized with a single step from an amine and an aldehyde (Scheme 2).^37^ However, this reaction did not allow the introduction of a substituent at the 4-position. Compounds **31**, **32**, and **33** had the substituents in positions 4 and 5 switched compared to the other oxazoles, and the amine substituent was connected via a carbonyl group resulting in an amide bond (Scheme 2).^38^

### 2.2 Biological Activity

#### 2.2.1 Inhibition of the Proteolytic Activity

The inhibitory activities of the oxazoles were first assessed. The IC_50_ values were determined using recombinant porcine PREP and a fluorescence-based method with Suc-Gly-Pro-AMC as the substrate. The results are presented in Table 2. **HUP-55** had an IC_50_ value of 5 nM. The stereochemistry of **HUP-55** was important as its enantiomer, compound **3**, had an IC_50_ value of only 1660 nM. Replacing the pyrrolidine ring at R^5^ with a piperidine ring resulted in an inactive compound **4**, and the opening of the ring structure resulted in compounds **5** and **6** with very low inhibitory activities. Compounds **7** and **11** having an isopropyl and a 2-(methylthio)ethyl group, respectively, at R^4^ were still fairly active inhibitors with IC_50_ values in the range 156–445 nM. However, compound **8** with an isobutyl group at R^4^ had a weaker IC_50_ value of 4580 nM, and compounds **9** and **10** with even bulkier substituents in this position had further reduced inhibitory activities. Compound **12** with a bulky and electron-withdrawing trifluoroacetyl group at R^4^ also had a strongly reduced inhibitory activity. Compounds **13**, **14**, and **15** with truncated linkers comprising 0, 1, and 2 CH_2_ groups at R^2^ had IC_50_ values of 7940, 288, and 692 nM, respectively. Compound **17**, where the CH_2_ group in the linker adjacent to the phenyl group was replaced by an oxygen atom, also had a significantly reduced inhibitory activity with an IC_50_ value of 1160 nM. Methoxy substituents could be added to the phenyl group linked to R^2^; however, all modifications reduced the inhibitory activity, and the shortening of the linker did not compensate for the increased size caused by the added methoxy substituents. The resulting compounds **18**, **19**, and **20** had IC_50_ values in the range 65–1293 nM. Other aryls were also studied linked to R^2^, and a thienyl group gave a potent compound **21** with an IC_50_ value of 18 nM. This was the only compound in the series that was equipotent to **HUP-55**. The indolyl group was studied with one CH_2_ group shorter linker to compensate for the larger size, and the resulting compounds **22** and **23** had IC_50_ values in the range 120–654 nM. Interestingly, replacing the phenyl group with a nonaromatic azepanyl group attached to the linker via an amide bond did not remove the inhibitory activity completely, as the resulting compound **24** had an IC_50_ value of 654 nM. Adding an electron-withdrawing group as a substituent at R^2^ or R^4^ increases the stability and also allows an unsubstituted pyrrolidine ring at R^5^. A carbonyl group in the linker next to the oxazole ring at R^2^ and an unsubstituted pyrrolidine ring at R^5^ gave compound **25**, which was a very weak inhibitor, indicating that the additional carbonyl group is not preferred next to the oxazole ring at R^2^; however, the unsubstituted pyrrolidine at R^5^ might also lower inhibitory activity of this compound. A nitrile group, which should not be too bulky, at R^4^ gave compounds **27** and **28**, which were also very weak inhibitors, indicating that an electron-withdrawing substituent was not allowed at R^4^. Compounds **29** and **30** having a phenyl and a thienyl group, respectively, as the R^5^ substituent and lacking an alkyl substituent at R^4^ both had strongly reduced inhibitory activities. Introducing a carbonyl group to attach the pyrrolidine ring to the oxazole ring as an amide and switching the R^4^ and R^5^ substituents resulted in compounds **31**, **32**, and **33** with very low inhibitory activities. A molecular modeling study was performed where **HUP-55** and some close analogues were docked into the active site of PREP. The postulated binding mode supports the observed SAR for inhibition of the proteolytic activity of PREP (Figures S65 and S66).

#### 2.2.2 Impact of Oxazoles on αSyn Dimerization, Autophagy, and Oxidative Stress

αSyn dimerization, which initiates αSyn aggregation, was assessed with a split *Gaussia* luciferase-based method using Neuro2A (N2A) cells,^5^ where αSyn is allowed to dimerize for 48 h before incubation with the test compounds (Table 2; Figure S67A). Proteasomal inhibitors, lactacystin (10 μM) and MG-132 (10 μM), were used as positive controls to induce αSyn dimerization, and KYP-2047 (10 μM) was used as a reference PREP ligand. Oxazoles were used at 10 μM concentrations based on earlier studies.^17,41^ KYP-2047, which has been shown to reduce αSyn aggregation in cells and *in vivo*,^19^ reduces the luminescence signal to 87% of the DMSO control in this assay, and based on this, a compound reducing the signal to this level can be considered active. Compounds **15**, **22**, and **26** were the most effective ones, decreasing the signal to at least 75% compared to the control, and compounds **HUP-55**, **4**, **7**, **8**, **13**, and **30** were also more effective than the reference compound KYP-2047 (not significantly), decreasing the signal to 80–85% compared to the control. Compounds **11**, **23**, and **27** had a comparable effect as KYP-2047. As expected, lactacystin and MG-132 significantly increased αSyn dimerization (*p* < 0.01 and *p* < 0.05, respectively; one-way analysis of variance (ANOVA) with Dunnett’s post-test), but with the oxazoles, no significant differences compared to the DMSO control were seen (*F*_34,109_ = 5.835, *p* < 0.0001; one-way ANOVA).

Autophagic flux was assessed using human embryonic kidney 293 (HEK-293) cells stably expressing green fluorescent protein-tagged microtubule-associated proteins 1A/1B light chain 3B (GFP-LC3B) (Table 2; Figure S67B). Bafilomycin A1 (20 nM) was used as an autophagy inhibitor, while rapamycin (500 nM) and serum starvation served as positive controls for autophagy induction. Oxazoles were used at 10 μM concentrations based on earlier studies^17,41^ and KYP-2047 (10 μM) was used as a reference. It should be noted that in this assay, even a small decrease in the GFP signal indicates an increased autophagic flux as 500 nM rapamycin, a classical autophagy activator via mammalian target of rapamycin (mTOR) inhibition, decreased the signal to 65% of the DMSO control. Rapamycin is considered a highly potent autophagy inducer, and it is reported to augment cell death via an apoptotic pathway in some cases, and therefore, a decrease of the signal to 65% is practically the maximal effect in this assay.^42,43^ Additionally, KYP-2047, which can induce autophagy *in vivo*,^22^ decreases the signal to 89% in this assay. Compounds **HUP-55**, **3**, **5**, **7**, **8**, **15**, **21**, and **24** outperformed KYP-2047, decreasing the signal to at least 88% (differences between compounds were nonsignificant). Compound **21** significantly decreased the signal compared to the DMSO control (*p* < 0.05; one-way ANOVA with Dunnett’s post-test), but for the other listed compounds, the observed decrease compared to the control was not statistically significant (*F*_37,185_ = 7.494, *p* < 0.0001; one-Way ANOVA). Similar to αSyn dimerization, positive controls showed a significant effect in the GFP signal compared to the DMSO control (starvation, rapamycin, bafilomycin A1; *p* < 0.01, *p* < 0.001 and *p* < 0.001, respectively; one-way ANOVA with Dunnett’s post-test).

The effect of selected oxazoles on ROS production during oxidative stress (OS) was assessed using a fluorogenic ROS assay (Table 2; Figure S67C) as reported in a study by Eteläinen et al.^10^ Cells were stressed with H_2_O_2_ and FeCl_2_ (Fenton reaction) and incubated with the compounds (10 μM) for 3 h. **HUP-55** was also tested at 1 μM concentration. Reference compound KYP-2047 at 1 and 10 μM concentrations were used as controls. The decrease in ROS production was then compared to a normalized value of ROS production in DMSO-treated cells (*F*_30,211_ = 15.16, *p* < 0.0001; one-way ANOVA with Dunnett’s post-test). A significant decrease in the ROS production compared to vehicle-treated cells was observed with 10 μM KYP-2047 (88% of the control, *p* = 0.0494), **HUP-55** (85% of the control, *p* = 0.002), compound **10** (75% of the control, *p* = 0.042), and compounds **17** and **19** (70% of the control, *p* = 0.0075 and *p* = 0.0066, respectively). However, several other oxazoles **7**, **8**, **9**, **12**, **13**, **14**, **18, 20**, **22**, and **23** also outperformed KYP-2047 by decreasing the ROS production below 88% of the control (differences between compounds were nonsignificant).

Compounds **HUP-55**, **7**, **8**, and **15** were all equally potent in both the αSyn and autophagy assays. The IC_50_ values, on the other hand, varied from 5 nM to 5 μM for the same compounds. The drug-like properties were predicted using QikProp (Table S2).^44^ Compounds **7** and **8** were on the upper limit in lipophilicity for drug molecules (QPlogPo/w = 4.9 and 5.1, QPlogS = −7.0 and −7.1, respectively). The choice for selecting one compound for further studies was therefore between **HUP-55** (QPlogPo/w = 4.0, QPlogS = −6.1) and **15** (QPlogPo/w = 3.5, QPlogS = −5.3). Looking at the SARs for the evaluated functions, the inhibitory activity was less tolerant than the PPI-derived effects to a slight increase in the bulkiness of the 4-alkyl group on the oxazole and shortening of the linker between the aromatic rings by one carbon atom. As **HUP-55** was the more potent inhibitor of proteolytic activity, it was chosen for further studies.

#### 2.2.3 Specificity of **HUP-55** and the Effect on PP2A Levels and Autophagy

**Figure 2:**
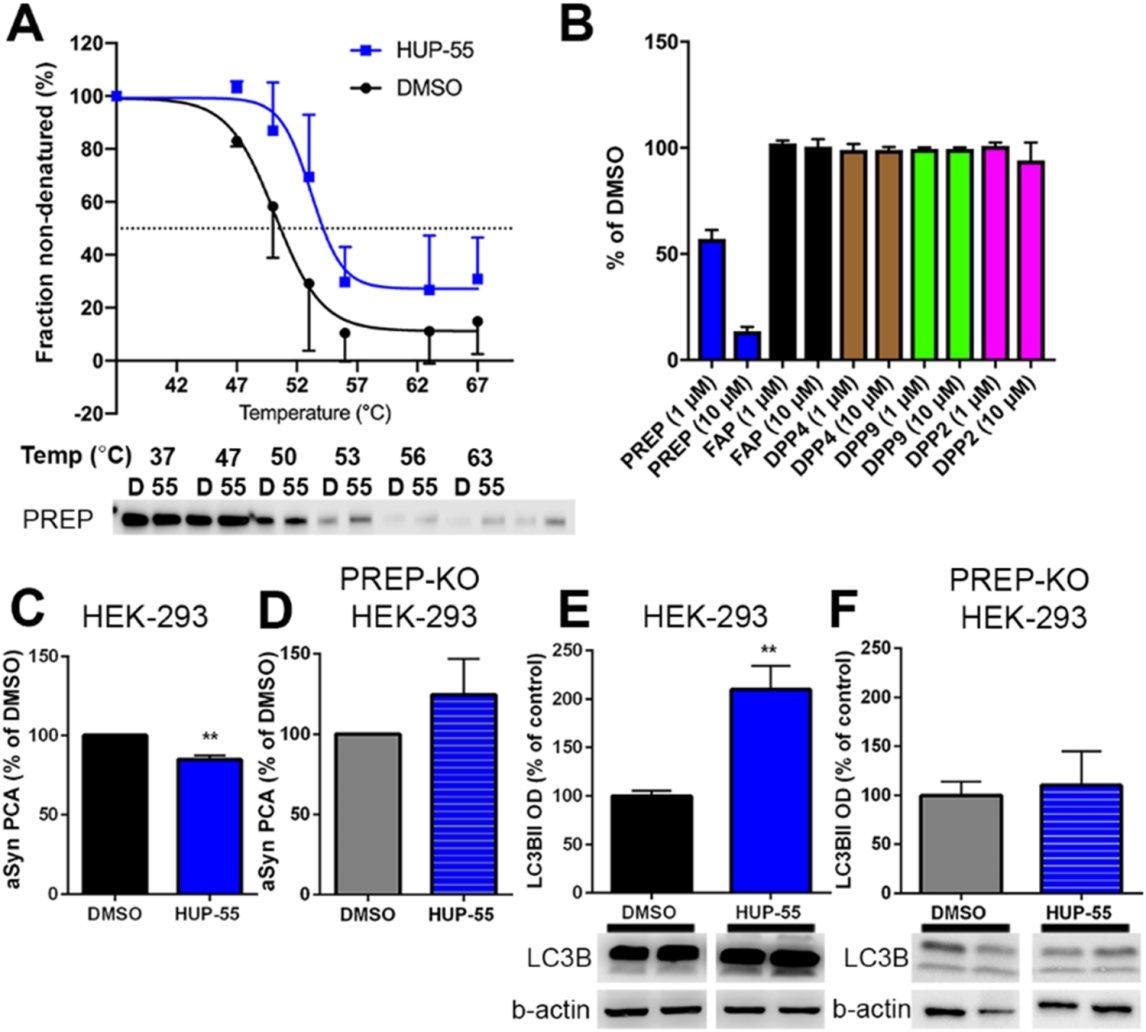
Cellular thermal shift assay (CETSA) shows target engagement for 10 μM **HUP-55** on PREP in HEK-293 cells (A). **HUP-55** at 1 or 10 μM concentration does not inhibit close relative enzymes FAP, DPP4, DPP9, or DPP2 (B). In HEK-293 cells, **HUP-55** decreases αSyn dimerization (C), but when PREP is not expressed (PREP knock-out (KO) HEK-293 cells), no such effect is seen (D). A similar effect was seen in autophagy when assayed with LC3BII protein levels in HEK-293 (E) and PREP-KO HEK-293 cells (F), indicating a PREP-mediated effect. Data are presented as mean + SEM. **, *p* < 0.01; student’s *t*-test.

Target engagement was tested using the cellular thermal shift assay (CETSA; Figure 2A). Ten micromolar **HUP-55** caused a 3.01 °C increase in the fitted mean aggregation temperatures (DMSO: 50.18 °C; HUP-55: 53.19 °C), which is similar to the results of PREP inhibitors tested in the study by Hellinen et al.,^41^ indicating an interaction between **HUP-55** and PREP. The specificity of **HUP-55** was tested on close-relative enzyme fibroblast activating protein (FAP) and dipeptidyl peptidase (DPP) 2, 4, and 9, but no inhibition of these enzymes was seen with 1 or 10 μM doses (Figure 2B). Additionally, PREP-specific effects of **HUP-55** were confirmed by using HEK-293 PREP-KO cells (Figure 2C–F). In the αSyn-dimerization assay, **HUP-55** had no effect on αSyn dimerization in PREP-KO cells (Figure 2C,D), and similarly, the levels of the autophagosome marker LC3BII were not altered by **HUP-55** in PREP-KO cells (Figure 2E,F). As PP2A modulation is potentially toxic for the cells, we also tested the impact of **HUP-55** and other selected compounds from the series on cell viability of cell cultures (HEK-293 and SH-SY5Y) and on mouse primary neurons. None of the compounds caused any toxicity, even with high concentrations (100 μM; Figure S68).

**Figure 3:**
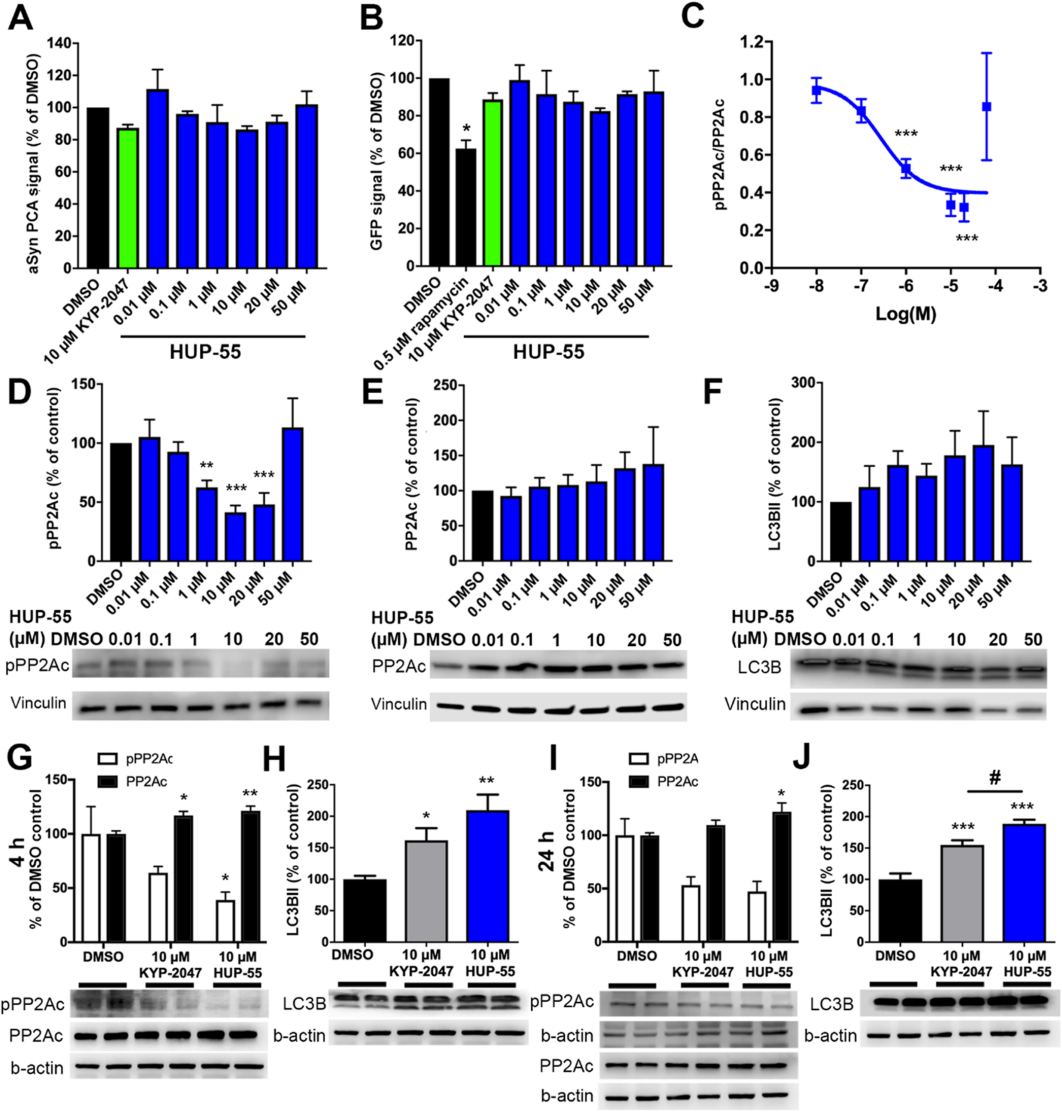
Concentration–response of **HUP-55** in the αSyn PCA assay (A) and on GFP-LC3B autophagy reporter cells (B) show that 10 μM **HUP-55** is the most effective concentration. Four hours of incubation with **HUP-55** significantly decreased the ratio between Tyr307 phosphorylated (inactive) protein phosphatase 2A catalytic subunit levels (pPP2Ac) and total PP2Ac, indicating activated PP2A at 1, 10, and 20 μM **HUP-55** concentrations when assayed with Western blot but not at 50 μM (C). Representative bands of pPP2Ac and total PP2Ac are presented in D and E. Increased levels of autophagosome marker LC3BII were seen but without significant results (F). Additionally, the effects of **HUP-55** and KYP-2047 (both at 10 μM) on pPP2A and PP2Ac levels (G and I) and LC3BII levels (H and J) were compared in HEK-293 cells after 4 and 24 h treatment. **HUP-55** had a more significant impact on all markers compared to KYP-2047 in 4 h treatment and outperformed KYP-2047 on autophagy activation and total PP2A levels in 24 h treatment. Data are presented as mean + SEM. #, *, *p* < 0.05; **, *p* < 0.01; and ***, *p* < 0.001; one-way ANOVA with Dunnett’s (A–D) or Tukey’s (E–L) post-hoc test.

As it had been shown that inhibition of the proteolytic activity of PREP does not correlate directly with the effects on PPI-derived functions, it was important to verify that **HUP-55** has a concentration-dependent effect on PP2A activation and autophagy. The concentration–response was tested with concentrations of 0.01, 0.1, 1, 10, 20, and 50 μM in the αSyn protein-fragment complementation assay (PCA) and GFP-LC3B autophagy reporter cells (Figure 3A,B). In both assays, 10 μM **HUP-55** showed the best efficacy, with no further impact being achieved with 20 or 50 μM concentrations, but interestingly, the signal returned toward the negative control levels, particularly with 50 μM.

To further study the efficacy of **HUP-55** on the PPI-related functions of PREP, the concentration-dependent impact of **HUP-55** on inactive, Tyr307 phosphorylated PP2Ac (pPP2Ac; specificity of the antibody for inactive PP2A has been previously verified^6,7^), total PP2Ac, and LC3BII, an autophagosome marker, was tested in HEK-293 cells by Western blot (WB) after 4 h incubation. The 4 h time point had been earlier shown to be optimal for PREP inhibitor-mediated autophagy activation.^7^ The ratio between inactive pPP2Ac and total PP2Ac, which can be used as an indicator of PP2A activation, was also significantly decreased after 1 μM **HUP-55** treatment (Figure 3C; *F*_6,44_ = 16.99, *p* < 0.0001; *p* < 0.001 DMSO vs 1, 10, and 20 μM **HUP-55**; one-way ANOVA with Dunnett’s post-test). Based on nonlinear curve fitting, the EC_50_ value for PP2A activation (pPP2Ac/PP2Ac ratio) was 275 nM. Inactive pPP2Ac alone was also significantly decreased already at 1 μM **HUP-55** concentration (*F*_6,44_ = 11.44, *p* < 0.0001; *p* < 0.01 DMSO vs 1 μM **HUP-55**; *p* < 0.001 DMSO vs 10 and 20 μM **HUP-55**; one-way ANOVA with Dunnett’s post-test), and no additional effect was seen after 10 μM of **HUP-55** treatment (Figure 3D). A nonsignificant increase was seen in total PP2A catalytic subunit (PP2Ac) levels after **HUP-55** incubation (Figure 3E). LC3BII levels showed a nonsignificant increase as the concentration of **HUP-55** was increased from 0.1 to 10 μM, but no further effect was seen with 20 or 50 μM (Figure 3F). No effect on PP2A markers was seen with the 0.01 or 0.1 μM concentration, which supports our earlier findings of disconnected SARs for inhibition of the proteolytic activity and the PPI-derived functions. Interestingly, in pPP2A, PP2A, and LC3B, the effect of the 50 μM concentration was close to the negative control, suggesting a protective mechanism for excessive PP2A activation.

We also wanted to compare the efficacy of **HUP-55** with the well-known PREP inhibitor KYP-2047 in the cellular assays. Based on the concentration–response (for that of KYP-2047 on PP2A, see Svarcbahs et al.^7^), 10 μM was selected for the assays. LC3B, PP2Ac, and pPP2A were immunoblotted after 4 and 24 h incubations with **HUP-55** and KYP-2047. A 4 h incubation with 10 μM **HUP-55** caused a significant decrease in pPP2Ac levels (Figure 3G; pPP2Ac: *F*_2,8_ = 5.524, *p* = 0.0311; *p* < 0.05 DMSO vs **HUP-55**; one-way ANOVA with Tukey’s post-test). Both KYP-2047 and **HUP-55** elevated total PP2Ac levels (Figure 3G; *F*_2,20_ = 9.478, *p* = 0.0013; *p* < 0.05 DMSO vs KYP-2047; *p* < 0.01 vehicle vs **HUP-55**; one-way ANOVA with Tukey’s post-test), but the pPP2Ac/PP2Ac ratio was only significantly modified by **HUP-55** (**HUP-55**: 0.32 ± 0.01; KYP-2047: 0.55 ± 0.09; *F*_2,8_ = 7.412, *p* = 0.0151; *p* < 0.05 DMSO vs **HUP-55**) Additionally, both compounds caused a significant increase in LC3BII levels (Figure 3H; *F*_2,27_ = 8.850, *p* = 0.0011; *p* < 0.05 DMSO vs KYP-2047; *p* < 0.01 DMSO vs **HUP-55**; one-way ANOVA with Tukey’s post-test). pPP2Ac levels were not significantly decreased after 24 h incubation (Figure 3I), while PP2Ac levels were significantly elevated by **HUP-55** but not with KYP-2047 (Figure 3I; *F*_2,11_ = 4.453, *p* = 0.0383; *p* < 0.05 vehicle vs **HUP-55**; one-way ANOVA with Tukey’s post-test). However, the ratio between pPP2Ac and PP2Ac was significantly increased, both with KYP-2047 and **HUP-55** (**HUP-55**: 0.39 ± 0.11; KYP-2047: 0.49 ± 0.12; *p* < 0.05 vehicle vs **HUP-55** or KYP-2047; one-way ANOVA with Tukey’s post-test). Both **HUP-55** and KYP-2047 also significantly elevated LC3BII levels, with **HUP-55** having a larger effect (Figure 3J; *F*_2,17_ = 31.09, *p* < 0.0001; *p* < 0.001 DMSO vs **HUP-55** or KYP-2047; *p* < 0.05 KYP-2047 vs **HUP-55**; one-way ANOVA with Tukey’s post-test).

#### 2.2.4 HUP-55 Penetrates the Blood–Brain Barrier

**Figure 4:**
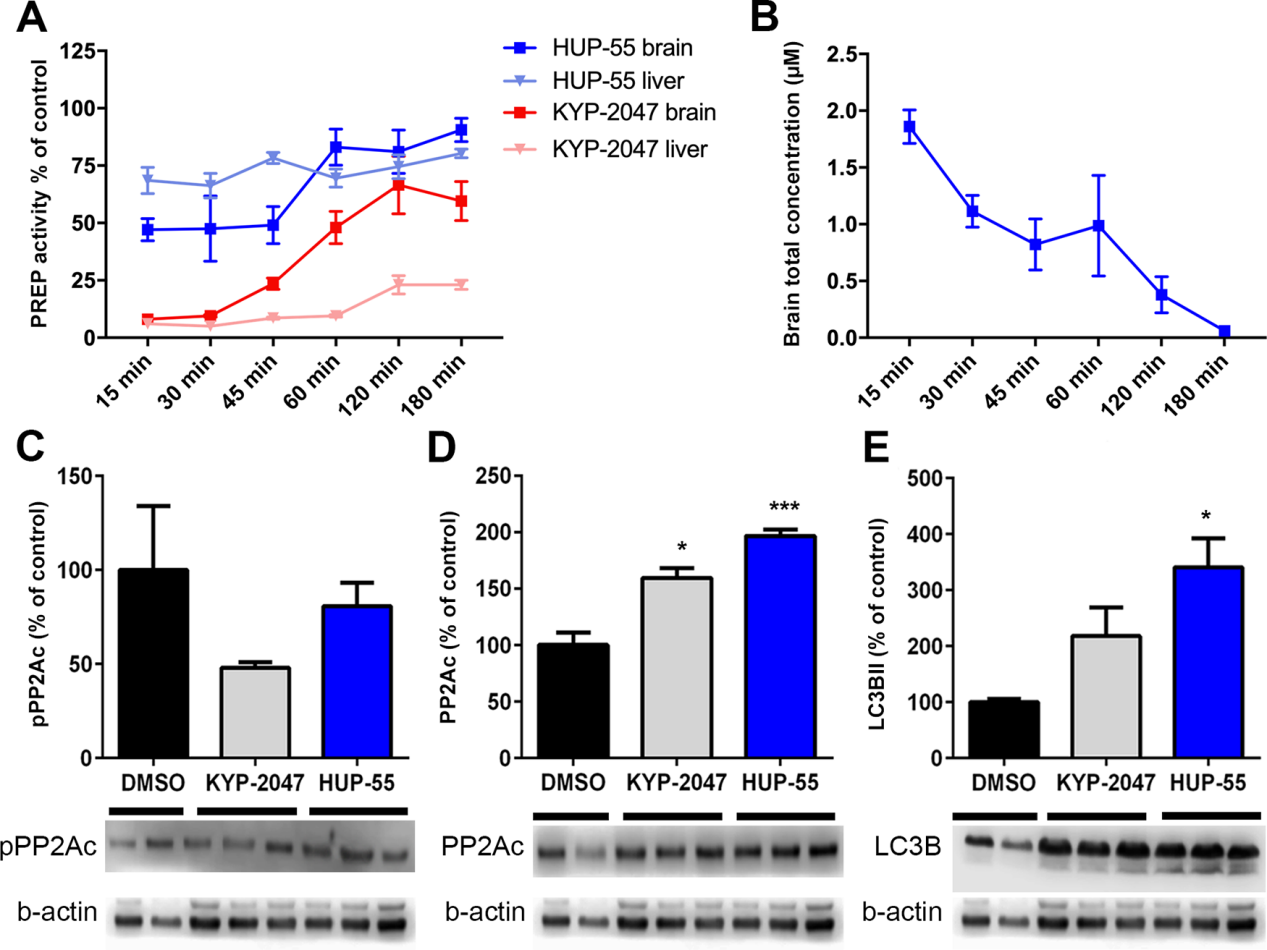
(A) PREP activity was measured from mouse brain and liver tissue samples after 10 mg/kg i.p. injection of KYP-2047 or **HUP-55** and was compared to vehicle-injected mice. As expected, KYP-2047 inhibited PREP more potently than **HUP-55** both in the brain and liver. (B) Brain penetration was verified by LC-MS analysis that revealed that **HUP-55** rapidly passed the blood–brain barrier. The effect of **HUP-55** and KYP-2047 on the levels of brain pPP2A (C), PP2A (D), and LC3B (E) was measured 45 min after i.p. injection. **HUP-55** and KYP-2047 did not have a significant effect on pPP2Ac (D) but significantly increased total PP2A and LC3BII levels (C-E). Data are presented as mean + SEM. #, *, *p* < 0.05; **, *p* < 0.01; and ***, *p* < 0.001; one-way ANOVA with Tukey’s post-hoc test (*n* = 2–4).

Brain penetration of **HUP-55** was assessed by measuring PREP activity from mouse brain tissue lysates after intraperitoneal (i.p., 10 mg/kg) administration of the compound, and KYP-2047 was used as a reference. **HUP-55** decreased brain PREP activity by 50% compared to the DMSO control during the first 45 min, while KYP-2047 diminished the activity almost completely during the first 30 min. However, at the end of the 3 h monitoring period, PREP activity with **HUP-55** was restored to 80% of the control, while with KYP-2047, the activity was still reduced to 50%. In the liver, **HUP-55** decreased PREP activity by approximately 25% throughout the monitoring period, but KYP-2047 kept the activity below 25% of normal activity until the 180 min endpoint (Figure 4A). The brain penetration of **HUP-55** was verified with liquid chromatography–mass spectrometry (LC-MS) analysis. LC-MS revealed that **HUP-55** passed the blood–brain barrier very rapidly at high concentrations (1.86 μM at the 15 min time point), but the concentration of **HUP-55** was decreased by almost 50% at the 30 min time point (1.11 μM; Figure 4B). However, the elimination of **HUP-55** did not remain as rapid, and detectable amounts were still present in the brain at 180 min (Figure 4B). The calculated area under the curve (AUC_0–180 min_) for **HUP-55** was 104.6 (min × μM). Restoration of the enzymatic activity of PREP was slightly behind the decrease in the brain concentration of **HUP-55**. A similar impact has been seen with KYP-2047 in rodent brains.^45,46^ Possible metabolites of **HUP-55** were also identified from the brain by LC-MS (Table S3).

To assess if **HUP-55** can induce autophagy and activate PP2A in the mouse brain, we studied the levels of pPP2Ac, PP2Ac, and LC3BII with WB, using KYP-2047 as a reference. Both compounds decreased inactive pPP2Ac levels, but this was not significant (Figure 4C), and significantly increased the levels of PP2Ac in mouse brain (Figure 4D; *F*_2,13_ = 39.09, *p* = 0.0009; *p* < 0.05 vehicle vs KYP-2047; *p* < 0.001 vehicle vs **HUP-55**; one-way ANOVA with Tukey’s post-test). **HUP-55** and KYP-2047 significantly increased the ratio between total PP2A and pPP2A, suggesting that PP2A activity was increased (**HUP-55**: 0.41 ± 0.004; KYP-2047: 0.30 ± 0.03; *F*_2,6_ = 1.623, *p* = 0.0175; *, *p* < 0.05 DMSO vs KYP-2047 and **HUP-55**; one-way ANOVA with Tukey’s post-test). Moreover, the levels of LC3BII were significantly elevated by **HUP-55** 45 min after the i.p. injection (Figure 4E; *F*_2,13_ = 5.872, *p* = 0.0152; *p* < 0.01 vehicle vs **HUP-55**; one-way ANOVA with Tukey’s post-test).

#### 2.2.5 HUP-55 Reduces αSyn Oligomers in Mouse *Substantia Nigra* and Striatum after AAV-αSyn Injection and Attenuates Motor Impairment

**Figure 5:**
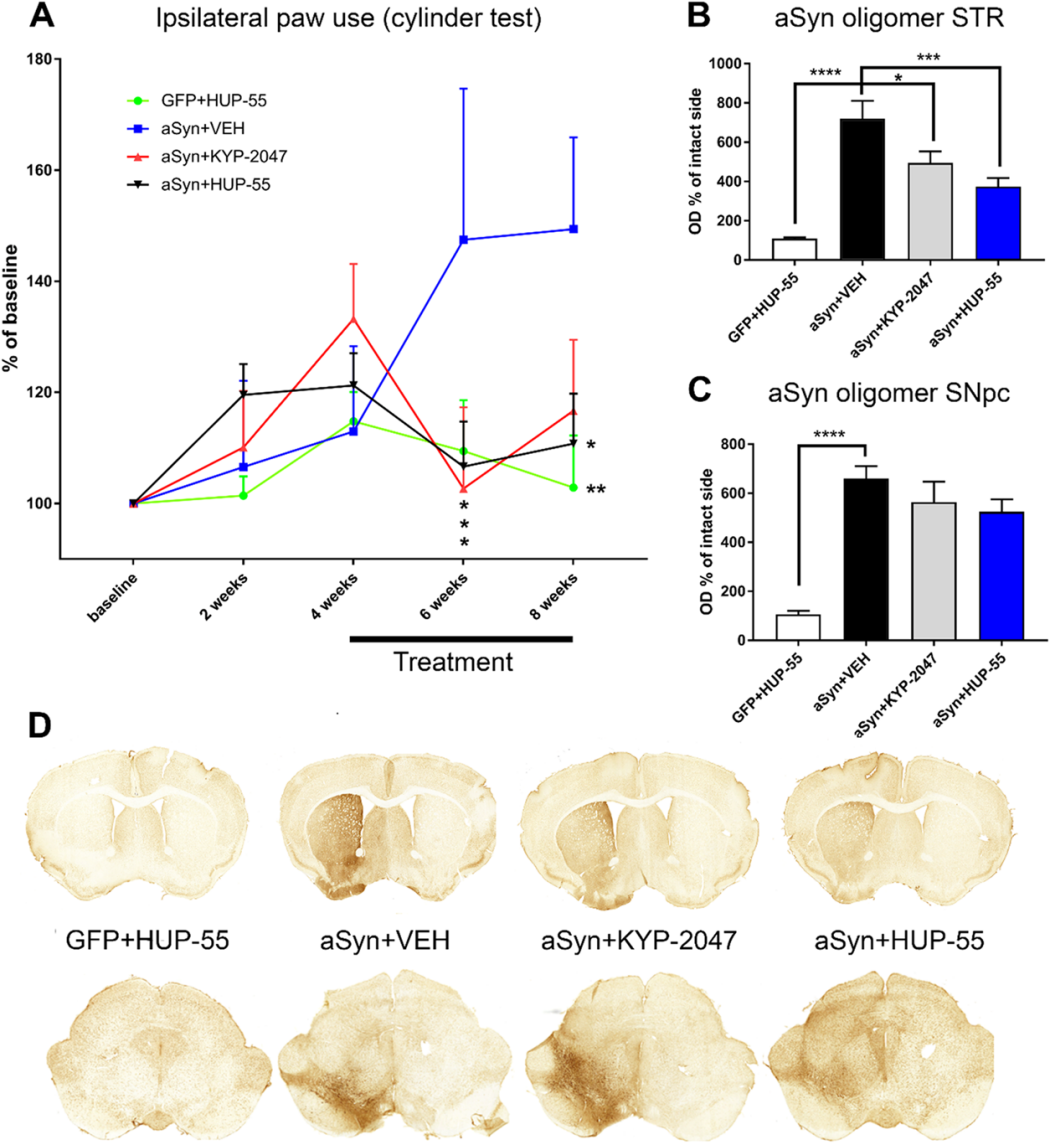
(A) At the 6-week time point, usage of the ipsilateral paw was significantly higher in the vehicle-treated AAV-αSyn-injected mice compared to the KYP-2047 or **HUP-55**-treated groups and the AAV-GFP-injected control mice. At the 8-week time point, the AAV-GFP and **HUP-55** groups had significantly decreased ipsilateral paw use compared to the vehicle treatment. (B, C) AAV-αSyn injection elevated the levels of immunoreactive oligomeric αSyn in the mouse striatum (STR) (B) and *substantia nigra pars compacta* (SNpc) (C). Both **HUP-55** and KYP-2047 significantly decreased oligomeric αSyn in the STR (B), but this was not seen in the SNpc (C). (D) Representative brain slices. (A) Data is presented as the mean of percentages from the baseline ± SEM, two-way ANOVA with Dunnett’s multiple comparisons, **p = 0.01 and *p < 0.05. *n* = 9 for the GFP group and *n* = 7 for VEH and *n* = 8 for **HUP-55** and KYP-2047 groups. (B, C) One-way ANOVA with Dunnett’s multiple comparison tests was used to compare treatments to the αSyn+VEH group, ****p < 0.0001, ***p = 0.0003, and *p = 0.0234. Data are presented as mean SEM.

The effect of **HUP-55** was evaluated using a PD mouse model based on unilateral AAV2-CBA-αSyn virus vector injection above the *substantia nigra pars compacta* (SNpc) as described in the study by Svarcbahs et al.^47^ In the cylinder test, vehicle-treated animals with nigrostriatal overexpression of αSyn developed a motor deficit 6 weeks after αSyn injection (Figure 5A. Interaction with two-way ANOVA, *F*_12,112_ = 1.993, *p* = 0.0311.). The treatment using **HUP-55** or KYP-2047 (i.p. Alzet minipump, 10 mg/kg/day) was initiated 4 weeks after AAV-αSyn injection based on the earlier study with KYP-2047, where similar administration led to a significant impact on brain αSyn levels and approximately 50% inhibition of brain PREP activity.^47^ Already after 2 weeks of treatment (6-week time point), both KYP-2047 and **HUP-55**-treated mice had significantly decreased their use of the ipsilateral forepaw compared to vehicle-treated AAV-αSyn-injected mice (Figure 5A; KYP-2047, *p* = 0.0108; HUP-55, *p* = 0.0225, two-way ANOVA with Dunnett’s Multiple Comparisons). At the endpoint (8 weeks post injection), statistically significant difference in ipsilateral paw use was observed between AAV-αSyn-injected vehicle and **HUP-55**-treated mice (*p* = 0.006; two-way ANOVA with Dunnett’s multiple comparisons) and AAV-αSyn-vehicle-treated and GFP-injected mice (*p* = 0.0331; two-way ANOVA with Dunnett’s multiple comparisons), pointing to successful disease-modifying treatment by **HUP-55** (Figure 5A). In locomotor activity measurements (Figures S69 and S70), no significant effects were seen between the treatment groups throughout the recording period. However, a unilateral model is not optimal for assessing locomotor dysfunction in mice.^48^

Optical density (OD) analysis of total oligomer-specific αSyn staining in the striatum (STR) and SNpc revealed significantly increased oligomeric αSyn immunoreactivity in αSyn-injected vehicle-treated animals, both in the STR (Figure 5B: *F*_3,31_ = 22.29, *p* < 0.0001, one-way ANOVA) and the SNpc (Figure 5C: *F*_3,33_ = 20.82, *p* < 0.0001, one-way ANOVA). In the STR, **HUP-55** (*p* = 0.0003, Dunnett’s multiple comparisons) and KYP-2047 (*p* = 0.0234, Dunnett’s multiple comparisons) decreased the OD of oligomer-specific αSyn compared to vehicle-treated mice (Figure 5B). However, in the SNpc, **HUP-55** and KYP-2047 did not significantly reduce oligomeric αSyn OD compared to the vehicle-treated group (Figure 5C). OD of total αSyn was also assessed from the STR, and the results were similar to oligomer-specific αSyn (Figure S71). Current results are in line with the earlier study by Svarcbahs et al.,^47^ showing that the accumulation of oligomeric αSyn, particularly in the STR, correlates with motor impairment in the cylinder test. αSyn aggregation impairs dopamine release by interfering with the physiological effects of αSyn in the SNARE complex and disturbs the functions of dopamine transporters in the synaptic cleft. This was seen *in vivo* in the study by Svarcbahs et al.,^47^ where AAV-αSyn injection and accumulation of oligomeric αSyn caused a significant reduction in extracellular dopamine in the STR.

**Figure 6:**
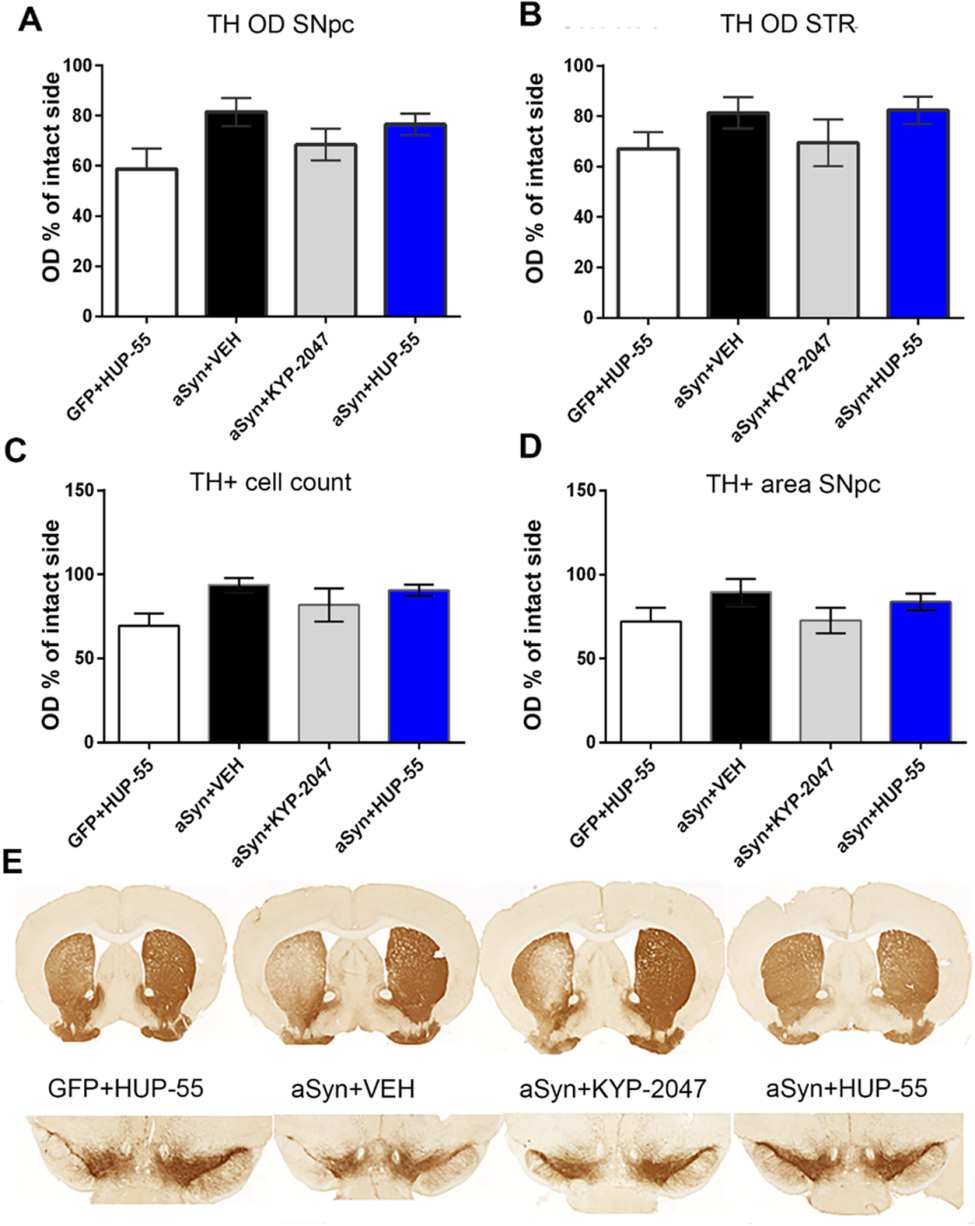
(A, B) OD of TH+ cells compared to the intact side in the striatum (STR) and *substantia nigra pars compacta* (SNpc). No statistical differences were found between the groups, but GFP reduces OD slightly. (C, D) Data from TH+ cell counts measured by the Aiforia platform; no differences were seen. (E) Representative brain slices. *n* = 7–9/group. Data are presented as mean ± SEM.

The effect of AAV-αSyn on the nigrostriatal dopaminergic system was assessed by measuring the OD of tyrosine hydroxylase positive (TH+) cell immunoreactivity in the SNpc and STR. However, no significant differences between groups were observed, similar to previous studies with the same virus vector^47^ (Figure 6A,B). TH+ cell count and area analysis were performed for the SNpc using the Aiforia platform^49^ (Figure 6C,D), but no significant differences were observed. Similar to earlier reports,^50,51^ our negative control, AAV-GFP, was more toxic for TH+ cells in the SNpc and STR than AAV-αSyn. Interestingly, AAV-GFP-induced TH+ cell loss was not visible in the cylinder test or locomotor activity. This further supports the impact of αSyn aggregation on the functionality of the nigrostriatal dopaminergic system. Moreover, TH+ results did not correlate with motor impairment and the amount of aggregated αSyn in the AAV-αSyn groups. αSyn overexpression is known to downregulate the expression and phosphorylation of TH,^52,53^ but this was not seen in the current study. It is possible that αSyn toxicity could have been more evident in a study design with later time points, as seen in previous viral vector studies^54,55^ or with transgenic mice.^56^ However, the aim of this study was to initiate the treatment at the onset of symptoms, based on the earlier study, and the primary outcome measure for the treatment was behavioral performance.

#### 2.2.6 HUP-55 Reduces αSyn Oligomers in αSyn Transgenic Mouse

**Figure 7:**
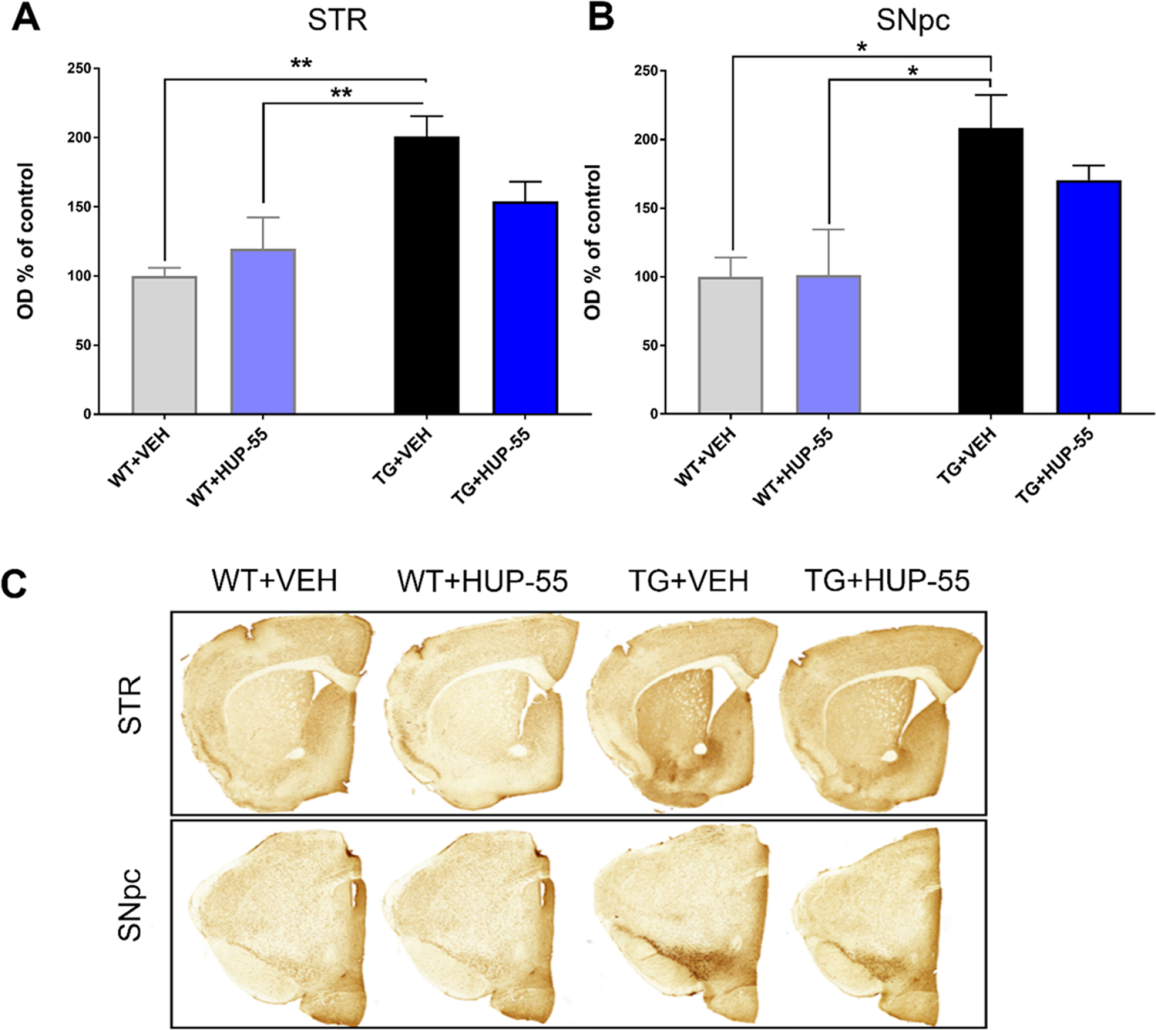
(A, B) Oligomeric αSyn was increased in the striatum (STR) (A) and the *substantia nigra pars compacta* (SNpc) (B) of vehicle-treated A30P*A53T transgenic mice (TG) compared to wild-type littermates (WT). A 7-day treatment with **HUP-55** (10 mg/kg i.p.) decreased the OD of striatal oligomeric αSyn both in STR and SNpc, but this was not statistically significant. (C) Representative immunostained brain slices. *p < 0.05 and **p < 0.01; one-way ANOVA with Tukey’s multiple comparisons (*n* = 5–8). Data are presented as mean SEM.

To test the short-term effects of **HUP-55** on αSyn, similar to KYP-2047,^18^ we performed an i.p. treatment (10 mg/kg every 12 h) for 15-month-old A30P*A53T transgenic mice (TG). The mouse line was previously characterized in the study by Kilpeläinen et al.,^56^ and it showed age-dependent accumulation of oligomeric αSyn in the STR and SNpc. **HUP-55** reduced the oligomeric αSyn in the STR of TG mice compared to vehicle treatment (Figure 7A; 47% decrease), but this was not statistically significant. A similar nonsignificant impact was also seen in the SNpc of TG mice (Figure 7B; 38% decrease). Overall, **HUP-55** showed beneficial effects by reducing αSyn oligomers in two αSyn-based PD mouse models and restoring behavioral impairment in an AAV-αSyn mouse model.

## 3 Conclusions

In the current study, we discovered a series of nonpeptidic oxazole-based PREP inhibitors that lack the carbonyl groups that have been considered to be critical for binding to PREP. Moreover, we showed that the SARs for inhibition of the proteolytic activity of PREP and PPI-mediated functions of PREP are disconnected, and four new oxazole-based PREP ligands in this study were especially potent modulators of the PPI-mediated effects of PREP, although they are only moderate to weak PREP inhibitors. Based on biological characterization and chemical properties, the oxazole **HUP-55** was selected for further studies. It showed an equal impact on αSyn dimerization, autophagy, and ROS production as KYP-2047, which has shown beneficial or even disease-modifying effects in several neurodegenerative *in vitro* and *in vivo* models. This is highly interesting as KYP-2047 is an over 100 times more potent PREP inhibitor than **HUP-55**. Furthermore, the concentration responses of **HUP-55** on αSyn dimerization, autophagy marker LC3BII, and PP2A activation do not correlate with its IC_50_ value. PREP is a highly dynamic protein, and ligand binding has been confirmed to restrict the conformational freedom of PREP. One possible explanation for the disconnected SARs is another ligand binding site inside the cavity of PREP, where binding does not block substrate binding. **HUP-55** passed the blood–brain barrier and had a disease-modifying effect on behavior and αSyn oligomers in an AAV-αSyn-based mouse model. Overall, the new oxazole-based PREP ligands are a promising new finding in drug discovery for PD and other synucleinopathies.

## 4 Experimental Section

### 4.1 Chemistry

#### 4.1.1 General Information

Unless otherwise specified, all reagents and solvents were obtained from commercial suppliers and used without purification. Microwave reactions were performed with fixed hold time in capped microwave vials using a Biotage Initiator+ (Biotage). Completion of reactions and purifications were monitored with thin-layer chromatography (TLC), which was performed on 60 *F*_254_ silica gel plates, using UV light (254 and 366 nm) and ninhydrin or iodine staining to detect products. Flash chromatography was performed manually with silica gel (230–400 μm mesh) or using a Biotage Isolera One (Biotage) with silica gel 60 (40–63 μm mesh). ^1^H and ^13^C NMR spectra were recorded at 400 and 101 MHz, respectively, using an Ascend 400 (Bruker). CDCl_3_ was used as the NMR solvent unless otherwise specified. Chemical shifts (δ) are reported in parts per million (ppm) with TMS or solvent residual peaks as a reference. Many of the compounds contain two or more stable rotamers caused by restricted rotation along the amide bond. NMR signals for minor rotamers making up less than 10% of the total signal are not reported. The purity of the compounds was determined either by LC-MS or combustion elemental analysis. LC-MS was performed using a Waters Acquity UPLC system (Waters) and a Waters Synapt G2 HDMS mass spectrometer (Waters) via an electrospray ionization (ESI) ion source in positive mode. Combustion elemental analysis (C, H, N) was performed at the University of Eastern Finland. The purity of all tested compounds was 95% or higher, except for compounds **17** and **18**, which had purities of 90% and 91%, respectively.

Only the synthesis steps shown in Table 1 and Scheme 2 are presented here. The synthesis of starting materials used for the TFAA dehydration to oxazole and of final compounds that were not tested for biological activity are described in the Supporting Information.

##### 4.1.1.1 Method A: Synthesis of (*S*)-1-(4-Methyl-2-(3-phenylpropyl)oxazol-5-yl)pyrrolidine-2-carbonitrile (**HUP-55**)

TFAA (11 mL, 80 mmol) was added slowly to a solution of compound **1** (13 g, 39 mmol) and Et_3_N (22 mL, 160 mmol) in anhydrous THF (120 mL) at 0 °C. The mixture was left to stir at 0 °C for 2 h before quenching with excess H_2_O and removing THF by evaporation. The residue was diluted with EtOAc, washed with a 10% aqueous solution of citric acid, a saturated solution of NaHCO_3_, and brine, dried over anhydrous Na_2_SO_4_, filtered, and evaporated to provide the crude product, which after flash chromatography (heptane/EtOAc 2:1 → 1:1) yielded **HUP-55** as a yellow sap (7.9 g, 68%). ^1^H NMR δ 7.33–7.23 (m, 2H), 7.23–7.14 (m, 3H), 4.15 (dd, *J* = 7.7, 4.2 Hz, 1H), 3.40 (ddd, *J* = 9.0, 7.9, 4.9 Hz, 1H), 3.23 (dt, *J* = 9.0, 7.3 Hz, 1H), 2.74–2.62 (m, 4H), 2.41–2.00 (m, 6H), 2.11 (s, 3H). ^13^C NMR δ 159.86, 146.20, 141.51, 128.65, 128.51, 126.09, 124.56, 119.82, 52.54, 51.10, 35.34, 31.45, 28.52, 28.13, 24.35, 11.20. HRMS (ESI-QTOF) *m*/*z*: [M + H]^+^ calcd for C_18_H_21_N_3_O: 296,1763; found: 296.1764.

##### 4.1.1.2 (*R*)-1-(4-Methyl-2-(3-phenylpropyl)oxazol-5-yl)pyrrolidine-2-carbonitrile (**3**)

Synthesized according to method A using compound **34** (0.83 g, 2.5 mmol). The crude product was obtained as a yellow sap, which after flash chromatography (heptane/EtOAc 9:1 → EtOAc) yielded compound **3** as a yellow oil (0.17 g, 22%). ^1^H NMR δ 7.33–7.24 (m, 2H), 7.24–7.14 (m, 3H), 4.15 (dd, *J* = 7.7, 4.2 Hz, 1H), 3.39 (ddd, *J* = 9.0, 7.9, 4.9 Hz, 1H), 3.22 (dt, *J* = 9.0, 7.3 Hz, 1H), 2.72–2.63 (m, 4H), 2.37–2.23 (m, 2H), 2.21–2.00 (m, 7H). ^13^C NMR δ 159.88, 146.19, 141.48, 128.62, 128.48, 126.06, 124.50, 119.80, 52.51, 51.07, 35.32, 31.43, 28.50, 28.09, 24.32, 11.13. HRMS (ESI-QTOF) *m*/*z*: [M + H]^+^ calcd for C_18_H_21_N_3_O: 296.1763; found: 296.1765.

##### 4.1.1.3 (*S*)-1-(4-Methyl-2-(3-phenylpropyl)oxazol-5-yl)piperidine-2-carbonitrile (**4**)

Synthesized according to method A using compound **35** (0.39 g, 1.1 mmol). The crude product was obtained as a yellow oil, which after flash chromatography (heptane/EtOAc 3:2) yielded compound **4** (0.25 g, 72%). ^1^H NMR δ 7.33–7.23 (m, 2H), 7.23–7.14 (m, 3H), 4.05 (t, *J* = 4.1 Hz, 1H), 3.33–3.21 (m, 1H), 3.05–2.95 (m, 1H), 2.74–2.62 (m, 4H), 2.12–2.02 (m, 5H), 2.00–1.91 (m, 2H), 1.82–1.59 (m, 4H). ^13^C NMR δ 160.10, 149.25, 141.39, 128.54, 128.38, 125.94, 123.85, 118.06, 52.26, 48.59, 35.21, 29.36, 28.37, 27.99, 25.07, 20.22, 11.05. HRMS (ESI-QTOF) *m*/*z*: [M + H]^+^ calcd for C_19_H_23_N_3_O 310.1919; found: 310.1917.

##### 4.1.1.4 *N*-(Cyanomethyl)-2,2,2-trifluoro-*N*-(4-methyl-2-(3-phenylpropyl)oxazol-5-yl)acetamide (**5**)

Synthesized according to method A using compound **36** (0.093 g, 0.34 mmol). The crude product was obtained as an oil, which after flash chromatography (heptane/EtOAc 9:1 → EtOAc) yielded compound **5** as a colorless oil (0.011 g, 9%). ^1^H NMR δ 7.31–7.26 (m, 2H), 7.23–7.17 (m, 3H), 4.53 (s, 2H), 2.75 (t, *J* = 7.4 Hz, 2H), 2.69 (t, *J* = 7.4 Hz, 2H), 2.17 (s, 3H), 2.13–2.06 (m, 2H). ^13^C NMR δ 164.1, 157.7 (q, ^*2*^*J*_*C,F*_ = 38.4 Hz), 141.0, 136.0, 134.8, 128.6, 126.3, 115.3 (q, ^*1*^*J*_*C,F*_ = 288.9 Hz), 113.3, 37.6, 35.1, 28.2, 27.9, 11.2. HRMS (ESI-QTOF) *m*/*z*: [M + H]^+^ calcd for C_17_H_17_N_3_O_2_F_3_: 352.1273; found: 352.1270.

##### 4.1.1.5 2-(Methyl(4-methyl-2-(3-phenylpropyl)oxazol-5-yl)amino)acetonitrile (**6**)

Synthesized according to method A using compound **37** (0.23 g, 0.80 mmol). The crude product was obtained as a yellow oil, which after flash chromatography (heptane/EtOAc 9:1 → EtOAc) yielded compound **6** as a colorless oil (0.13 g, 62%). ^1^H NMR (400 MHz, CDCl_3_) δ 7.30–7.26 (m, 2H), 7.20–7.16 (m, 3H), 3.80 (s, 2H), 2.85 (s, 3H), 2.71–2.65 (m, 4H), 2.11–2.03 (m, 5H). ^13^C NMR (101 MHz, CDCl_3_) δ 160.3, 149.1, 141.5, 128.6, 128.5, 126.1, 124.2, 115.8, 44.3, 41.1, 35.3, 28.5, 28.1, 11.1. HRMS (ESI-QTOF) *m*/*z*: [M + H]^+^ calcd for C_16_H_20_N_3_O: 270.1606; found: 270.1604.

##### 4.1.1.6 4-Phenylbutanoyl-(4-isopropyl-2-oxazol-5-yl)-2(S)-cyanopyrrolidine (**7**)

Synthesized according to method A using compound **38** (1.28 g, 3.4 mmol). The crude product was obtained, which after flash chromatography (heptane/EtOAc 3:1) yielded compound **7** as a yellow sap (0.13 g, 12%). ^1^H NMR δ 7.51–7.08 (m, 5H), 4.11 (dd, *J* = 7.8, 4.3 Hz, 1H), 3.40–3.33 (m, 1H), 3.28–3.08 (m, 1H), 2.95–2.83 (m, 1H), 2.80–2.45 (m, 4H), 2.45–2.21 (m, 2H), 2.20–1.95 (m, 4H), 1.23 (dd, *J* = 9.3, 7.0 Hz, 6H). ^13^C NMR δ 160.48, 144.42, 141.59, 135.70, 128.65, 128.50, 126.07, 119.87, 52.82, 51.96, 35.46, 31.55, 28.79, 28.48, 25.42, 24.45, 22.13, 22.08. HRMS (ESI-QTOF) *m*/*z*: [M + H]^+^ calcd for C_20_H_25_N_3_O: 324,2076; found: 324.2066.

##### 4.1.1.7 4-Phenylbutanoyl-(4-isobutyl-2-oxazol-5-yl)-2(S)-cyanopyrrolidine (**8**)

Synthesized according to method A using compound **39** (1.13 g, 3.0 mmol). The crude product was obtained, which after flash chromatography (heptane/EtOAc 4:1) yielded compound **8** as a yellow sap (0.68 g, 68%). ^1^H NMR δ 7.44–7.03 (m, 5H), 4.17 (dd, *J* = 7.8, 4.1 Hz, 1H), 3.48–3.33 (m, 1H), 3.22 (dt, *J* = 8.9, 7.3 Hz, 1H), 2.70 (t, *J* = 7.6 Hz, 4H), 2.41–1.92 (m, 10H), 1.03–0.88 (m, 6H). ^13^C NMR δ 160.10, 146.70, 141.57, 128.74, 128.65, 128.50, 126.07, 119.87, 52.72, 51.61, 35.37, 34.54, 31.54, 28.71, 28.28, 28.00, 24.41, 22.62, 22.47. HRMS (ESI-QTOF) *m*/*z*: [M + H]^+^ calcd for C_21_H_27_N_3_O: 338.2232; found: 338.2231.

##### 4.1.1.8 (*S*)-1-(4-(*tert*-Butyl)-2-(3-phenylpropyl)oxazol-5-yl)pyrrolidine-2-carbonitrile (**9**)

Synthesized according to method A using compound **40** (300 mg, 0.80 mmol). The crude product was obtained as a green oil, which after flash chromatography (heptane/EtOAc 4:1 → 2:3) yielded compound **9** as a green oil (152 mg, 56%). ^1^H NMR δ 7.38–7.09 (m, 5H), 4.09 (dd, *J* = 7.9, 4.5 Hz, 1H), 3.33 (ddd, *J* = 8.9, 7.8, 4.8 Hz, 1H), 3.11 (dt, *J* = 8.9, 7.4 Hz, 1H), 2.75–2.61 (m, 4H), 2.40–2.19 (m, 2H), 2.19–1.97 (m, 4H), 1.30 (s, 9H). ^13^C NMR δ 160.24, 143.86, 141.52, 139.55, 128.55, 128.39, 125.96, 119.72, 52.77, 52.72, 35.37, 31.61, 31.46, 29.59, 28.69, 28.37, 24.40. HRMS (ESI-QTOF) *m*/*z*: [M + H]^+^ calcd for C_21_H_28_N_3_O 338.2232; found: 338.2232.

##### 4.1.1.9 4-Phenylbutanoyl-(4-phenyl-2-oxazol-5-yl)-2(*S*)-cyanopyrrolidine (**10**)

Synthesized according to method A using compound **41** (1.72 g, 4.6 mmol). The crude product was obtained, which after flash chromatography (heptane/EtOAc 4:1) yielded compound **10** as a yellow sap (0.68 g, 41%). ^1^H NMR δ 7.87–783 (m, 2H), 7.52–7.17 (m, 8H), 4.36 (dd, *J* = 7.5, 3.8 Hz, 1H), 3.58–3.50 (m, 1H), 3.32–3.23 (m, 1H), 2.79 (q, *J* = 8.2 Hz, 4H), 2.49–2.07 (m, 6H). ^13^C NMR δ 160.07, 145.75, 141.52, 131.57, 128.68, 128.64, 128.53, 127.45, 126.66, 126.34, 126.11, 119.49, 51.58, 50.60, 35.35, 31.60, 28.60, 28.15, 24.17. HRMS (ESI-QTOF) *m*/*z*: [M + H]^+^ calcd for C_23_H_23_N_3_O: 358,1919; found: 358.1918.

##### 4.1.1.10 (*S*)-1-(4-(2-(Methylthio)ethyl)-2-(3-phenylpropyl)oxazol-5-yl)pyrrolidine-2-carbonitrile (**11**)

Synthesized according to method A using compound **42** (0.85 g, 2.1 mmol). The crude product was obtained, which after flash chromatography (heptane/EtOAc 3:2) yielded compound **11** (0.31 g, 47%). ^1^H NMR δ 7.34–7.25 (m, 2H), 7.25–7.17 (m, 3H), 4.24 (dd, *J* = 7.7, 4.1 Hz, 1H), 3.44 (ddd, *J* = 9.0, 7.9, 4.9 Hz, 1H), 3.26 (dt, *J* = 9.0, 7.3 Hz, 1H), 2.85–2.65 (m, 8H), 2.43–2.24 (m, 2H), 2.23–2.15 (m, 1H), 2.14 (s, 3H), 2.13–2.04 (m, 3H). ^13^C NMR δ 160.22, 146.70, 141.44, 128.61, 128.48, 127.02, 126.07, 119.74, 52.72, 51.36, 35.32, 33.35, 31.42, 28.55, 28.19, 25.86, 24.31, 15.71. HRMS (ESI-QTOF) *m*/*z*: [M + H]^+^ calcd for C_20_H_25_N_3_OS: 356.1797; found: 356.1795.

##### 4.1.1.11 (*S*)-1-(2-(3-Phenylpropyl)-4-(2,2,2-trifluoroacetyl)oxazol-5-yl)pyrrolidine-2-carbonitrile (**12**)

Synthesized according to method A using compound **43** (143 mg, 0.45 mmol). The crude product was obtained, which after flash chromatography (heptane/EtOAc 9:1 → 1:4) yielded compound **12** as a white solid (128 mg, 75%). ^1^H NMR δ 7.33–7.23 (m, 2H), 7.23–7.14 (m, 3H), 5.47 (dd, *J* = 7.2, 3.2 Hz, 1H), 3.91 (ddd, *J* = 10.9, 8.0, 4.2 Hz, 1H), 3.72 (dt, *J* = 11.0, 7.6 Hz, 1H), 2.78–2.65 (m, 4H), 2.46–2.14 (m, 4H), 2.14–2.04 (m, 2H). ^13^C NMR δ 172.08 (q, ^*2*^*J*_*C,F*_ = 34.9 Hz), 158.78, 154.00 (q, ^*3*^*J*_*C,F*_ = 1.8 Hz), 141.07, 128.66, 128.55, 126.20, 117.54, 117.22 (q, ^*1*^*J*_*C,F*_ = 290.4 Hz), 111.13, 50.29, 50.18, 35.08, 31.54, 28.02, 27.05, 23.89. HRMS (ESI-QTOF) *m*/*z*: [M + H]^+^ calcd for C_19_H_18_F_3_N_3_O_2_: 378.1429; found: 378.1425.

##### 4.1.1.12 (*S*)-1-(4-Methyl-2-phenyloxazol-5-yl)pyrrolidine-2-carbonitrile (**13**)

Synthesized according to method A using compound **44** (1.03 g, 3.6 mmol). The crude product was obtained as a red oil, which after flash chromatography (heptane/EtOAc 17:3 → 1:4) yielded compound **13** as a yellow oil (0.24 g, 27%). ^1^H NMR δ 7.98–7.90 (m, 2H), 7.46–7.36 (m, 3H), 4.35–4.27 (m, 1H), 3.52 (ddd, *J* = 9.0, 7.9, 4.8 Hz, 1H), 3.37 (dt, *J* = 9.0, 7.3 Hz, 1H), 2.44–2.29 (m, 2H), 2.23 (s, 3H), 2.22–2.06 (m, 2H). ^13^C NMR δ 156.01, 146.69, 129.84, 128.70, 127.69, 125.76, 124.68, 119.61, 52.22, 50.82, 31.40, 24.24, 11.38. HRMS (ESI-QTOF) *m*/*z*: [M + H]^+^ calcd for C_15_H_15_N_3_O: 254.1293; found: 254.1291.

##### 4.1.1.13 (*S*)-1-(2-Benzyl-4-methyloxazol-5-yl)pyrrolidine-2-carbonitrile (**14)**

Synthesized according to method A using compound **45** (0.44 g, 1.4 mmol). The crude product was obtained as an orange oil, which after flash chromatography (heptane/EtOAc 4:1 → 3:7) yielded compound **14** as a yellow oil (120 mg, 31%). ^1^H NMR δ 7.36–7.21 (m, 5H), 4.18–4.13 (m, 1H), 3.98 (s, 2H), 3.39 (ddd, *J* = 9.0, 7.8, 4.8 Hz, 1H), 3.21 (dt, *J* = 8.9, 7.4 Hz, 1H), 2.37–2.13 (m, 3H), 2.12 (s, 3H), 2.11–1.99 (m, 1H). ^13^C NMR δ 157.99, 146.74, 135.76, 128.87, 128.80, 127.07, 124.63, 119.78, 52.45, 51.05, 35.27, 31.44, 24.33, 11.26. HRMS (ESI-QTOF) *m*/*z*: [M + H]^+^ calcd for C_16_H_17_N_3_O: 268.1450; found: 268.1449.

##### 4.1.1.14 (*S*)-1-(4-Methyl-2-phenethyloxazol-5-yl)pyrrolidine-2-carbonitrile (**15**)

Synthesized according to method A using compound **46** (490 mg, 1.54 mmol). The crude product was obtained as a yellow sap, which after flash chromatography (heptane/EtOAc 17:3 → 1:4) yielded compound **15** as a yellow oil (87 mg, 20%). ^1^H NMR δ 7.29–7.04 (m, 5H), 4.05 (dd, *J* = 7.7, 4.4 Hz, 1H), 3.36–3.27 (m, 1H), 3.17–3.08 (m, 1H), 3.01–2.93 (m, 2H), 2.92–2.83 (m, 2H), 2.32–2.15 (m, 2H), 2.04 (s, 3H), 2.14–1.92 (m, 2H). ^13^C NMR δ 159.15, 146.15, 140.49, 128.53, 128.33, 126.31, 124.65, 119.71, 52.41, 51.06, 33.16, 31.33, 30.47, 24.25, 11.09. HRMS (ESI-QTOF) *m*/*z*: [M + H]^+^ calcd for C_17_H_19_N_3_O: 282.1606; found: 282.1607.

##### 4.1.1.15 (*S*)-1-(4-Methyl-2-(4-phenylbutyl)oxazol-5-yl)pyrrolidine-2-carbonitrile (**16**)

Synthesized according to method A using compound **47** (854 mg, 2.47 mmol). The crude product was obtained as a yellow sap, which after flash chromatography (heptane/EtOAc 9:1 → EtOAc) yielded compound **16** as a yellow oil (246 mg, 32%). ^1^H NMR δ 7.33–7.23 (m, 2H), 7.23–7.11 (m, 3H), 4.19–4.11 (m, 1H), 3.39 (ddd, *J* = 9.0, 7.8, 4.9 Hz, 1H), 3.22 (dt, *J* = 8.9, 7.3 Hz, 1H), 2.71–2.59 (m, 4H), 2.40–2.22 (m, 2H), 2.22–1.99 (m, 5H), 1.83–1.63 (m, 4H). ^13^C NMR δ 160.03, 146.14, 142.28, 128.52, 128.42, 125.86, 124.52, 119.82, 52.52, 51.09, 35.58, 31.43, 30.98, 28.53, 26.55, 24.33, 11.17. HRMS (ESI-QTOF) *m*/*z*: [M + H]^+^ calcd for C_19_H_23_N_3_O: 310.1919; found: 310.1921.

##### 4.1.1.16 (*S*)-1-(4-Methyl-2-(2-phenoxyethyl)oxazol-5-yl)pyrrolidine-2-carbonitrile (**17)**

Synthesized according to method A using compound **48** (0.90 g, 2.7 mmol). The crude product was obtained as a yellow foam, which after flash chromatography (heptane/EtOAc 4:1 → EtOAc) yielded compound **17** as a brown sap (41 mg, 5%). ^1^H NMR δ 7.36–7.25 (m, 2H), 7.01–6.91 (m, 3H), 4.35 (t, *J* = 6.9 Hz, 2H), 4.18 (dd, *J* = 7.7, 4.3 Hz, 1H), 3.43 (ddd, *J* = 9.0, 7.8, 4.8 Hz, 1H), 3.26 (dt, *J* = 9.0, 7.3 Hz, 1H), 3.16 (t, *J* = 6.9 Hz, 2H), 2.41–2.23 (m, 2H), 2.14 (s, 3H), 2.13–2.00 (m, 2H). ^13^C NMR δ 158.56, 156.82, 146.71, 129.61, 124.72, 121.17, 119.81, 114.80, 64.69, 52.46, 51.13, 31.47, 29.14, 24.39, 11.12. HRMS (ESI-QTOF) *m*/*z*: [M + H]^+^ calcd for C_17_H_19_N_3_O_2_: 298.1556; found: 298.1554.

##### 4.1.1.17 (*S*)-1-(2-(3-(4-Methoxyphenyl)propyl)-4-methyloxazol-5-yl)pyrrolidine-2-carbonitrile (**18**)

Synthesized according to method A using compound **49** (0.21 g, 0.58 mmol). The crude product was obtained as a yellow oil, which after flash chromatography (heptane/EtOAc 9:1 → EtOAc) yielded compound **18** as a yellow oil (18 mg, 10%). ^1^H NMR δ 7.10–6.98 (m, 2H), 6.83–6.70 (m, 2H), 4.08 (dd, *J* = 7.7, 4.3 Hz, 1H), 3.71 (s, 3H), 3.35–3.29 (m, 1H), 3.15 (dt, *J* = 9.0, 7.3 Hz, 1H), 2.64–2.51 (m, 4H), 2.29–1.90 (m, 6H), 2.02 (s, 3H). ^13^C NMR δ 160.17, 158.01, 146.24, 133.54, 129.53 (2 signals), 124.33, 119.77, 113.99, 113.94, 55.40, 52.51, 51.07, 34.42, 31.44, 28.74, 27.98, 24.34, 10.97. HRMS (ESI-QTOF) *m*/*z*: [M + H]^+^ calcd for C_19_H_23_N_3_O_2_: 326.1869; found: 326.1870.

##### 4.1.1.18 (*S*)-1-(2-(3-(3,4-Dimethoxyphenyl)propyl)-4-methyloxazol-5-yl)pyrrolidine-2-carbonitrile (**19**)

Synthesized according to method A using compound **50** (0.51 g, 1.29 mmol). The crude product was obtained as a yellow sap, which after flash chromatography (heptane/EtOAc 4:1 → EtOAc) yielded compound **19** as a yellow oil (112 mg, 24%). ^1^H NMR δ 6.83–6.66 (m, 3H), 4.19–4.14 (m, 1H), 3.87 (s, 3H), 3.85 (s, 3H), 3.40 (ddd, *J* = 8.9, 7.9, 4.9 Hz, 1H), 3.23 (dt, *J* = 9.0, 7.3 Hz, 1H), 2.72–2.60 (m, 4H), 2.39–2.23 (m, 2H), 2.22–1.98 (m, 7H). ^13^C NMR δ 159.96, 148.96, 147.39, 146.20, 134.13, 124.29, 120.46, 119.76, 111.93, 111.34, 56.05, 55.96, 52.49, 51.00, 34.93, 31.41, 28.68, 28.04, 24.29, 11.09. HRMS (ESI-QTOF) *m*/*z*: [M + H]^+^ calcd for C_20_H_25_N_3_O_3_: 356.1974; found: 356.1974.

##### 4.1.1.19 (*S*)-1-(2-(3,5-Dimethoxyphenethyl)-4-methyloxazol-5-yl)pyrrolidine-2-carbonitrile (**20**)

Synthesized according to method A using compound **51** (0.43 g, 1.13 mmol). The crude product was obtained as a yellow oil, which after flash chromatography (heptane/EtOAc 4:1 → EtOAc) yielded compound **20** as a yellow sap (137 mg, 36%). ^1^H NMR δ 6.40–6.29 (m, 3H), 4.18–4.12 (m, 1H), 3.77 (s, 6H), 3.39 (ddd, *J* = 9.0, 7.8, 4.8 Hz, 1H), 3.22 (dt, *J* = 9.0, 7.3 Hz, 1H), 3.04–2.88 (m, 4H), 2.41–2.22 (m, 2H), 2.22–2.00 (m, 5H). ^13^C NMR δ 160.99, 159.24, 146.32, 142.96, 124.78, 119.83, 106.47, 98.46, 55.40, 52.53, 51.18, 33.56, 31.47, 30.47, 24.39, 11.17. HRMS (ESI-QTOF) *m*/*z*: [M + H]^+^ calcd for C_19_H_25_N_3_O_3_: 342.1818; found: 342.1821.

##### 4.1.1.20 (*S*)-1-(4-Methyl-2-(3-(2-thienyl)propyl)oxazol-5-yl)pyrrolidine-2-carbonitrile (**21**)

Synthesized according to method A using compound **52** (0.48 g, 1.5 mmol). The crude product was obtained, which after flash chromatography (heptane/EtOAc 3:2 → 1:2) yielded compound **21** as a yellow oil (95 mg, 20%). ^1^H NMR δ 7.12 (dd, *J* = 5.1, 1.2 Hz, 1H), 6.91 (dd, *J* = 5.1, 3.4 Hz, 1H), 6.81 (dq, *J* = 3.3, 1.0 Hz, 1H), 4.16 (dd, *J* = 7.8, 4.2 Hz, 1H), 3.40 (ddd, *J* = 9.0, 7.9, 4.9 Hz, 1H), 3.23 (dt, *J* = 9.0, 7.3 Hz, 1H), 2.97–2.88 (m, 2H), 2.70 (t, *J* = 7.6 Hz, 2H), 2.40–2.23 (m, 2H), 2.23–2.02 (m, 7H). ^13^C NMR δ 159.52, 146.26, 144.16, 126.91, 124.71, 124.54, 123.32, 119.80, 52.51, 51.09, 31.43, 29.30, 28.80, 27.87, 24.34, 11.18. HRMS (ESI-QTOF) *m*/*z*: [M + H]^+^ calcd for C_16_H_20_N_3_OS: 302.1327; found: 302.1328.

##### 4.1.1.21 (*S*)-1-(2-(2-(1*H*-Indol-3-yl)ethyl)-4-methyloxazol-5-yl)pyrrolidine-2-carbonitrile (**22**)

Synthesized according to method A using compound **53** (1.29 g, 3.6 mmol), with the exception that the compound was left with the basic washing solution for an extended period of time. The crude product was obtained as a yellow foam, which after flash chromatography (heptane/EtOAc 9:1 → 3:7) yielded compound **22** as a yellow sap (0.62 g, 41%). ^1^H NMR δ 8.13 (s, 1H), 7.62–7.50 (m, 1H), 7.38–7.28 (m, 1H), 7.18 (ddd, *J* = 8.1, 6.9, 1.2 Hz, 1H), 7.10 (ddd, *J* = 8.0, 7.1, 1.1 Hz, 1H), 7.03–6.93 (m, 1H), 4.03 (dd, *J* = 7.5, 4.5 Hz, 1H), 3.36 (ddd, *J* = 8.9, 7.8, 4.8 Hz, 1H), 3.26–3.10 (m, 3H), 3.10–2.97 (m, 2H), 2.34–2.18 (m, 2H), 2.18–1.95 (m, 2H), 2.12 (s, 3H). ^13^C NMR δ 159.84, 146.24, 136.36, 127.37, 124.68, 122.07, 121.71, 119.90, 119.39, 118.78, 114.92, 111.24, 52.46, 51.13, 31.39, 29.65, 24.35, 22.92, 11.16. HRMS (ESI-QTOF) *m*/*z*: [M + H]^+^ calcd for C_19_H_20_N_4_O: 321.1715; found: 321.1716.

##### 4.1.1.22 (*S*)-1-(4-Methyl-2-(2-(1-(2,2,2-trifluoroacetyl)-1*H*-indol-3-yl)ethyl)oxazol-5-yl)pyrrolidine-2-carbonitrile (**23**)

Synthesized according to method A using compound **53** (0.49 g, 1.4 mmol). The crude product was obtained as a pale yellow foam, which after flash chromatography (heptane/EtOAc 9:1 → 1:4) yielded compound **23** as a yellow oil (78 mg, 18%). ^1^H NMR δ 8.47–8.40 (m, 1H), 7.58–7.52 (m, 1H), 7.48–7.34 (m, 2H), 7.32–7.27 (m, 1H), 4.09 (dd, *J* = 7.5, 4.4 Hz, 1H), 3.37 (ddd, *J* = 8.9, 7.9, 4.9 Hz, 1H), 3.22–3.12 (m, 3H), 3.12–3.01 (m, 2H), 2.37–2.21 (m, 2H), 2.20–2.01 (m, 2H), 2.11 (s, 3H). ^13^C NMR δ 158.55, 146.51, 136.35, 130.64, 126.55, 125.69, 124.93, 124.81, 120.79, 119.75, 119.37, 117.22, 52.47, 51.00, 31.44, 28.18, 24.31, 22.37, 11.15 (CF_3_ and carbonyl carbon are not visible due to splitting). HRMS (ESI-QTOF) *m*/*z*: [M + H]^+^ calcd for C_21_H_19_F_3_N_4_O_2_: 417.1538; found: 417.1539.

##### 4.1.1.23 (*S*)-1-(2-(3-(Azepan-1-yl)-3-oxopropyl)-4-methyloxazol-5-yl)pyrrolidine-2-carbonitrile (**24**)

Synthesized according to method A using compound **54** (1.6 g, 4.4 mmol). The crude product was obtained as an orange oil, which after flash chromatography (EtOAc → EtOAc/MeOH 19:1) yielded compound **24** as an orange oil (0.79 g, 55%). ^1^H NMR δ 4.20–4.15 (m, 1H), 3.57–3.51 (m, 2H), 3.50–3.44 (m, 2H), 3.40 (ddd, *J* = 8.9, 7.7, 4.8 Hz, 1H), 3.24 (dt, *J* = 9.0, 7.3 Hz, 1H), 3.06–2.98 (m, 2H), 2.84–2.75 (m, 2H), 2.38–2.02 (m, 4H), 2.10 (s, 3H), 1.79–1.65 (m, 4H), 1.61–1.52 (m, 4H). ^13^C NMR δ 170.57, 159.26, 146.17, 124.68, 119.78, 52.40, 51.13, 47.76, 46.05, 31.34, 29.77, 29.03, 27.59, 27.09, 26.88, 24.30, 24.13, 11.09. HRMS (ESI-QTOF) *m*/*z*: [M + H]^+^ calcd for C_18_H_26_N_4_O_2_: 331.2134; found: 331.2135.

##### 4.1.1.24 Formylalanine (**56**)

Ac_2_O (53 mL, 557 mmol) was added dropwise to a solution of dl-alanine (7.1 g, 80 mmol) in HCO_2_H (100 mL, 89%) at 0 °C. The mixture was left to stir at room temperature for 4 days. Water was added and the contents were evaporated to obtain the crude product (quantitative). The product was used in the next step without further purification. ^1^H NMR (Methanol-*d*_4_) δ 8.06 (s, 1H), 4.47 (d, *J* = 7.3 Hz, 1H), 1.40 (d, *J* = 7.3 Hz, 3H). ^13^C NMR (Methanol-*d*_4_) δ 175.42, 163.29, 48.01, 17.98.

##### 4.1.1.25 Method B: Synthesis of *N*-(1-oxo-1-(pyrrolidin-1-yl)propan-2-yl)formamide (**57**)

Pivaloyl chloride (9.9 mL, 80 mmol) was added dropwise to a solution of compound **56** (9.3 g, 80 mmol) and Et_3_N (12 mL, 88 mmol) in anhydrous DCM (200 mL) at 0 °C. The mixture was left to stir at 0 °C for 1 h before adding pyrrolidine (6.6 g, 80 mmol) and Et_3_N (12 mL, 88 mmol). The resulting mixture was left to stir at room temperature for 1 day. The organic phase was washed with a 30% aqueous solution of citric acid, brine, and a saturated solution of NaHCO_3_, dried over anhydrous Na_2_SO_4_, filtered, and evaporated to provide the crude product, which after flash chromatography (EtOAc/MeOH 9:1 → 1:1) yielded compound **57** as a white solid (8.4 g, 62%). ^1^H NMR δ 8.18 (s, 1H), 6.80 (s, 1H), 4.91–4.71 (m, 1H), 3.66–3.40 (m, 4H), 2.06–1.97 (m, 2H), 1.96–1.87 (m, 2H), 1.39 (d, *J* = 6.8 Hz, 3H). ^13^C NMR δ 170.42, 160.28, 46.51, 46.28, 45.75, 26.18, 24.24, 18.55.

##### 4.1.1.26 2-Isocyano-1-(pyrrolidin-1-yl)propan-1-one (**58**)

POCl_3_ (14 mL, 148 mmol) was added dropwise to a solution of compound **57** (8.4 g, 49 mmol) and Et_3_N (35 mL, 247 mmol) in anhydrous DCM (500 mL) at −25 °C. The mixture was left to stir at −25 °C for 2 h, quenched with excess of a saturated solution of NaHCO_3_, allowed to warm to room temperature, then left to stir at room temperature overnight. The aqueous phase was extracted with DCM, and the combined organic phases were washed with brine, dried over anhydrous Na_2_SO_4_, filtered, and evaporated to provide a crude product, which after flash purification (hexane/EtOAc 1:1 → EtOAc) yielded compound **58** as a pale yellow solid (4.4 g, 58%). ^1^H NMR δ 4.39 (q, *J* = 6.9 Hz, 1H), 3.68 (dt, *J* = 10.0, 6.9 Hz, 1H), 3.62–3.42 (m, 3H), 2.15–1.85 (m, 4H), 1.62 (d, *J* = 6.9 Hz, 3H). ^13^C NMR δ 195.10, 163.78, 51.20, 46.93, 46.81, 26.37, 24.12, 18.57.

##### 4.1.1.27 1-(4-Methyl-5-(pyrrolidin-1-yl)oxazol-2-yl)-3-phenylpropan-1-one (**25**)

A solution of 3-phenylpropionyl chloride (0.12 mL, 0.80 mmol) in anhydrous DCM (0.75 mL) was added dropwise to a solution of compound **58** (121 mg, 0.80 mmol) and Et_3_N (0.11 mL, 0.80 mmol) in anhydrous DCM (3.5 mL). The mixture was left to stir at room temperature for 2 h. The organic phase was washed with a solution of Na_2_CO_3_, dried over anhydrous Na_2_SO_4_, filtered, and evaporated to provide a crude product, which after flash purification (heptane/EtOAc 7:3) yielded compound **25** as a white solid (37 mg, 16%). ^1^H NMR δ 7.24–7.07 (m, 5H), 3.56–3.44 (m, 4H), 3.18–3.07 (m, 2H), 3.01–2.93 (m, 2H), 2.22 (s, 3H), 1.99–1.83 (m, 4H). ^13^C NMR δ 183.37, 154.27, 148.11, 141.40, 128.65, 128.46, 126.06, 111.96, 48.61, 39.12, 30.74, 25.57, 12.58. HRMS (ESI-QTOF) *m*/*z*: [M + H]^+^ calcd for C_17_H_21_N_2_O_2_: 285.1603; found: 285.1603.

##### 4.1.1.28 5-Amino-2-(3-phenylpropyl)oxazole-4-carbonitrile (**26**)

SOCl_2_ (0.53 mL, 7.3 mmol) was added to 4-phenylbutyric acid (1.0 g, 6.1 mmol) at 70 °C. The flask was covered with a CaCl_2_ drying tube, and the mixture was stirred at 70 °C for 1 h. Excess SOCl_2_ was evaporated to give the crude intermediate as an orange oil (quantitative). Aminomalononitrile *p*-toluenesulfonate (1.54 g, 6.1 mmol) was added to the intermediate in anhydrous DMF (30 mL). The mixture was heated to 120 °C and left to stir for 30 min before diluting with EtOAc. The organic phase was washed with H_2_O, dried over anhydrous Na_2_SO_4_, filtered, and evaporated to provide the crude product as an orange oil, which after flash chromatography (heptane/EtOAc 3:1 → 1:1) yielded compound **26** as a white solid (0.86 g, 62%). ^1^H NMR δ 7.34–7.24 (m, 2H), 7.24–7.13 (m, 3H), 4.92 (s, 2H), 2.69 (t, *J* = 7.5 Hz, 2H), 2.62 (t, *J* = 7.5 Hz, 2H), 2.08–1.99 (m, 2H). ^13^C NMR δ 160.42, 154.99, 141.04, 128.59, 126.25, 114.35, 87.00, 34.97, 28.06, 27.10. HRMS (ESI-QTOF) *m*/*z*: [M + H]^+^ calcd for C_13_H_13_N_3_O: 228.1137; found: 228.1135.

##### 4.1.1.29 5-Bromo-2-(3-phenylpropyl)oxazole-4-carbonitrile (**59**)

Compound **26** (0.84 g, 3.7 mmol) was added slowly to a suspension of CuBr_2_ (1.64 g, 7.4 mmol) and *tert*-butyl nitrite (90%, 0.48 mL, 4.1 mmol) in anhydrous MeCN (22 mL). The mixture was left to stir at room temperature for 19 h, before diluting with Et_2_O. The organic phase was washed with 1 M HCl, dried over anhydrous Na_2_SO_4_, filtered, and evaporated to provide the crude product as a yellow oil, which after flash chromatography (heptane/EtOAc 19:1 → 3:2) yielded compound **59** as a colorless oil (0.49 g, 46%). ^1^H NMR δ 7.34–7.25 (m, 2H), 7.25–7.13 (m, 3H), 2.80 (t, *J* = 7.5 Hz, 2H), 2.72 (t, *J* = 7.4 Hz, 2H), 2.19–2.06 (m, 2H). ^13^C NMR δ 167.45, 140.54, 130.61, 128.68, 128.59, 126.43, 115.86, 111.22, 34.91, 27.85, 27.65.

##### 4.1.1.30 Method C: 2-(3-Phenylpropyl)-5-(pyrrolidin-1-yl)oxazole-4-carbonitrile (**27**)

Pyrrolidine (0.05 mL, 0.58 mmol) and potassium *tert*-butoxide (65 mg, 0.58 mmol) were added to a solution of compound **59** (130 mg, 0.45 mmol), Pd(OAc)_2_ (1 mg, 5 μmol), and BINAP (8 mg, 10 μmol) in anhydrous 1,4-dioxane (3.8 mL) in a microwave vial. The mixture was heated to 170 °C in the microwave for 15 min before diluting with EtOAc. The organic phase was washed with H_2_O, dried over anhydrous Na_2_SO_4_, filtered, and evaporated to provide the crude product as a brown oil, which after flash chromatography (heptane/EtOAc 9:1 → 1:4) yielded compound **27** as a yellow oil (85 mg, 67%). ^1^H NMR δ 7.34–7.24 (m, 2H), 7.24–7.11 (m, 3H), 3.62–3.48 (m, 4H), 2.69 (t, *J* = 7.5 Hz, 2H), 2.61 (t, *J* = 7.5 Hz, 2H), 2.09–1.95 (m, 6H). ^13^C NMR δ 159.29, 153.31, 141.21, 128.60, 128.52, 126.13, 116.85, 83.13, 48.17, 35.04, 28.10, 27.19, 25.55. HRMS (ESI-QTOF) *m*/*z*: [M + H]^+^ calcd for C_17_H_19_N_3_O: 282.1606; found: 282.1605.

##### 4.1.1.31 (*S*)-1-(4-Cyano-2-(3-phenylpropyl)oxazol-5-yl)pyrrolidine-2-carboxamide (**60**)

Synthesized according to method C using l-prolinamide (163 mg 1.43 mmol). The crude product was obtained, which after flash chromatography (EtOAc → EtOAc/MeOH 19:1) yielded compound **60** as an orange oil (86 mg, 22%). ^1^H NMR δ 7.44–7.08 (m, 5H), 6.39–6.17 (m, 1H), 6.00–5.76 (m, 1H), 4.32 (ddd, *J* = 7.9, 3.8, 1.4 Hz, 1H), 3.93–3.79 (m, 1H), 3.64–3.51 (m, 1H), 2.73–2.63 (m, 2H), 2.63–2.55 (m, 2H), 2.34–2.14 (m, 2H), 2.15–1.88 (m, 4H). ^13^C NMR δ 174.07, 159.10, 154.56, 141.05, 128.59, 128.54, 126.16, 115.91, 85.17, 62.68, 49.69, 34.97, 31.32, 27.94, 27.10, 24.25.

##### 4.1.1.32 (*S*)-5-(2-Cyanopyrrolidin-1-yl)-2-(3-phenylpropyl)oxazole-4-carbonitrile (**28**)

Synthesized according to method A using compound **60** (86 mg, 0.27 mmol). The crude product was obtained as a brown oil, which after flash chromatography (heptane/EtOAc 9:1 → 1:4) yielded compound **28** as an orange oil (29 mg, 36%). ^1^H NMR δ 7.34–7.23 (m, 2H), 7.23–7.13 (m, 3H), 4.69–4.58 (m, 1H), 3.88–3.77 (m, 1H), 3.63 (dtd, *J* = 10.0, 7.5, 1.2 Hz, 1H), 2.76–2.62 (m, 4H), 2.47–2.18 (m, 4H), 2.12–2.02 (m, 2H). ^13^C NMR δ 157.26, 155.37, 140.99, 128.64, 128.58, 126.22, 117.72, 114.93, 86.62, 49.33, 48.31, 34.99, 31.54, 27.95, 27.14, 24.47. HRMS (ESI-QTOF) *m*/*z*: [M + H]^+^ calcd for C_18_H_18_N_4_O: 307.1559; found: 307.1563.

##### 4.1.1.33 Method D: Synthesis of 2-(3-Phenylpropyl)-5-(2-thienyl)oxazole (**30**)

A solution of 2-(2-thienyl)acetaldehyde (supporting info compound **76**) (622 mg, 4.9 mmol) in toluene (6 mL) and 4-phenylbutylamine (1.2 mL, 7.4 mmol) were added to a solution of CuBr_2_ (1.65 g, 7.4 mmol), K_2_CO_3_ (1.36 g, 9.9 mmol), and pyridine (79 μL, 0.98 mmol) in toluene (80 mL). The mixture was left to stir at 80 °C for 18 h before filtering through Celite using EtOAc and evaporating. The residue was dissolved in EtOAc, washed with a 30% aqueous solution of citric acid, a saturated solution of NaHCO_3_, and brine, dried over anhydrous Na_2_SO_4_, filtered, and evaporated to provide the crude product, which after flash chromatography (toluene/EtOAc 19:1 → 9:1) yielded compound **30** (100 mg, 8%). ^1^H NMR δ 7.24–7.08 (m, 7H), 7.01 (s, 1H), 6.98 (dd, *J* = 5.1, 3.6 Hz, 1H), 2.74 (t, *J* = 7.5 Hz, 2H), 2.66 (t, *J* = 7.6 Hz, 2H), 2.07 (p, *J* = 7.6 Hz, 2H). ^13^C NMR δ 163.48, 146.54, 141.39, 130.26, 128.65, 128.55, 127.84, 126.14, 125.32, 123.96, 121.66, 35.22, 28.65, 27.64. HRMS (ESI-QTOF) *m*/*z*: [M + H]^+^ calcd for C_16_H_15_NOS: 270.0952; found: 270.0952.

##### 4.1.1.34 5-Phenyl-2-(3-phenylpropyl)oxazole (**29**)

Synthesized according to method D using 2-phenylacetaldehyde (561 mg, 4.1 mmol). The crude product was obtained, which after flash chromatography (toluene/EtOAc 19:1) yielded compound **29** as a pale yellow oil (273 mg, 50%). ^1^H NMR δ 7.58–7.49 (m, 2H), 7.36–7.29 (m, 2H), 7.26–7.19 (m, 3H), 7.17–7.09 (m, 4H), 2.78 (t, *J* = 7.6 Hz, 2H), 2.66 (t, *J* = 7.6 Hz, 2H), 2.09 (p, *J* = 7.5 Hz, 2H). ^13^C NMR δ 164.35, 151.09, 141.46, 128.99, 128.66, 128.56, 128.37, 128.26, 126.15, 124.11, 121.91, 35.27, 28.72, 27.79. HRMS (ESI-QTOF) *m*/*z*: [M + H]^+^ calcd for C_18_H_18_NO 264.1388; found: 264.1388.

##### 4.1.1.35 (4-Phenylbutanoyl)-l-threonine Methyl Ester (**61**)

Synthesized according to method B using 4-phenylbutyric acid (2.4 g, 15 mmol) and methyl 2-amino-3-hydroxybutanoate hydrochloride (supporting info compound **78**) (2.5 g, 15 mmol). The crude product was obtained, which after flash chromatography (heptane/EtOAc 3:1) yielded compound **61** (3.0 g, 75%). ^1^H NMR δ 7.32–7.24 (m, 2H), 7.25–7.14 (m, 3H), 6.25 (d, *J* = 8.8 Hz, 1H), 4.61 (dd, *J* = 8.9, 2.5 Hz, 1H), 4.34 (qd, *J* = 6.3, 1.8 Hz, 1H), 3.76 (s, 3H), 2.67 (t, *J* = 7.6 Hz, 2H), 2.34 (s, 1H), 2.32–2.25 (m, 2H), 2.06–1.93 (m, 2H), 1.21 (d, *J* = 6.4 Hz, 3H). ^13^C NMR δ 173.51, 171.75, 141.53, 128.64, 128.55, 126.13, 68.10, 57.18, 52.71, 35.81, 35.25, 27.24, 20.13.

##### 4.1.1.36 Methyl (4*S*,5*S*)-5-Methyl-2-(3-phenylpropyl)-4,5-dihydrooxazole-4-carboxylate (**62**)

The Burgess reagent (0.95 g, 3.99 mmol) was added to a solution of **61** (1.01 g, 3.62 mmol) in anhydrous THF (78 mL). The mixture was refluxed for 7 h and then left to stir at room temperature overnight. The mixture was concentrated and washed with brine to obtain the crude product as a yellow oil and white solids, which after flash chromatography (heptane/EtOAc 1:1) yielded compound **62** as a colorless oil (440 mg, 47%). ^1^H NMR δ 7.32–7.23 (m, 2H), 7.23–7.13 (m, 3H), 4.84 (qd, *J* = 10.2, 6.4 Hz, 1H), 4.75 (td, *J* = 10.2, 1.2 Hz, 1H), 3.75 (s, 3H), 2.70 (t, *J* = 7.6 Hz, 2H), 2.39–2.30 (m, 2H), 2.07–1.92 (m, 2H), 1.27 (d, *J* = 6.4 Hz, 3H). ^13^C NMR δ 170.61, 170.50, 141.55, 128.65, 128.46, 126.04, 77.33, 71.38, 52.12, 35.20, 27.73, 27.59, 16.28.

##### 4.1.1.37 Methyl 5-Methyl-2-(3-phenylpropyl)oxazole-4-carboxylate (**63**)

Pyridine (5.5 mL, 68 mmol) and tetrachloromethane (3.7 mL, 38 mmol) were added to a solution of compound **62** (440 mg, 1.7 mmol) in anhydrous MeCN (5.5 mL). DBU (1.0 mL, 7 mmol) was added dropwise, and the mixture was left to stir at room temperature overnight. The mixture was concentrated to obtain the crude product as a brown oil, which after flash chromatography (EtOAc) yielded compound **63** as a yellow oil (300 mg, 68%). ^1^H NMR δ 7.32–7.23 (m, 2H), 7.23–7.13 (m, 3H), 3.89 (s, 3H), 2.80–2.72 (m, 2H), 2.69 (t, *J* = 7.6 Hz, 2H), 2.58 (s, 3H), 2.16–2.05 (m, 2H). ^13^C NMR δ 163.00, 162.75, 156.21, 141.18, 128.58, 128.52, 127.21, 126.16, 51.95, 35.26, 28.57, 27.53, 11.98.

##### 4.1.1.38 5-Methyl-2-(3-phenylpropyl)oxazole-4-carboxylic Acid (**64**)

LiOH·H_2_O (72 mg, 1.7 mmol) was added to a solution of compound **63** (300 mg, 1.15 mmol) in MeOH (4 mL) and H_2_O (1 mL). The mixture was left to stir at room temperature for 16 h before removing MeOH by evaporation. The aqueous phase was washed with DCM, acidified with 1 M HCl, and extracted with DCM. The organic phase was dried over anhydrous Na_2_SO_4_, filtered, and evaporated to provide the crude product (242 mg, 86%), which was used without further purification. ^1^H NMR δ 10.20 (s, 1H), 7.34–7.23 (m, 2H), 7.23–7.13 (m, 3H), 2.80 (t, *J* = 7.6 Hz, 2H), 2.70 (t, *J* = 7.5 Hz, 2H), 2.60 (s, 3H), 2.16–2.04 (m, 2H). ^13^C NMR δ 166.00, 162.97, 157.12, 141.04, 128.48, 128.43, 126.72, 126.06, 35.09, 28.32, 27.27, 12.03.

##### 4.1.1.39 (5-Methyl-2-(3-phenylpropyl)oxazol-4-yl)(pyrrolidin-1-yl)methanone (**31**)

Synthesized according to method B using compound **64** (225 mg, 0.92 mmol) and pyrrolidine (0.17 mL, 2.0 mmol). The crude product was obtained, which after two flash chromatographies (heptane/EtOAc 1:2 and DCM/MeOH 19.9:0.1) yielded compound **31** (106 mg, 39%). ^1^H NMR δ 7.34–7.24 (m, 2H), 7.24–7.15 (m, 3H), 3.88 (t, *J* = 6.6 Hz, 2H), 3.60 (t, *J* = 6.7 Hz, 2H), 2.78–2.66 (m, 4H), 2.55 (s, 3H), 2.07 (p, *J* = 7.6 Hz, 2H), 1.99–1.81 (m, 4H). ^13^C NMR δ 162.19, 160.78, 153.67, 141.47, 130.65, 128.62, 128.54, 126.14, 48.73, 46.64, 35.18, 28.61, 27.41, 26.74, 23.98, 12.04. Anal. calcd for C_18_H_22_N_2_O_2_·0.2 H_2_O: C 71.38, H 7.49, N 9.25; found: C 71.637, H 7.450, N 9.280.

##### 4.1.1.40 (*S*)-1-(5-Methyl-2-(3-phenylpropyl)oxazole-4-carbonyl)pyrrolidine-2-carboxamide (**32**)

Synthesized according to method B using compound **64** (330 mg, 1.35 mmol) and l-prolinamide (180 mg, 1.6 mmol). The crude product was obtained, which after flash chromatography (DCM/MeOH 19:1) yielded compound **32** (240 mg, 52%). ^1^H NMR δ 7.36–7.24 (m, 2H), 7.24–7.15 (m, 3H), 6.89 (s, 0.7H), 6.31 (s, 0.3H), 5.46 (s, 1H), 5.20 (d, *J* = 7.6 Hz, 0.3H), 4.83–4.66 (m, 0.7H), 4.17–3.89 (m, 1.3H), 3.88–3.63 (m, 0.7H), 2.78–2.63 (m, 4H), 2.63–2.52 (m, 3H), 2.44–2.29 (m, 1H), 2.18–1.87 (m, 5H) (two rotamers 13:7). ^13^C NMR δ 175.67, 173.86, 163.56, 162.87, 161.03 (2 signals), 155.46, 154.98, 141.35 (2 signals), 129.94, 129.46, 128.62 (2 signals), 128.57 (2 signals), 126.19 (2 signals), 62.26, 60.45, 49.69, 47.30, 35.16, 31.58, 28.53, 28.05, 27.36, 27.30, 27.23, 25.59, 22.02, 21.19, 14.34, 12.25. (second set of signals (ca. 30%) from a minor rotamer). Anal. calcd for C_19_H_23_N_3_O_3_·0.3 H_2_O: C 65.80, H 6.86, N 12.12; found: C 65.960, H 6.693, N 12.043.

##### 4.1.1.41 (*S*)-1-(5-Methyl-2-(3-phenylpropyl)oxazole-4-carbonyl)pyrrolidine-2-carbonitrile (**33**)

Synthesized according to method A using compound **32** (159 g, 0.58 mmol). The crude product was obtained, which after flash chromatography (heptane/EtOAc 2:1 → 1:1) yielded compound **33** (118 mg, 63%). ^1^H NMR δ 7.33–7.24 (m, 2H), 7.24–7.13 (m, 3H), 5.85 (d, *J* = 7.5 Hz, 0.5H), 4.91–4.78 (m, 0.5H), 4.18–4.07 (m, 0.5H), 4.07–3.94 (m, 0.5H), 3.88–3.75 (m, 0.5H), 3.67–3.55 (m, 0.5H), 2.79–2.66 (m, 4H), 2.59 (s, 3H), 2.45–2.34 (m, 0.5H), 2.33–2.02 (m, 5.5H) (two rotamers 1:1). ^13^C NMR δ 162.32, 161.61, 161.26, 161.08, 156.02, 155.96, 141.35 (2 signals), 129.27, 129.07, 128.66, 128.62, 128.57 (2 signals), 126.21, 126.17, 119.77, 118.83, 49.23, 48.84, 47.40, 46.67, 35.15 (2 signals), 32.78, 29.68, 28.48, 28.26, 27.33 (2 signals), 25.84, 22.30, 12.27, 12.25 (two equal sets of signals from rotamers). Anal. calcd for C_19_H_21_N_3_O_2_·0.2 H_2_O: C 69.60, H 6.61, N 12.82; found: C 69.787, H 6.333, N 12.970.

### 4.2 Biological Assays

#### 4.2.1 Chemicals and Reagents

For biological assays and animal experiments, reagents were purchased from Sigma-Aldrich (St-Louis, MO) unless otherwise stated in the text. Ethanol was purchased from Altia (Helsinki, Finland).

### 4.3 *In Vitro* Studies

#### 4.3.1 PREP Activity Assay

IC_50_ values of the compounds against PREP were determined with purified recombinant porcine PREP. PREP enzyme was purified according to the protocol described in the study by Venäläinen et al.^40^ In the microplate assay procedure, 10 μL of the enzyme dilution was preincubated with 65 μL of 0.1 M sodium–potassium phosphate buffer (pH 7.0) containing the compounds at different concentrations at 30 °C for 30 min. The reaction was initiated by adding 25 μL of 4 mM Suc-Gly-Pro-AMC dissolved in 0.1 M sodium–potassium phosphate buffer (pH 7.0), and the mixture was incubated at 30 °C for 60 min. The reaction was terminated by adding 100 μL of 1 M sodium acetate buffer (pH 4.2). The formation of 7-amido-4-methylcoumarin was determined fluorometrically with a microplate fluorescence reader (excitation at 360 nm and emission at 460 nm). The final concentration of the compounds in the assay mixture varied from 1 mM to 1 nM, and the final concentration of the enzyme was approximately 2 nM. The inhibitory activities (percent of control) were plotted against the log concentration of the compound, and the IC_50_-value was determined by nonlinear regression utilizing GraphPad Prism 3.0 software.

#### 4.3.2 Cell Cultures

Mouse neuronal N2A, HEK-293, and human neuroblastoma (SH-SY5Y) cell lines were used in this study. The cells were obtained from ATCC (Manassas, VA). N2A cells were cultured in Dulbecco’s modified Eagle medium (DMEM-Glutamax; #31966021; ThermoFisher Scientific) with additional 10% (v/v) fetal bovine serum (FBS; #16000-044, ThermoFisher Scientific) and 1% l-glutamine–penicillin–streptomycin solution (15140122; ThermoFisher Scientific). HEK-293 cells were cultured in DMEM (#D6429, Sigma) with an additional 10% FBS and 1% l-glutamine–penicillin–streptomycin solution (15140122; ThermoFisher Scientific). HEK-293 PREP-KO cells^57^ were cultured similarly to HEK-293 cells but with 20% FBS. SH-SY5Y cells were cultured in DMEM-Glutamax with 15% (v/v) FBS, 1% non-essential amino acids (NEAA; #11140050; ThermoFisher Scientific), and 50 μg/mL Gentamycin (15750-045; ThermoFisher Scientific). During the culturing, the cells were kept in a humified incubator at +37 °C with 5% CO_2_ and used in passages 3–15.

#### 4.3.3 Mouse Primary Cortical Neuron Cultures

Pure primary mouse cortical neurons were obtained from C57BL/6JRccHsd mice (WT) (P1). Cortical tissue was dissected out and dissociated in ice-cold Hank’s Balanced Salt Solution (HBSS) medium (#14175, ThermoFisher Scientific) containing trypsin (0.25 mg/mL) and DNAse (5 U/mL). After centrifugation, cortical neurons were resuspended in Neurobasal Medium (NBM; #21103049, ThermoFisher Scientific) containing 2% (v/v) B27 (#17504044, ThermoFisher Scientific), 1% (v/v) penicillin–streptomycin solution, 100 μg/mL Primocin (#ant-pm-1, InvivoGen, Toulouse, France), and 2 mM glutamine (#25030081, ThermoFisher Scientific) and seeded onto poly-l-lysine (#p4707, Sigma)-coated cell culture plates at an appropriate density (40,000 cells/well to 96-well plates). After 24 h seeding, the medium was half-changed, and neurons were maintained at 37 °C in a 5% CO_2_ incubator. The neurons were cultured for 7 days for experimental use.

#### 4.3.4 α-Syn-Dimerization Assay

To study the effect of compounds on early phases of αSyn aggregation, αSyn dimerization was assessed by a PCA that has been described in the study by Savolainen et al.^5^ and used by us in several studies.^17,24,25^ Briefly, N2A cells were seeded on 96-well plates (Isoplate white wall, PerkinElmer Life Sciences) at a density of 13,000 cells/well and transfected with 25 ng of both αSyn-Gluc1 and αSyn-Gluc2 or 50 ng mock plasmid as a control by using Lipofectamine 3000 (L3000001; ThermoFisher Scientific) as the transfection reagent. Forty-eight hours post-transfection, cells were incubated for 4 h with study compounds (10 μM) in DMEM without phenol red (11039021; ThermoFisher Scientific). DMSO (0.1%) served as a vehicle control, and proteasomal inhibitor lactacystin (10 μM; L-1147; AG Scientific, San Diego, CA) and MG-132 (10 μM) served as assay controls for αSyn dimerization. The PCA signal was assessed by injecting 25 μL of native coelenterazine (Nanolight Technology) in DMEM without phenol red per well. The emitted luminescence was read using a Varioskan LUX multimode microplate reader (ThermoFisher Scientific). A similar protocol was performed for HEK-293 PREP-KO cells to verify PREP-specific effects. For each experimental condition, 4 replicate wells were used in each experiment and 2–6 separate experiments for each treatment.

#### 4.3.5 Autophagic Flux

To assess the effect of compounds on autophagy, autophagic flux was determined by using HEK-293 cells with stable GFP-LC3B-RFP construct expression.^58^ The assay was performed as described in Svarcbahs et al.^7^ Briefly, the cells were seeded at a density of 30,000 cells/well on black 96-well plates (Costar, Corning) and treated for 24 h with study compounds of 24 h post-plating with 10 μM concentration. Rapamycin (500 nM), an mTOR inhibitor (BML-A275; Enzo Life Sciences), was used as a positive control for autophagy induction and 20 nM bafilomycin 1A (ML1661) as an autophagy inhibitor. After 24 h treatment (compound concentration 10 μM), the cells were washed once with warm phosphate-buffered saline (PBS), and the GFP signal was read with a Victor2 multilabel counter (PerkinElmer; excitation/emission 485 nm/535 nm). Four replicate wells were used for each experimental condition in each experiment, and 2–14 independent experiments were performed for each condition.

#### 4.3.6 ROS Detection Assay

The impact of compounds on ROS production under OS was assessed as we have done earlier in the study by Eteläinen et al.^10^ In short, SH-SY5Y cells were plated on clear-bottom black-walled 96-well plates (30,000 cells per well) and incubated overnight. OS was induced by treating the cells with culturing medium, including 100 μM H_2_O_2_ (H1009; Merck) and 10 mM FeCl_2_ (44939-50G) with or without concurrent treatment compounds for 3 h (compound concentration 10 μM). The cells in the control wells received only fresh cell growth medium during OS induction. Stress-induced ROS production was studied using the DCFDA cellular ROS detection assay kit (ab113851, Abcam) according to the protocol provided with it. The ROS proportional fluorescence signal was measured with the Victor2 multilabel counter (PerkinElmer; excitation/emission 485 nm/535 nm). Four replicate wells were used for each experimental condition in each experiment, and at least 4 independent experiments were performed for each condition.

#### 4.3.7 Protein Purification for Close-Relative Enzyme Specificity Assay

The recombinant proteins were prepared and purified as described in the study by Van der Veken et al.^30^

*PREP:* Human recombinant PREP was expressed in BL21(DE3) cells and purified using immobilized Co-chelating chromatography (GE Healthcare), followed by anion-exchange chromatography on a 1 mL Mono Q column (GE Healthcare).

*FAP:* A gateway-entry clone for human FAP was purchased from Dharmacon (Accession number DQ891423), and the human secretion signal was replaced with the HoneyBee melittin secretion signal. For transfection and expression of FAP in Sf9 insect cells, the C-terminal BaculoDirect kit from LifeTechnologies was used. The enzyme was purified from the supernatant of the insect cells using immobilized Ni-chelating chromatography (GE Healthcare, Diegem, Belgium), followed by anion-exchange chromatography using a 1 mL HiTrap Q and size exclusion chromatography using the Superdex 200 column (GE Healthcare, Diegem, Belgium).

*DPP4:* DPPIV was purified from human seminal plasma, as described previously.

*DPP2:* Recombinant human DPP2 was purchased from R&D Systems (#3438-SE-010).

*DPP9:* Gateway-entry clones for human DPP9 were purchased from Dharmacon (Accession number DQ892325). For transfection and expression of DPP9 in Sf9 insect cells, the N-terminal BaculoDirect kit from LifeTechnologies was used. The enzyme was purified using immobilized Ni-chelating chromatography (GE healthcare, Diegem, Belgium), followed by anion-exchange chromatography using 1 mL of Mono Q (GE Healthcare, Diegem, Belgium).

#### 4.3.8 Enzyme Activity Measurements for Close-Relative Enzyme Specificity Assay

*PREP:* Initial screening of **HUP-55** was done using *N*-succinyl-Gly-Pro-7-amino-4-methylcoumarine (AMC) (Bachem) as the substrate at a concentration of 250 μM at pH 7.4 (0.1 M K-phosphate, 1 mM EDTA, 1 mM DTT). **HUP-55** was tested at two concentrations, 1 and 10 μM, being the final concentration in the well. **HUP-55** was preincubated with the enzyme for 15 min at 37 °C; afterward, the substrate was added and the velocities of AMC release were measured kinetically at λ_ex_ = 380 nm and λ_em_ = 465 nm for at least 10 min at 37 °C. Measurements were done on the Infinite 200 (Tecan Group Ltd.), and the Magellan software was used to process the data.

*FAP:* Initial screening of the **HUP-55** was done using Z-Gly-Pro-AMC (Bachem) as the substrate at a concentration of 50 μM at pH 8 (0.05 M Tris-HCl buffer with 0.1% glycerol, 1 mg/mL BSA, and 140 mM NaCl). **HUP-55** was preincubated with the enzyme for 15 min at 37 °C; afterward, the substrate was added and the velocities of AMC release were measured kinetically at λ_ex_ = 380 nm and λ_em_ = 465 nm for at least 10 min at 37 °C. Measurements were done on the Infinite 200 as above.

*DPP4 and DPP9:* Ala-Pro-para-nitroanilide (pNA) was used as the substrate at the respective concentrations of 25 μM (DPP4) or 150 μM (DPP9) at pH 7.4 (0.05 M HEPES–NaOH buffer with 0.1% Tween-20, 0.1 mg/mL BSA, and 150 mM NaCl). **HUP-55** was preincubated with the enzyme for 15 min at 37 °C; afterward, the substrate was added and the velocities of pNA release were measured kinetically at 405 nm for at least 10 min at 37 °C. Measurements were done on the Infinite 200 as above.

DPP2: Lys-Ala-pNA was used as the substrate at a concentration of 1 mM at pH 5.5 (100 mM NaAc, 10 mM EDTA, 14 μg/mL aprotinin). Similar to above, **HUP-55** was tested at two concentrations (1 and 10 μM) and preincubated for 15 min at 37 °C. The substrate was added, and the velocities of pNA release were measured kinetically at 405 nm for at least 10 min at 37 °C. Measurements were done on the Infinite 200 as above.

#### 4.3.9 Autophagy and PP2A Marker Assays

To assess the effect of the lead compound, **HUP-55**, on autophagy marker LC3BII and PP2A levels, HEK-293 or HEK-293 PREP-KO cells were plated on a 6-well plate (500,000 cells/well) and allowed to attach overnight. Thereafter, the cells were incubated with 0.1% DMSO (vehicle), 10 μM KYP-2047, or **HUP-55** for 4 or 24 h based on our earlier study.^7^ After the incubation, the cells were lysed in RIPA buffer as described earlier,^7^ and the supernatant was collected. The levels of the autophagosome marker (LC3B) and catalytic subunit of PP2A (PP2Ac) were detected by using WB, as described below.

#### 4.3.10 Western Blot

WB analysis was used to study protein markers from **HUP-55**/KYP-2047 incubated cell lysates, as well as from mouse brain tissue homogenates. The standard sodium dodecyl sulfate-polyacrylamide gel electrophoresis (SDS-PAGE) protocol was used, and 30 μg of protein per sample was loaded on 4–20% (#4568094, Bio-Rad, CA) stain-free precast gels. The gels were transferred to PVDF membranes (Trans-blot Turbo Midi 0.2 μm, #1704157, Bio-Rad) using the Trans-blot Turbo Transfer System (Bio-Rad). The membranes were blocked with 5% skim milk in TTBS, which was followed by the addition of the primary antibody diluted in 5% skim milk in TTBS and overnight incubation on a swinger at +4 °C. Following primary antibodies were used: Rb LC3B (1:1000, L7543, Sigma-Aldrich), Rb PP2A phospho-T307 (1:500, PA5-36874, ThermoFisher Scientific), Rb PP2AC (α + β); Clone Y119 (1:2000, ab32141, AbCam), Rb PREP (1:1000, ab58988, Abcam), Rb β-actin (1:2000, loading control, ab8227, Abcam), and Rb Vinculin (1:10,000, ab129002, AbCam).

The following day, the membranes were washed, followed by a 2 h incubation at room temperature with Gt-anti-Rb (#31460, Invitrogen, 1:2000). After incubation, the membranes were washed and incubated with SuperSignal West Pico (#34577) or Femto (#34095) Chemiluminescent Substrate (ThermoFisher Scientific) for 5 min, and the images were captured with the ChemiDoc XRS+ Gel Imaging System (Bio-Rad) controlled by ImageLab software (version 6.01, Bio-Rad).

To verify that bands were in the linear range of detection, increasing exposure time and automatic detection of saturated pixels in ImageLab software (version 6.01, Bio-Rad) was used. Thereafter, images were converted to 8-bit greyscale format, and the OD (arbitrary units, a.u.) of the bands were measured with ImageJ (histogram area analysis; version 1.53c; NIH). The OD obtained from each band was normalized against the corresponding β-actin or vinculin band, which was used as a loading control.

#### 4.3.11 Cellular Thermal Shift Assay

CETSA was performed as previously described.^41^ HEK-293 cells were seeded to T25 flasks with a density of 1 × 10^6^ cells. After 24 h, the cells were exposed to 10 μM **HUP-55** or corresponding vehicle (0.01% DMOS) in the medium for 1 h. After the exposure, the cells were collected in PBS and aliquoted into 7 PCR tubes (100,000 cells/tube). The cells were prewarmed at 37 °C for 3 min, then heated to 37, 47, 50, 53, 56, 63, or 67 °C for 3 min and subsequently cooled at 25 °C for 3 min using a PCR Mastercycler (T100 Thermal Cycler, Bio-Rad). After heating, the cells were disrupted with two freeze–thaw cycles by submerging the tubes into liquid nitrogen and subsequently thawed by incubation at 25 °C for 3 min. The aggregated proteins were removed by centrifugation (at 20,000*g* for 20 min at 4 °C), and the soluble fractions were diluted with Laemmli buffer (Bio-Rad, Hercules, CA) and analyzed with Western blot as described above. The nondenaturated protein fractions (%) were calculated by comparing the intensities of temperature-treated cell samples to the corresponding cell samples from 37 °C.

### 4.4 *In Vivo* Studies

#### 4.4.1 Animals

For the AAV-αSyn experiment, 10 to 11 weeks old male C57BL/6JRccHsd mice (*n* = 40) obtained from Envigo (The Netherlands) were used. The mice were singly housed in individually ventilated cages (Mouse IVC Green Line, Techniplast, Italy), kept under standard laboratory conditions (room temperature 23 ± 2 °C, 12 h light/dark cycle), and had access to food (Teklad 2016, Envigo) and irradiated tap water *ad libitum*. For the brain penetration study, the animals were 10 weeks old (*n* = 40). Animals were given 10 mg/kg **HUP-55** or KYP-2047 or vehicle (5% Tween-20 in 0.9% NaCl (Braun)), followed by the collection of the brains 15, 30, 45, 60, 120, and 180 min after the injection. For the 7-day i.p. treatment with **HUP-55**, 15-month-old male and female C57BL/6J-Tg(Th-SNCA*A30P*A53T)39Eric/J mice characterized in the study by Kilpeläinen et al.^56^ were used. For these mice, **HUP-55** (10 mg/kg) or vehicle (5% Tween-20 in 0.9% NaCl (Braun)) was administered i.p. every 12 h for 7 days.

Animal experiments were conducted according to the ARRIVE guidelines and 3R principles of the EU directive 2010/63/EU regarding the care and use of experimental animals and following the local laws and regulations (Finnish Act on the Protection of Animals Used for Scientific or Educational Purposes (497/2013), Government Degree on the Protection of Animals Used for Scientific or Educational Purposes (564/2013)). The experiment protocols were authorized by the National Animal Experiment Board of Finland (ESAVI/441/04.10.07/2016).

#### 4.4.2 LC-MS Detection of HUP-55 in the Mouse Brain

I.p. injection of **HUP-55** (10 mg/kg) was given to C57BL/6JRccHsd mice (*n* = 3/time point), and the mice were deeply anesthetized with sodium pentobarbital anesthesia (i.p., 200 mg/kg), perfused briefly with PBS, and the brains were removed at 0, 30, 60, 120, and 180 min after the injection.

The mouse brain tissue and cells were disrupted with a ball mill, followed by a freeze–thaw cycle integrated with ultrasonication. The samples were extracted with 500 μL of ACN twice, evaporated to dryness, and reconstituted in 200 μL of ACN. The chromatographic separation was performed in the Waters Acquity UPLC BEH C18 column (1.7 μm 2.1 mm × 50 mm) at 40 °C and with a flow rate of 0.6 mL/min. The mobile phases consisted of 0.1% formic acid in MQ H_2_O (A) and 0.1% formic acid in ACN (B). The linear gradient started from 5% B and increased to 95% B in 9 min. HUP-55 was analyzed with Exion UPLC - 6500+ QTRAP/MS instrument (Sciex) following the transitions in the Multiple Reaction Monitoring (MRM) method: MRM 312 → 285 for **HUP-55**. The concentration of **HUP-55** was quantified using a calibration curve with the corresponding standard, and the data was normalized to the fresh weight (FW) of the samples.

#### 4.4.3 Stereotactic AAV Virus Vector Microinjections

The mice were injected with AAV2-CBA-αSyn (*n* = 30) or AAV2-CBA-GFP (*n* = 10) (obtained from Michal J Fox Foundation for Parkinson’s disease) under isoflurane anesthesia (4% induction, 2% maintenance). The injections were given above the left SNpc, A/P: −3.1, L/M: −1.2, and D/V: −4.2 from bregma according to Franklin and Paxinos,^59^ as we have previously done in several studies.^47,57,60^ An injection volume of 1 μL was administered at the rate of 0.2 μL/min, and a rest time of 5 min was used before removing the needle from the brain to prevent AAV leakage up the needle tract. Topical lidocaine (10 mg/mL), buprenorphine (0.1 mg/kg), and carprofen (5 mg/kg) subcutaneous (s.c.) injections were provided as pre- and postoperative pain management.

#### 4.4.4 Treatments and Experiment Setup

Mice injected with AAV-αSyn received 10 mg/kg/day KYP-2047 (*n* = 10) or **HUP-55** (*n* = 10) and vehicle (*n* = 10) treatment in minipumps 4 weeks after the virus vector surgeries. The dose was based on our earlier studies with AAV2-CBAαSyn virus vector experiment with mice,^47,57^ αSyn transgenic mice,^22^ and on brain pharmacokinetic study with KYP-2047.^46^ The treatment lasted for 4 weeks. AAV-GFP-injected mice received **HUP-55** treatment 10 mg/kg/day. Osmotic minipump (Alzet 1004, Durect; flow rate of 0.11 μL/h) implanted in the abdominal cavity was used to provide chronic administration. Priming doses dissolved in 5% Tween 80 in saline (i.p., 10 mg/kg) were given on the first day of the treatment to ensure the immediate onset of the drug effect.

#### 4.4.5 Minipump Surgeries

Osmotic minipump (Alzet 1004, Durect; flow rate of 0.11 μL/h) implantation was performed 4 weeks after virus vector injections in a stereotaxic operation as described in the study by Svarcbahs et al.^47^ Minipumps were filled with 16 mM KYP-2047 solution (0.2% dimethyl sulfoxide (DMSO) in PBS) or 16 mM **HUP-55** in 5% Tween in saline (Braun) and primed according to producer’s instructions. 5% Tween in saline was used as a vehicle. A cannula (Alzet Brain Infusion Kit 3, Durect) was implanted in the left hemisphere at 0.7 mm anterior and 1.4 mm lateral to bregma as described in the study by Hof et al.,^61^ and was lowered 2.5 mm deep to lateral ventricle (stereotaxic coordinates according to Franklin and Paxinos^59^) and the attached osmotic minipump was implanted subcutaneously in the intracapsular region. Topical lidocaine (10 mg/mL), buprenorphine, (0.1 mg/kg) and carprofen (5 mg/kg) s.c. injections were provided as pre- and postoperative pain management. Osmotic minipumps were kept in mice for 28 days.

#### 4.4.6 Cylinder Test

Asymmetry in spontaneous forepaw use was studied with the cylinder test, similar to the study by Svarcbahs et al.^47^ The mice were video recorded in transparent plastic cylinders (height 15 cm; diameter 12 cm) for 5 min or until they had touched the cylinder wall at least 20 times. Each forepaw contact with the cylinder wall was counted (“left”; “both”, “right”). A baseline cylinder test was done before the viral vector injections and then repeated every 2 weeks. The data is presented as a percentage of the ipsilateral forepaw use compared to the overall forepaw use: [(ipsilateral paw + 0.5 × both)/(ipsilateral paw + contralateral paw + both)] × 100%.

#### 4.4.7 Tissue Processing

At the end of the experiment, mice were transcardially perfused with PBS followed by 4% paraformaldehyde (PFA) under terminal sodium pentobarbital anesthesia (i.p., 200 mg/kg), and the brains were collected. The brains were postfixed in 4% PFA at 4 °C for 24 h and subsequently transferred into 10% sucrose in PBS and kept there overnight at 4 °C. On the following day, brains were transferred further into 30% sucrose in PBS and kept at 4 °C for another 24 h. After this, the brains were frozen on dry ice and kept at −80 °C until sectioning. The brains were cut into 30 μm free-floating sections on a cryostat (Leica CM3050) and kept in cryopreservation solution (30% ethylene glycol and 30% glycerol in 0.5 M phosphate buffer) until staining.

#### 4.4.8 Immunohistochemistry

IHC staining of 30 μm striatal and nigral sections was performed for tyrosine hydroxylase (TH) and oligomer-specific αSyn. For the TH staining, the sections were quenched with 10% methanol and 3% hydrogen peroxide in PBS for 10 min to inactivate the endogenous peroxidase activity. The nonspecific binding was blocked with 10% normal goat serum (S-1000–20, Vector Laboratories) in 0.5% Triton-X in PBS for 30 min. After blocking, sections were incubated overnight at room temperature with rabbit anti-TH primary antibody (1:2000 in 1% normal goat serum in 0.5% Triton-X in PBS, AB152, Sigma-Aldrich). Sections were then incubated with biotinylated goat anti-rabbit secondary antibody (1:500 in 1% normal goat serum in 0.5% Triton-X in PBS, BA1000, Vector Laboratories) at room temperature for 2 h. The signal was enhanced with the avidin–biotin complex method (Vectastain ABC standard kit, PK-6100, Vector laboratories) according to instructions provided by the manufacturer, and the immunoreactivity was visualized with 0.05% DAB solution (3,3′-diaminobenzidine and 0.03% H_2_O_2_ in PBS). The sections were then moved on gelatin-coated glass slides, air-dried overnight at room temperature, dehydrated in an alcohol series, and coverslipped using Pertex mounting medium (HistoLab).

Oligomer-specific αSyn IHC was done using the Basic Vector Mouse on Mouse (M.O.M.) Immunodetection kit (BMK-2202, Vector Laboratories) according to Brännström et al.^62^ with a few modifications. The sections were quenched as above, and nonspecific binding was blocked by incubating the sections with M.O.M. Ig blocking reagent for 30 min. Further, the sections were incubated with M.O.M. diluent for 5 min and then transferred to mouse anti-human αSynO5 primary antibody (1:200 in M.O.M. diluent, AS132718, Agrisera) for overnight incubation. αSynO5 primary antibody is oligomer-specific^46^ and does not react with mouse endogenous αSyn in tissue IHC.^47^ The sections were then incubated with biotinylated anti-mouse lgG secondary antibody (1:300 in M.O.M. diluent, MKB-2225, Vector Laboratories) for 2 h. The signal was again enhanced with avidin–biotin complex method (Vectastain ABC standard kit, PK-6100, Vector laboratories), and the immunoreactivity visualized with DAB as described above.

#### 4.4.9 Microscopy and Stereological Count of Dopaminergic Neurons

The OD of TH and αSynO5 from STR and SNpc were determined. Digital images were single-layer scanned at 20× magnification with a Pannoramic Flash II Scanner (version 1.15.4., 3DHISTECH). Three sections of STR and four sections from SNpc from each mouse were processed for further analyses with Pannoramic Viewer (version 1.15.4., 3DHISTECH), and images were converted to greyscale and inverted with ImageJ (version 1.53c, NIH). The line analysis tool (for αSynO5 in STR) and freehand tool (for αSynO5 in SNpc and TH in both STR and SNpc) in ImageJ were used to measure the OD. To correct the effect of background staining, correction values were obtained from the corpus callosum (for STR) and cerebral peduncle (for SNpc).

The number of TH+ cells in SNpc was estimated using a stereological counting algorithm based on convolutional neural networks in the Aiforia Cloud (version RELEASE_4.9_HOTFIX_4, Aiforia Technologies). The counting algorithm for TH+ neurons in SNpc has been developed and characterized earlier in the study of Penttinen et al.^49^ The digital images were obtained with extended focus at 20× magnification with the Pannoramic Flash II Scanner (3DHISTECH). Four coronal sections were selected for analysis from each mouse, and the data was presented as mentioned above.

#### 4.4.10 Statistical Analysis

All data are expressed as mean values ± standard error of the mean (mean ± SEM). In cases where negative control was used, its average was set as 100% on each assay to reduce variability between repeats. To analyze the statistical differences between groups, one- or two-way analysis of variance (ANOVA) was followed by a suitable post-hoc comparison if the ANOVA assay gave statistical significance (*p* < 0.05). In all cases, *p* < 0.05 were considered to be significant. Statistical analysis, curve fitting (CETSA, PP2Ac/pPP2A ratio), and area under curve calculations were performed using PRISM GraphPad statistical software (version 9.0, GraphPad Software, Inc.).

## Supporting Information Available

Docking model of **HUP-55** with PREP (PDB)
Docking model of compound **7** with PREP (PDB)
Docking model of compound **8** with PREP (PDB)
Docking model of compound **14** with PREP (PDB)
Docking model of compound **15** with PREP (PDB)
Molecular formula strings and the associated biological data (CSV)
Synthesis of intermediates **1** and **34**–**54**; synthesis of untested final compounds **65**–**70**; UPLC traces and NMR spectra for the tested compounds; monitoring of long-term stability, results from the NMR, MS, and configurational stability studies of **HUP-55**; figures illustrating the postulated binding modes for **HUP-55** and some close analogues; selected QikProp results; a figure illustrating the impact of the tested compounds on αSyn dimerization, autophagy, and OS; toxicity testing results; LC-MS measurements of **HUP-55** metabolites in mouse brains; locomotor activity measurements; and total αSyn immunoreactivity in the STR results (PDF)

## Supplementary Material

jm3c00235_si_001.pdb

jm3c00235_si_002.pdb

jm3c00235_si_003.pdb

jm3c00235_si_004.pdb

jm3c00235_si_005.pdb

## Abbreviations Used

αSyn: α-synuclein
AAV: adeno-associated virus
AMC: 7-amino-4-methyl coumarin
BINAP: 2,2′-bis(diphenylphosphino)-1,1′-binaphthyl
DBU: 1,8-diazabicyclo[5.4.0]undec-7-ene
DMEM: Dulbecco’s modified Eagle medium
DPP: dipeptidyl peptidase
FAP: fibroblast activating protein
FBS: fetal bovine serum
HBSS: Hank’s Balanced Salt Solution
KO: knock-out
LC3B: microtubule-associated proteins 1A/1B light chain 3B
mTOR: mammalian target of rapamycin
N2A: Neuro2A
NBM: Neurobasal Medium
PCA: protein-fragment complementation assay
PFA: paraformaldehyde
PP2A: protein phosphatase 2A
PPI: protein–protein interaction
PREP: prolyl oligopeptidase
STR: striatum
SNpc: *substantia nigra pars compacta*
TG: transgenic
TH: tyrosine hydroxylase
WB: western blot
