# Supplementary material for: Nonpeptidic Oxazole-Based Prolyl Oligopeptidase Ligands with Disease-Modifying Effects on α-Synuclein Mouse Models of Parkinson’s Disease
Source: J Med Chem. 2023 May 29;66(11):7475–96. doi: 10.1021/acs.jmedchem.3c00235 (PMC10258805; doi:10.1021/acs.jmedchem.3c00235)
Supplement: Supplementary file 7 — jm3c00235_si_007.pdf [file jm3c00235_si_007.pdf]

Supporting Information

Non-peptidic oxazole-based prolyl oligopeptidase ligands with disease-modifying effects on α-synuclein mouse models of Parkinson's disease

Tommi P. Kilpeläinen[1,†], Henri T. Pätsi[2,†], Reinis Svarcbahs[1], Ulrika H. Julku[1], Tony S. Eteläinen[1], Hengjing Cui[1,3], Samuli Auno[1], Nina Sipari[4], Susanna Norrbacka[1], Teppo O. Leino[2], Maria Jäntti[1], Timo T. Myöhänen[1,3], Erik A. A. Wallén[2*]

[1]Division of Pharmacology and Pharmacotherapy, and [2]Division of Pharmaceutical Chemistry and Technology, Drug Research Program, Faculty of Pharmacy, University of Helsinki, P.O. Box 56, 00014 Helsinki, Finland
[3]School of Pharmacy, Faculty of Health Sciences, University of Eastern Finland, Yliopistonranta 1C, 70211 Kuopio, Finland
[4]Viikki Metabolomics Unit, Department of Biosciences, University of Helsinki, Viikinkaari 5 E, 00014 Helsinki, Finland

[†]These authors contributed equally.

*Corresponding author: Dr. Erik A. A. Wallén
E-mail: erik.wallen@helsinki.fi

Contents

Chemistry S2
- General information S2
- Synthesis of intermediates S2
- Synthesis of untested final compounds S17
- UPLC-MS traces and NMR spectra S22
- Monitoring stability S62
- HUP-55 NMR stability S63
- HUP-55 MS stability S64
- HUP-55 configurational stability S65
- Molecular modelling S65
- QikProp results for selected compounds S68

Biological data S69
- Results of the screening assay S69

Toxicity testing of selected compounds S70
LC-MS detection of HUP-55 metabolites in the mouse brain S70
Results of the locomotor activity recordings S71
Results of total aSyn immunoreactivity in striatum S72
References S73

Chemistry

General information

Unless otherwise specified, all reagents and solvents were obtained from commercial suppliers and used without purification. Microwave reactions were performed with fixed hold time in capped microwave vials using a Biotage Initiator+ (Biotage). Completion of reactions and purifications were monitored with TLC, which was performed on 60 F_{254} silica gel plates, using UV light (254 and 366 nm) and ninhydrin or iodine staining to detect products. Flash chromatography was performed manually with silica gel (230-400 µm mesh) or using a Biotage Isolera One (Biotage) with silica gel 60 (40-63 µm mesh). ^{1}H and ^{13}C NMR spectra were recorded at 400 MHz and 101 MHz, respectively, using an Ascend 400 (Bruker). $CDCl_3$ was used as the NMR solvent unless otherwise specified. Chemical shifts (δ) are reported in parts per million (ppm) with TMS or solvent residual peaks as reference. Many of the compounds contain two or more stable rotamers caused by restricted rotation along the amide bond. NMR signals for minor rotamers making up less than 10 % of the total signal are not reported.

Synthesis of intermediates

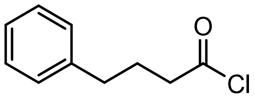

Method A: Synthesis of 4-phenylbutanoyl chloride (71). $SOCl_2$ (11 ml, 151 mmol) was added to 4-phenylbutyric acid (20 g, 122 mmol) at 70 °C. The flask was covered with a $CaCl_2$ drying tube and the mixture was stirred at 70 °C for 2h. Excess $SOCl_2$ was evaporated to give the crude product as an orange oil (quantitative), which was used without further purification.

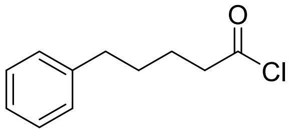

5-Phenylpentanoyl chloride (72). Synthesized according to method A using 5-phenylvaleric acid (1.0 g, 5.6 mmol). The crude product was obtained (quantitative), which was used without further purification.

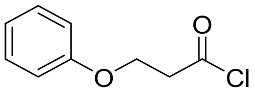

3-Phenoxypropanoyl chloride (73). Synthesized according to method A using 3-phenoxypropionic acid (1.0 g, 6.0 mmol). The crude product was obtained as an orange oil (quantitative), which was used without further purification.

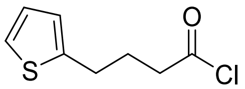

4-(2-Thienyl)butanoyl chloride (74). Synthesized according to method A using 4-(2-thienyl)butyric acid (0.55 ml, 3.7 mmol). The crude product was obtained (quantitative), which was used without further purification.

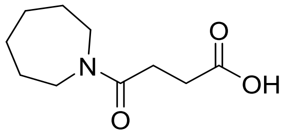

4-(Azepan-1-yl)-4-oxobutanoic acid (75). Hexamethyleneimine (11 ml, 100 mmol) was added to a solution of succinic anhydride (5.0 g, 50 mmol) in anhydrous DCM (150 ml) at 0 °C. The mixture was left to stir at room temperature for 1 d. The organic phase was washed with 0.5 M HCl and extracted with a saturated solution of $NaHCO_3$. The basic aqueous phase was acidified and extracted with DCM. The resulting organic phase was dried over anhydrous Na_2SO_4, filtered, and evaporated to provide the crude product as a colourless oil (4.1 g, 41 %), which was used without further purification. ^{1}H NMR δ 10.98 (s, 1H), 3.54 (t, *J* = 6.1 Hz, 2H), 3.46 (t, *J* = 6.1 Hz, 2H), 2.77 – 2.63 (m, 4H), 1.81 – 1.66 (m, 4H), 1.64 – 1.49 (m, 4H). ^{13}C NMR δ 176.54, 171.88, 48.08, 46.48, 29.95, 28.80, 28.22, 27.48, 27.05, 26.89.

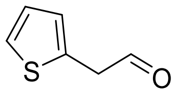

2-(2-Thienyl)acetaldehyde (76). IBX (45 %, 7.4 g, 12 mmol) was added to a solution of 2-thiopheneethanol (1.4 ml, 13 mmol) in anhydrous MeCN (63 ml). The mixture was left to stir at 80 °C for 2 h before it cooling to room temperature and filtering through Celite®. The filtrate was evaporated and the residue purified by flash chromatography (heptane/EtOAc 4:1). Because some benzoic acid remained, the product was dissolved in EtOAc, washed with a saturated solution of $NaHCO_3$ and brine, dried over anhydrous Na_2SO_4, filtered, and evaporated, which yielded compound **76** as a yellow oil (888 mg, 56 %). ^{1}H NMR δ 9.66 (t, *J* = 2.1 Hz, 1H), 7.20 (dd, *J* = 5.1, 1.2 Hz, 1H), 6.96 (dd, *J* = 5.2, 3.5 Hz, 1H), 6.87 (dq, *J* = 3.5, 1.0 Hz, 1H), 3.81 (dd, *J* = 2.2, 0.9 Hz, 2H). ^{13}C NMR δ 197.69, 132.92, 127.67, 127.41, 125.73, 44.18.

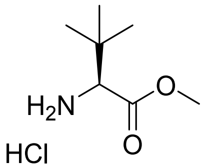

Method B: Synthesis of DL-*t*-leucine methyl ester hydrochloride (77). $SOCl_2$ (1.24 ml, 17 mmol) was added dropwise to a solution of DL-*t*-leucine (1.11 g, 8.5 mmol) in anhydrous MeOH (20 ml) at 0 °C. The mixture was refluxed for 38 h before removing $SOCl_2$ and the solvent through evaporation. The crude product was obtained as an off white foam (1.49 g, 97%), which was used without further purification. Unreacted starting material (c.a. 12 %) was also identified. ^{1}H NMR (Acetonitrile-d_3) δ 8.51 (s, 2H), 3.85 (s, 1H), 3.81 (s, 3H), 1.14 (s, 9H). ^{13}C NMR (Acetonitrile-d_3) δ 169.79, 62.80, 53.42, 34.25, 26.97.

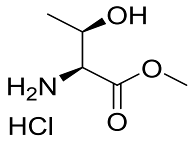

L-Threonine methyl ester hydrochloride (78). Synthesized according to method B using L-threonine (1.78 g, 15 mmol) with a reaction time of 2 h. The crude product was obtained (2.48 g, 97 %), which was used without further purification. ^{1}H NMR δ 8.33 (s, 2H), 5.26 (s, 1H), 4.44 – 4.30 (m, 1H), 4.24 – 4.10 (m, 1H), 3.86 (s, 3H), 1.48 (d, J = 6.2 Hz, 3H). ^{13}C NMR δ 168.95, 66.18, 59.60, 53.76, 20.65.

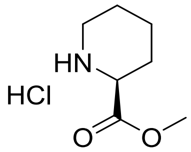

Methyl (*S*)-piperidine-2-carboxylate (79). Synthesized according to method B using L-pipecolic acid (1.78 g, 15 mmol) with a reaction time of 2 h. The crude product was obtained (quantitative), which was used without further purification. ^{1}H NMR (Methanol-d_4) δ 4.04 (dd, J = 11.4, 3.5 Hz, 1H), 3.85 (s, 3H), 3.48 – 3.37 (m, 1H), 3.05 (td, J = 12.4, 3.4 Hz, 1H), 2.33 – 2.21 (m, 1H), 1.96 – 1.82 (m, 2H), 1.81 – 1.59 (m, 3H). ^{13}C NMR (Methanol-d_4) δ 170.35, 57.88, 53.74, 45.19, 27.15, 22.87, 22.74.

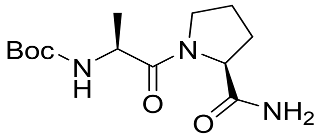

***t*-Butyl ((*S*)-1-((*S*)-2-carbamoylpyrrolidin-1-yl)-1-oxopropan-2-yl)carbamate (80).** A solution of Boc-*L*-alanine *N*-succinimidyl ester (2.5 g, 8.8 mmol) in anhydrous THF (30 ml) was added slowly to a solution of L-prolinamide (1.0 g, 8.8 mmol) in anhydrous THF (30 ml) at 0 °C. The reaction was left to stir at room temperature for 1 d before removing the solvent by evaporation. The residue was dissolved in EtOAc, washed with brine, dried over anhydrous Na_2SO_4, filtered, and evaporated to provide a crude product as a white foam, which after flash chromatography (EtOAc/MeOH 19:1 → EtOAc/MeOH 7:3) yielded compound **80** as a white foam (quantitative). ^{1}H NMR δ 6.82 (s, 1H), 5.69 (s, 1H), 5.38 (d, J = 8.2 Hz, 1H), 4.61 (dd, J = 8.1, 2.8 Hz, 1H), 4.48 (t, J = 7.0 Hz, 1H), 3.78 – 3.47 (m, 2H), 2.44 – 2.28 (m, 1H), 2.19 – 1.83 (m, 5H), 1.47 (s, 10H), 1.34 (d, J = 6.9 Hz, 3H). ^{13}C NMR δ 173.39, 173.18, 155.22, 59.46, 47.80, 47.22, 28.36, 27.14, 25.09, 18.42.

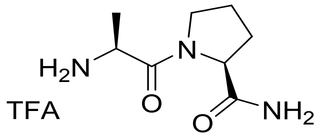

(*S*)-1-(L-Alanyl)pyrrolidine-2-carboxamide trifluoroacetate (81). TFA (4.4 ml, 58 mmol) was added slowly to a solution of compound **80** (1.3 g, 4.4 mmol) in anhydrous DCM (11 ml) at 0 °C. The mixture was left to stir at 0 °C for 2.5 h before removing the solvent and remaining TFA by evaporation. The crude product was obtained as a colourless sap (quantitative), which was used without further purification. ^{1}H NMR (Methanol-d_4) δ 4.49 – 4.42 (m, 1H), 4.24 (q, J = 7.0 Hz, 1H), 3.71 – 3.48 (m, 2H), 2.34 – 1.85 (m, 5H), 1.52 (d, J = 7.0 Hz, 3H). ^{13}C NMR (Methanol-d_4) δ 176.52, 169.64, 61.34, 49.43, 48.37, 30.81, 26.04, 16.06.

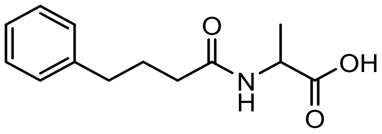

Method C: Synthesis of (4-phenylbutanoyl)alanine (82). Compound **71** (3.2 g, 18 mmol) was added to a solution of D/L-alanine (1.7 g, 19 mmol) in Na_2CO_3 (41 ml, 10 % (m/V), 39 mmol) and Et_2O (40 ml) at 0 °C. The mixture was left to stir vigorously at room temperature overnight. The aqueous phase was washed with Et_2O, acidified with 1 M HCl, and extracted with EtOAc. The combined organic phase was washed with 0.1 M HCl, dried over anhydrous Na_2SO_4, filtered, and evaporated to provide the crude product as a pale yellow solid (3.6 g, 88%), which was used without further purification. Unreacted 4-phenylbutyric acid (c.a. 25 %) was also identified. ^{1}H NMR δ 8.12-7.38 (m, 1H), 7.37 – 7.11 (m, 5H), 6.05 (d, J = 7.0 Hz, 1H), 4.59 (quint, J = 7.2 Hz, 1H), 2.66 (t, J =7.4 Hz, 2H), 2.24 (t, J=7.4, 2H), 2.04 – 1.91 (m, 2H), 1.44 (d, J = 7.2 Hz, 3H). ^{13}C NMR δ 176.69, 173.48, 141.39, 128.64, 128.57, 126.19, 48.31, 35.64, 35.16, 27.03, 18.16.

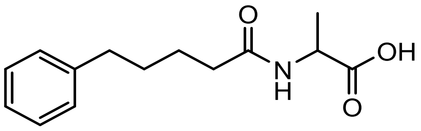

(5-Phenylpentanoyl)-D/L-alanine (83). Synthesized according to method C using compound **72** (1.1 g, 5.6 mmol) and D/L-alanine (0.6 g, 6.7 mmol). The crude product was obtained as a colourless sap (1.35 g, 97 %), which was used without further purification. Unreacted 5-phenylvaleric acid (c.a. 25 %) was also identified. ^{1}H NMR δ 11.06 (s, 1H), 7.33 – 7.23 (m, 2H), 7.23 – 7.09 (m, 3H), 6.21 (d, *J* = 7.5 Hz, 1H), 4.59 (p, *J* = 7.2 Hz, 1H), 2.68 – 2.58 (m, 2H), 2.25 (t, *J* = 7.1 Hz, 2H), 1.75 – 1.59 (m, 4H), 1.43 (d, *J* = 7.2 Hz, 3H). ^{13}C NMR δ 176.73, 173.71, 142.19, 128.50, 128.46, 125.92, 48.29, 36.33, 35.71, 30.99, 25.29, 18.23.

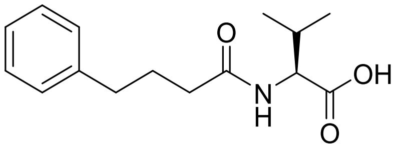

(4-Phenylbutanoyl)-L-valine (84). Synthesized according to method C using compound **71** (1.8 g, 10 mmol) and L-valine (1.3 g, 11 mmol). The crude product was obtained as a pale yellow powder (1.8 g, 71 %). Unreacted 4-phenylbutyric acid (c.a. 20 %) was also identified. ^{1}H NMR δ 7.37 – 7.10 (m, 5H), 5.99 (s, 1H), 4.59 (dd, J = 8.6, 4.8 Hz, 1H), 2.66 (t, J = 7.4 Hz, 2H), 2.27 (t, J = 7.5 Hz, 2H), 2.06 – 1.92 (m, 2H), 0.96 (dd, *J* = 15.9, 6.9 Hz, 6H). ^{13}C NMR δ 176.00, 175.97, 141.45, 128.65, 128.57, 126.17, 57.16, 35.89, 35.21, 31.07, 27.21, 19.16, 17.84.

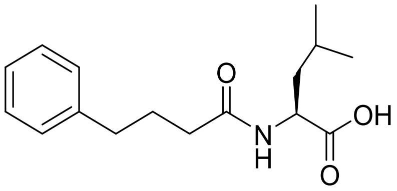

4-Phenylbutanoyl-L-leucine (85). Synthesized according to method C using compound **71** (1.8 g, 10 mmol) and L-leucine (1.4 g, 11 mmol). The crude product was obtained as a pale yellow powder (2.4 g, 88 %). ^{1}H NMR δ 7.35 – 7.04 (m, 5H), 5.87 (d, J = 7.6 Hz, 1H), 4.65-4.55 (m, 1H), 2.65 (t, J = 7.5 Hz, 2H), 2.24 (t, J = 7.5 Hz, 2H), 2.04 – 1.91 (m, 2H), 1.75 – 1.61 (m, 2H), 1.61 – 1.49 (m, 1H), 0.95 (dd, J = 6.2, 1.1 Hz, 6H). ^{13}C NMR δ 176.85, 173.64, 141.45, 128.65, 128.57, 126.17, 50.96, 41.25, 35.70, 35.17, 27.08, 25.04, 22.95, 21.98.

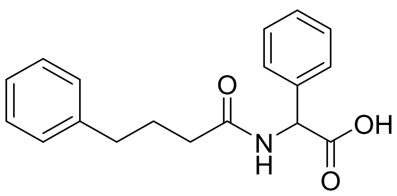

4-Phenylbutanoyl-D/L-phenylglycine (86). Synthesized according to method C using compound **71** (1.8 g, 10 mmol) and D/L-phenylglycine (1.8 g, 12 mmol). The crude product was obtained as a pale orange powder (2.1 g, 72 %). Unreacted 4-phenylbutyric acid (c.a. 50 %) was also identified. ^{1}H NMR δ 9.14 (s, 1H), 7.46 – 7.05 (m, 10H), 6.48 (d, *J* = 7.0 Hz, 1H), 5.63 (d, *J* = 7.0 Hz, 1H), 2.76 – 2.58 (m, 2H), 2.35 – 2.21 (m, 2H), 2.07 – 1.90 (m, 2H). ^{13}C NMR δ 175.08, 172.91, 141.39, 136.02, 129.21, 128.93, 128.64, 128.54, 127.51, 126.14, 56.60, 35.51, 33.36, 26.96.

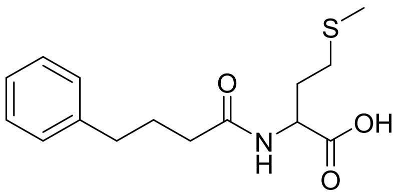

4-Phenylbutanoyl-D/L-methionine (87). Synthesized according to method C using compound **71** (1.8 g, 10 mmol) and D/L-methionine (1.8 g, 12 mmol). The crude product was obtained as a white powder (2.1 g, 72 %). ^{1}H NMR δ 7.48 – 7.04 (m, 5H), 6.37 (d, J = 7.6 Hz, 1H), 4.74 (td, J = 7.4, 5.2 Hz, 1H), 2.75 – 2.62 (m, 2H), 2.61 – 2.36 (m, 2H), 2.34 – 2.15 (m, 2H), 2.11 (s, 2H), 2.07 (s, 3H), 2.06 – 1.95 (m, 2H). ^{13}C NMR δ 175.43, 173.74, 141.36, 128.62, 128.56, 126.18, 77.16, 51.85, 35.71, 35.18, 31.25, 30.16, 27.07, 15.56.

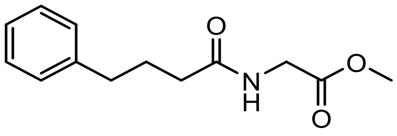

Methyl (4-phenylbutanoyl)glycinate (88). Synthesized according to method C using compound **71** (0.35 g, 1.9 mmol) and glycine methyl ester hydrochloride (0.20 g, 1.6 mmol) with a reaction time of 18 h. The crude product was obtained (quantitative), which was used without further purification. ^{1}H NMR δ 7.32 – 7.15 (m, 5H), 5.92 (s, 1H), 4.04 (d, *J* = 5.2 Hz, 2H), 3.76 (s, 3H), 2.67 (t, *J* = 7.5 Hz, 2H), 2.25 (t, *J* = 7.5 Hz, 2H), 2.04 – 1.94 (m, 2H). ^{13}C NMR *δ* 172.84, 170.55, 141.41, 128.53, 128.42, 126.00, 52.39, 41.19, 35.47, 35.10, 26.93.

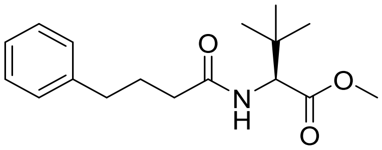

Methyl 3,3-dimethyl-2-(4-phenylbutanamido)butanoate (89). Synthesized according to method C using compound **71** (0.85 g, 4.6 mmol) and compound **77** (0.70 g, 3.9 mmol) with a reaction time of 2 d. The crude product was obtained as an orange oil (quantitative), which was used without further purification. ^{1}H NMR δ 7.36 – 7.08 (m, 6H), 5.92 (d, *J* = 9.1 Hz, 1H), 4.50 (d, *J* = 9.4 Hz, 1H), 3.72 (s, 3H), 2.66 (t, *J* = 7.5 Hz, 2H), 2.27 – 2.20 (m, 2H), 2.04 – 1.90 (m, 2H), 0.97 (s, 9H). ^{13}C NMR δ 172.36, 172.35, 141.44, 128.53, 128.43, 126.01, 59.74, 51.82, 35.91, 35.18, 34.72, 27.12, 26.58.

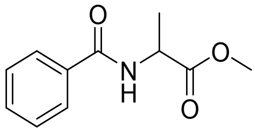

Methyl benzoylalaninate (90). Synthesized according to method C using benzoyl chloride (0.91 ml, 7.8 mmol) and D/L-alanine methyl ester hydrochloride (1.0 g, 7.1 mmol) with a reaction time of 18 h. The crude product was obtained as an orange oil (quantitative), which was used without further purification. ^{1}H NMR δ 7.89 – 7.75 (m, 2H), 7.58 – 7.49 (m, 1H), 7.49 – 7.40 (m, 2H), 4.81 (p, *J* = 7.2 Hz, 1H), 3.80 (s, 3H), 1.53 (d, *J* = 7.2 Hz, 3H). ^{13}C NMR δ 173.84, 166.91, 134.07, 131.88, 128.73, 127.17, 52.72, 48.61, 18.83.

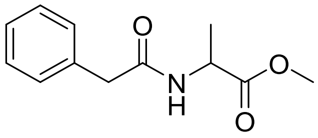

Methyl (2-phenylacetyl)alaninate (91). Synthesized according to method C using phenylacetyl chloride (0.73 ml, 5.5 mmol) and D/L-alanine methyl ester hydrochloride (0.70 g, 5.0 mmol) with a reaction time of 1 d. The crude product was obtained, which after flash chromatography (heptane/EtOAc 2:1 → 1:1) yielded compound **91** as an off white solid (0.56 g, 48 %). ^{1}H NMR δ 7.41 – 7.19 (m, 5H), 6.21 (s, 1H), 4.56 (p, *J* = 7.2 Hz, 1H), 3.70 (s, 3H), 3.57 (s, 2H), 1.33 (d, *J* = 7.2 Hz, 3H). ^{13}C NMR δ 173.38, 170.62, 134.64, 129.37, 128.94, 127.33, 52.42, 48.11, 43.45, 18.23.

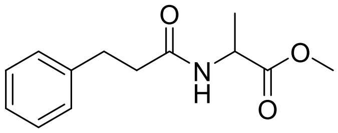

Methyl (3-phenylpropanoyl)-*DL*-alaninate (92). Synthesized according to method C using hydrocinnamoyl chloride (0.82 ml, 5.5 mmol) and D/L-alanine methyl ester hydrochloride (0.70 g, 5.0 mmol) with a reaction time of 3 d. The crude product was obtained, which after flash chromatography (heptane/EtOAc 9:1 → EtOAc) yielded compound **92** (0.91 g, 73 %). ^{1}H NMR δ 7.32 – 7.23 (m, 2H), 7.23 – 7.15 (m, 3H), 6.07 (s, 1H), 4.58 (p, *J* = 7.2 Hz, 1H), 3.76 – 3.66 (m, 3H), 2.96 (t, *J* = 7.8 Hz, 2H), 2.60 – 2.42 (m, 2H), 1.33 (d, *J* = 7.1 Hz, 3H). ^{13}C NMR δ 173.63, 171.67, 140.78, 128.58, 128.42, 126.31, 52.50, 47.99, 38.21, 31.59, 18.50.

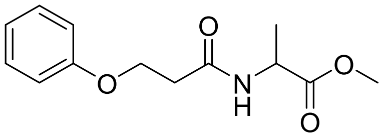

Methyl (3-phenoxypropanoyl)alaninate (93). Synthesized according to method C using compound **73** (1.1 g, 6.0 mmol) and D/L-alanine methyl ester hydrochloride (0.92 g, 6.6 mmol) with a reaction time of 19 h. The crude product was obtained as an off-white solid (1.4 g, 92 %), which was used without further purification. ^{1}H NMR δ 7.27 – 7.17 (m, 2H), 6.94 – 6.82 (m, 3H), 4.55 (p, *J* = 7.2 Hz, 1H), 4.27 – 4.13 (m, 2H), 3.67 (s, 3H), 2.63 (t, *J* = 6.0 Hz, 2H), 1.35 (d, *J* = 7.2 Hz, 3H). ^{13}C NMR δ 173.54, 170.17, 158.36, 129.66, 121.38, 114.78, 64.08, 52.59, 48.20, 36.64, 18.66.

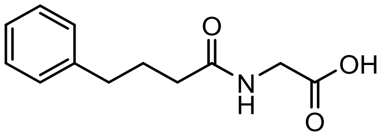

Method D: Synthesis of (4-phenylbutanoyl)glycine (94). LiOH · H_2O (100 mg, 2.39 mmol) was added to a solution of compound **88** (375 mg, 1.59 mmol) in MeOH (9 ml) and H_2O (3 ml). The mixture was left to stir at room temperature for 2 h before removing MeOH by evaporation. The aqueous phase was washed with DCM, acidified with 1 M HCl, and extracted with DCM. The organic phase was dried over anhydrous Na_2SO_4, filtered, and evaporated to provide the crude product as an off-white solid (296 mg, 84%), which was used without further purification. ^{1}H NMR (DMSO) δ 12.44 (s, 1H), 8.13 (t, J = 5.8 Hz, 1H), 7.31 – 7.13 (m, 5H), 3.72 (d, J = 5.9 Hz, 2H), 2.60 – 2.54 (m, 2H), 2.14 (t, J = 7.4 Hz, 2H), 1.85 – 1.72 (m, 2H). ^{13}C NMR (DMSO) δ 172.76, 171.92, 142.31, 128.81, 128.73, 126.20, 41.04, 35.00, 34.97, 27.53.

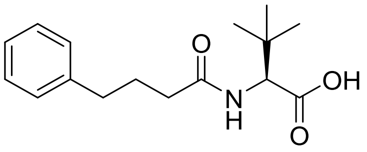

(*S*)-3,3-Dimethyl-2-(4-phenylbutanamido)butanoic acid (95). Synthesized according to method D using compound **89** (1.1 g, 3.8 mmol) with a reaction time of 1 d. The crude product was obtained (0.91 g, 85%), which was used without further purification. ^{1}H NMR δ 7.38 – 7.11 (m, 5H), 6.06 (d, J = 8.8 Hz, 1H), 4.51 (d, J = 9.2 Hz, 1H), 2.65 (t, J = 7.5 Hz, 2H), 2.33 – 2.17 (m, 2H), 2.05 – 1.91 (m, 2H), 1.02 (s, 9H). ^{13}C NMR δ 175.15, 173.28, 141.30, 128.51, 128.45, 126.05, 60.01, 35.86, 35.13, 34.51, 27.10, 26.60.

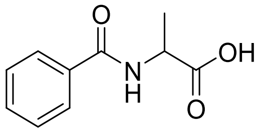

Benzoylalanine (96). Synthesized according to method D using compound **90** (1.5 g, 7.1 mmol) with a reaction time of 2.5 h. The crude product was obtained as a white solid (quantitative), which was used without further purification. ^{1}H NMR (Methanol-d_4) δ 7.90 – 7.79 (m, 2H), 7.56 – 7.40 (m, 3H), 4.60 (qd, J = 7.3, 2.6 Hz, 1H), 1.51 (d, J = 7.3 Hz, 3H). ^{13}C NMR (Methanol-d_4) δ 176.19, 170.10, 135.29, 132.79, 129.51, 128.48, 49.94, 17.50.

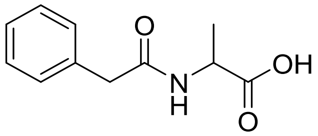

(2-Phenylacetyl)alanine (97). Synthesized according to method D using compound **91** (516 mg, 2.3 mmol) with a reaction time of 1 d. The crude product was obtained (465 mg, 96 %), which was used without further purification. ^{1}H NMR (Methanol-d_4) δ 7.47 – 7.15 (m, 5H), 4.37 (q, J = 7.3 Hz, 1H), 3.54 (s, 2H), 1.38 (d, J = 7.3 Hz, 3H). ^{13}C NMR (Methanol-d_4) δ 175.93, 173.75, 136.80, 130.15, 129.50, 127.82, 49.43, 43.39, 17.65.

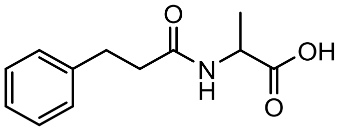

(3-Phenylpropanoyl)-*DL*-alanine (98). Synthesized according to method D using compound **92** (0.91 g, 3.9 mmol) with a reaction time of 2 h. The crude product was obtained as a white foam (0.83 g, 96%), which was used without further purification. ^{1}H NMR (Methanol-d_4) δ 7.30 – 7.10 (m, 5H), 4.36 (q, J = 7.3 Hz, 1H), 2.96 – 2.83 (m, 2H), 2.57 – 2.43 (m, 2H), 1.32 (d, J = 7.3 Hz, 3H). ^{13}C NMR (Methanol-d_4) δ 174.68, 173.58, 140.82, 128.04, 127.98, 125.76, 47.85, 37.24, 31.37, 16.24.

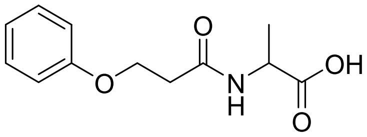

(3-Phenoxypropanoyl)-alanine (99). Synthesized according to method D using compound **93** (1.4 g, 5.5 mmol) with a reaction time of 2 h. The crude product was obtained as a white solid (1.2 g, 90 %), which was used without further purification. ^{1}H NMR (Methanol-d_4) δ 7.29 – 7.18 (m, 2H), 6.95 – 6.85 (m, 3H), 4.42 (q, J = 7.3 Hz, 1H), 4.29 – 4.14 (m, 2H), 2.77 – 2.60 (m, 2H), 1.40 (d, J = 7.3 Hz, 3H). ^{13}C NMR (Methanol-d_4) δ 176.02, 173.09, 160.12, 130.43, 121.89, 115.62, 65.02, 49.36, 36.81, 17.72.

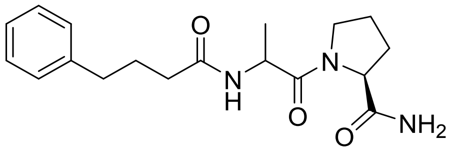

Method E: Synthesis of (2*S*)-1-((4-phenylbutanoyl)-D/L-alanyl)pyrrolidine-2-carboxamide (1). Pivaloyl chloride (0.72 ml, 5.8 mmol) was added to a solution of compound **82** (1.36 g, 5.8 mmol) and Et_3N (0.96 ml, 7.3 mmol) in anhydrous DCM (20 ml) at 0 °C. The mixture was left to stir at 0 °C for 1 h. A solution of L-prolinamide (0.83 g, 7.3 mmol) and Et_3N (0.96 ml, 7.3 mmol) in anhydrous DCM (80 ml) was added. The mixture was raised to room temperature and left to stir for 3 h. The mixture was diluted with DCM and the organic phase was washed with a 10% aqueous solution of citric acid, a saturated solution of $NaHCO_3$, and brine, dried over anhydrous Na_2SO_4, filtered, and evaporated to provide the crude product, which after flash chromatography (EtOAc/MeOH 9:1) yielded compound **1** as a white powder (1.42 g, 74 %). ^{1}H NMR δ 7.32 – 7.23 (m, 2H), 7.23 – 7.13 (m, 3H), 6.93 (s, 0.65H), 6.65 (s, 0.35H), 6.50 (d, J = 6.1 Hz, 0.65H), 6.43 (d, J = 7.5 Hz, 0.35H), 5.71 (s, 0.35H), 5.59 (s, 0.6H), 4.75 (p, J = 7.0 Hz, 0.35H), 4.60 – 4.51 (m, 1.65H), 3.93 (ddd, J = 10.3, 7.4, 3.0 Hz, 0.65H), 3.75 – 3.65 (m, 0.35H), 3.62 – 3.44 (m, 1H), 2.69 – 2.58 (m, 2H), 2.37 – 2.28 (m, 1H), 2.25 – 1.89 (m, 7H), 1.37 – 1.32 (m, 3H) (two rotamers 13:7). ^{13}C NMR δ 173.84, 173.71, 173.31, 172.94, 172.43, 172.27, 141.56, 141.48, 128.61, 128.54, 128.51, 126.12, 126.09, 60.37, 59.66, 47.65, 47.43, 47.16, 46.64, 35.81, 35.40, 35.32, 35.22, 28.91, 27.37, 27.15, 27.09, 25.18, 24.48, 18.38, 16.75 (additional set of signals (ca. 35 %) from minor stereisomer).

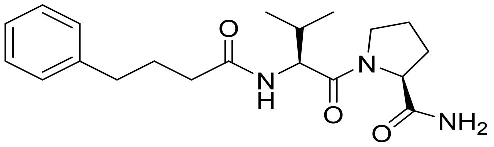

4-Phenylbutanoyl-L-valinyl-L-prolinamide (38). Synthesized according to method E using compound **84** (1.00 g, 3.8 mmol). The crude product was obtained, which after flash chromatography (EtOAc/MeOH 9:1) yielded compound **38** as a white solid (1.23 g, 90 %). ^{1}H NMR δ 7.32 – 7.22 (m, 2H), 7.22 – 7.12 (m, 3H), 7.06 (s, 0.3H), 6.91 (s, 0.7H), 6.50 (d, J = 6.9 Hz, 0.7H), 6.31 (d, J = 8.8 Hz, 0.3H), 5.69 (s, 0.3H), 5.54 (s, 0.7H), 4.65 – 4.59 (m, 0.3H), 4.58 – 4.51 (m, 1H), 4.30 – 4.22 (m, 0.7H), 4.08 – 3.98 (m, 0.7H), 3.87 – 3.77 (m, 0.3H), 3.65 – 3.50 (m, 1H), 2.73 – 2.55 (m, 2H), 2.45 – 1.74 (m, 8H), 1.31 – 1.23 (m, 1H), 1.06 – 0.95 (m, 5H) (two rotamers 7:3). ^{13}C NMR δ 174.45, 173.95, 171.99, 141.46, 128.60, 128.55, 126.13, 60.34, 57.60, 47.49, 35.45, 35.17, 30.34, 29.17, 27.17, 24.39, 19.39, 18.93 (signals only reported for major rotamer).

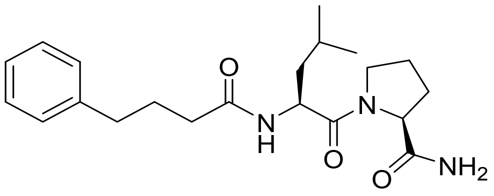

4-Phenylbutanoyl-L-leucinyl-L-prolinamide (39). Synthesized according to method E using compound **85** (1.40 g, 5.1 mmol,). The crude product was obtained, which after flash chromatography (EtOAc/MeOH 23:2) yielded compound **39** as a white solid (1.23 g, 90 %). ^{1}H NMR δ 7.31 – 7.22 (m, 2H), 7.22 – 7.11 (m, 3H), 6.94 (s, 0.7H), 6.67 (s, 0.3H), 6.59 (d, *J* = 6.6 Hz, 0.7H), 6.30 (d, *J* = 8.5 Hz, 0.3H), 5.79 (s, 0.3H), 5.64 (s, 0.7H), 4.82 (ddd, *J* = 10.0, 8.5, 4.2 Hz, 0.3H), 4.58 – 4.48 (m, 1.7H), 4.02 (td, *J* = 9.0, 7.6, 2.9 Hz, 0.7H), 3.84 – 3.75 (m, 0.3H), 3.60 – 3.43 (m, 1H), 2.64 – 2.58 (m, 2H), 2.35 – 1.86 (m, 8H), 1.75 – 1.39 (m, 3H), 1.00 – 0.91 (m, 6H) (two rotamers 7:3). ^{13}C NMR δ 174.43, 173.99, 173.47, 173.11, 172.83, 172.53, 141.59, 141.49, 128.60, 128.52, 128.50, 126.11, 126.07, 60.44, 59.61, 50.43, 48.97, 47.44, 47.11, 41.99, 40.26, 35.74, 35.38, 35.31, 35.19, 29.06, 27.35, 27.13, 25.13, 24.93, 24.88, 24.41, 23.50, 21.92, 21.87 (second set of signals (ca. 30 %) from minor rotamer).

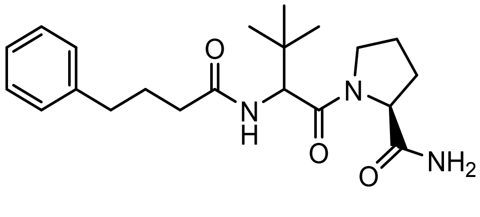

(2*S*)-1-(3,3-Dimethyl-2-(4-phenylbutanamido)butanoyl)pyrrolidine-2-carboxamide (40). Synthesized according to method E using compound **95** (500 mg, 1.80 mmol). The crude product was obtained as an orange oil, which after flash chromatography (EtOAc/MeOH 97:3 → 19:1) yielded **40** as a white foam (311 mg, 46 %). ^{1}H NMR δ 7.34 – 7.24 (m, 2H), 7.24 – 7.14 (m, 3H), 6.90 (s, 0.8H), 6.73 (s, 0.2H), 6.15 (d, *J* = 9.6 Hz, 0.2H), 6.07 (d, *J* = 7.3 Hz, 0.8H), 5.47 (s, 0.2H), 5.34 (s, 0.8H), 4.70 (d, *J* = 9.4 Hz, 0.2H), 4.62 – 4.51 (m, 1H), 4.36 (d, *J* = 7.3 Hz, 0.8H), 4.09 – 4.00 (m, 0.8H), 3.88 – 3.78 (m, 0.2H), 3.70 – 3.52 (m, 1H), 2.71 – 2.57 (m, 2H), 2.40 – 1.84 (m, 8H), 1.05 (s, 7.2H), 1.01 (s, 1.8H) (two rotamers 4:1). ^{13}C NMR δ 174.36, 173.93, 173.16, 172.67, 171.93, 171.08, 141.36, 141.35, 128.64, 128.62, 128.58, 128.55, 126.19, 126.14, 60.41, 59.43, 58.67, 56.78, 48.70, 48.02, 35.95, 35.63, 35.52, 35.35, 35.06, 34.15, 29.18, 27.24, 27.12, 26.99, 26.70, 26.59, 25.23, 24.48 (additional set of signals (ca. 20 %) from minor rotamer).

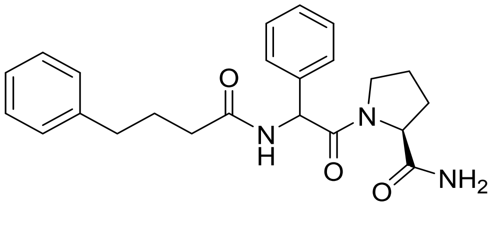

4-Phenylbutanoyl-D/L-phenylglycinyl-L-prolineamide (41). Synthesized according to method E using compound **86** (2.13 g, 7.1 mmol). The crude product was obtained, which after flash chromatography (EtOAc/MeOH 39:1 -> 183:17) yielded compound **41** as a white solid (2.04 g, 72 %). ^{1}H NMR δ 7.39 – 7.02 (m, 10H), 7.02 – 6.83 (m, 1H), 6.85-6.65 (m, 1H), 5.74 – 5.41 (m, 1H), 4.63 – 4.39 (m, 1H), 3.82-3.1 (m, 2H), 2.65-2.48 (m, 2 H), 2.41 – 2.06 (m, 4H), 2.06 – 1.61 (m, 6H) (rotamers visible but could not be separately integrated). ^{13}C NMR δ 173.78, 173.60, 173.22, 173.01, 172.07, 169.76, 141.59, 141.49, 136.72, 135.40, 129.36, 129.34, 128.99, 128.60, 128.59, 128.51, 128.48, 128.45, 128.28, 128.02, 126.10, 126.05, 60.51, 60.49, 59.37, 56.33, 47.52, 47.01, 35.39, 35.24, 35.13, 33.72, 28.53, 27.26, 27.01, 26.13, 25.02, 24.49 (additional set of signals (c.a. 35 %) from minor rotamer).

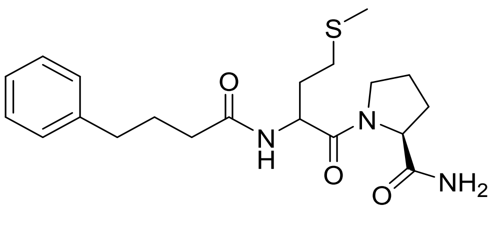

4-Phenylbutanoyl-D/L-methionine-L-prolineamide (42). Synthesized according to method E using compound **87** (2.10 g, 7.14 mmol). The crude product was obtained (2.63 g, 94 %), which was used without further purification. ^{1}H NMR δ 7.26 – 7.15 (m, 2H), 7.14 – 7.05 (m, 3H), 7.02 – 6.96 (m, 0.5H), 6.87 – 6.77 (m, 0.5H), 5.98 – 5.68 (m, 1H), 4.68 – 4.57 (m, 0.5H), 4.52 – 4.47 (m, 0.5H), 4.46 – 4.38 (m, 0.5H), 3.98 – 3.89 (m, 0.5H), 3.78 – 3.44 (m, 1H), 3.44 – 3.35 (m, 0.5H), 3.26 (td, *J* = 9.5, 7.0 Hz, 0.5H), 2.67 – 2.43 (m, 4H), 2.32 – 2.08 (m, 4H), 2.06 – 1.70 (m, 8H) (two rotamers 1:1). ^{13}C NMR δ 174.34, 174.08, 174.02, 173.11, 172.85, 171.65, 141.58, 141.49, 128.57, 128.56, 128.49, 128.47, 126.08, 126.07, 60.51, 59.42, 50.97 (two signals), 47.50, 47.36, 35.34, 35.23, 35.21, 33.71, 30.62, 30.43, 29.14, 27.54, 27.50, 27.09, 26.60, 26.12, 24.99, 24.43, 15.77, 15.71 (two equal sets of signals from rotamers).

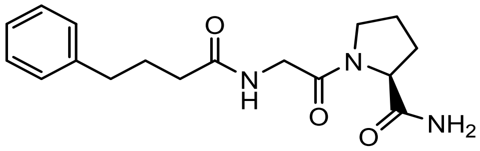

(*S*)-1-((4-Phenylbutanoyl)glycyl)pyrrolidine-2-carboxamide (43). Synthesized according to method E using compound **94** (286 mg, 1.29 mmol). The crude product was obtained as a white solid, which after flash chromatography (DCM → DCM/MeOH 4:1) yielded compound **43** as a white solid (149 mg, 41 %). ^{1}H NMR *δ* 7.33 – 7.13 (m, 6H), 6.71 (s, 1H), 6.46 (s, 1H), 5.52 (s, 1H), 4.56 (dd, *J* = 8.1, 2.3 Hz, 1H), 4.04 (d, *J* = 2.8 Hz, 2H), 3.64 – 3.38 (m, 2H), 2.66 (t, *J* = 7.6 Hz, 2H), 2.41 – 2.33 (m, 1H), 2.30 – 2.23 (m, 2H), 2.18 – 1.88 (m, 6H). ^{13}C NMR *δ* 173.02, 172.92, 168.44, 141.42, 128.51, 128.41, 125.99, 59.89, 46.50, 42.09, 35.59, 35.23, 27.68, 27.08, 24.77.

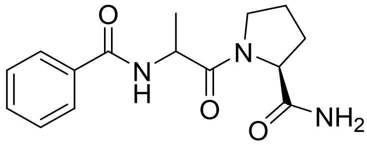

(2*S*)-1-(Benzoylalanyl)pyrrolidine-2-carboxamide (44). Synthesized according to method E using compound **96** (0.80 g, 4.14 mmol). The crude product was obtained as an off white foam, which after flash chromatography (EtOAc/MeOH 19:1 → 4:1) yielded compound **44** as a white foam (1.04 g, 87 %). ^{1}H NMR δ 7.88 – 7.71 (m, 2H), 7.59 – 7.33 (m, 4H), 7.00 (s, 1H), 5.65 (s, 1H), 4.78 (p, *J* = 6.8 Hz, 1H), 4.66 – 4.48 (m, 1H), 3.82 – 3.38 (m, 2H), 2.43 – 2.25 (m, 1H), 2.22 – 1.77 (m, 3H), 1.51 – 1.43 (m, 3H). ^{13}C NMR δ 173.86, 172.61, 168.01, 133.44, 131.94, 128.61, 127.36, 60.45, 48.27, 47.22, 27.57, 24.53, 14.32.

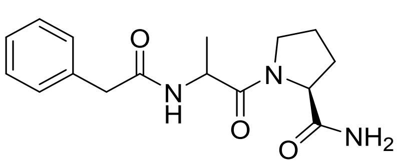

(2*S*)-1-((2-Phenylacetyl)alanyl)pyrrolidine-2-carboxamide (45). Synthesized according to method E using compound **97** (444 mg, 2.14 mmol). The crude product was obtained as a white foam, which after flash chromatography (EtOAc/MeOH 19:1 → 4:1) yielded compound **45** as a white foam (436 mg, 67 %). ^{1}H NMR δ 7.41 – 7.20 (m, 5H), 6.92 – 6.68 (m, 1.5H), 6.67 – 6.46 (m, 0.5H), 5.79 (s, 0.25H), 5.56 (s, 0.75H), 4.67 – 4.47 (m, 2H), 3.99 – 3.85 (m, 0.5H), 3.77 – 3.40 (m, 3.5H), 2.37 – 2.23 (m, 1H), 2.18 – 1.78 (m, 3H), 1.32 – 1.26 (m, 3H) (two rotamers 3:1). ^{13}C NMR δ 173.74, 173.30, 172.50, 172.22, 171.95, 170.39, 134.69, 134.66, 129.33, 129.25, 128.94, 128.88, 127.30, 127.27, 60.32, 59.54, 47.75, 47.28, 47.04, 46.79, 43.55, 43.01, 28.86, 27.46, 25.04, 24.34, 18.09, 16.43. (additional set of signals (ca. 25 %) from minor rotamer).

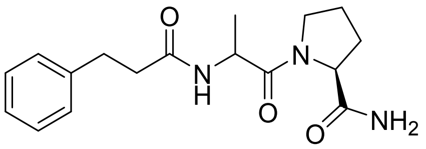

(2*S*)-1-((3-Phenylpropanoyl)alanyl)pyrrolidine-2-carboxamide (46). Synthesized according to method E using compound **98** (819 mg, 3.70 mmol). The crude product was obtained as a white foam, which after flash chromatography (EtOAc/MeOH 9:1 → 7:3) yielded compound **46** as a white foam (500 mg, 48 %). ^{1}H NMR δ 7.38 – 7.25 (m, 2H), 7.25 – 7.15 (m, 3H), 6.83 (s, 0.7H), 6.80 – 6.56 (m, 1H), 6.46 (s, 0.3H), 5.77 (s, 0.3H), 5.50 (s, 0.7H), 4.76 (p, *J* = 7.0 Hz, 0.3H), 4.63 – 4.47 (m, 1.7H), 3.98 – 3.88 (m, 0.6H), 3.76 – 3.43 (m, 1.4H), 3.03 – 2.87 (m, 2H), 2.61 – 2.42 (m, 2H), 2.40 – 2.26 (m, 1H), 2.23 – 1.76 (m, 3H), 1.35 – 1.27 (m, 3H) (two rotamers 7:3). ^{13}C NMR δ 174.12, 173.82, 173.05, 172.84, 172.48, 171.61, 140.81, 140.73, 128.66, 128.62, 128.46, 128.44, 126.41, 126.36, 60.42, 59.67, 47.63, 47.42, 47.19, 46.65, 38.23, 37.66, 31.66, 31.53, 28.93, 27.46, 25.16, 24.49, 18.29, 16.70 (additional set of signals (ca. 30 %) from minor rotamer).

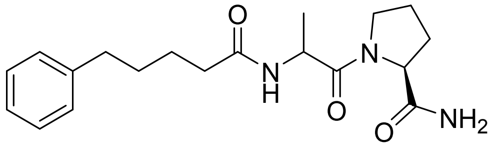

(2*S*)-1-((5-Phenylpentanoyl)alanyl)pyrrolidine-2-carboxamide (47). Synthesized according to method E using compound **83** (1.34 g, 5.4 mmol). The crude product was obtained, which after flash chromatography (EtOAc/MeOH 19:1 → 4:1) yielded compound **47** as a colorless sap (0.87 g, 47 %). ^{1}H NMR δ 7.32 – 7.21 (m, 2H), 7.21 – 7.11 (m, 3H), 6.90 (s, 0.8H), 6.62 (d, *J* = 6.4 Hz, 1.2H), 6.47 (d, *J* = 7.7 Hz, 0.2H), 5.81 (s, 0.2H), 5.56 (s, 0.8H), 4.75 (p, *J* = 7.0 Hz, 0.2H), 4.62 – 4.48 (m, 1.8H), 3.93 (td, *J* = 8.8, 7.5, 2.9 Hz, 0.8H), 3.75 – 3.65 (m, 0.2H), 3.62 – 3.42 (m, 1H), 2.67 – 2.56 (m, 2H), 2.37 – 2.27 (m, 1H), 2.26 – 2.16 (m, 2H), 2.16 – 1.83 (m, 3H), 1.73 – 1.55 (m, 4H), 1.36 – 1.31 (m, 3H) (two rotamers 4:1). ^{13}C NMR δ 173.91, 173.83, 173.38, 172.94, 172.50, 172.43, 142.29, 142.25, 128.51, 128.50, 128.45, 128.42, 125.91, 125.88, 60.37, 59.67, 47.62, 47.41, 47.15, 46.63, 36.41, 36.00, 35.77, 35.75, 31.13, 31.06, 28.93, 27.57, 27.43, 25.31, 25.16, 24.46, 18.35, 16.71 (additional set of signals (ca. 20 %) from minor rotamer).

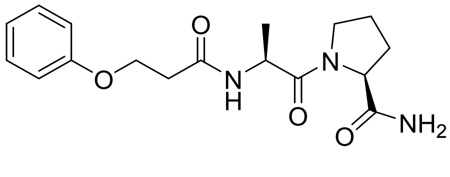

(*S*)-1-((3-Phenoxypropanoyl)-L-alanyl)pyrrolidine-2-carboxamide (48). Synthesized according to method E using compound **99** (1.17 g, 4.9 mmol) with ethyl chloroformate (0.47 ml, 4.9 mmol) replacing pivaloyl chloride. The crude product was obtained as a white foam, which after flash chromatography (EtOAc/MeOH 49:1 → 4:1) yielded compound **48** as a white foam (1.3 g, 79 %). ^{1}H NMR δ 7.34 – 7.25 (m, 2H), 7.08 (d, *J* = 6.1 Hz, 0.75H), 7.01 – 6.87 (m, 3.25H), 5.78 (s, 0.25H), 5.57 (s, 0.75H), 4.80 (p, *J* = 7.3 Hz, 0.25H), 4.67 – 4.53 (m, 1.75H), 4.30 – 4.20 (m, 2H), 3.99 – 3.89 (m, 0.75H), 3.74 – 3.66 (m, 0.25H), 3.65 – 3.43 (m, 1H), 2.76 – 2.63 (m, 2H), 2.44 – 2.28 (m, 1H), 2.16 – 1.87 (m, 3H), 1.43 – 1.32 (m, 3H) (two rotamers 3:1). ^{13}C NMR δ 173.71, 173.30, 172.61, 172.21, 171.18, 169.88, 158.34, 158.29, 129.56, 129.52, 121.24, 121.17, 114.67, 114.61, 63.92, 63.84, 60.28, 59.57, 47.62, 47.32, 47.10, 46.76, 36.48, 36.07, 28.72, 27.31, 25.06, 24.40, 18.23, 16.71 (additional set of signals (ca. 25 %) from minor rotamer).

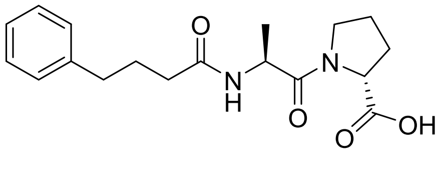

(4-Phenylbutanoyl)-L-alanyl-D-proline (100). Synthesized according to method E using compound **82** (1.34 g, 5.7 mmol) with D-proline (0.72 g, 6.3 mmol) replacing L-prolinamide. The crude product was obtained as an orange sap (quantitative), which was used without further purification or characterization.

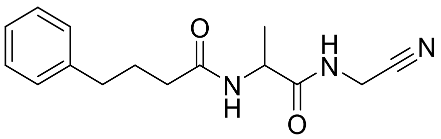

***N*-(1-((Cyanomethyl)amino)-1-oxopropan-2-yl)-4-phenylbutanamide (36).** Synthesized according to method E using compound **82** (0.28 g, 1.2 mmol) with aminoacetonitrile hydrochloride (0.12 g, 1.3 mmol) replacing L-prolinamide. The crude product was obtained as a brown oil, which after flash chromatography (heptane/EtOAc 9:1 → EtOAc) yielded compound **36** as a white solid (0.10 g, 31 %). ^{1}H NMR *δ* 8.13–8.10 (m, 1H), 7.29–7.24 (m, 2H), 7.20–7.14 (m, 3H), 6.71–6.68 (m, 1H), 4.63–4.57 (m, 1H), 4.13–4.01 (m, 2H), 2.66–2.61 (m, 2H), 2.26–2.19 (m, 2H), 1.99–1.91 (m, 2H), 1.36–1.32 (m, 3H). ^{13}C NMR δ 173.51, 173.19, 141.27, 128.56, 128.53, 126.19, 116.08, 48.45, 35.56, 35.18, 27.59, 27.08, 18.13.

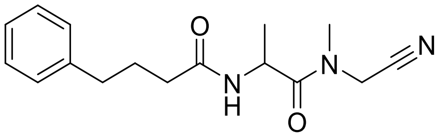

***N*-(1-((Cyanomethyl)(methyl)amino)-1-oxopropan-2-yl)-4-phenylbutanamide (37).** Synthesized according to method E using compound **82** (0.28 g, 1.2 mmol) with methylaminoacetonitrile hydrochloride (0.14 g, 1.3 mmol) replacing L-prolinamide. The crude product was obtained as a yellow oil, which after flash chromatography (heptane/EtOAc 9:1 → EtOAc) yielded compound **37** as a colourless oil (0.30 g, 86 %). ^{1}H NMR *δ* 7.30–7.26 (m, 2H), 7.21–7.16 (m, 3H), 6.34 (d, *J* = 7.6 Hz, 1H), 4.97–4.90 (m, 1H), 4.44 (d, *J* = 17.2 Hz, 1H), 4.23 (d, *J* = 17.2 Hz, 1H), 3.21 (s, 3H), 2.65 (t, *J* = 7.6 Hz, 2H), 2.23–2.19 (m, 2H), 2.04–1.93 (m, 2H), 1.33 (d, *J* =6.8 Hz, 3H). ^{13}C NMR δ 173.24, 172.16, 141.48, 128.62, 128.53, 126.12, 114.89, 45.08, 35.79, 35.76, 35.37, 35.28, 27.10, 18.57.

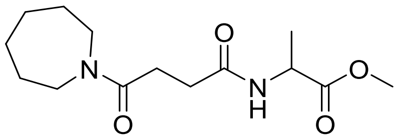

Methyl (4-(azepan-1-yl)-4-oxobutanoyl)-DL-alaninate (101). Synthesized according to Method E using compound **75** (4.1 g, 21 mmol). The crude product was obtained as a colourless oil (quantitative), which was used without further purification. ^{1}H NMR δ 6.72 (d, *J* = 7.3 Hz, 1H), 4.55 (p, *J* = 7.2 Hz, 1H), 3.73 (s, 3H), 3.57 – 3.48 (m, 2H), 3.48 – 3.41 (m, 2H), 2.78 – 2.55 (m, 4H), 1.79 – 1.65 (m, 4H), 1.61 – 1.49 (m, 4H), 1.39 (d, *J* = 7.2 Hz, 3H). ^{13}C NMR δ 173.58, 172.43, 171.44, 52.44, 48.17, 47.90, 46.29, 31.54, 29.01, 28.88, 27.66, 27.19, 26.96, 18.37.

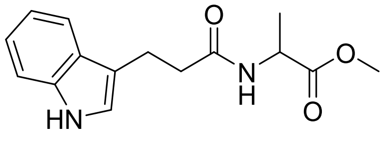

Methyl (3-(1H-indol-3-yl)propanoyl)-*DL*-alaninate (102). Synthesized according to method E using 3-indole propionic acid (4.0 g, 21 mmol). The crude product was obtained as a white foam (5.7 g, 99%), which was used without further purification. ^{1}H NMR δ 8.04 (s, 1H), 7.56 – 7.49 (m, 1H), 7.27 (dt, *J* = 8.1, 0.9 Hz, 1H), 7.11 (ddd, *J* = 8.2, 7.0, 1.2 Hz, 1H), 7.04 (ddd, *J* = 8.0, 7.0, 1.1 Hz, 1H), 6.93 (dd, *J* = 2.2, 1.1 Hz, 1H), 5.94 – 5.80

(m, 1H), 4.50 (p, J = 7.2 Hz, 1H), 3.63 (s, 3H), 3.09 – 2.97 (m, 2H), 2.59 – 2.48 (m, 2H), 1.23 (d, J = 7.2 Hz, 3H). ^{13}C NMR δ 173.58, 172.21, 136.35, 127.16, 122.05, 121.74, 119.34, 118.71, 114.89, 111.20, 52.43, 47.95, 37.16, 21.14, 18.44.

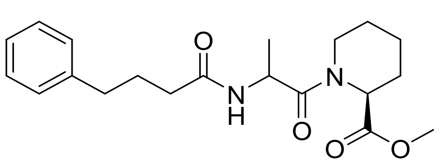

Methyl (2S)-1-((4-phenylbutanoyl)alanyl)piperidine-2-carboxylate (103). Synthesized according to method E using compound **82** (1.76 g, 7.5 mmol) and compound **79** (1.61 g, 9.0 mmol). The crude product was obtained as a brown oil, which after flash chromatography (heptane/EtOAc 3:2) yielded compound **103** (1.07 g, 40 %). ^{1}H NMR δ 7.32 – 7.24 (m, 2H), 7.23 – 7.14 (m, 3H), 6.71 – 6.55 (m, 1H), 5.39 – 5.33 (m, 0.5H), 5.32 – 5.27 (m, 0.5H), 5.03 – 4.89 (m, 1H), 3.86 – 3.74 (m, 1H), 3.74 – 3.69 (m, 3H), 3.31 – 3.18 (m, 1H), 2.69 – 2.59 (m, 2H), 2.33 – 2.25 (m, 1H), 2.25 – 2.18 (m, 2H), 2.02 – 1.92 (m, 2H), 1.80 – 1.37 (m, 5H), 1.37 – 1.32 (m, 3H) (two rotamers 1:1). ^{13}C NMR δ 172.71, 172.47, 171.93, 171.82, 171.44, 171.33, 141.62, 141.61, 128.62, 128.61, 128.49, 128.49, 126.05, 126.04, 52.58, 52.51, 52.45, 52.31, 45.49, 45.32, 43.55, 43.49, 36.05, 36.04, 35.36, 35.35, 27.27, 27.25, 26.68, 26.51, 25.27, 25.24, 21.04, 21.01, 19.77, 18.37 (two sets of signals from rotamers).

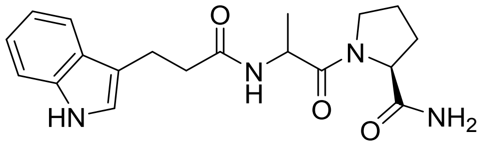

(2*S*)-1-((3-(1*H*-Indol-3-yl)propanoyl)alanyl)pyrrolidine-2-carboxamide (53). Compound **102** (5.7 g, 21 mmol) was demethylated according to method D with a reaction time of 4 h. The crude product was obtained as a white solid, which was used without further purification. The reaction was continued according to method E using the intermediate. The crude product was obtained as a white solid, which after flash chromatography ($CHCl_3$/EtOH 9:1) yielded compound **53** as a white foam (2.1 g, 58 %). ^{1}H NMR δ 8.82 (s, 0.2H), 8.70 (s, 0.8H), 7.58 – 7.46 (m, 1H), 7.33 – 7.23 (m, 1H), 7.17 – 7.08 (m, 1H), 7.08 – 7.00 (m, 1H), 6.94 – 6.88 (m, 1.2H), 6.88 – 6.80 (m, 0.8H), 6.66 (s, 1H), 6.15 (s, 1H), 4.65 (p, J = 7.0 Hz, 0.8H), 4.34 (dd, J = 8.0, 3.6 Hz, 0.8H), 4.27 – 4.16 (m, 0.4H), 3.63 – 3.36 (m, 2H), 3.14 – 2.93 (m, 2H), 2.65 – 2.46 (m, 2H), 2.10 – 1.70 (m, 4H), 1.29 – 1.14 (m, 3H) (two rotamers 4:1). ^{13}C NMR δ 174.21, 174.19, 173.71, 172.72, 172.61, 171.99, 136.44, 136.37, 127.27, 127.21, 122.30, 121.94, 121.85 (2 signals), 119.12 (2 signals), 118.71, 118.57, 114.63, 114.31, 111.45, 111.35, 60.60, 59.78, 48.06, 47.34, 46.95, 46.76, 36.95, 36.45, 31.59, 28.24, 24.99, 22.12, 21.21, 20.92, 17.68, 16.75 (additional set of signals (c.a. 20 %) from minor rotamer).

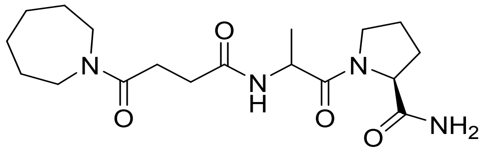

(2*S*)-1-((4-(Azepan-1-yl)-4-oxobutanoyl)alanyl)pyrrolidine-2-carboxamide (54). Compound **101** (5.9 g, 21 mmol) was demethylated according to method D with a reaction time of 2.5 h. The crude product was obtained as a white solid, which was used without further purification. The reaction was continued according to method E using the intermediate. The crude product was obtained as an off-white foam, which after flash chromatography (EtOAc/MeOH 19:1 → 3:2) yielded compound **54** as a white foam (3.2 g, 93 %). ^{1}H NMR δ 7.31 (d, J = 6.1 Hz, 0.65H), 7.12 (d, J = 7.5 Hz, 0.35H), 7.08 – 7.00 (m, 0.65H), 6.97 (s, 0.35H), 5.63 (s, 1H), 4.76 (p, J = 7.0 Hz, 0.35H), 4.65 – 4.49 (m, 1.65H), 3.99 – 3.90 (m, 0.65H), 3.78 – 3.67 (m, 0.35H), 3.65 – 3.37 (m, 6H), 2.75 – 2.48 (m, 4H), 2.36 – 2.21 (m, 1H), 2.20 – 1.93 (m, 3H), 1.79 – 1.63 (m, 4H), 1.63 – 1.48 (m, 4H),

1.39 – 1.32 (m, 3H) (two rotamers 13:7). ^{13}C NMR δ 174.12, 173.57, 173.50, 172.78, 172.36, 172.17, 171.47, 171.46, 60.48, 59.61, 47.90, 47.54, 47.42, 47.18, 46.77, 46.23, 31.31, 31.11, 29.06, 29.02, 28.96, 28.75, 28.64, 27.66, 27.59, 27.19, 26.98, 26.96, 25.18, 24.51, 18.16, 16.63 (additional set of signals (35 %) from minor rotamer).

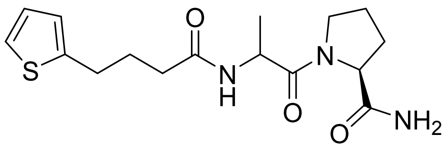

(2*S*)-1-((4-(2-Thienyl)butanoyl)alanyl)pyrrolidine-2-carboxamide (52). Compound **74** (698 mg, 3.7 mmol) was added slowly to a solution of compound **81** (1.11 g, 3.7 mmol) and Et_3N (3.66 ml, 26 mmol) in anhydrous DCM (20 ml) at 0 °C. The mixture was left to stir at room temperature overnight, before diluting with DCM. The organic phase was washed with a 20 % aqueous solution of citric acid, a saturated solution of $NaHCO_3$, and brine, dried over anhydrous Na_2SO_4, filtered, and evaporated to provide the crude product as a dark brown oil, which after flash chromatography (EtOAc/MeOH 9:1 → 17:3) yielded compound **52** (481 mg, 39 %). ^{1}H NMR δ 7.15 – 7.07 (m, 1H), 6.99 – 6.86 (m, 1.6H), 6.86 – 6.75 (m, 1H), 6.66 (s, 0.4H), 6.56 (d, *J* = 6.0 Hz, 0.6H), 6.46 (d, *J* = 7.6 Hz, 0.4H), 5.73 (s, 0.4H), 5.61 (s, 0.6H), 4.76 (p, *J* = 7.0 Hz, 0.4H), 4.63 – 4.48 (m, 1.6H), 3.99 – 3.87 (m, 0.6H), 3.76 – 3.66 (m, 0.4H), 3.65 – 3.43 (m, 1H), 2.95 – 2.74 (m, 2H), 2.41 – 1.78 (m, 8H), 1.37 – 1.31 (m, 3H) (two rotamers 3:2). ^{13}C NMR δ 173.74, 173.36, 173.22, 172.80, 172.33, 171.90, 144.18, 144.10, 126.84, 126.81, 124.61, 124.57, 123.25, 123.23, 60.28, 59.56, 47.57, 47.32, 47.06, 46.56, 35.34, 34.93, 29.16, 29.07, 28.81, 27.35, 27.32, 27.29, 25.07, 24.38, 18.25, 16.63 (second set of signals (40 %) from minor rotamer).

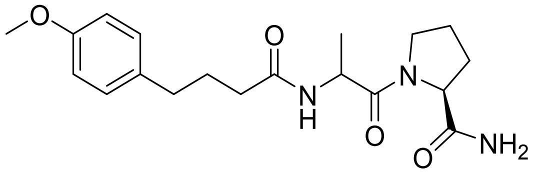

Method F: Synthesis of (2*S*)-1-((4-(4-Methoxyphenyl)butanoyl)alanyl)pyrrolidine-2-carboxamide (49). 4-(4-Methoxyphenyl)butyric acid (0.30 g, 1.55 mmol), EDC hydrochloride (0.31 g, 1.62 mmol), and HOBt hydrate (0.25 g, 1.85 mmol) were dissolved in anhydrous MeCN (15 ml) at 0 °C. The mixture was left to stir at 0 °C for 1 h, before adding a solution of **81** (0.51 g, 1.70 mmol) and DIPEA (0.80 ml, 4.62 mmol) in anhydrous MeCN (5 ml). Stirring was continued at 0 °C for 1 h and the mixture was stored at 0 °C without stirring overnight. The solvents were removed by evaporation and the resulting residue was dissolved in EtOAc. The organic phase was washed with a 20 % aqueous solution of citric acid, a saturated solution of $NaHCO_3$, and brine, dried over anhydrous Na_2SO_4, filtered, and evaporated to provide the crude product as a colourless oil, which after flash chromatography (EtOAc/MeOH 19:1 → 7:3) yielded compound **49** as a white solid (0.21 g, 38 %). ^{1}H NMR δ 7.14 – 7.02 (m, 2H), 6.90 – 6.66 (m, 4H), 6.39 – 6.05 (m, 1H), 4.81 – 4.65 (m, 1H), 4.57 – 4.45 (m, 1H), 3.81 – 3.76 (m, 3H), 3.75 – 3.66 (m, 1H), 3.62 – 3.50 (m, 1H), 2.64 – 2.51 (m, 2H), 2.28 – 1.78 (m, 8H), 1.37 – 1.21 (m, 3H). ^{13}C NMR δ 173.77, 172.84, 172.44, 157.91, 133.62, 129.43, 113.85, 59.73, 55.31, 47.39, 46.61, 35.63, 34.36, 27.81, 27.36, 25.12, 18.00.

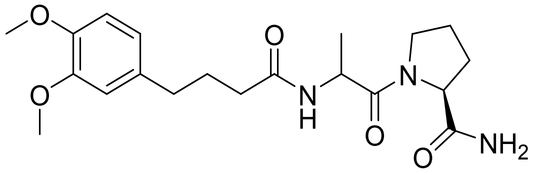

(2*S*)-1-((4-(3,4-Dimethoxyphenyl)butanoyl)alanyl)pyrrolidine-2-carboxamide (50). Synthesized according to method F using 4-(3,4-dimethoxyphenyl)butyric acid (0.41 g, 1.85 mmol). The crude product was obtained as a white foam, which after flash chromatography (EtOAc/MeOH 19:1 → 3:2) yielded compound **50** as a white foam (0.51 g, 71 %). ^{1}H NMR δ 7.61 (s, 0.15H), 6.83 – 6.75 (m, 1H), 6.75 – 6.68 (m, 2H), 6.63 (s, 0.85H), 6.38 (d, *J* = 7.5 Hz, 0.85H), 6.23 (d, *J* = 6.0 Hz, 0.15H), 5.78 (s, 0.15H), 5.54 (s, 0.85H), 4.76 (p, *J* = 7.0 Hz, 0.85H), 4.57 (dd, *J* = 8.1, 2.9 Hz, 0.85H), 4.35 – 4.28 (m, 0.3H), 3.95 – 3.77 (m, 6H), 3.75 – 3.64 (m, 1H), 3.64 – 3.51 (m, 1H), 2.64 – 2.54 (m, 2H), 2.41 – 2.30 (m, 1H), 2.26 – 2.18 (m, 2H), 2.18 – 1.84 (m, 5H), 1.35 (d, *J* = 6.9 Hz, 2.55H), 1.31 (d, *J* = 7.0 Hz, 0.45H) (two rotamers 17:3). ^{13}C NMR δ 173.03, 172.84, 172.17, 148.87, 147.30, 134.09, 120.32, 111.77, 111.24, 59.51, 55.95, 55.84, 47.31, 46.53, 35.70, 34.83, 27.24, 27.13, 25.07, 18.31 (minor rotamer also visible).

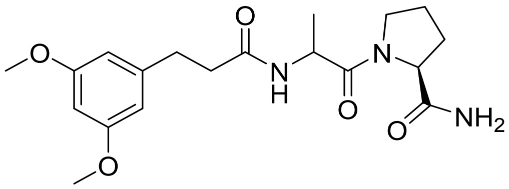

(2*S*)-1-((3-(3,5-Dimethoxyphenyl)propanoyl)alanyl)pyrrolidine-2-carboxamide (51). Synthesized according to method F using 4-(3,5-dimethoxyphenyl)propionic acid (0.40 g, 1.90 mmol). The crude product was obtained as a pale yellow sap, which after flash chromatography (EtOAc/MeOH 19:1 → 3:2) yielded compound **51** (0.43 g, 60 %). ^{1}H NMR δ 6.63 (s, 1H), 6.38 (d, *J* = 7.7 Hz, 1H), 6.36 – 6.34 (m, 2H), 6.32 – 6.29 (m, 1H), 5.48 (s, 1H), 4.76 (p, *J* = 7.0 Hz, 1H), 4.57 (dd, *J* = 8.1, 2.9 Hz, 1H), 3.77 (s, 6H), 3.73 – 3.63 (m, 1H), 3.63 – 3.52 (m, 1H), 2.95 – 2.82 (m, 2H), 2.56 – 2.42 (m, 2H), 2.42 – 2.31 (m, 1H), 2.21 – 1.88 (m, 3H), 1.32 (d, *J* = 6.9 Hz, 3H). ^{13}C NMR δ 172.97, 172.76, 171.38, 160.89, 143.09, 106.38, 98.22, 59.50, 55.28, 47.31, 46.57, 38.03, 31.86, 27.08, 25.06, 18.27.

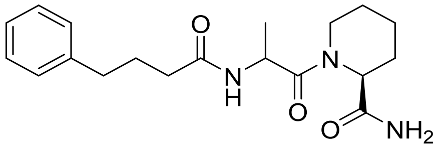

(2*S*)-1-((4-Phenylbutanoyl)alanyl)piperidine-2-carboxamide (35). Compound **103** (1.1 g, 3.0 mmol) was demethylated according to method D with a reaction time of 16 h. The crude product was obtained, which was used without further purification. Ethyl chloroformate (0.22 ml, 2.37 mmol) and ammonia (7 M, 1.7 ml, 11.8 mmol) were added to a solution of the intermediate (0.82 g, 2.37 mmol) and Et_3N (0.35 ml, 2.37 mmol) in anhydrous THF (15 ml) at -20 °C. The reaction was stirred at -20 °C for 10 min then raised to stir at room temperature overnight. THF was evaporated and the resulting residue was dissolved in EtOAc. The organic phase was washed with a 20 % aqueous solution of citric acid, a saturated solution of $NaHCO_3$, and brine, dried over anhydrous Na_2SO_4, filtered, and evaporated to provide the crude product, which after flash chromatography (EtOAc/MeOH 19:1) yielded compound **35** (393 mg, 50 %). ^{1}H NMR δ 7.33 – 7.23 (m, 2H), 7.23 – 7.13 (m, 3H), 6.72 – 6.53 (m, 2H), 5.67 (s, 1H), 5.26 (d, *J* = 5.4 Hz, 1H), 4.76 (p, *J* = 6.8 Hz, 1H), 3.90 – 3.77 (m, 1H), 3.30 – 3.14 (m, 1H), 2.72 – 2.59 (m, 2H), 2.28 – 2.15 (m, 2H), 2.01 – 1.86 (m, 2H), 1.82 – 1.18 (m, 9H). ^{13}C NMR δ 173.54, 172.87, 172.40, 141.39, 128.49, 128.41, 126.00, 60.41, 52.67, 45.61, 43.79, 35.36, 35.15, 27.01, 25.68, 20.55, 17.54.

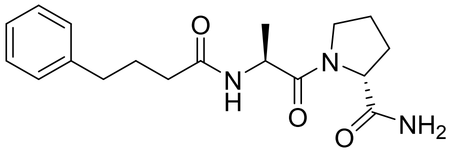

(*R*)-1-((4-Phenylbutanoyl)-L-alanyl)pyrrolidine-2-carboxamide (34).** Ethyl chloroformate (0.65 ml, 6.8 mmol) was added to a solution of compound **100** (1.89 g, 5.7 mmol) and Et_3N (0.95 ml, 6.8 mmol) in anhydrous THF (38 ml) at -10 °C. The mixture was stirred at -10 °C for 30 min. Ammonia (7 M, 4.1 ml, 29 mmol) was added and stirring was continued at room temperature for 3 h. MeOH and THF were evaporated and the resulting residue was dissolved in DCM. The organic phase was washed with a 20 % aqueous solution of citric acid, a saturated solution of $NaHCO_3$, and brine, dried over anhydrous Na_2SO_4, filtered, and evaporated to provide the crude product as a white foam, which after flash chromatography (EtOAc → EtOAc/MeOH 4:1) yielded compound **34** as a colourless sap (850 mg, 45 %). ^{1}H NMR δ 7.33 – 7.23 (m, 2H), 7.23 – 7.13 (m, 3H), 7.12 – 6.89 (m, 1H), 6.81 – 6.60 (m, 1H), 5.81 – 5.60 (m, 1H), 4.64 – 4.47 (m, 2H), 3.77 – 3.42 (m, 2H), 2.66 – 2.57 (m, 2H), 2.24 – 2.16 (m, 2H), 2.15 – 1.79 (m, 6H), 1.36 – 1.31 (m, 3H). ^{13}C NMR δ 173.93, 173.76, 172.51, 141.52, 128.58, 128.50, 126.08, 60.50, 47.64, 47.15, 35.35, 35.22, 27.10, 24.46, 21.15, 16.66.

Synthesis of untested final compounds

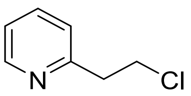

2-(2-Chloroethyl)pyridine (104). $SOCl_2$ (4.9 ml, 67 mmol) was added to a solution of 2-pyridineethanol (5.0 ml, 44 mmol) in anhydrous DCM (20 ml). The flask was covered with a $CaCl_2$ drying tube and the mixture was stirred at room temperature for 1 d. The mixture was poured into cold water and basified with KOH (50 % in cold water). The phases were separated and the aqueous phase extracted with DCM. The combined organic phase was dried over anhydrous Na_2SO_4, filtered, and evaporated to provide the crude product as a brown oil (quantitative), which was used without further purification. ^{1}H NMR δ 8.63 – 8.50 (m, 1H), 7.71 – 7.55 (m, 1H), 7.25 – 7.08 (m, 2H), 3.93 (td, *J* = 7.0, 1.2 Hz, 2H), 3.23 (td, *J* = 6.9, 1.3 Hz, 2H). ^{13}C NMR δ 157.99, 149.68, 136.56, 123.85, 122.00, 43.70, 41.16.

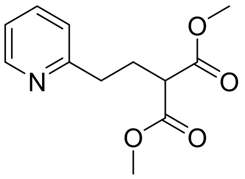

Dimethyl 2-(2-(pyridin-2-yl)ethyl)malonate (105). Diethyl malonate (6.7 ml, 44 mmol) was added to a suspension of NaH (60 % in mineral oil, 1.8 g, 44 mmol) and KI (0.37 g, 2.2 mmol) in anhydrous DMF (50 ml) at 0 °C. The mixture was left to stir at 0 °C for 30 min, before adding a solution of compound **104** (6.3 g, 44 mmol) in anhydrous DMF (15 ml). The mixture was heated to 80 °C and left to stir for 2 d, before cooling to room temperature and pouring into a pH 9.2 aqueous buffer solution ($NaHCO_3$ + Na_2CO_3). The aqueous phase was extracted with DCM and the organic phase was dried over anhydrous Na_2SO_4, filtered, and evaporated to provide the crude product as a brown oil, which after flash chromatography (heptane/EtOAc 2:3 → EtOAc) yielded compound **105** as an orange oil (6.5 g, 55 %). ^{1}H NMR δ 8.59 – 8.47 (m, 1H), 7.60 (td, *J* = 7.6, 1.8 Hz, 1H), 7.22 – 7.06 (m, 2H), 4.20 (qd, *J* = 7.1, 1.4 Hz, 4H), 3.40 (t, *J* = 7.5 Hz, 1H), 2.93 – 2.76 (m, 2H), 2.44 – 2.27 (m, 2H), 1.27 (t, *J* = 7.1 Hz, 7H). ^{13}C NMR δ 169.41, 160.56, 149.47, 136.59, 123.11, 121.48, 61.51, 51.51, 35.71, 28.53, 14.21.

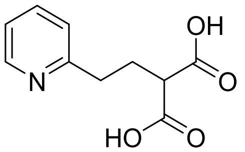

2-(2-(Pyridin-2-yl)ethyl)malonic acid (106). KOH (6.9 g, 123 mmol) was added gradually to a solution of compound **105** (6.5 g, 25 mmol) in H_2O (11 ml) and EtOH (11 ml). The mixture was refluxed for 3 h, before adjusting the pH to 4 and evaporating the solvents. The residue was dissolved in MeOH, filtered through silica, and evaporated to obtain the crude product as an off white foam (4.0 g, 78 %), which was used without further purification. ^1H NMR (Methanol-d_4) δ 8.41 (ddd, J = 5.0, 1.8, 0.9 Hz, 1H), 7.75 (td, J = 7.7, 1.8 Hz, 1H), 7.34 (dt, J = 7.9, 1.1 Hz, 1H), 7.24 (ddd, J = 7.5, 5.0, 1.2 Hz, 1H), 2.92 – 2.69 (m, 2H), 2.43 – 2.16 (m, 2H). ^{13}C NMR (Methanol-d_4) δ 176.95, 162.37, 149.48, 138.79, 124.72, 122.90, 36.35, 31.65.

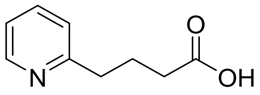

4-(pyridin-2-yl)butanoic acid (107). Compound **106** (4.0 g, 19 mmol) was heated to 150 °C for 3 h to obtain the crude product, which after flash chromatography (EtOAc/MeOH 9:1 → 2:3) yielded compound **107** as a brown solid (1.9 g, 61 %). ^1H NMR (Methanol-d_4) δ 8.43 (ddd, J = 5.0, 1.8, 0.9 Hz, 1H), 7.77 (td, J = 7.7, 1.8 Hz, 1H), 7.33 (dt, J = 7.9, 1.1 Hz, 1H), 7.26 (ddd, J = 7.6, 5.0, 1.2 Hz, 1H), 2.86 – 2.78 (m, 2H), 2.32 (t, J = 7.4 Hz, 2H), 2.05 – 1.94 (m, 2H). ^{13}C NMR (Methanol-d_4) δ 177.33, 162.45, 149.52, 138.86, 124.75, 122.95, 37.88, 34.52, 26.43.

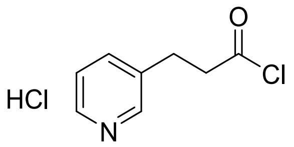

3-(Pyridin-3-yl)propanoyl chloride (108). Synthesized according to method A using 3-pyridinepropionic acid (1.2 g, 7.9 mmol). The crude product was obtained (quantitative), which was used without further purification.

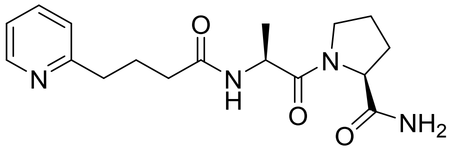

(*S*)-1-((4-(Pyridin-2-yl)butanoyl)-L-alanyl)pyrrolidine-2-carboxamide (109). Compound **107** (0.20 g, 1.2 mmol), compound **81** (0.40 g, 1.3 mmol), HATU (0.60 g, 1.6 mmol), and DIPEA (0.63 ml, 3.6 mmol) were dissolved in anhydrous DMF (7.5 ml). The mixture was left to stir at room temperature for 4 h, then diluted with H_2O, basified with NaOH, saturated with NaCl, and extracted with EtOAc. The organic phase was dried over anhydrous Na_2SO_4, filtered, and evaporated to provide the crude product as an orange oil (quantitative), which was used without further purification. ^1H NMR δ 8.59 – 8.50 (m, 1H), 7.87 (s, 0.2H), 7.73 – 7.64 (m, 1H), 7.54 (s, 0.2H), 7.26 – 7.21 (m, 1H), 7.21 – 7.15 (m, 1H), 7.03 (s, 0.8H), 6.81 (s, 0.8H), 5.94 (s, 0.2H), 5.61 (s, 0.8H), 4.76 (p, J = 7.0 Hz, 0.8H), 4.59 (dd, J = 8.1, 3.0 Hz, 0.8H), 4.40 – 4.28 (m, 0.4H), 3.77 – 3.64 (m, 2H), 3.16 – 3.06 (m, 2H), 2.35 – 2.24 (m, 3H), 2.17 – 1.89 (m, 5H), 1.39 – 1.34 (m, 3H) (two rotamers 4:1). ^{13}C NMR δ 173.44, 173.00, 172.40, 160.99, 148.40, 137.63, 123.60, 121.70, 59.65, 47.44, 46.81, 42.01, 36.64, 35.47, 25.83, 25.17, 18.12 (minor rotamer also visible).

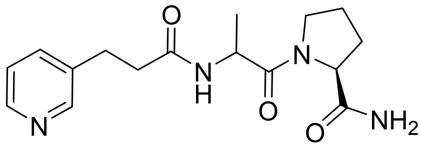

3-(Pyridin-3-yl)propanoyl-D/L-Alanyl-L-prolinamide (110). Et_3N (2.8 ml, 20 mmol) was added to a solution of compound **81** (0.78 g, 2.6 mmol) in anhydrous DCM (20 ml) at 0 °C. The mixture was added to a solution of compound **108** (0.80 g, 4 mmol) in anhydrous DCM (15 ml) at -20 °C. The mixture was allowed to warm to 0 °C over one hour, then left to stir at 0 °C for 3 d. The solvent was evaporated to obtain the crude product, which after flash chromatography (EtOAc/MeOH/Et_3N 79.5:20:0.5 → 59.5:40:0.5) yielded compound **110** (quantitative). Et_3N could not be fully removed from the product by evaporation. ^1H NMR δ 8.58 – 8.34 (m, 2H), 7.61 – 7.53 (m, 1H), 7.49 (d, *J* = 5.8 Hz, 0.6H), 7.27 – 7.20 (m, 1H), 7.04 (s, 0.6H), 6.93 (d, *J* = 7.3 Hz, 0.4H), 6.85 (s, 0.4H), 5.88 (s, 0.4H), 5.70 (s, 0.6H), 4.71 (p, *J* = 7.0 Hz, 0.4H), 4.59 – 4.46 (m, 1.6H), 3.94 – 3.83 (m, 0.6H), 3.77 – 3.53 (m, 1.4H), 3.02 – 2.89 (m, 2H), 2.63 – 2.50 (m, 2H), 2.31 – 1.96 (m, 4H), 1.33 – 1.29 (m, 3H) (two rotamers 3:2). ^{13}C NMR δ 174.09, 173.53, 172.35, 172.34, 172.16, 170.98, 162.46, 162.12, 149.59, 149.54, 147.34, 147.32, 136.34, 136.31, 123.50, 123.49, 60.38, 59.69, 47.64, 47.28, 47.06, 46.78, 37.18, 36.67, 29.06, 28.53, 28.50, 27.88, 25.02, 24.39, 17.84, 16.41 (second set of signals (40 %) from minor rotamer).

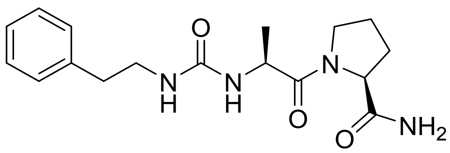

Method G: Synthesis of (*S*)-1-((Phenethylcarbamoyl)-L-alanyl)pyrrolidine-2-carboxamide (111). Phenethyl isocyanate (0.52 ml, 3.7 mmol) was added dropwise to a solution of compound **81** (1.0 g, 3.4 mmol) and Et3N (1.5 ml, 11 mmol) in anhydrous DCM (12 ml). The mixture was left to stir at room temperature for 3 h. The solvent was removed by evaporation to provide a crude product, which after flash chromatography (EtOAc/MeOH 19:1 → 4:1) yielded compound **111** as a yellow sap (0.94 g, 83 %). ^1H NMR (Methanol-d_4) δ 7.30 – 7.23 (m, 2H), 7.23 – 7.14 (m, 3H), 4.48 – 4.35 (m, 1H), 3.96 – 3.86 (m, 1H), 3.67 – 3.44 (m, 2H), 3.37 – 3.26 (m, 2H), 2.74 (t, *J* = 7.2 Hz, 2H), 2.38 – 1.86 (m, 4H), 1.30 – 1.23 (m, 3H) (minor rotamer also visible). ^{13}C NMR (Methanol-d_4) δ 177.28, 175.08, 160.51, 140.71, 129.88, 129.47, 127.25, 61.88, 49.03, 48.29, 42.56, 37.43, 30.75, 25.37, 17.07 (minor rotamer also visible).

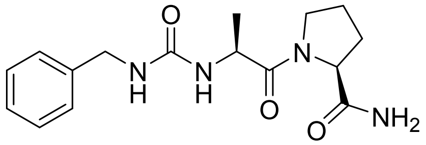

(*S*)-1-((Benzylcarbamoyl)-L-alanyl)pyrrolidine-2-carboxamide (112). Synthesized according to method G using benzyl isocyanate (0.71 ml, 5.8 mmol). The crude product was obtained, which after flash chromatography (EtOAc/MeOH 19:1 → 4:1) yielded compound **112** as a white foam (quantitative). ^1H NMR (Methanol-d_4) δ 7.35 – 7.16 (m, 5H), 4.48 (q, *J* = 6.9 Hz, 1H), 4.38 (dd, *J* = 8.7, 3.1 Hz, 1H), 4.29 (s, 2H), 3.66 – 3.44 (m, 2H), 2.38 – 1.83 (m, 4H), 1.33 – 1.29 (m, 3H). ^{13}C NMR (Methanol-d_4) δ 177.24, 175.07, 169.57, 141.11, 129.48, 128.21, 128.01, 61.53, 49.14, 48.30, 44.67, 30.75, 25.35, 17.12.

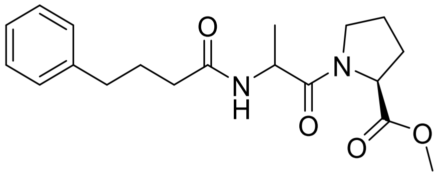

Methyl (4-phenylbutanoyl)alanyl-L-prolinate (113). Synthesized according to method E using compound **82** (1.0 g, 4.3 mmol) with ethyl chloroformate (0.41 ml, 4.3 mmol) replacing pivaloyl chloride and L-proline methyl ester hydrochloride (0.77 g, 4.7 mmol) replacing L-prolinamide. The crude product was obtained as an orange, which after flash chromatography (EtOAc/MeOH 49:1 → 9:1) yielded compound **113** as a

colourless oil (1.08 g, 73 %). ^{1}H NMR δ 7.32 – 7.22 (m, 2H), 7.22 – 7.12 (m, 3H), 6.47 (d, *J* = 7.7 Hz, 0.75H), 6.22 (d, *J* = 8.0 Hz, 0.25H), 4.83 – 4.72 (m, 1H), 4.54 – 4.49 (m, 0.25H), 4.47 – 4.41 (m, 0.75H), 3.82 – 3.77 (m, 0.75H), 3.73 – 3.71 (m, 3H), 3.65 – 3.60 (m, 0.25H), 3.59 – 3.48 (m, 1H), 2.70 – 2.61 (m, 2H), 2.30 – 1.84 (m, 8H), 1.37 (d, *J* = 6.8 Hz, 0.75H), 1.33 (d, *J* = 6.7 Hz, 2.25H) (two rotamers 3:1). ^{13}C NMR δ 172.39, 172.37, 172.09, 171.88, 171.50, 171.36, 141.61, 141.57, 128.62, 128.59, 128.48, 128.47, 126.06, 126.02, 59.15, 58.87, 52.41, 52.37, 46.98, 46.95, 46.88, 46.65, 35.93, 35.90, 35.30, 35.23, 29.20, 29.04, 27.22, 27.04, 25.04, 24.74, 18.60, 18.22 (additional set of signals (ca. 25 %) from minor rotamer).

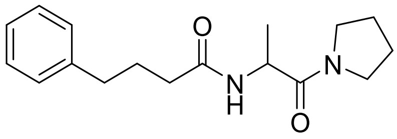

***N*-(1-Oxo-1-(pyrrolidin-1-yl)propan-2-yl)-4-phenylbutanamide (114).** Synthesized according to method E using compound **82** (2.2 g, 9.2 mmol) with pyrrolidine (0.91 ml, 11 mmol) replacing L-prolinamide. The crude product was obtained, which after flash chromatography (EtOAc/MeOH 99:1 → 4:1) yielded compound **114** (1.1 g, 41 %). ^{1}H NMR δ 7.34 – 7.23 (m, 2H), 7.23 – 7.13 (m, 3H), 6.54 (d, *J* = 7.6 Hz, 1H), 4.72 (p, *J* = 6.9 Hz, 1H), 3.61 (dt, *J* = 10.2, 6.6 Hz, 1H), 3.56 – 3.36 (m, 3H), 2.64 (t, *J* = 7.6 Hz, 2H), 2.24 – 2.15 (m, 2H), 2.02 – 1.83 (m, 6H), 1.32 (d, *J* = 6.8 Hz, 3H). ^{13}C NMR δ 171.97, 171.03, 141.63, 128.61, 128.47, 126.02, 46.81, 46.48, 46.15, 35.98, 35.35, 27.24, 26.15, 24.23, 18.58.

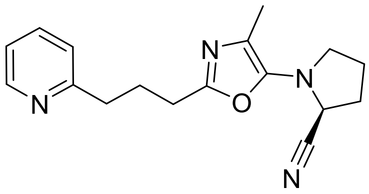

Method H: Synthesis of (*S*)-1-(4-methyl-2-(3-(pyridin-2-yl)propyl)oxazol-5-yl)pyrrolidine-2-carbonitrile (69).

TFAA (0.18 ml, 1.3 mmol) was added slowly to a solution of compound **109** (200 mg, 0.60 mmol) and Et_3N (0.37 ml, 2.7 mmol) in anhydrous THF (6 ml) at 0 °C. The mixture was left to stir at 0 °C for 2 h, before quenching with H_2O and removing THF by evaporation. The residue was diluted with EtOAc, washed with a 10% aqueous solution of citric acid, a saturated solution of $NaHCO_3$, and brine, dried over anhydrous Na_2SO_4, filtered, and evaporated to provide the crude product as a dark green oil, which after flash chromatography (EtOAc/MeOH 19:1 → 4:1) yielded compound **69** as a brown oil (16 mg, 9 %). ^{1}H NMR (Methanol-d_4) δ 8.43 (ddd, *J* = 5.0, 1.8, 0.9 Hz, 1H), 7.75 (td, *J* = 7.7, 1.8 Hz, 1H), 7.32 (dt, *J* = 7.9, 1.1 Hz, 1H), 7.24 (ddd, *J* = 7.6, 5.0, 1.2 Hz, 1H), 4.36 (dd, *J* = 8.0, 4.2 Hz, 1H), 3.40 (ddd, *J* = 8.9, 7.4, 5.3 Hz, 1H), 3.26 (dt, *J* = 8.9, 7.2 Hz, 1H), 2.88 – 2.81 (m, 2H), 2.71 (t, *J* = 7.4 Hz, 2H), 2.44 – 2.08 (m, 6H), 2.07 (s, 3H). ^{13}C NMR (Methanol-d_4) δ 162.13, 161.21, 149.67, 148.27, 138.76, 124.82, 124.47, 122.96, 121.00, 53.40, 51.95, 37.85, 32.31, 28.63, 28.01, 25.26, 10.80.

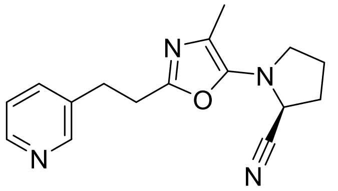

***N*-(4-Methyl-2-(2-(pyridin-3-yl)ethyl)oxazol-5-yl)-2(S)-cyanopyrrolidine (70).** Synthesized according to method H using compound **110** (0.83 g, 2.6 mmol). The crude product was obtained, which after two flash chromatographies (DCM/MeOH 9:1 and DCM/toluene/MeOH 92:3:5) yielded compound **70** as a yellow sap

(66 mg, 9 %). ^{1}H NMR δ 8.48-8.34 (m, 2H), 7.52 (d, J = 6 Hz, 1H), 7.21 (dd, J = 7.8, 4.8 Hz, 1H), 4.11 (dd, J = 7.6, 4.3 Hz, 1H), 3.41-3.34 (m, 1H), 3.23-3.15 (m, 1H), 3.12 – 2.99 (m, 2H), 2.99 – 2.89 (m, 2H), 2.39 – 2.17 (m, 2H), 2.17 – 1.97 (m, 5H). ^{13}C NMR δ 158.38, 149.90, 147.92, 146.41, 136.01, 135.88, 124.64, 123.57, 119.73, 52.47, 51.03, 31.40, 30.30, 30.07, 24.29, 11.18.

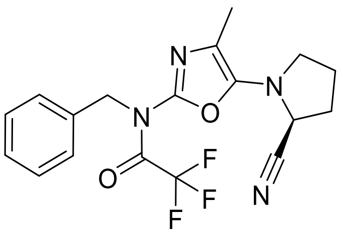

(*S*)-*N*-Benzyl-*N*-(5-(2-cyanopyrrolidin-1-yl)-4-methyloxazol-2-yl)-2,2,2-trifluoroacetamide (66). Synthesized according to method H using compound **112** (0.75 g, 2.4 mmol). The crude product was obtained as a yellow oil, which after flash chromatography (heptane/EtOAc 9:1 → 1:1) yielded compound **66** as a yellow oil (33 mg, 5 %). ^{1}H NMR δ 7.38 – 7.23 (m, 6H), 5.03 (d, *J* = 14.4 Hz, 1H), 4.95 (d, *J* = 14.4 Hz, 1H), 4.07 – 3.98 (m, 1H), 3.36 (ddd, *J* = 9.0, 7.8, 4.9 Hz, 1H), 3.14 (dt, *J* = 9.0, 7.3 Hz, 1H), 2.37 – 2.20 (m, 2H), 2.19 – 1.97 (m, 5H). ^{13}C NMR δ 156.87 (q, $^2J_{C,F}$ = 38.4 Hz), 146.52, 146.45, 134.30, 128.93, 128.87, 128.59, 126.07, 119.17, 115.81 (q, $^1J_{C,F}$ = 288.0 Hz), 53.24, 52.32, 51.04, 31.46, 24.40, 11.32.

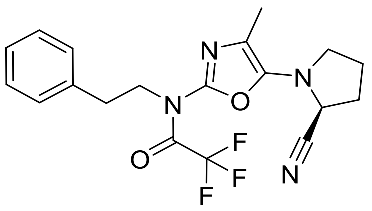

(*S*)-*N*-(5-(2-Cyanopyrrolidin-1-yl)-4-methyloxazol-2-yl)-2,2,2-trifluoro-*N*-phenethylacetamide (67). Synthesized according to method H using compound **111** (350 mg, 1.05 mmol). The crude product was obtained, which after flash chromatography (heptane/EtOAc 9:1 → 2:3) yielded compound **67** (42 mg, 13 %). ^{1}H NMR δ 7.32 – 7.25 (m, 2H), 7.25 – 7.17 (m, 3H), 4.15 – 4.01 (m, 3H), 3.40 (ddd, *J* = 9.0, 7.8, 4.9 Hz, 1H), 3.20 (dt, *J* = 9.0, 7.3 Hz, 1H), 3.04 – 2.95 (m, 2H), 2.40 – 2.24 (m, 2H), 2.15 (s, 3H), 2.24 – 2.02 (m, 2H). ^{13}C NMR δ 156.80 (q, $^2J_{C,F}$ = 38.5 Hz), 146.77, 146.21, 137.28, 129.05, 128.70, 126.91, 125.95, 119.31, 115.74 (q, $^1J_{C,F}$ = 288.2 Hz), 52.32, 51.11, 50.96, 34.00, 31.52, 24.48, 11.32.

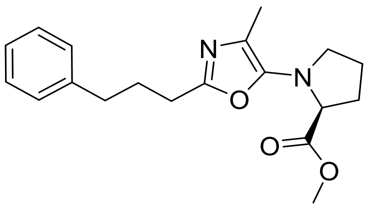

Method I: Synthesis of methyl (4-methyl-2-(3-phenylpropyl)oxazol-5-yl)-L-prolinate (68). Ph_3PBr_2 (0.84 g, 2.0 mmol) followed by Et_3N (0.70 ml, 5.0 mmol) were added to a solution of compound **113** (0.58 g, 1.7 mmol) in anhydrous DCM (8 ml). The mixture was stirred at reflux for 30 min, then at room temperature for 17 h. The solution was diluted with hexane and filtered. The filtrate was evaporated to provide the crude product as oily brown solids, which after flash chromatography (heptane/EtOAc 17:3 → 3:7) yielded compound **68** as a yellow oil (0.23 g, 42 %). ^{1}H NMR δ 7.32 – 7.22 (m, 2H), 7.22 – 7.13 (m, 3H), 4.05 (dd, *J* = 8.7, 4.3 Hz, 1H), 3.68 (s, 3H), 3.53 – 3.44 (m, 1H), 3.26 (dt, *J* = 8.6, 7.1 Hz, 1H), 2.65 (dt, *J* = 15.1, 7.6 Hz, 4H), 2.31 – 1.93 (m, 9H). ^{13}C NMR δ 174.31, 158.00, 148.31, 141.66, 128.64, 128.49, 126.04, 63.26, 52.15, 51.90, 35.35, 30.50, 28.69, 28.04, 24.82, 11.24.

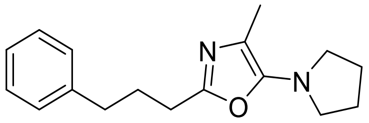

4-Methyl-2-(3-phenylpropyl)-5-(pyrrolidin-1-yl)oxazole (65). Synthesized according to method I using compound **114** (1.25 g, 4.3 mmol). The crude product was obtained as a brown oil, which after flash chromatography using an alumina column (heptane/EtOAc 9:1) yielded compound **65** (260 mg, 22 %). Degradation product also visible in NMR. ^{1}H NMR δ 7.30 – 7.24 (m, 2H), 7.22 – 7.16 (m, 3H), 3.24 – 3.17 (m, 4H), 2.71 – 2.65 (m, 2H), 2.65 – 2.59 (m, 2H), 2.09 (s, 3H), 2.08 – 2.00 (m, 2H), 1.93 – 1.87 (m, 4H). ^{13}C NMR δ 156.52, 150.06, 141.70, 128.58, 128.41, 125.94, 115.60, 50.77, 35.33, 28.75, 27.93, 25.18, 11.43.

UPLC-MS traces and NMR spectra

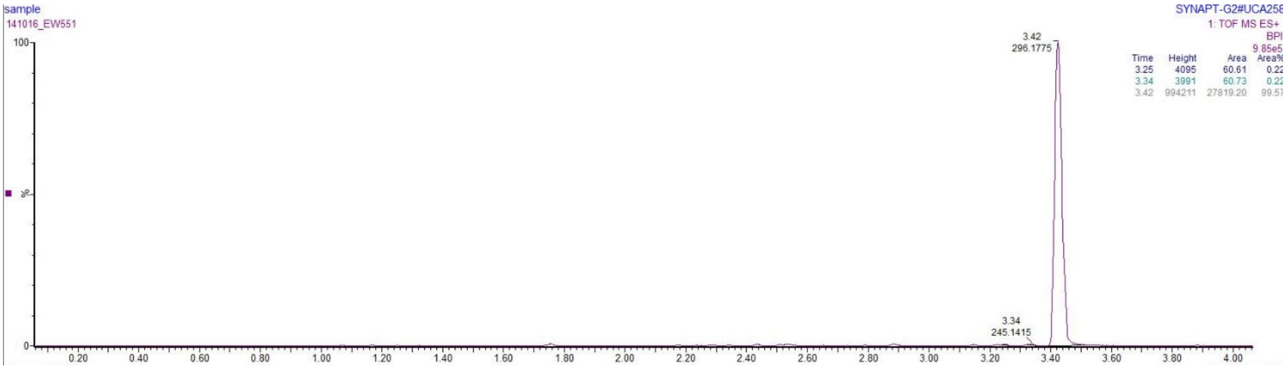

Figure S1. UPLC-MS trace from **HUP-55**.

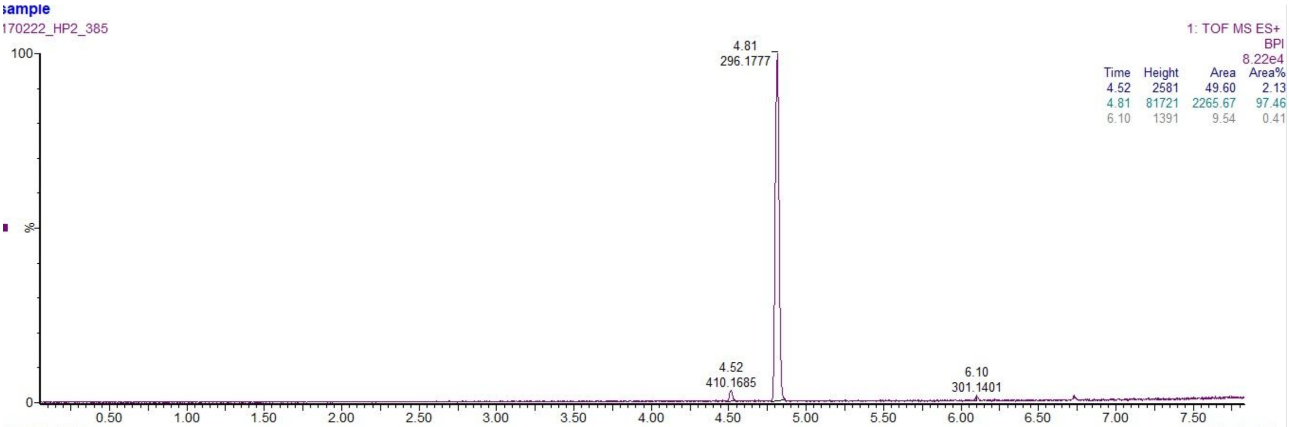

Figure S2. UPLC-MS trace from compound **3**.

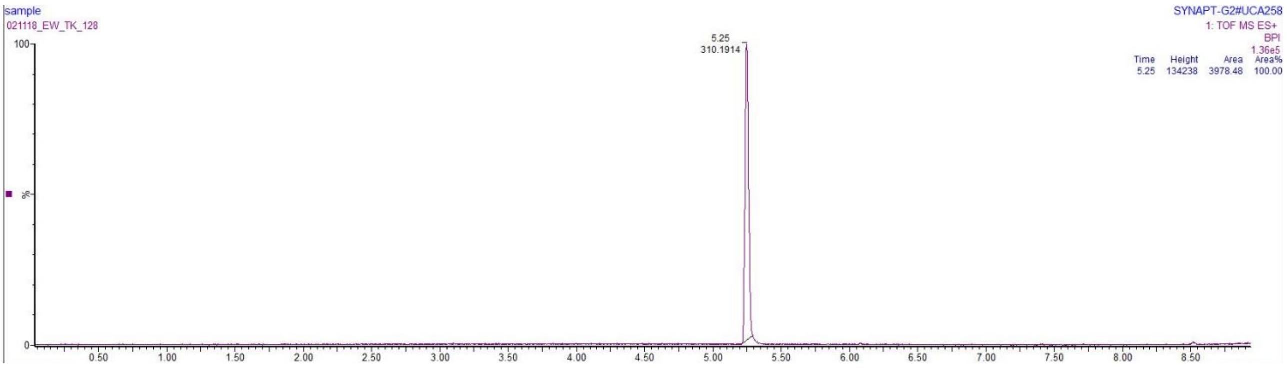

Figure S3. UPLC-MS trace from compound **4**.

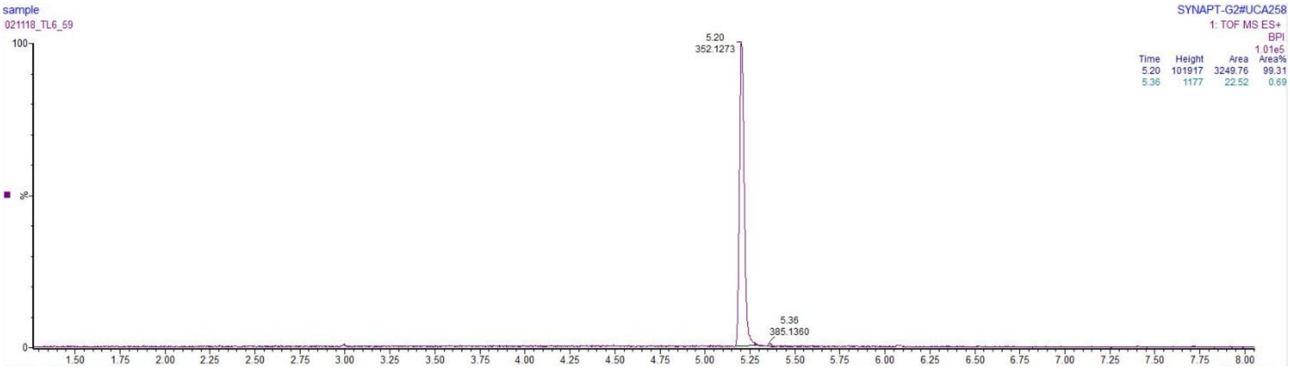

Figure S4. UPLC-MS trace from compound **5**.

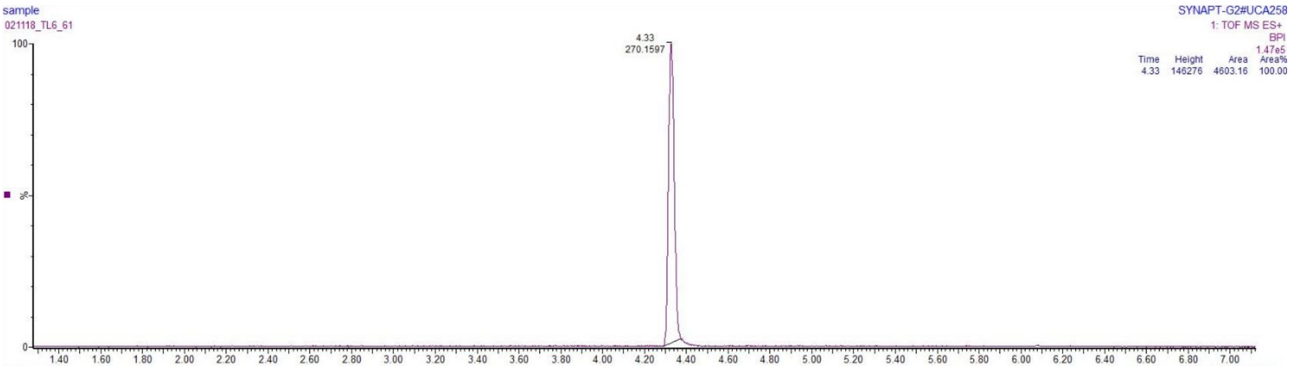

Figure S5. UPLC-MS trace from compound **6**.

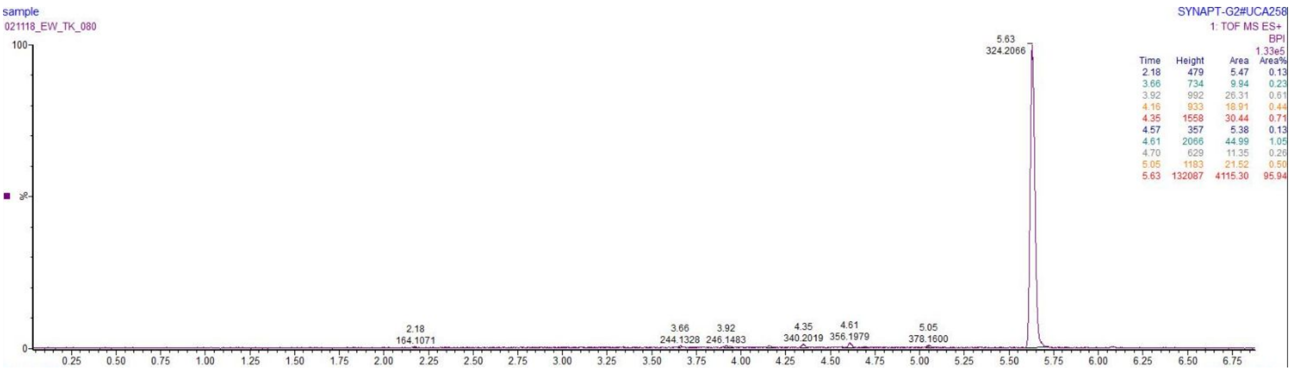

Figure S6. UPLC-MS trace from compound **7**.

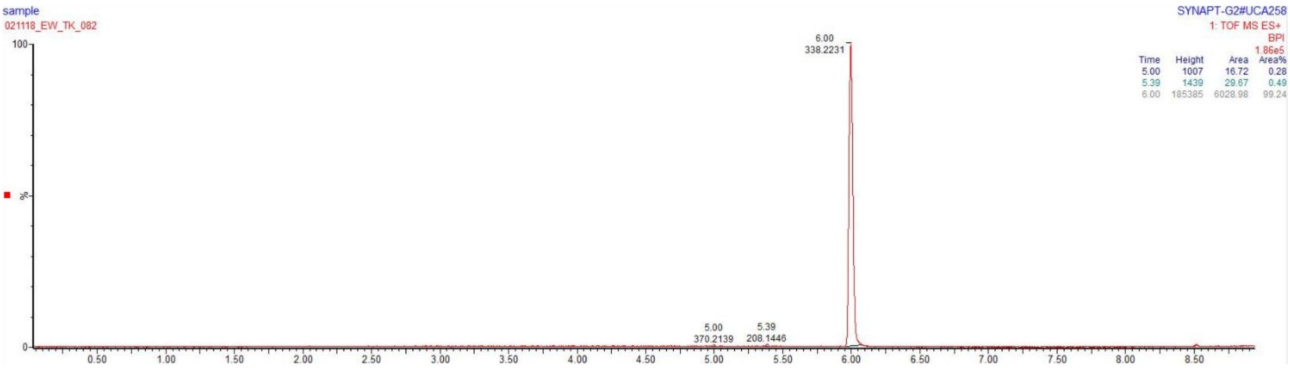

Figure S7. UPLC-MS trace from compound **8**.

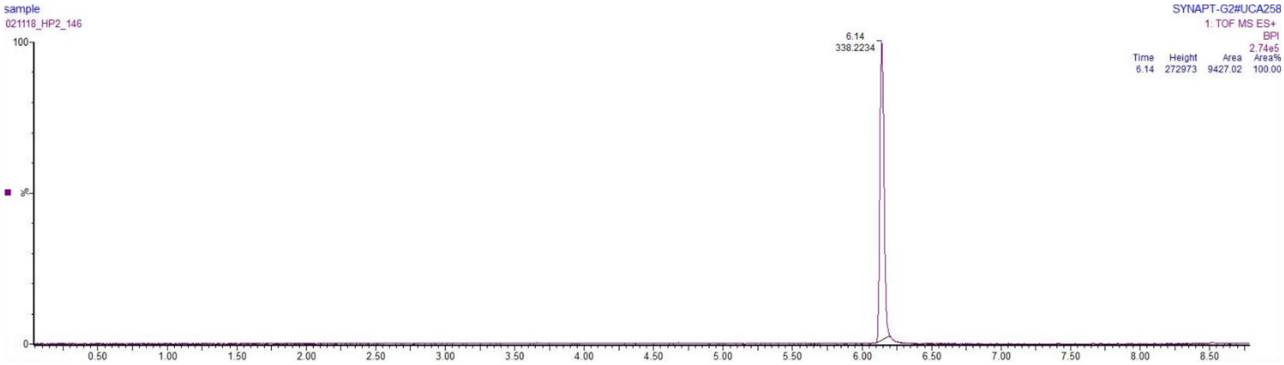

Figure S8. UPLC-MS trace from compound **9**.

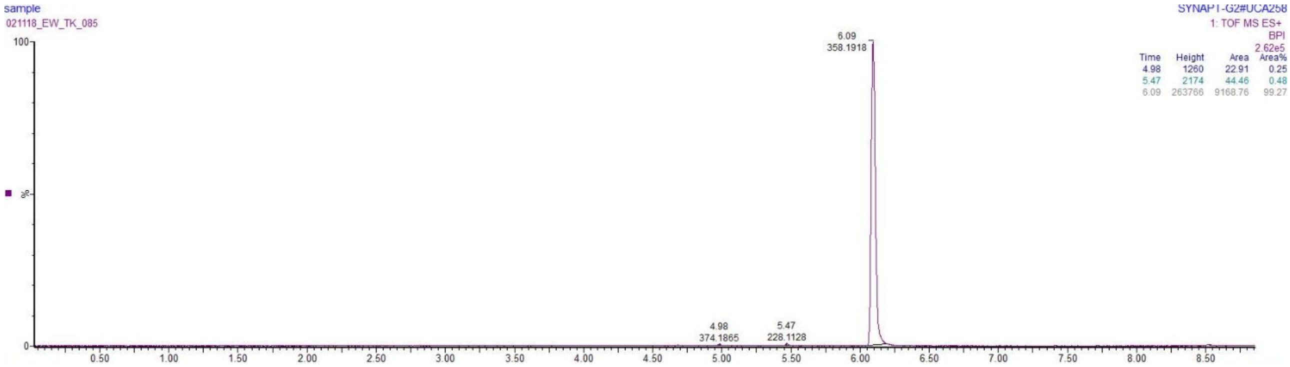

Figure S9. UPLC-MS trace from compound **10**.

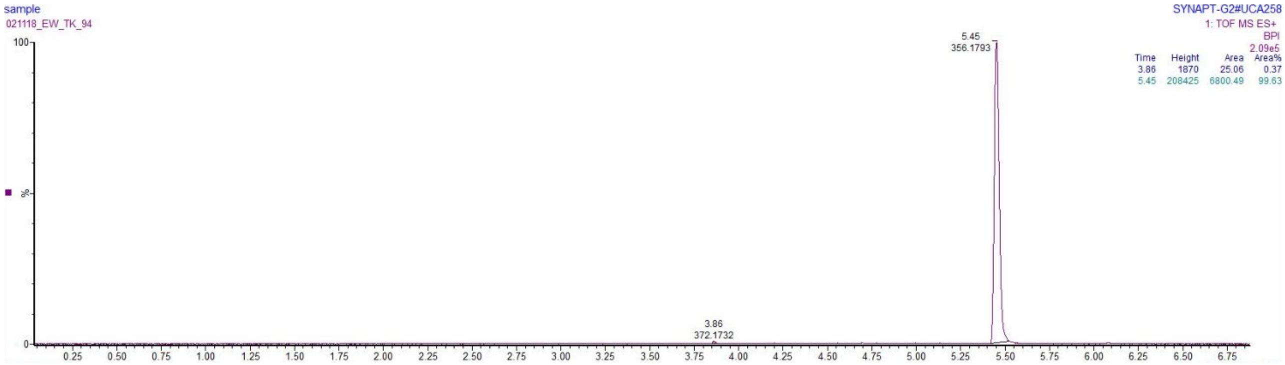

Figure S10. UPLC-MS trace from compound **11**.

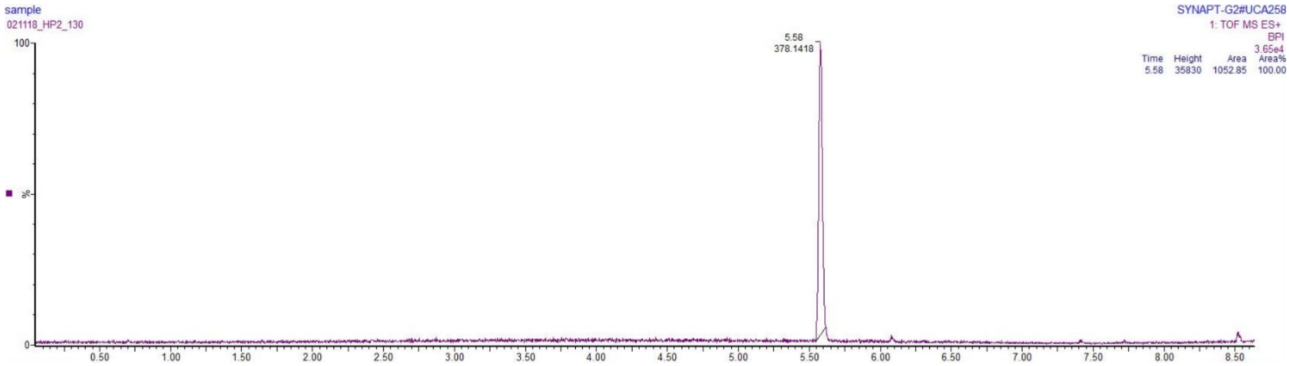

Figure S11. UPLC-MS trace from compound **12**.

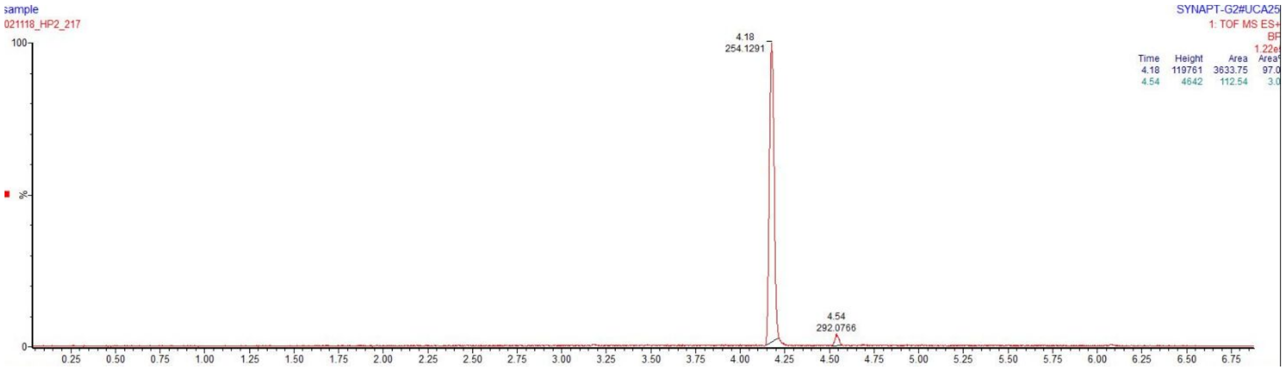

Figure S12. UPLC-MS trace from compound **13**.

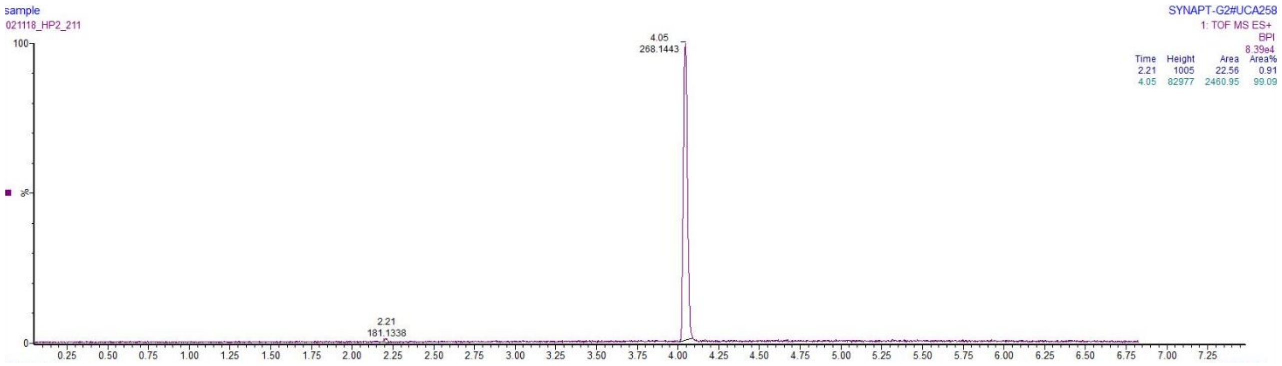

Figure S13. UPLC-MS trace from compound **14**.

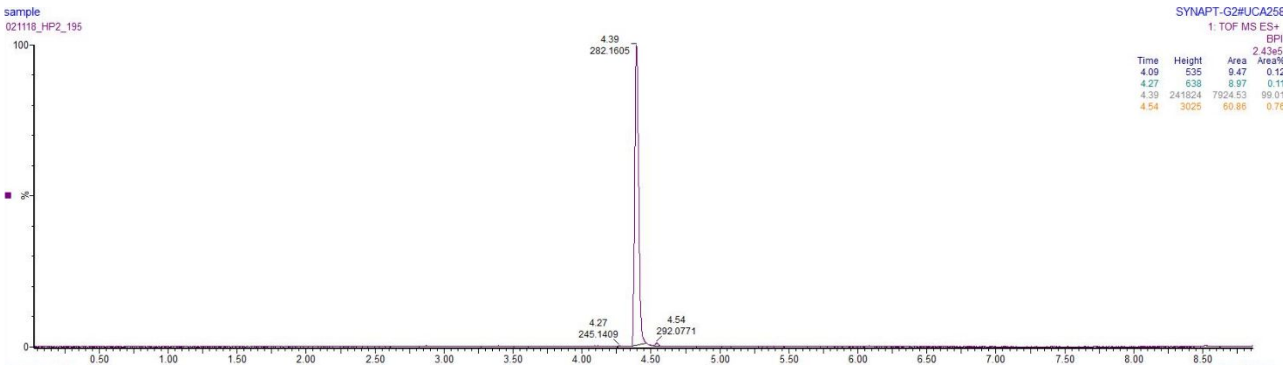

Figure S14. UPLC-MS trace from compound **15**.

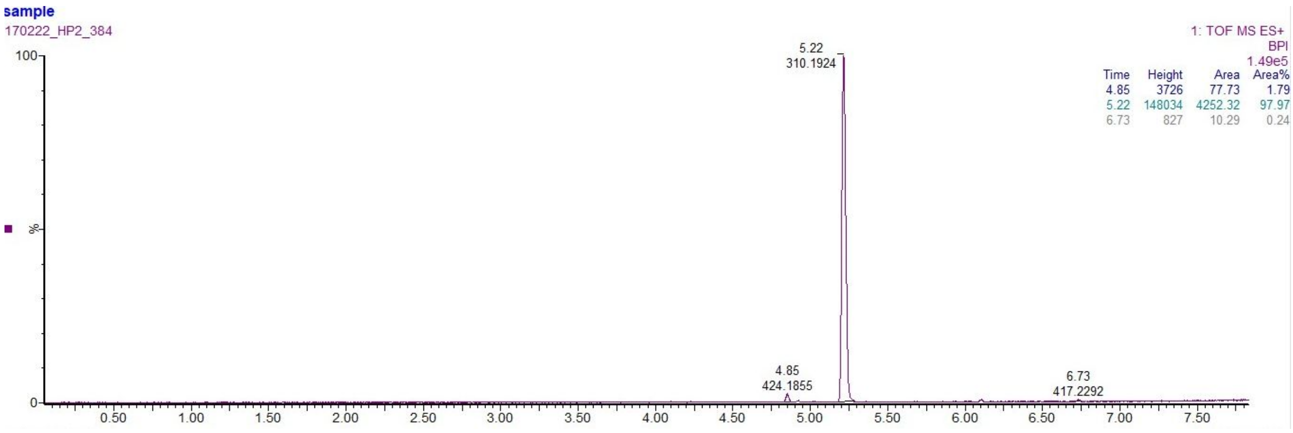

Figure S15. UPLC-MS trace from compound **16**.

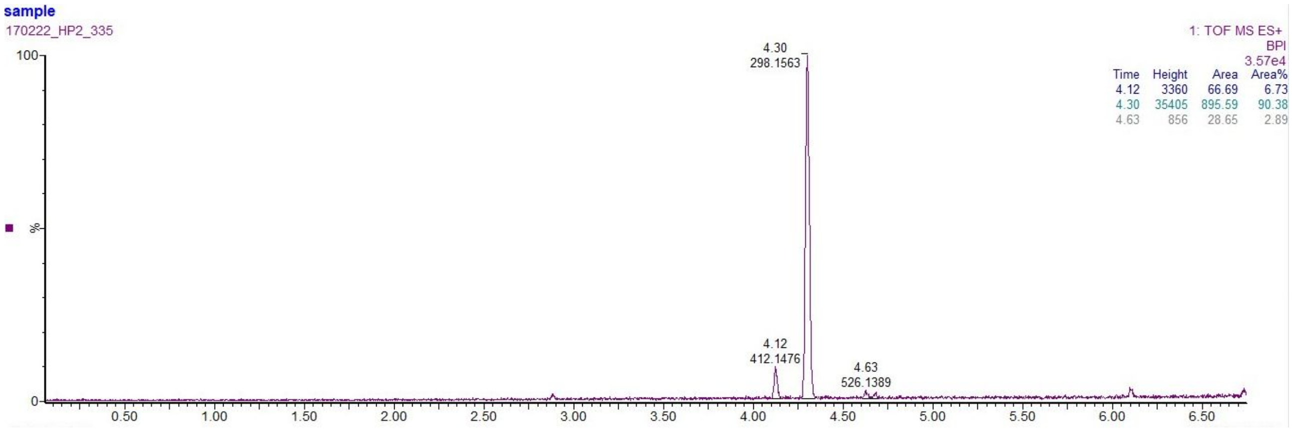

Figure S16. UPLC-MS trace from compound **17**.

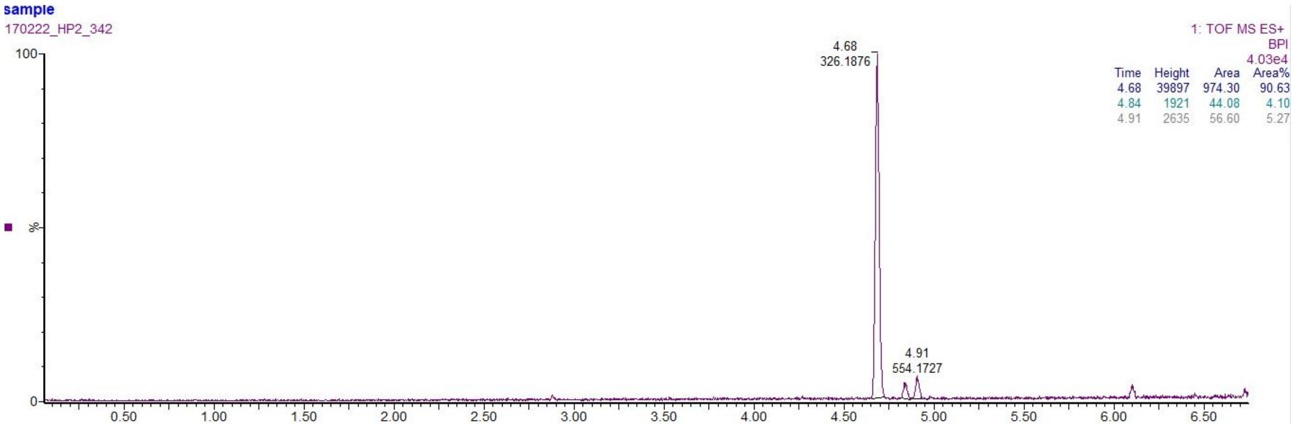

Figure S17. UPLC-MS trace from compound **18**.

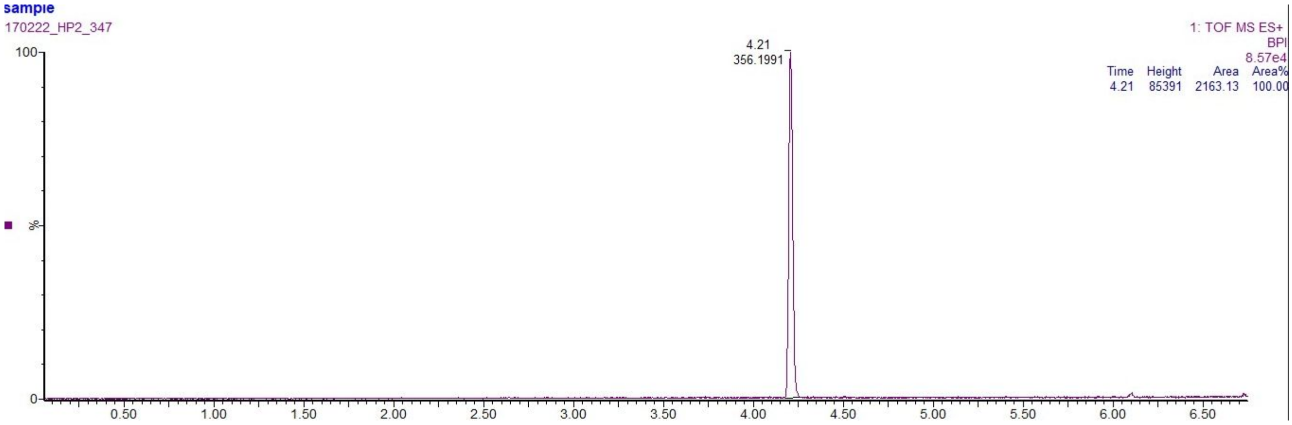

Figure S18. UPLC-MS trace from compound **19**.

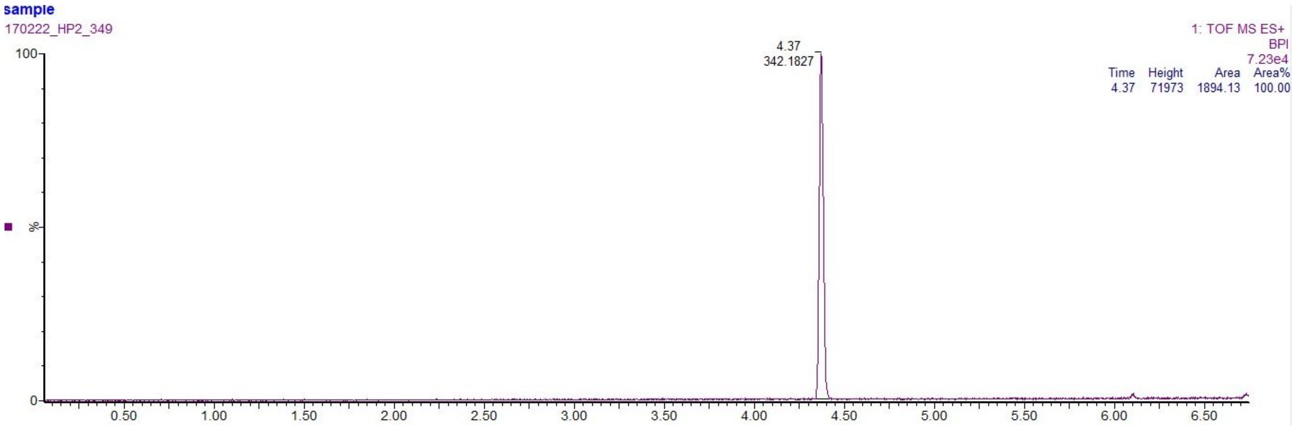

Figure S19. UPLC-MS trace from compound **20**.

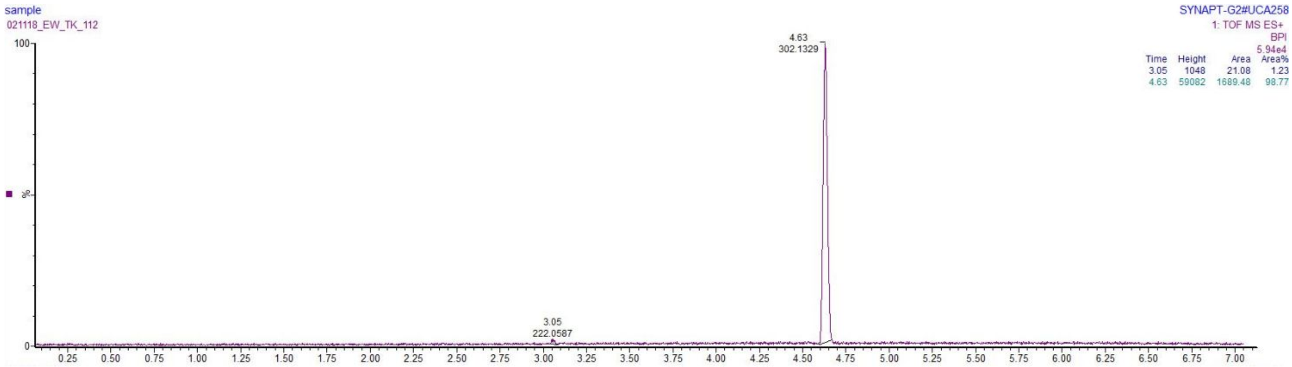

Figure S20. UPLC-MS trace from compound **21**.

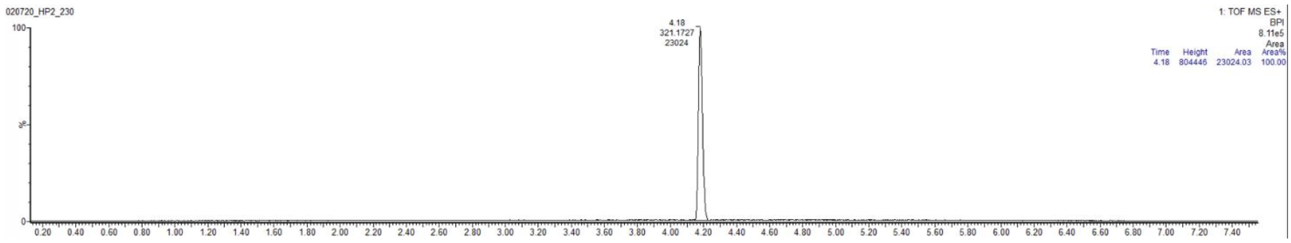

Figure S21. UPLC-MS trace from compound **22**.

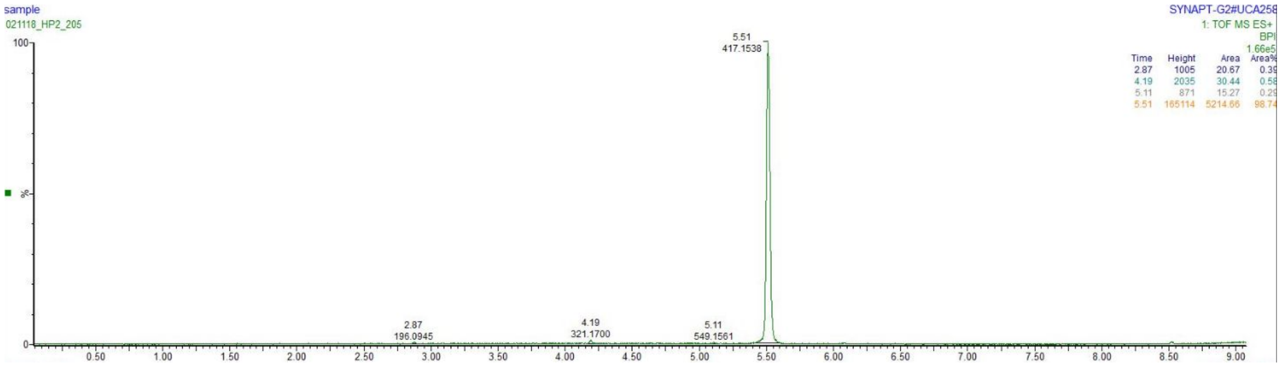

Figure S22. UPLC-MS trace from compound **23**.

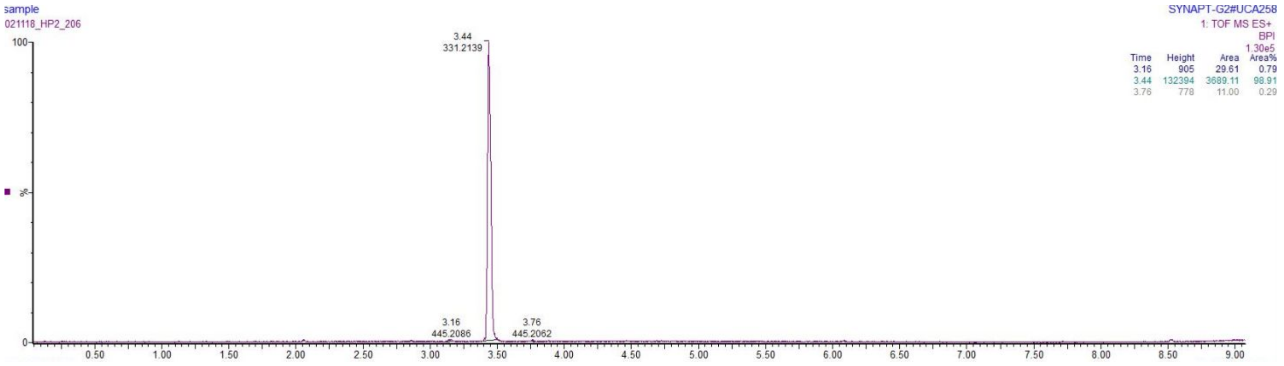

Figure S23. UPLC-MS trace from compound **24**.

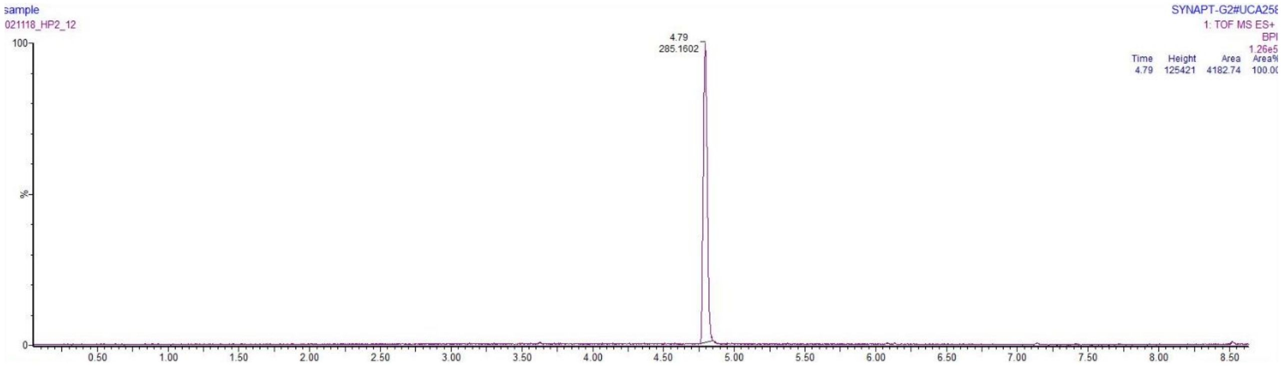

Figure S24. UPLC-MS trace from compound **25**.

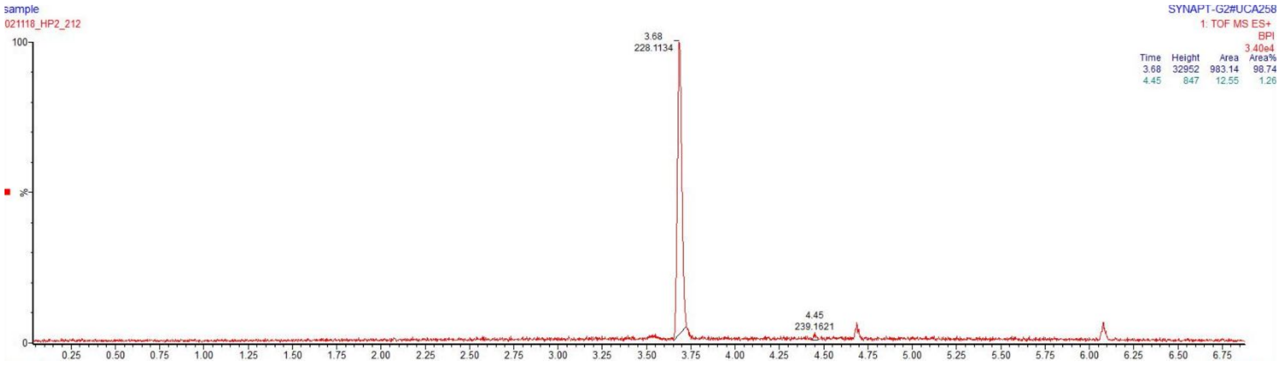

Figure S25. UPLC-MS trace from compound **26**.

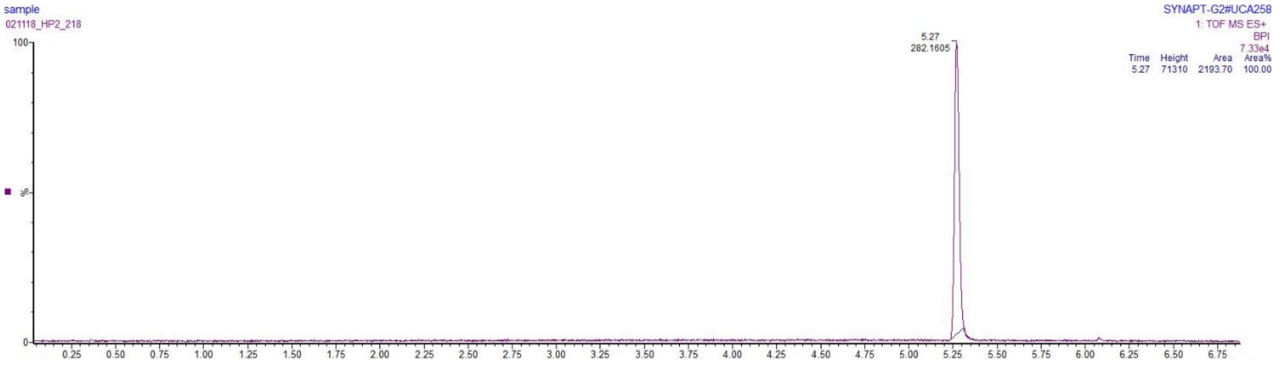

Figure S26. UPLC-MS trace from compound **27**.

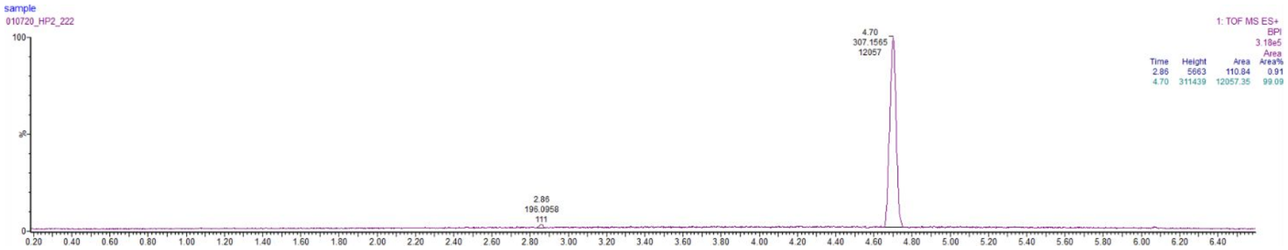

Figure S27. UPLC-MS trace from compound **28**.

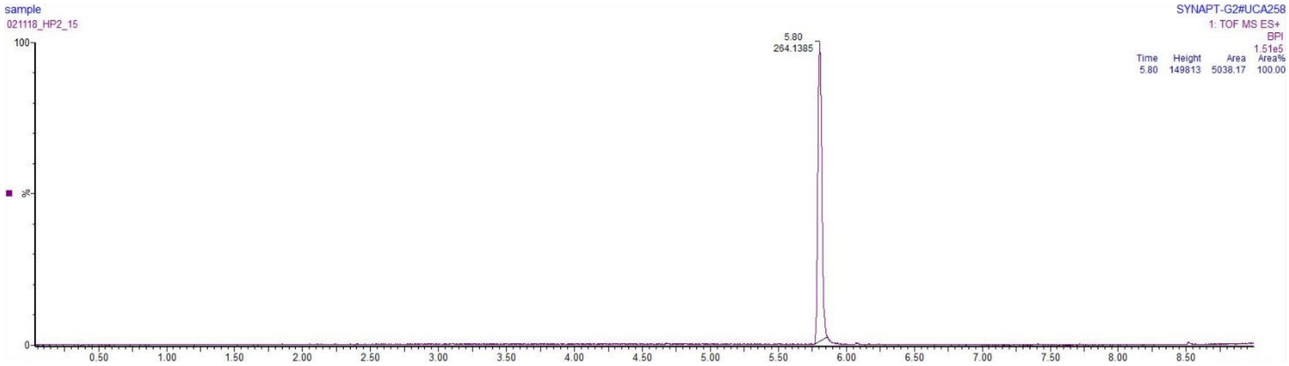

Figure S28. UPLC-MS trace from compound **29**.

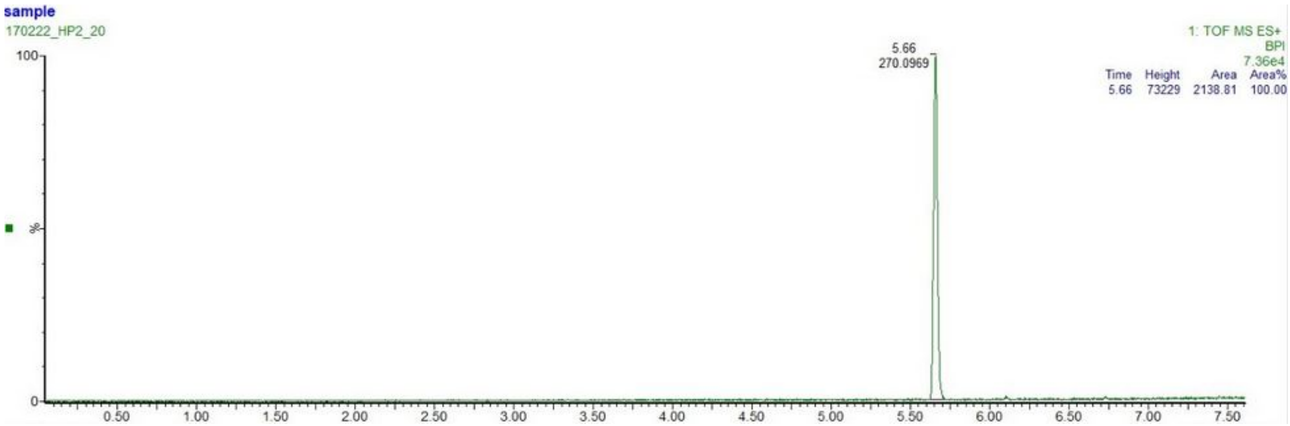

Figure S29. UPLC-MS trace from compound **30**.

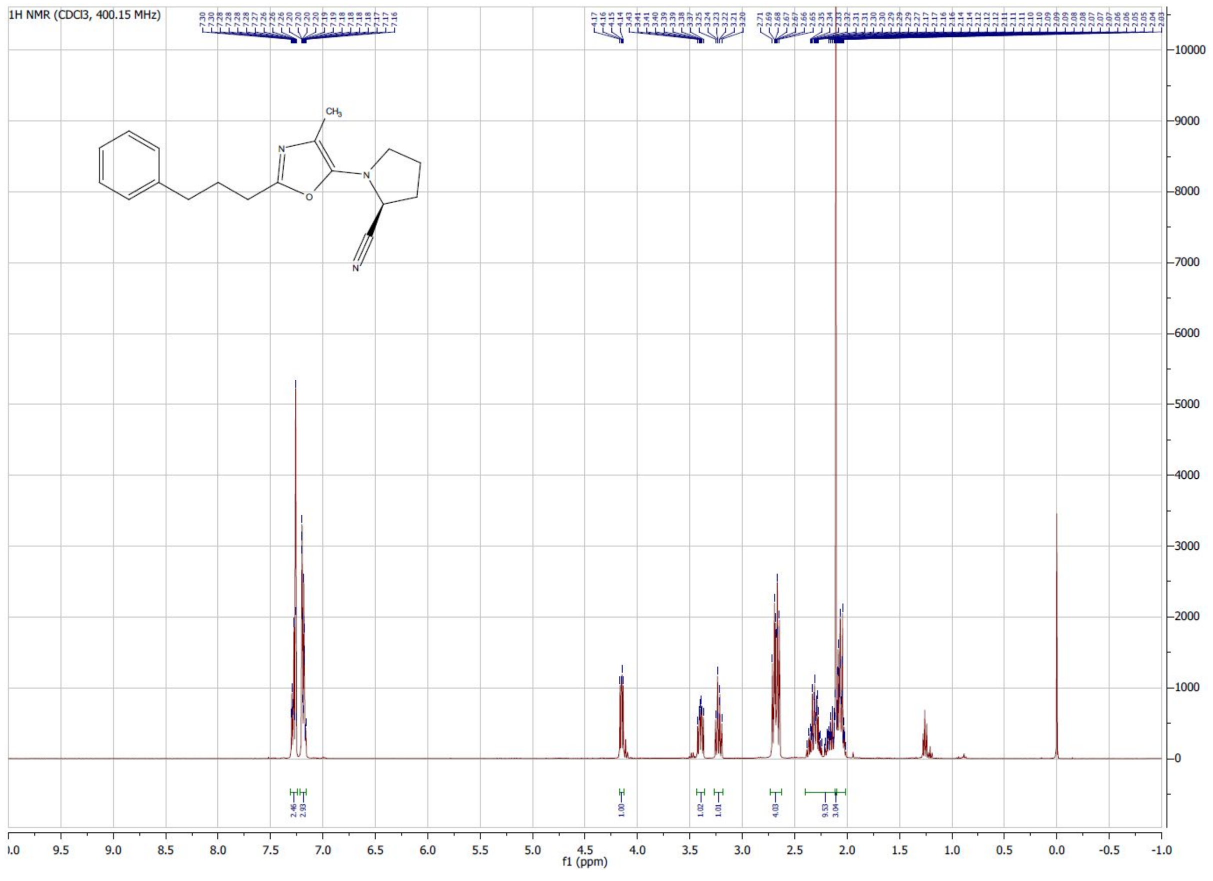

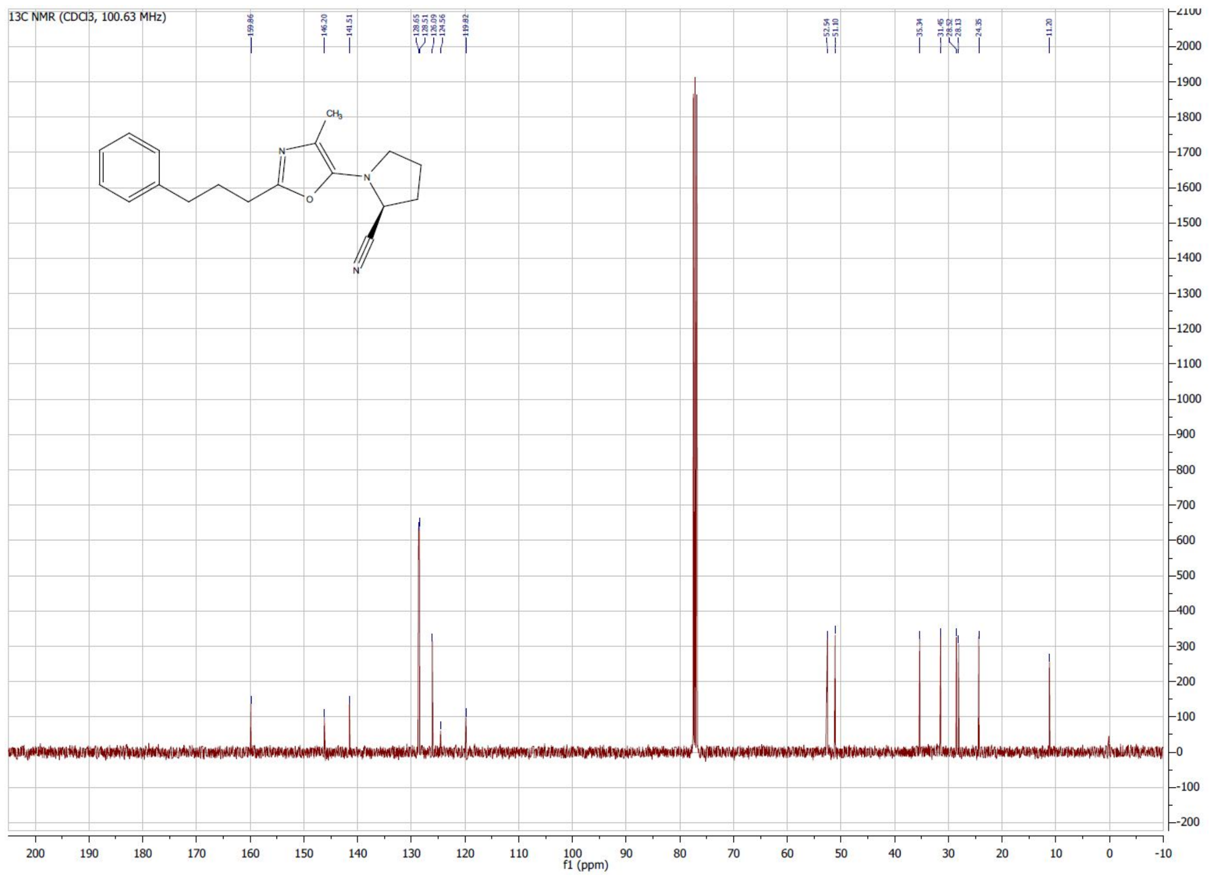

Figure S30. ^{1}H and ^{13}C NMR spectra of HUP-55.

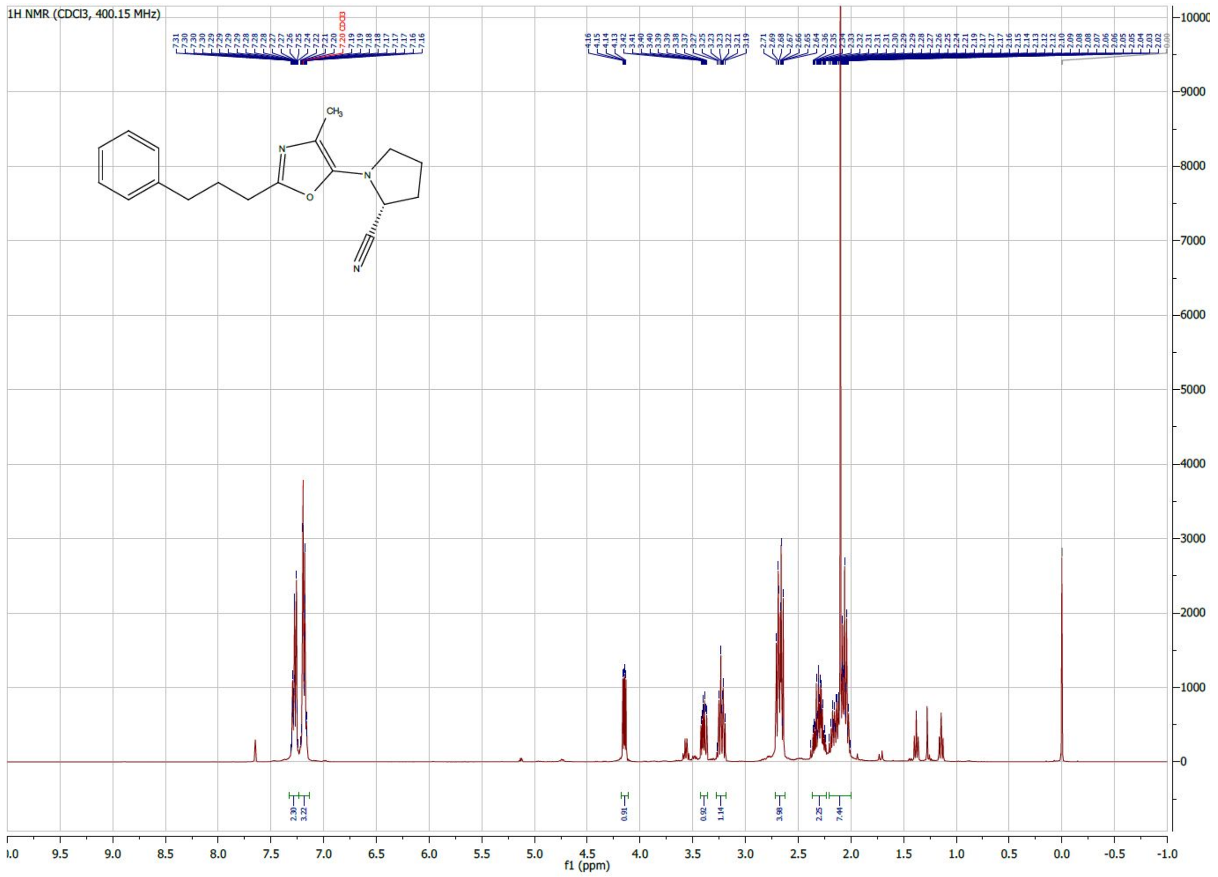

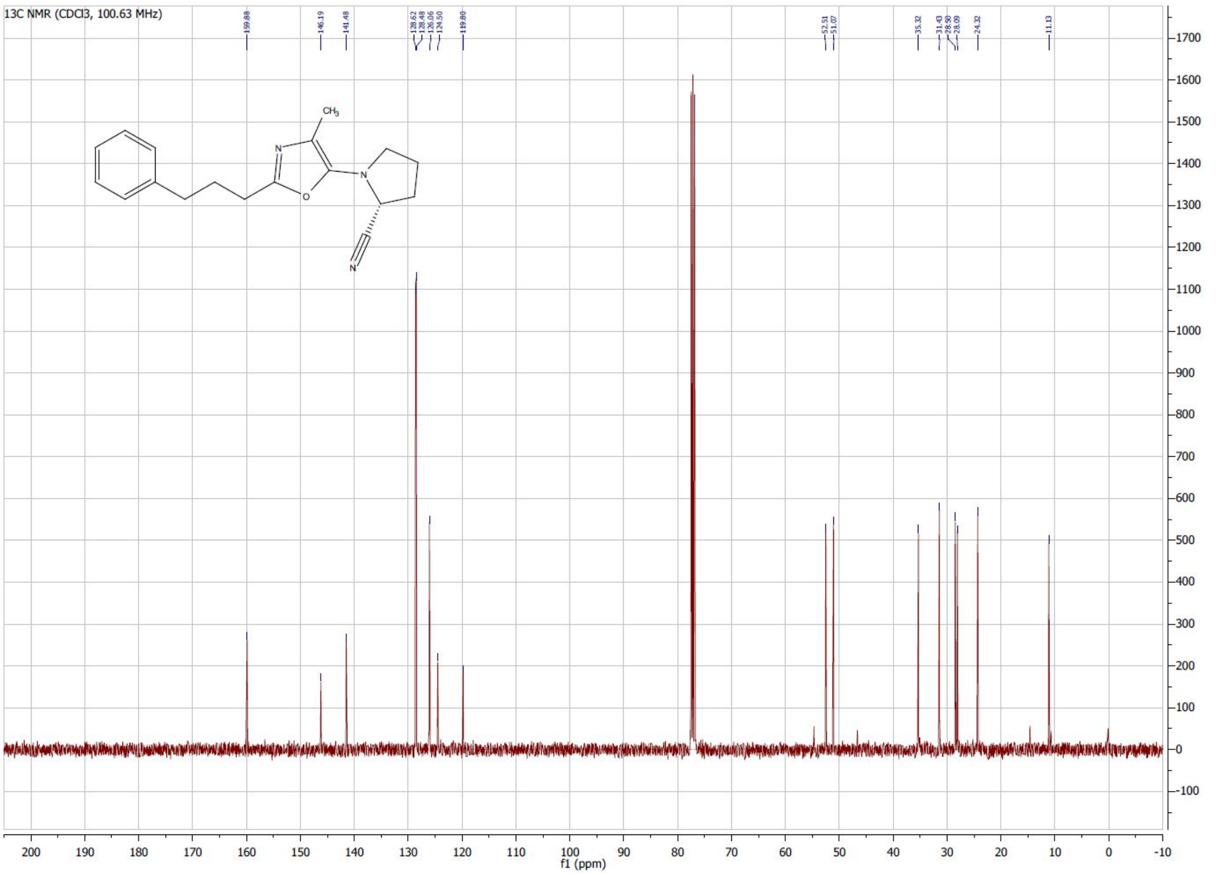

Figure S31. ^{1}H and ^{13}C NMR spectra of compound **3**.

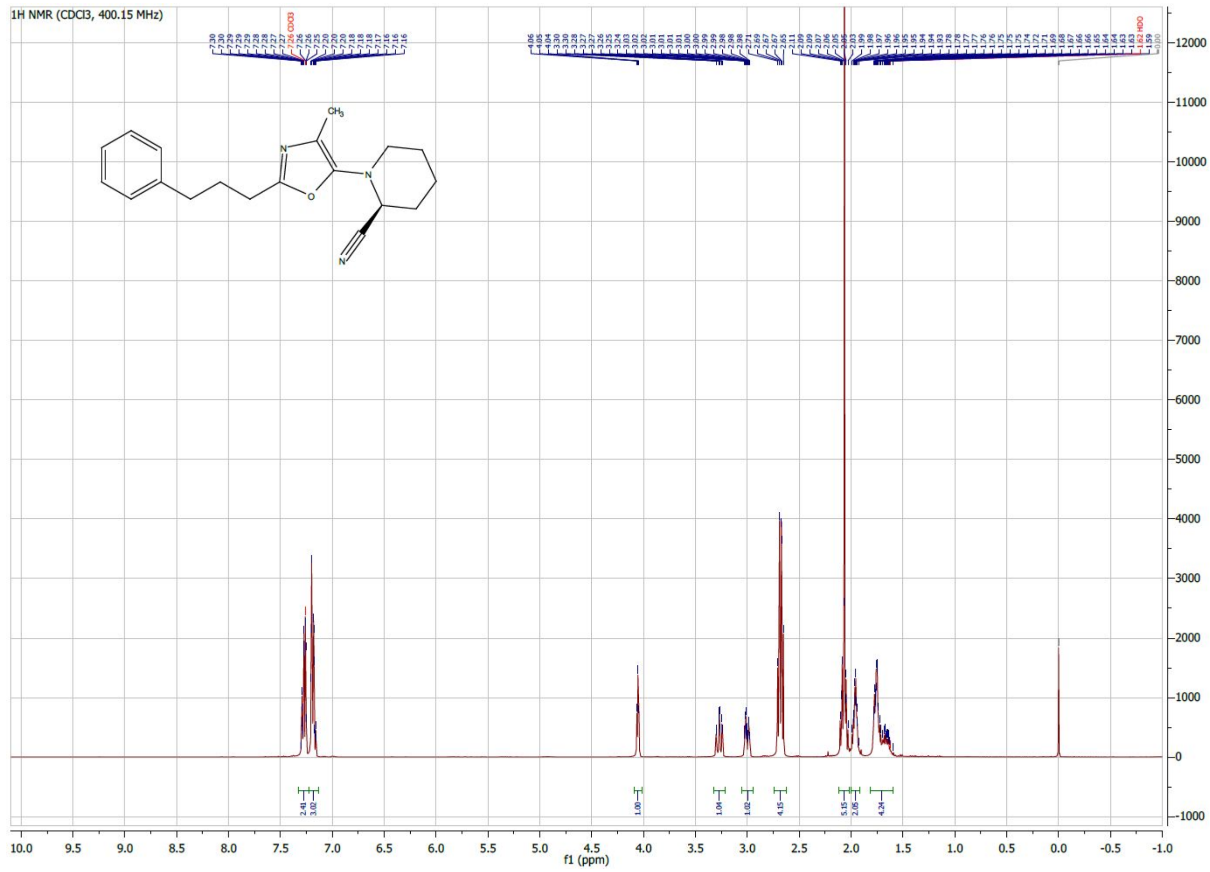

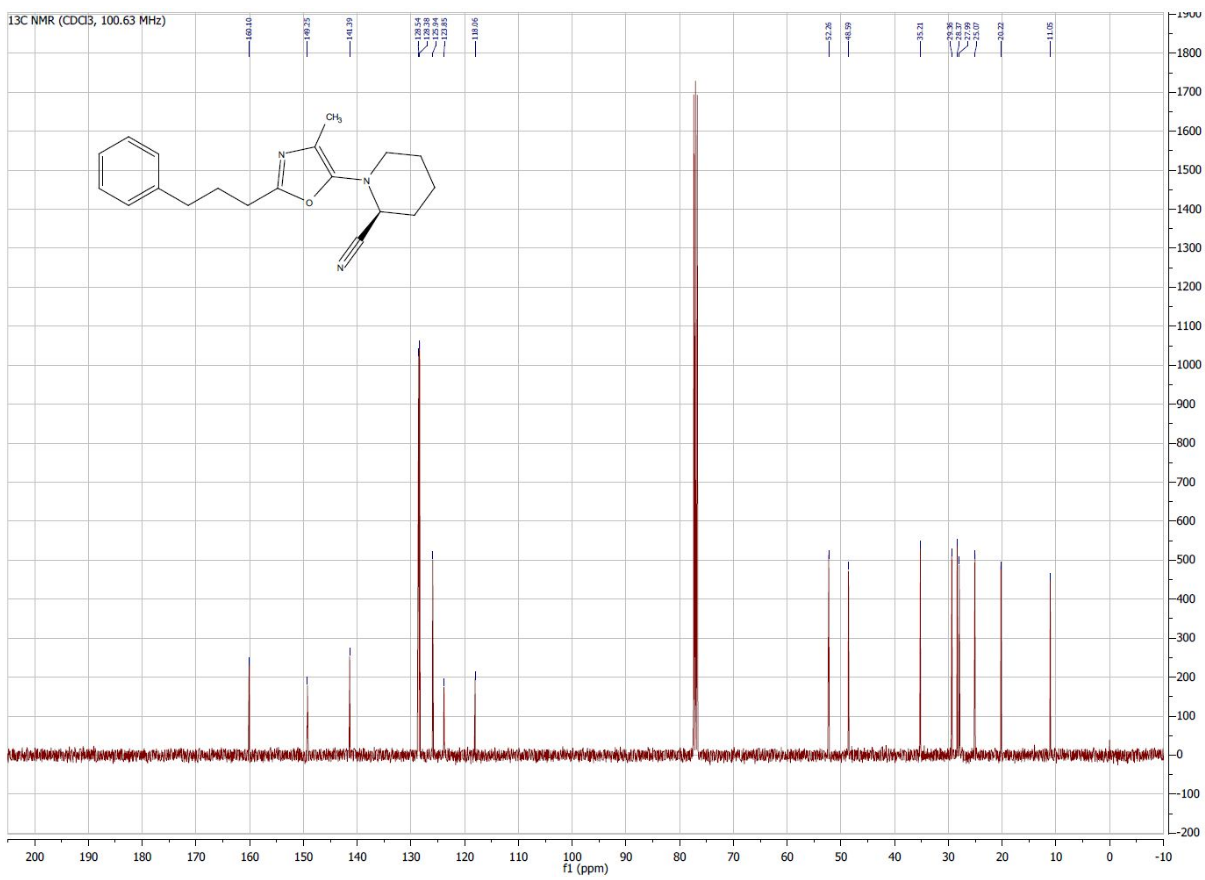

Figure S32. ^{1}H and ^{13}C NMR spectra of compound **4**.

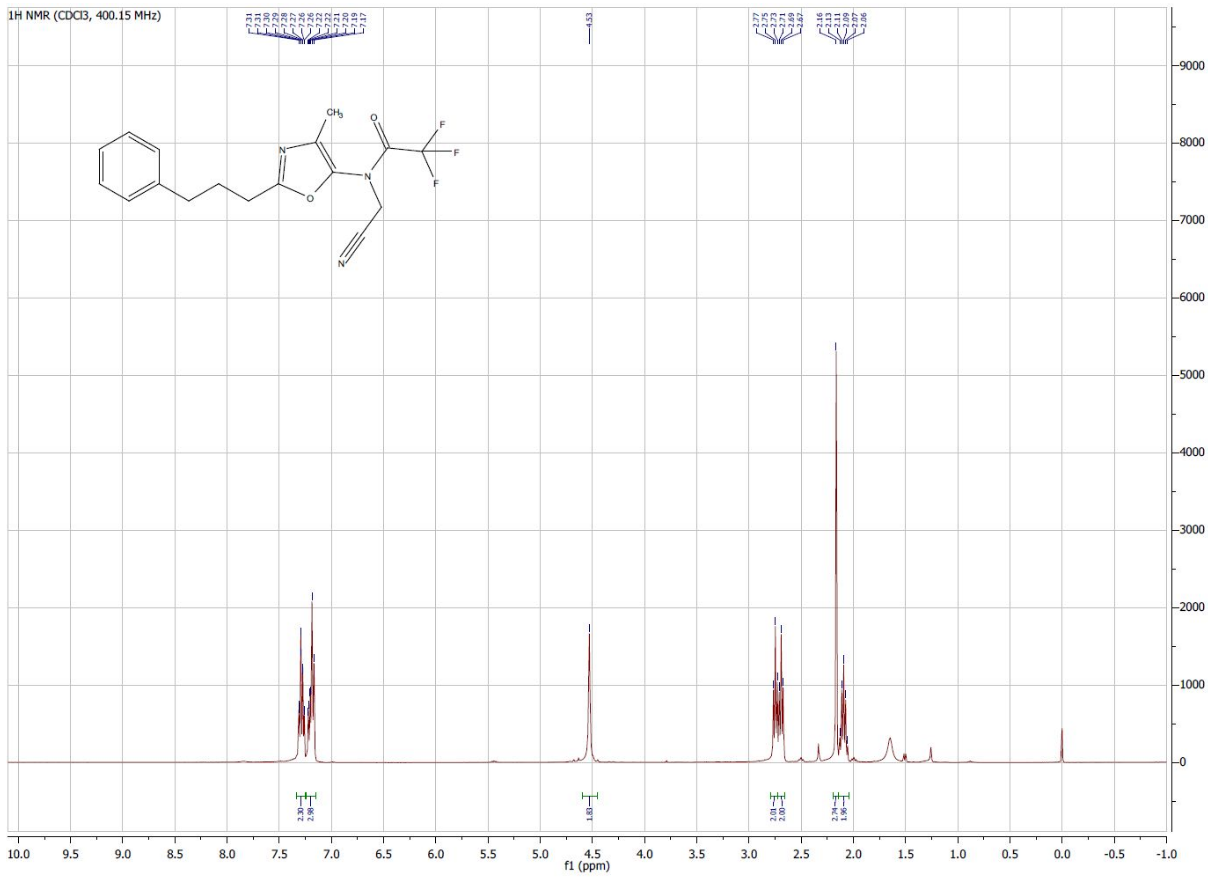

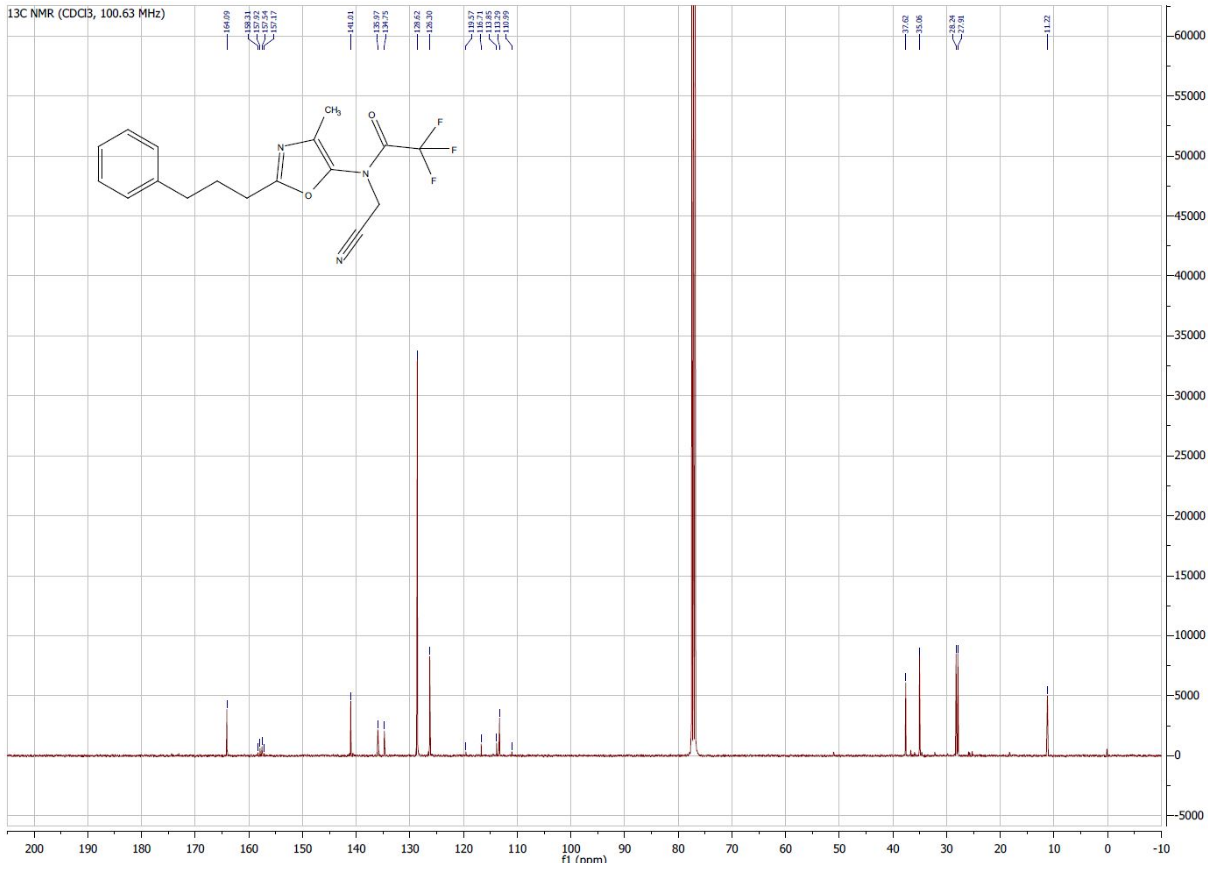

Figure S33. ^{1}H and ^{13}C NMR spectra of compound **5**.

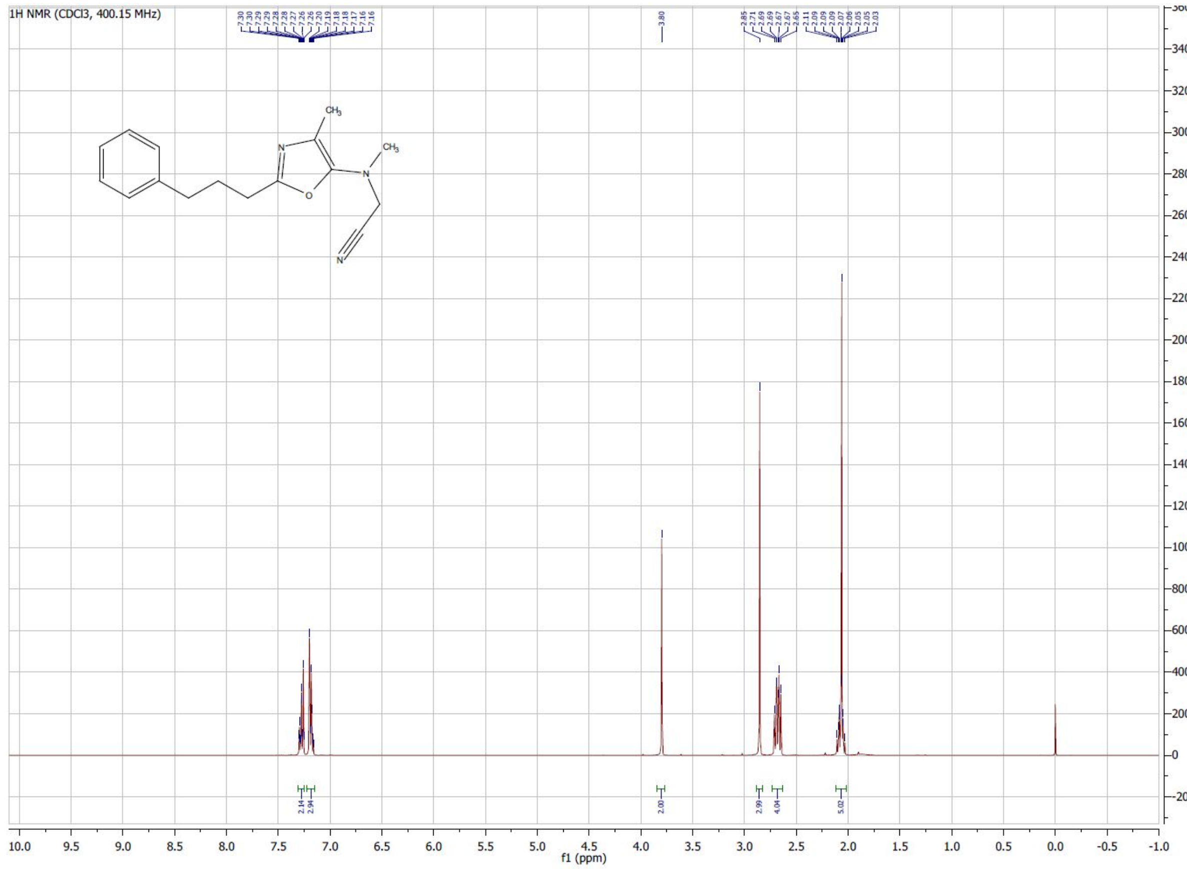

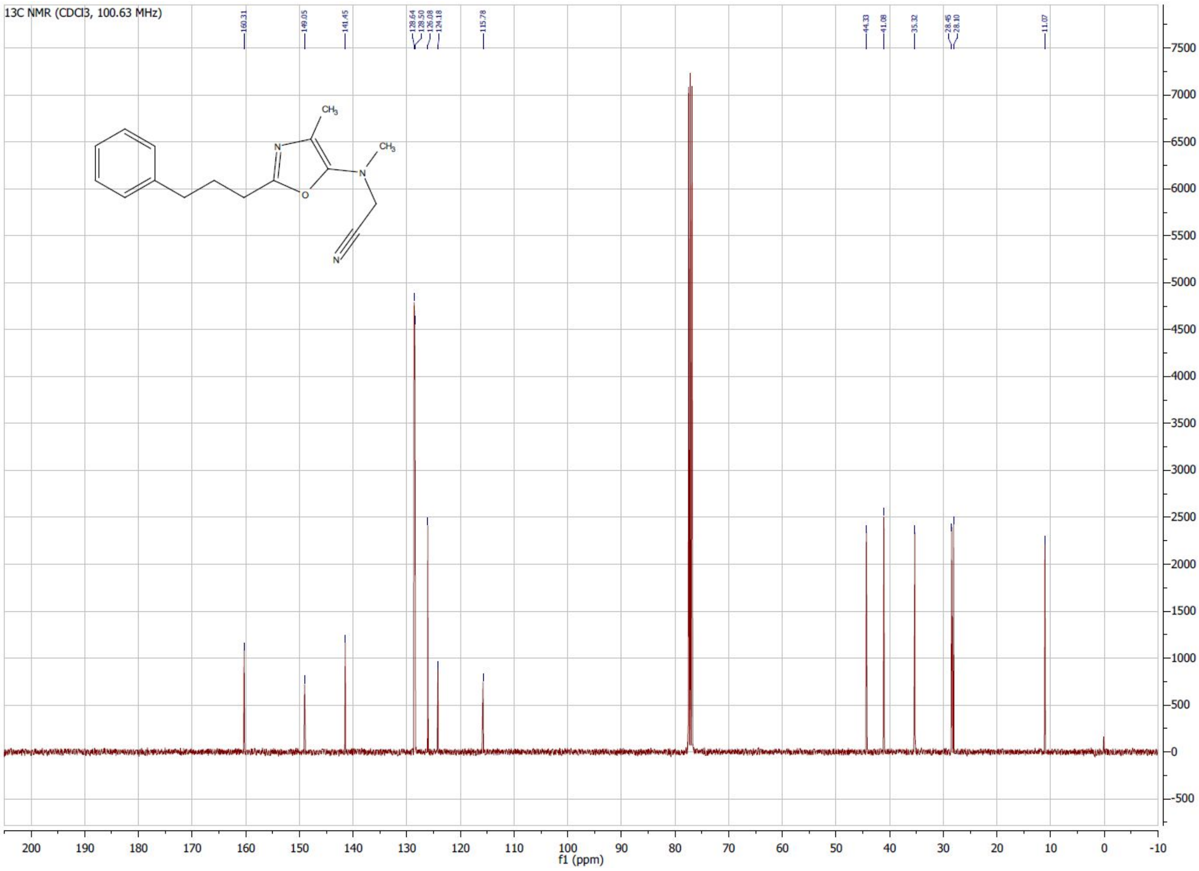

Figure S34. ^{1}H and ^{13}C NMR spectra of compound **6**.

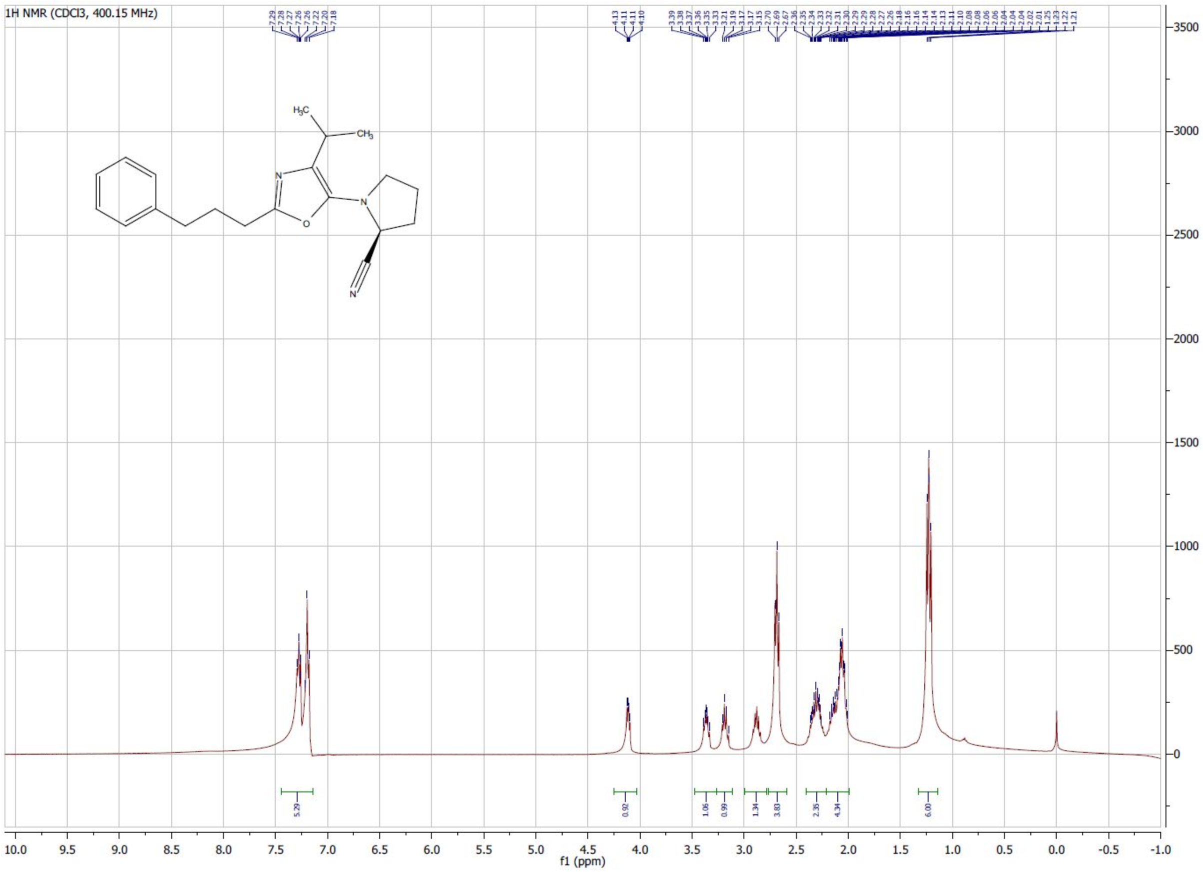

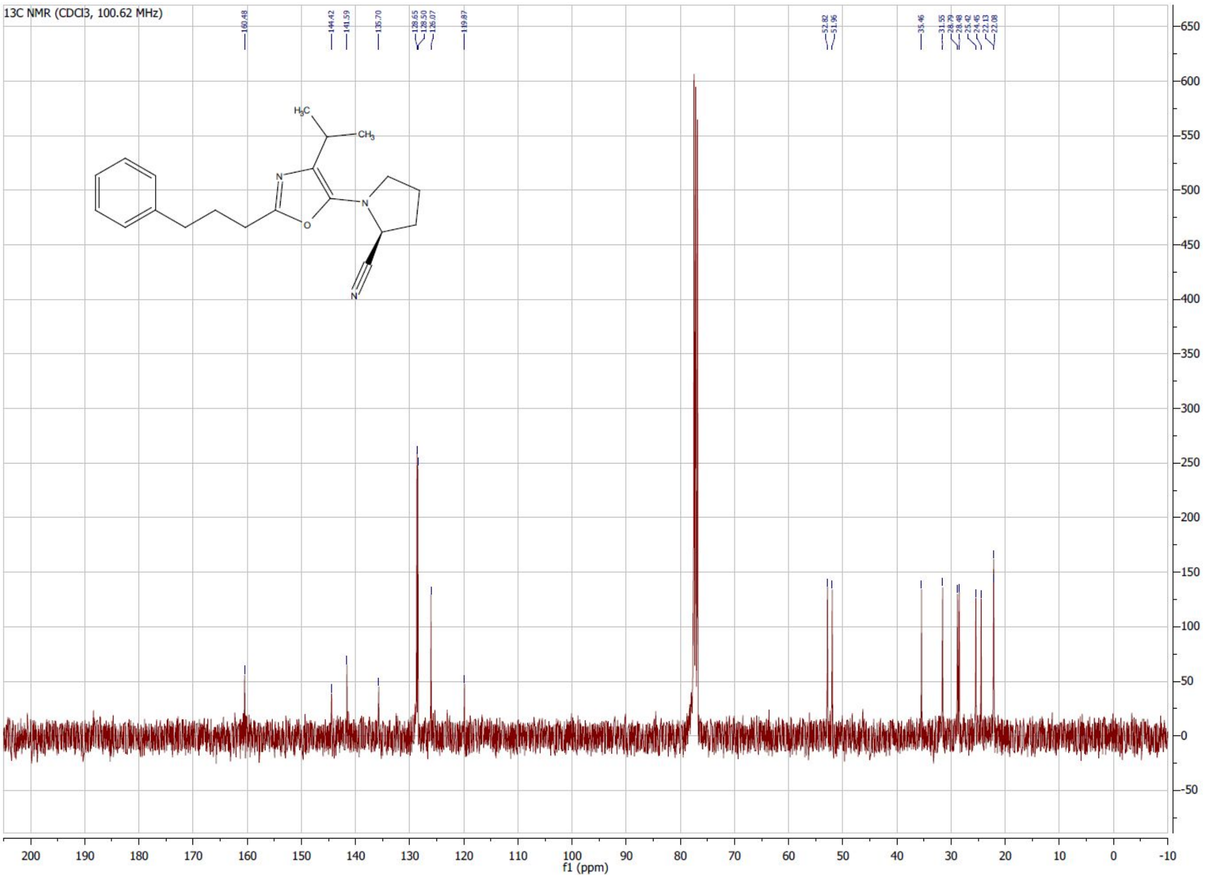

Figure S35. ^{1}H and ^{13}C NMR spectra of compound **7**.

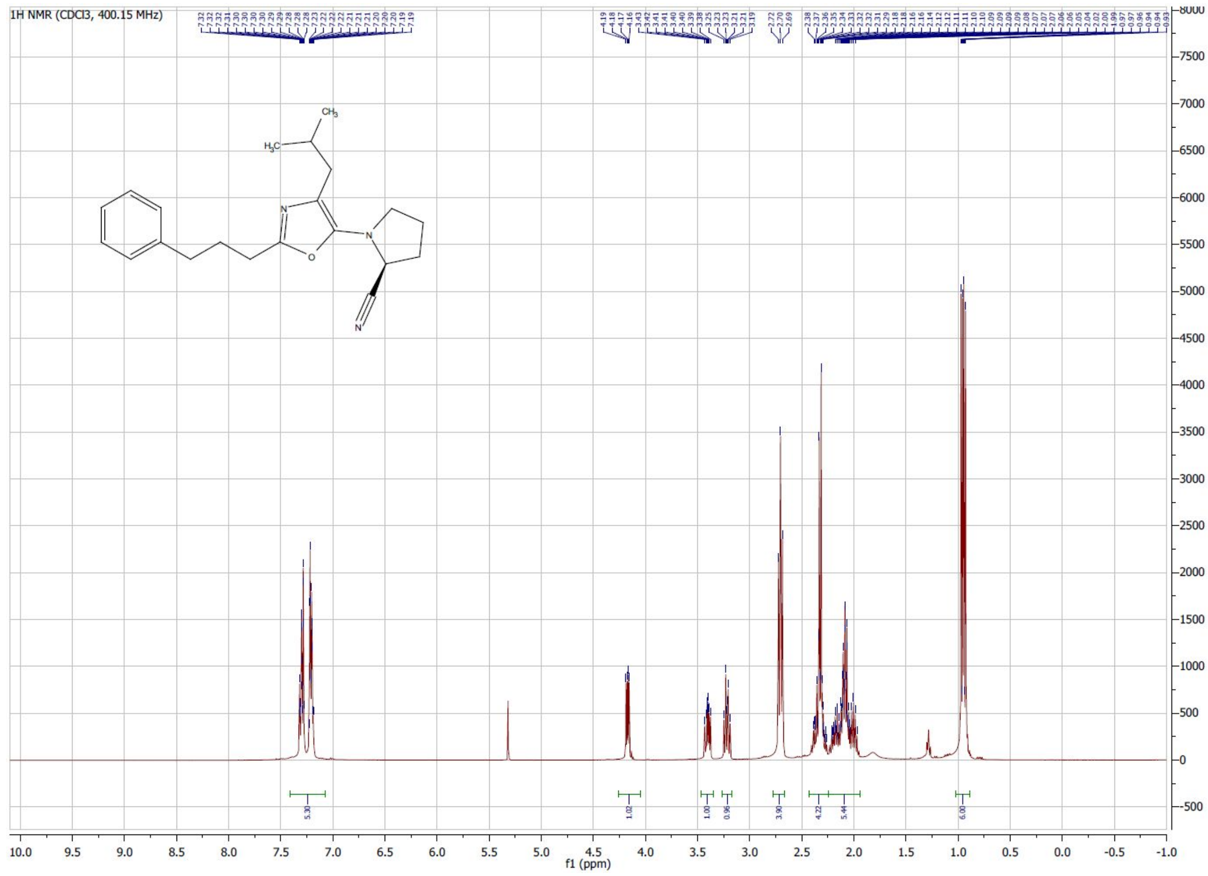

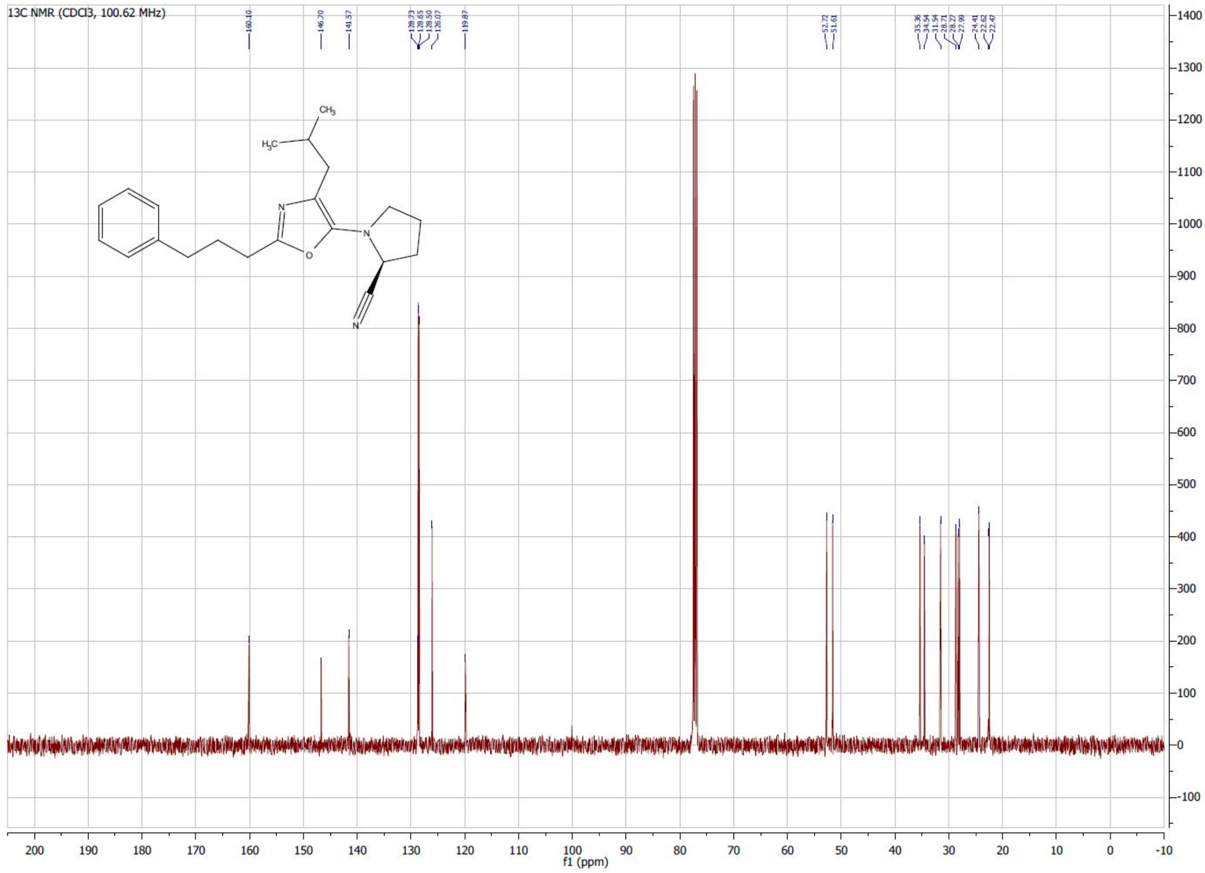

Figure S36. ^{1}H and ^{13}C NMR spectra of compound **8**.

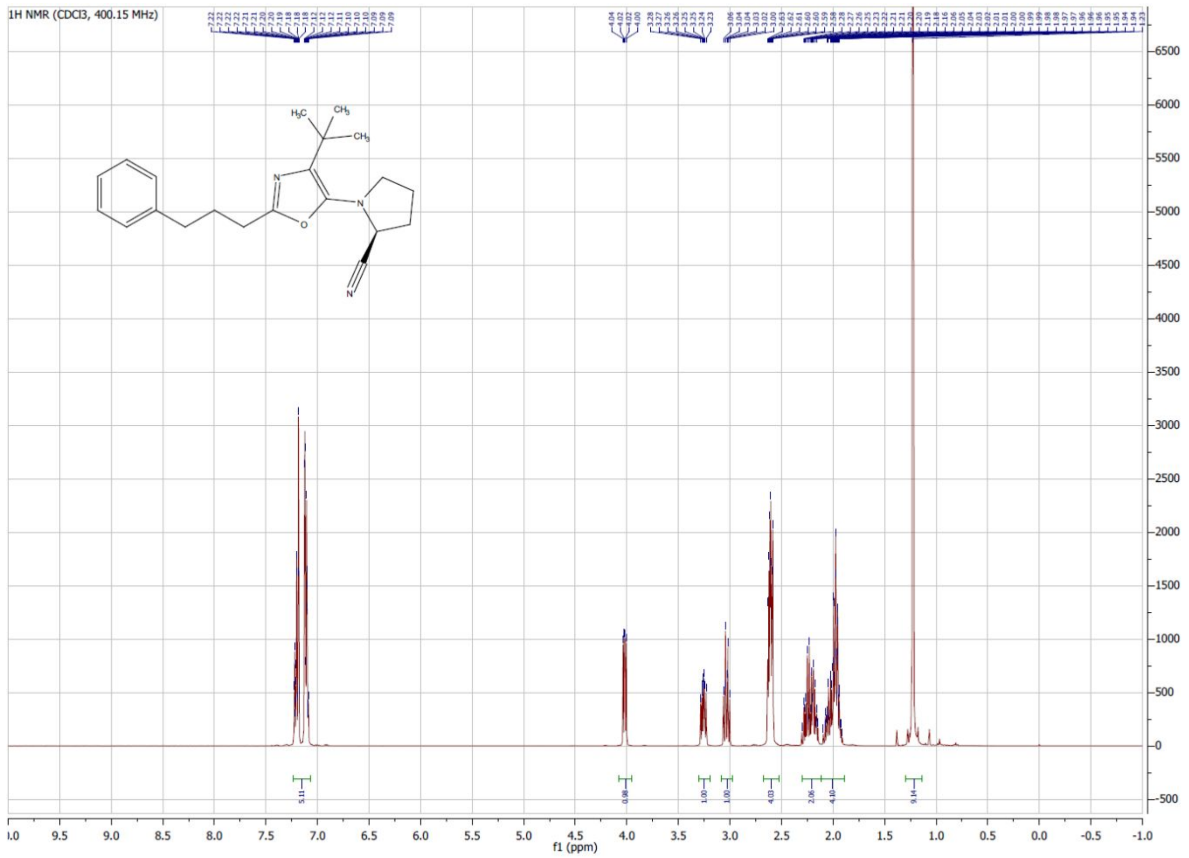

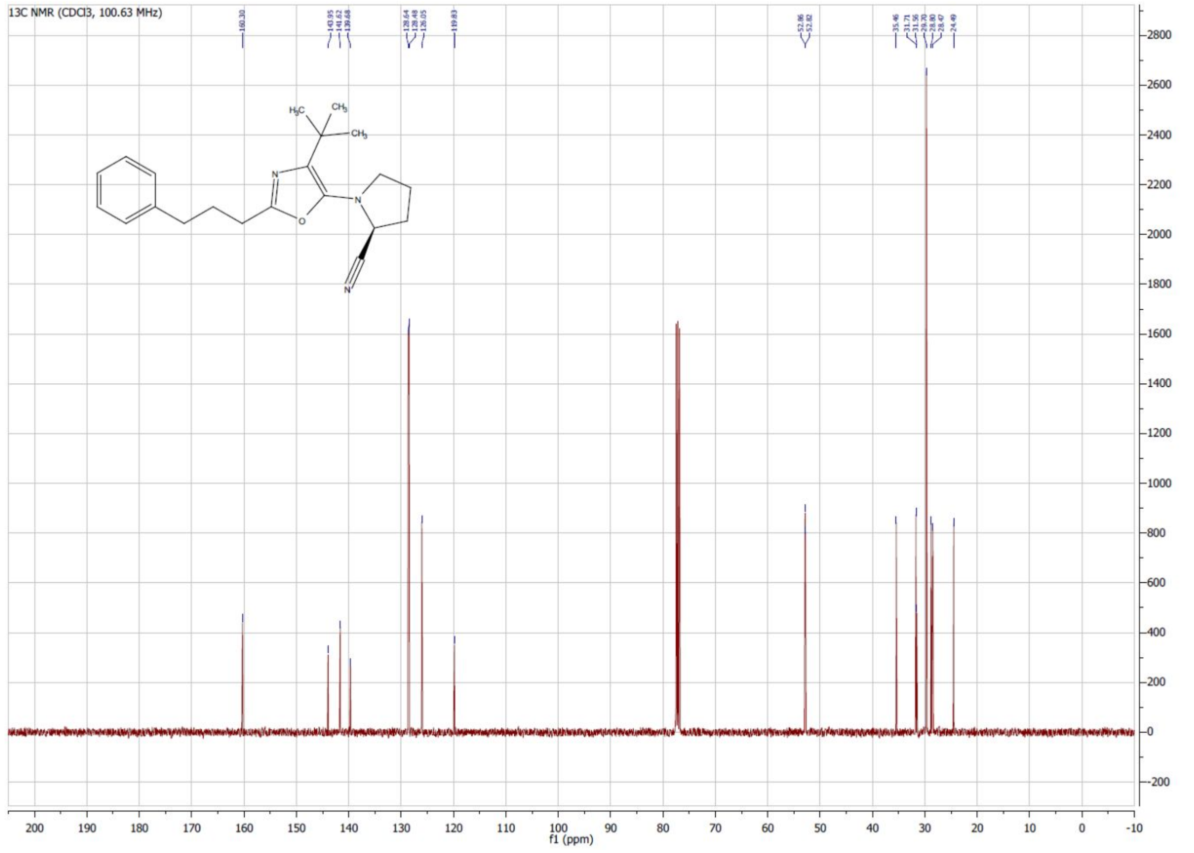

Figure S37. ^{1}H and ^{13}C NMR spectra of compound **9**.

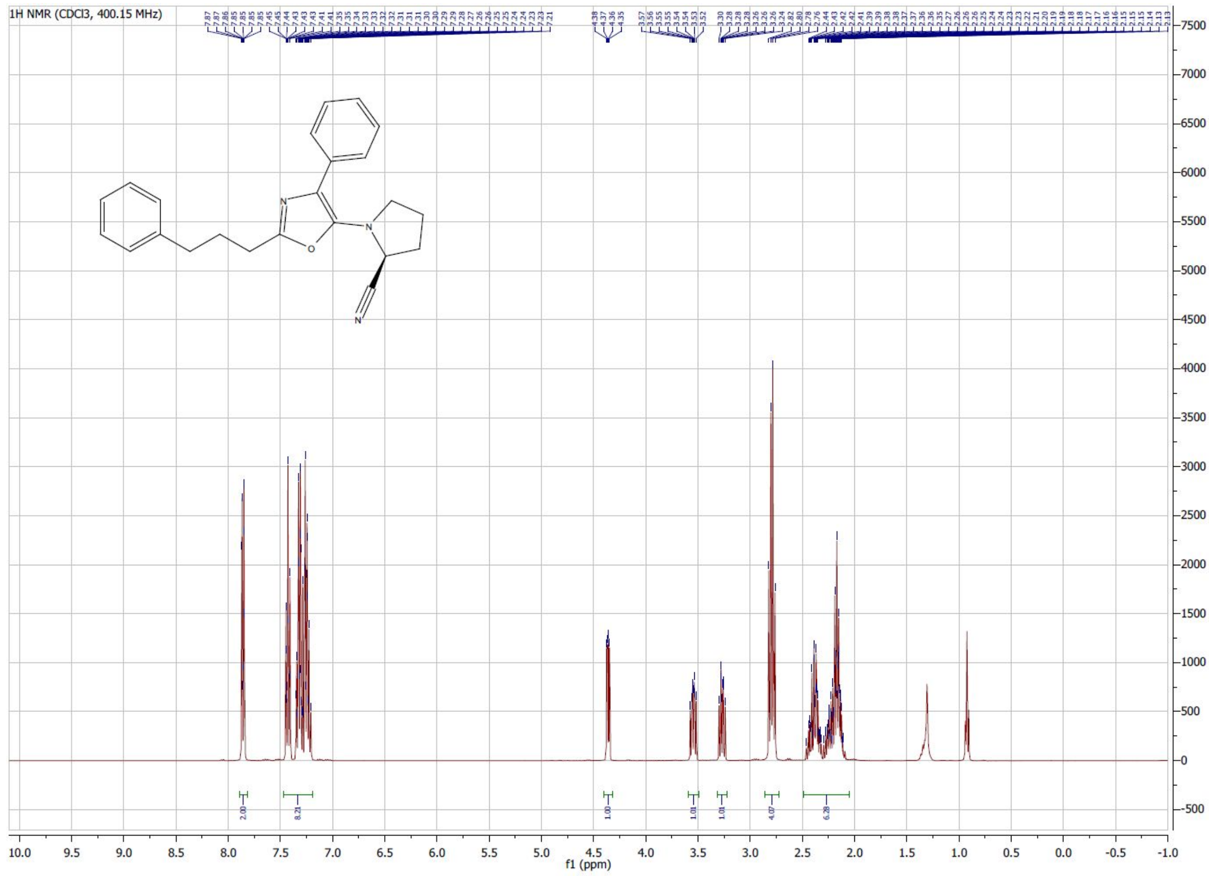

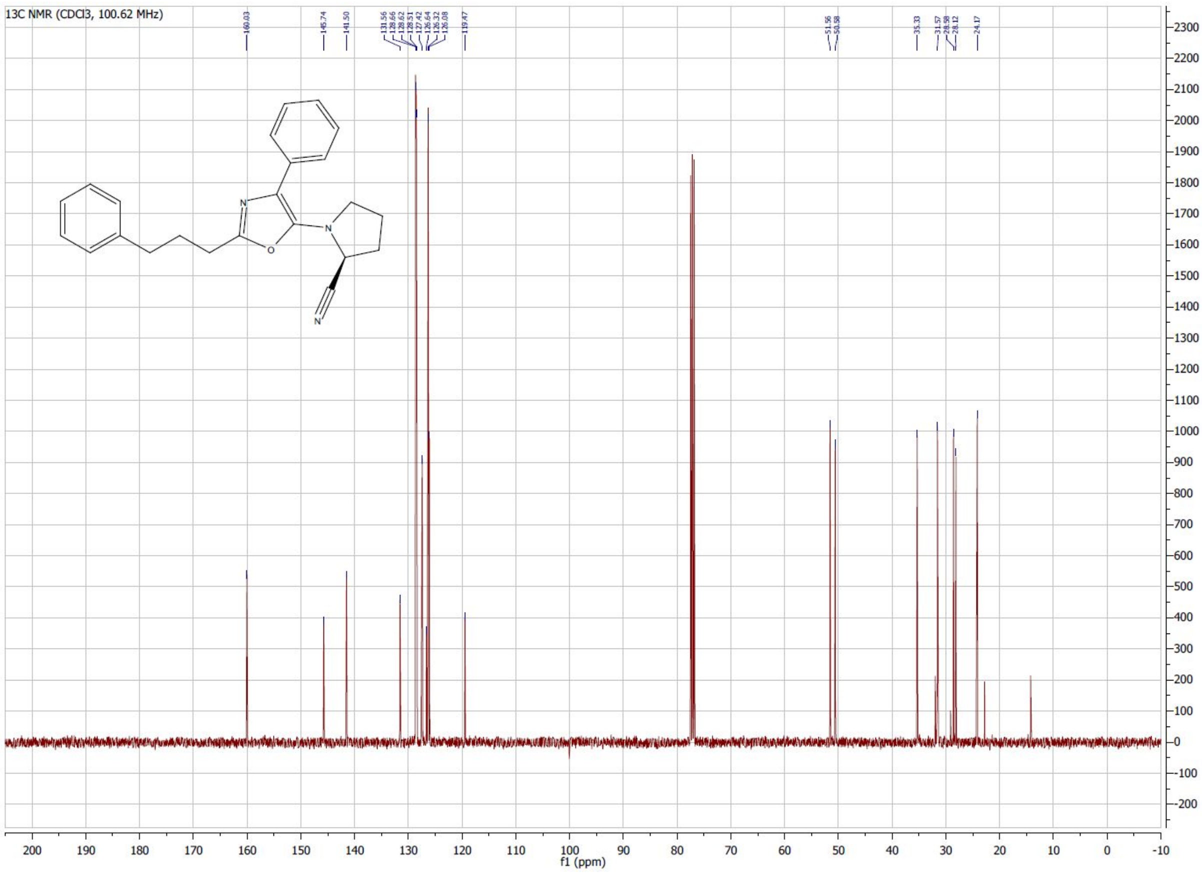

Figure S38. ^{1}H and ^{13}C NMR spectra of compound **10**.

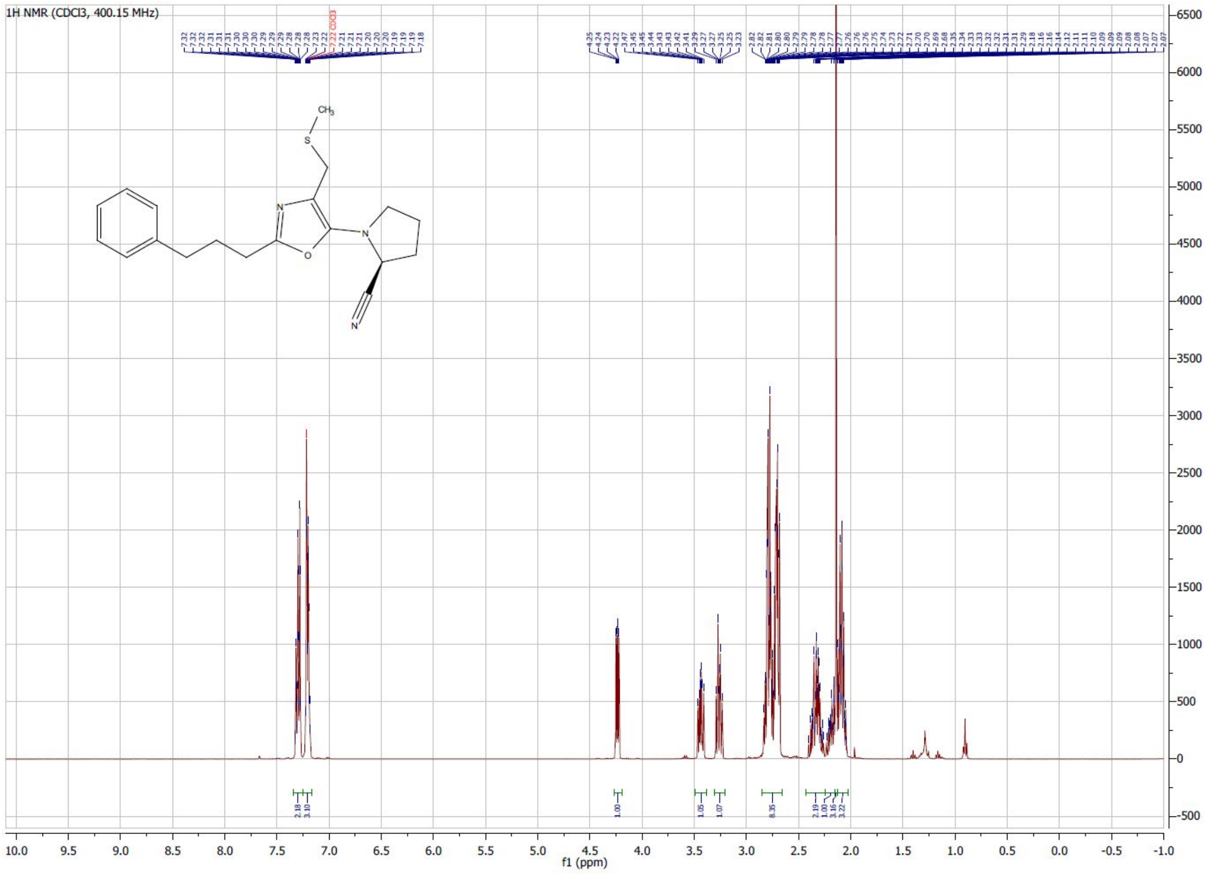

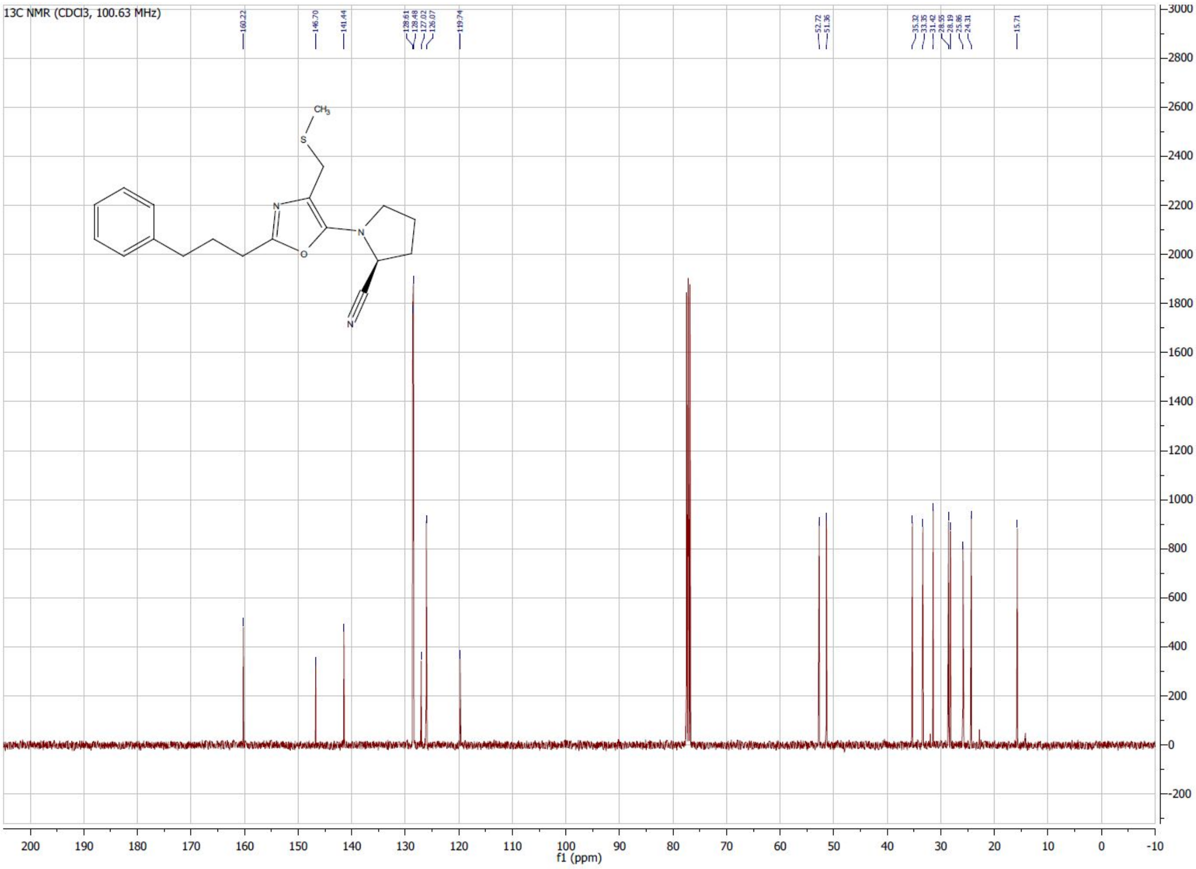

Figure S39. ^{1}H and ^{13}C NMR spectra of compound **11**.

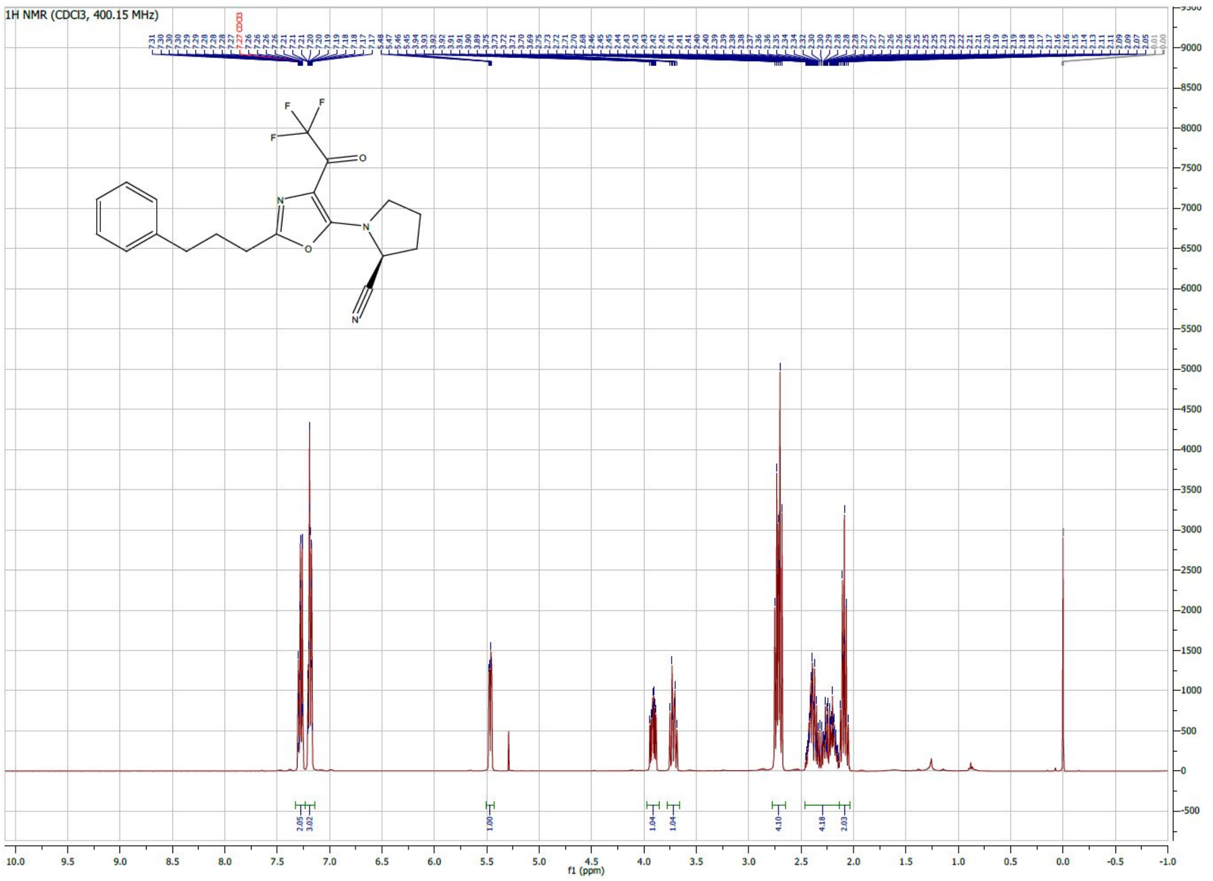

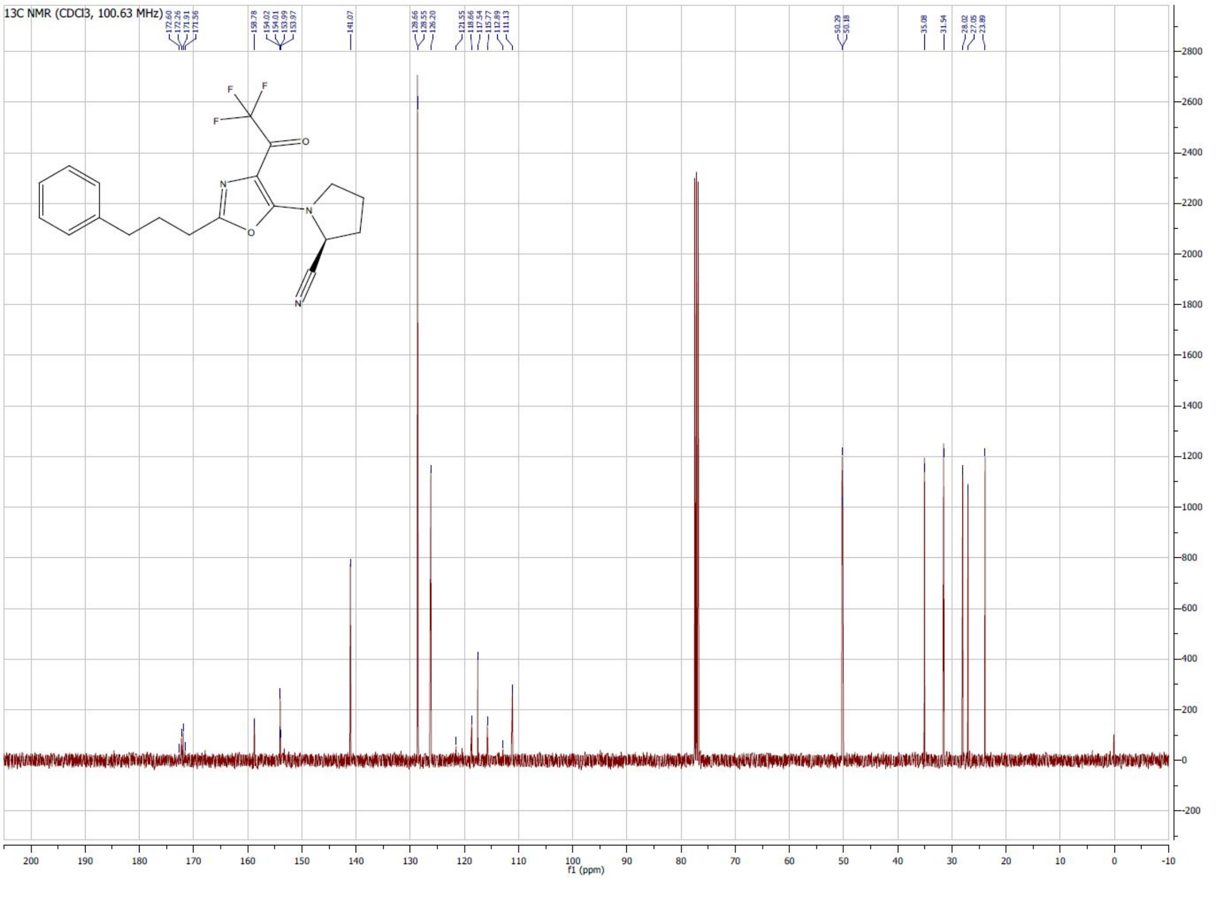

Figure S40. ^{1}H and ^{13}C NMR spectra of compound **12**.

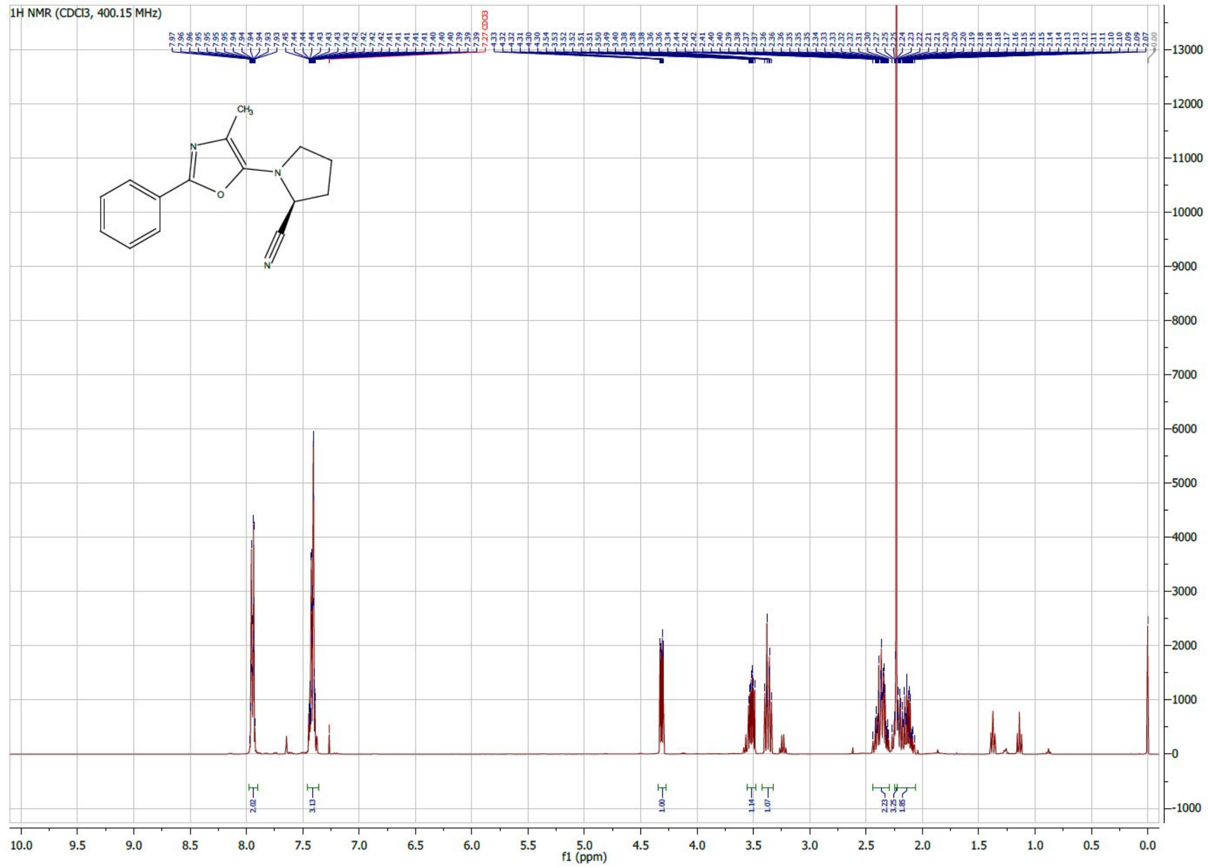

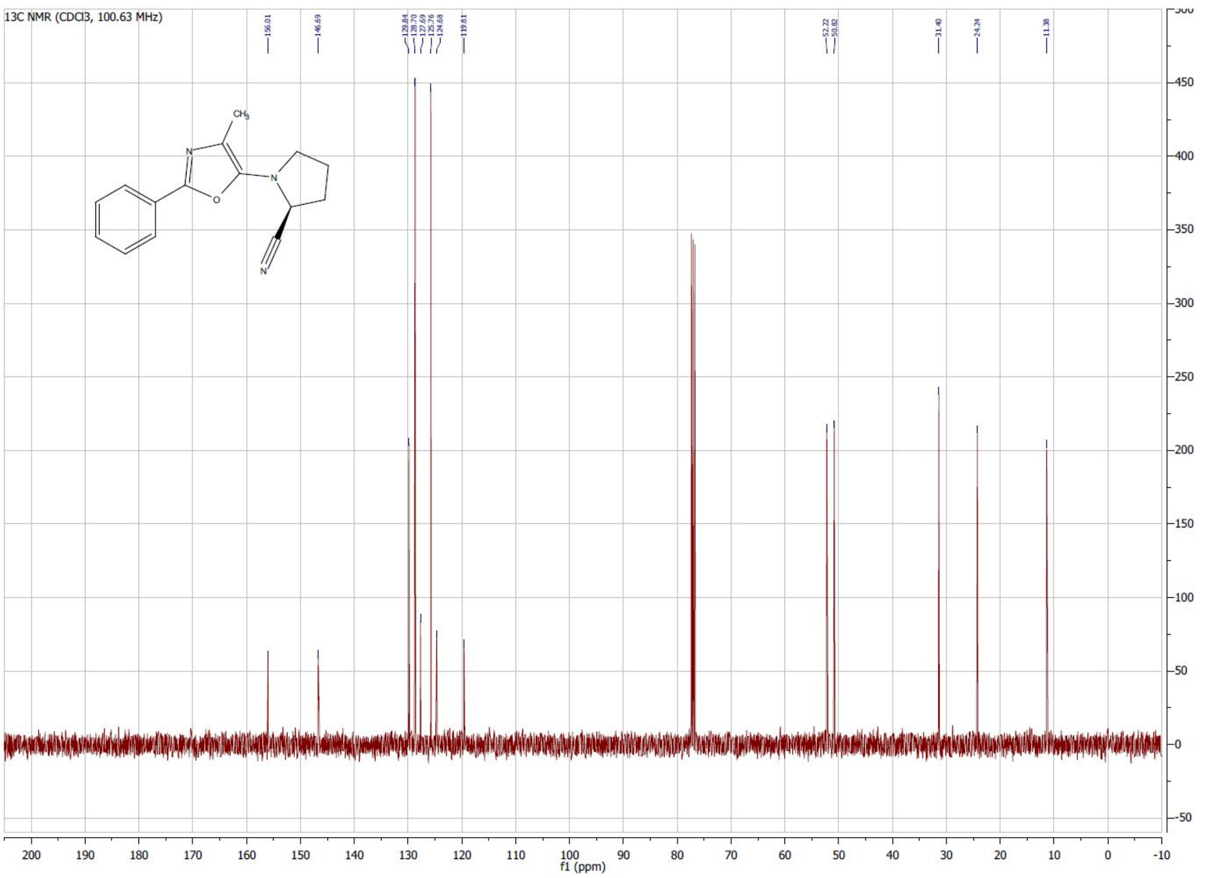

Figure S41. ^{1}H and ^{13}C NMR spectra of compound **13**.

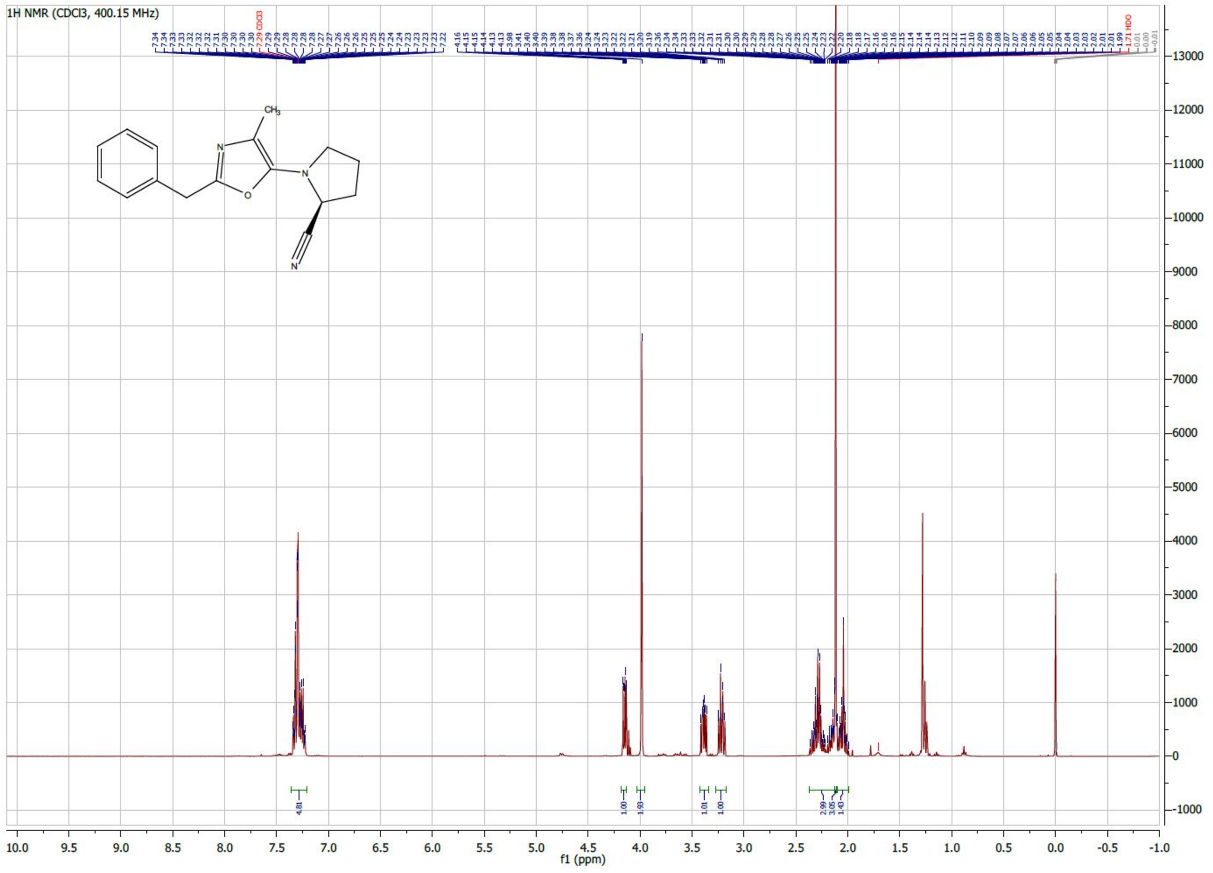

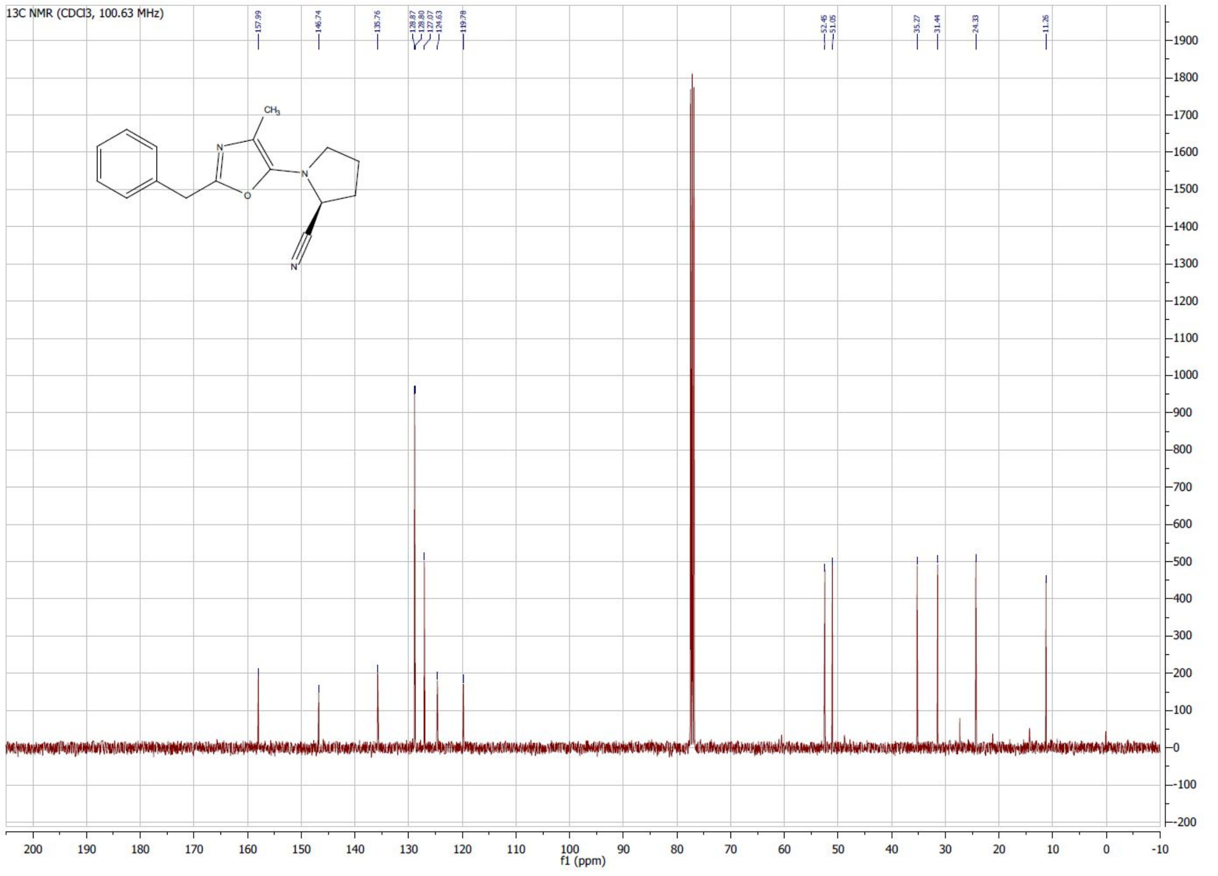

Figure S42. ^{1}H and ^{13}C NMR spectra of compound **14**.

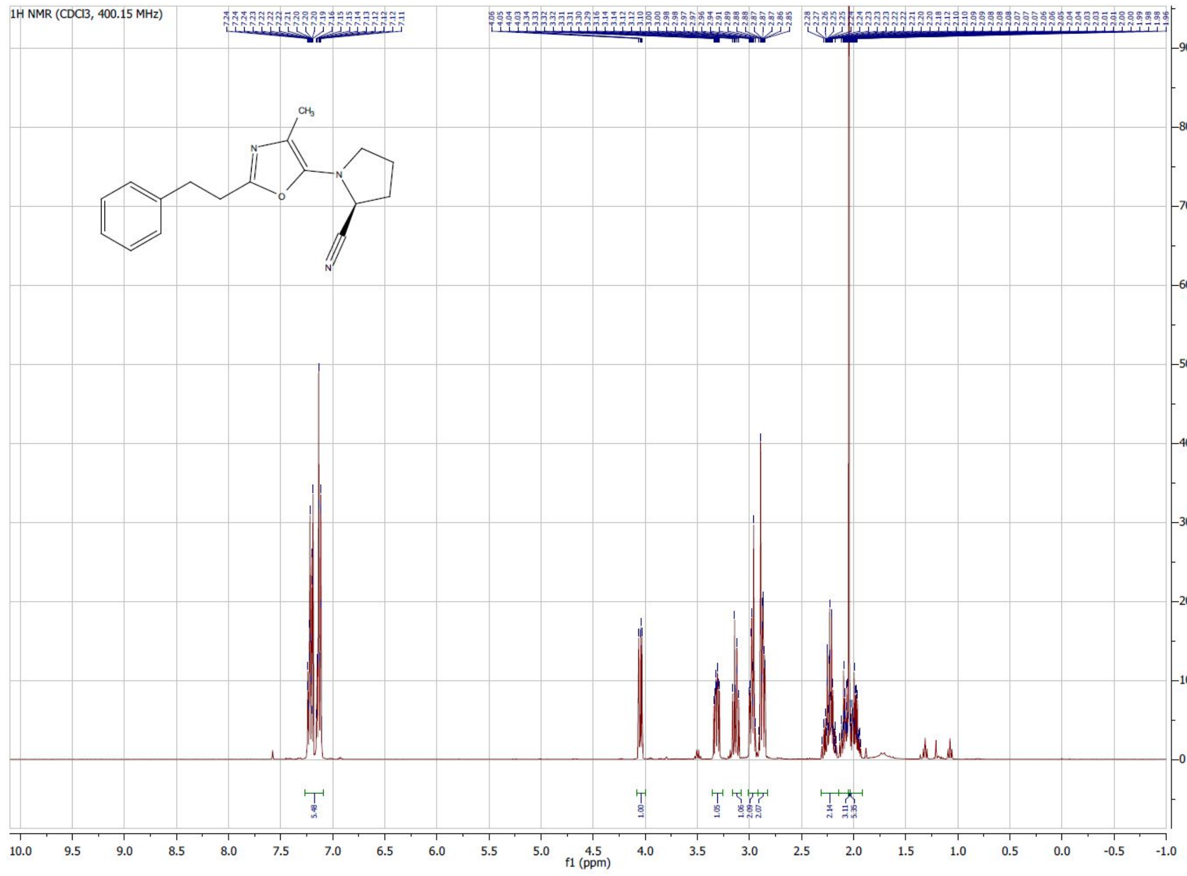

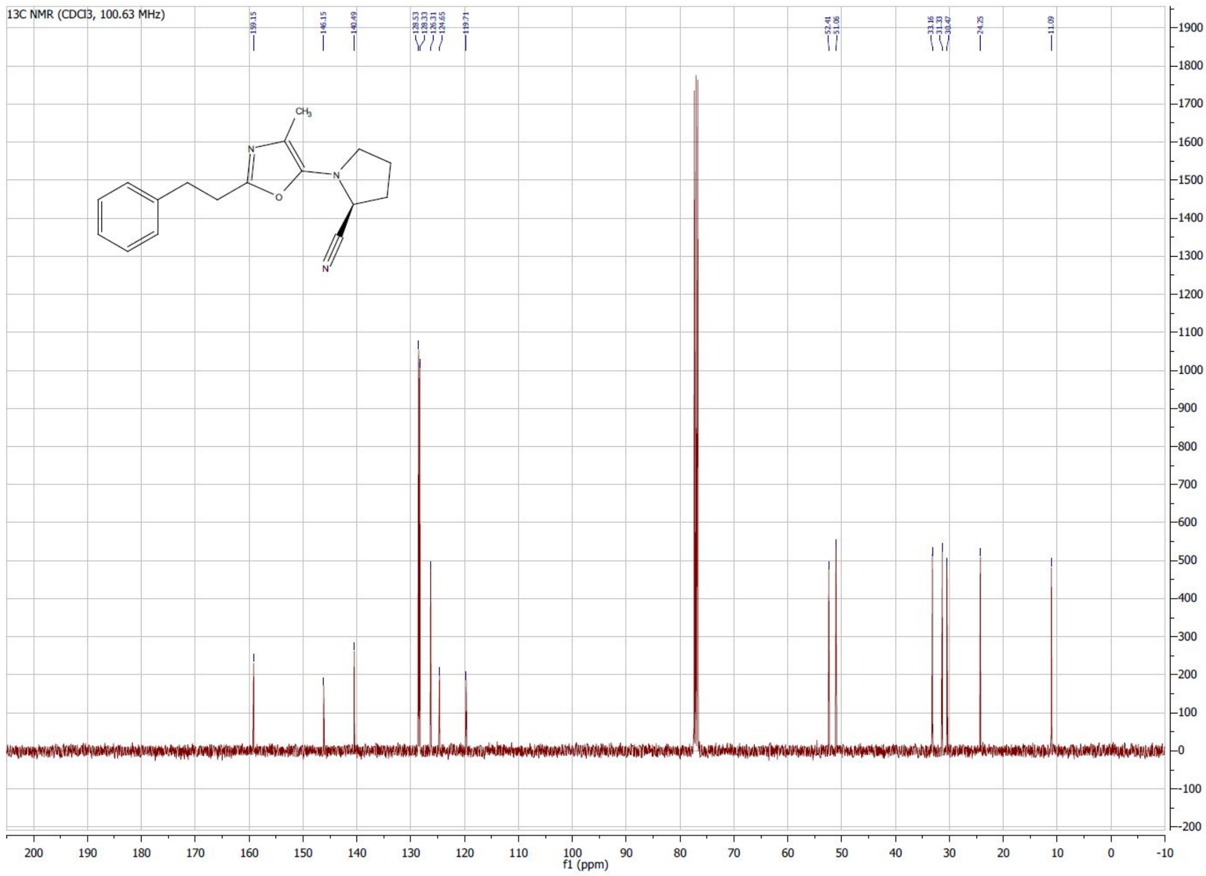

Figure S43. ^{1}H and ^{13}C NMR spectra of compound **15**.

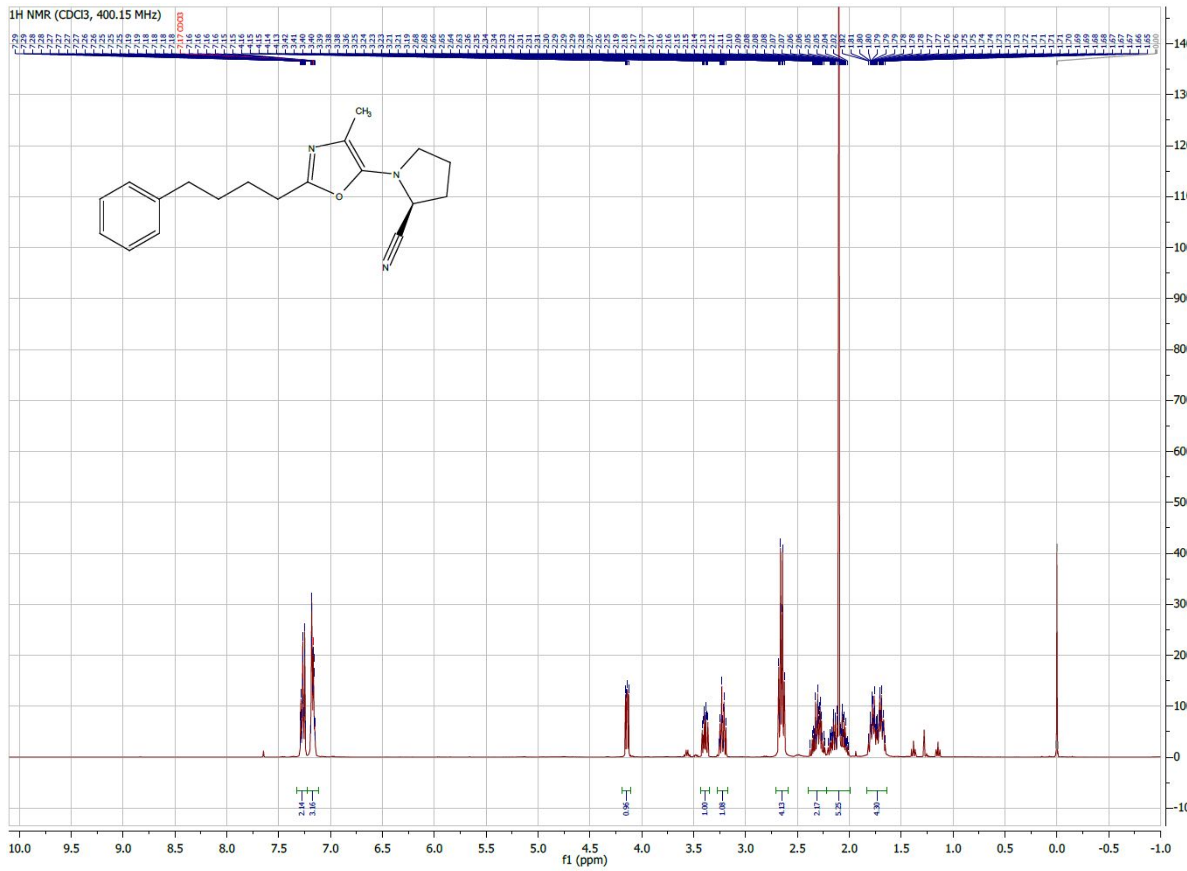

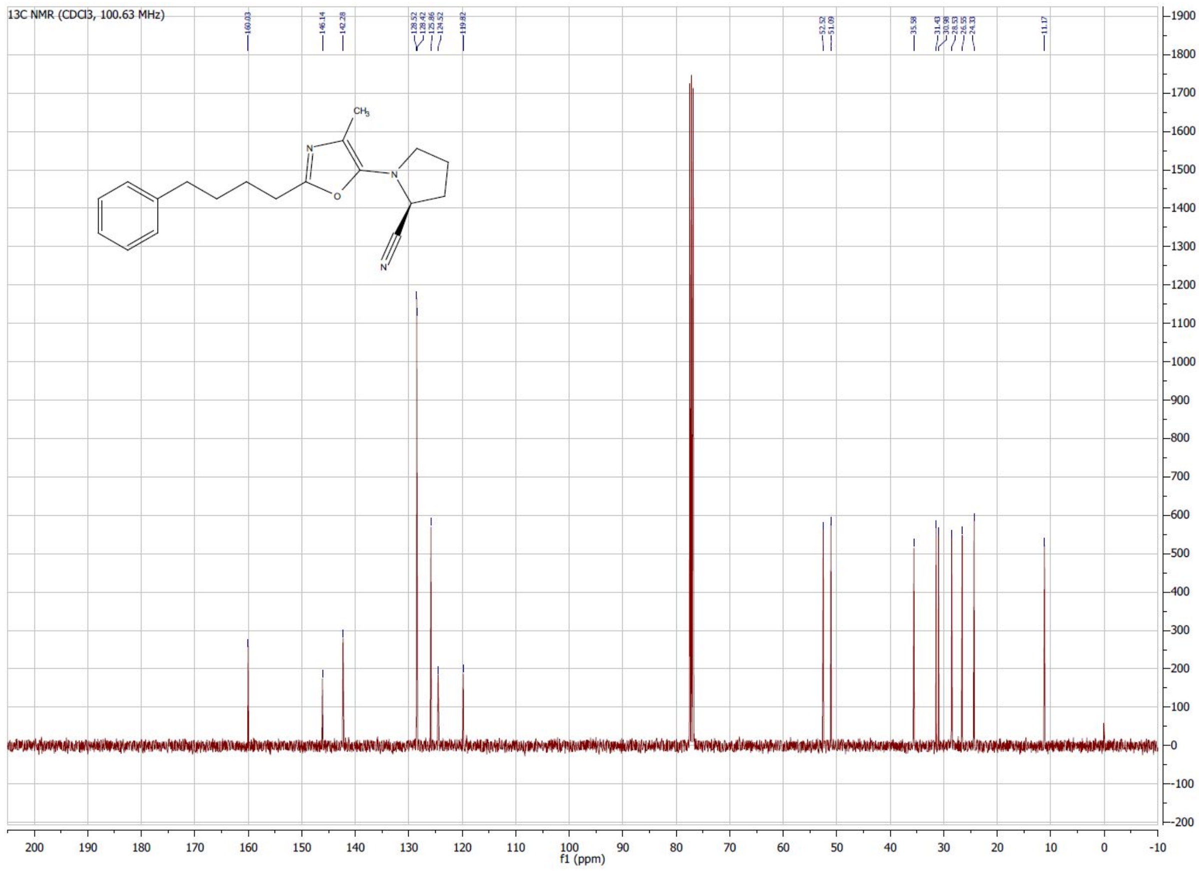

Figure S44. ^{1}H and ^{13}C NMR spectra of compound **16**.

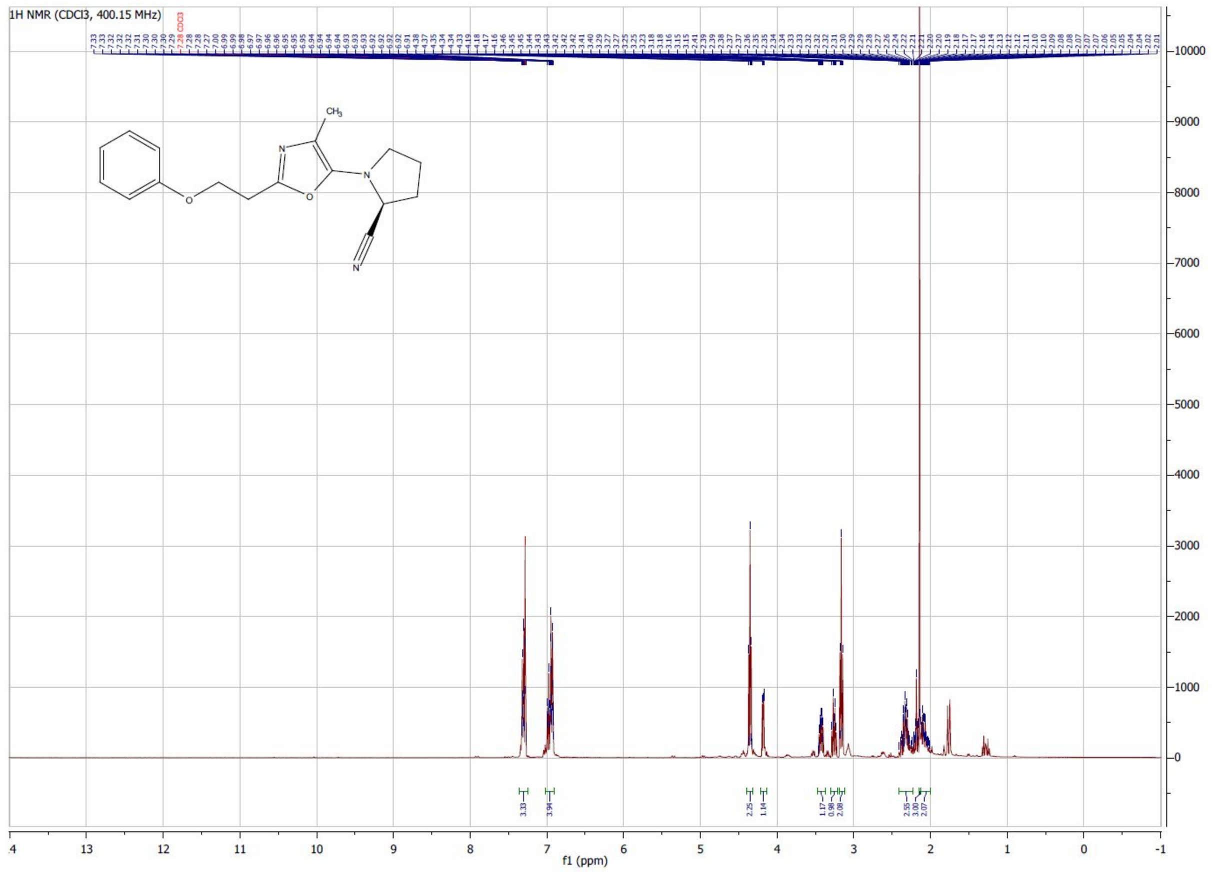

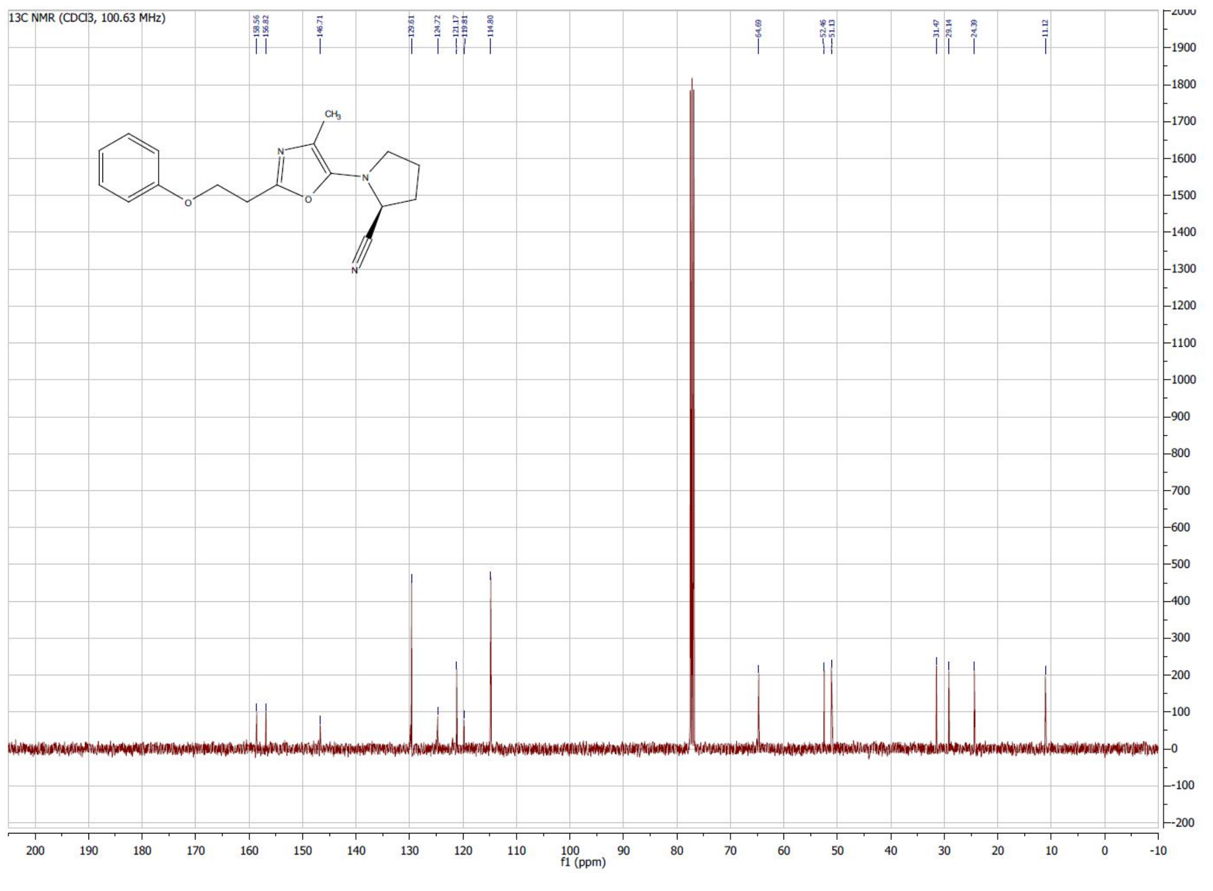

Figure S45. ^{1}H and ^{13}C NMR spectra of compound **17**.

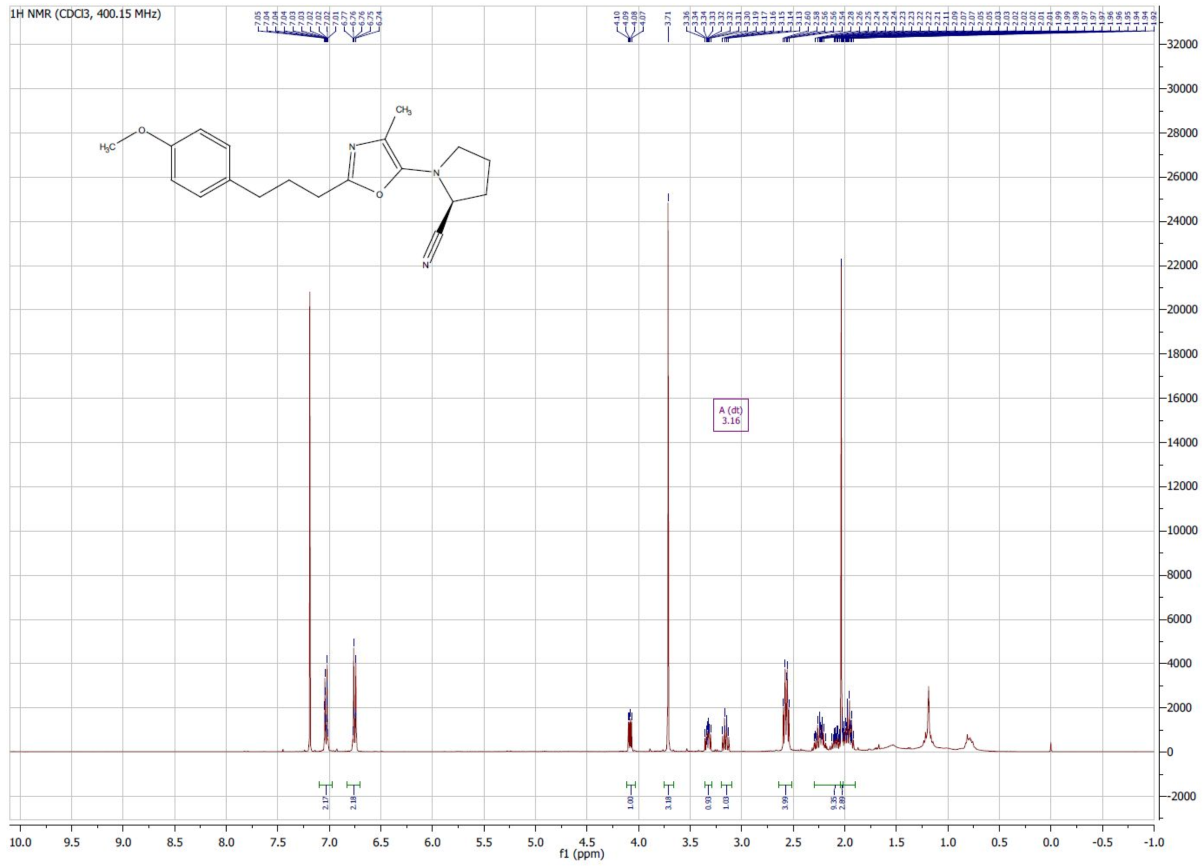

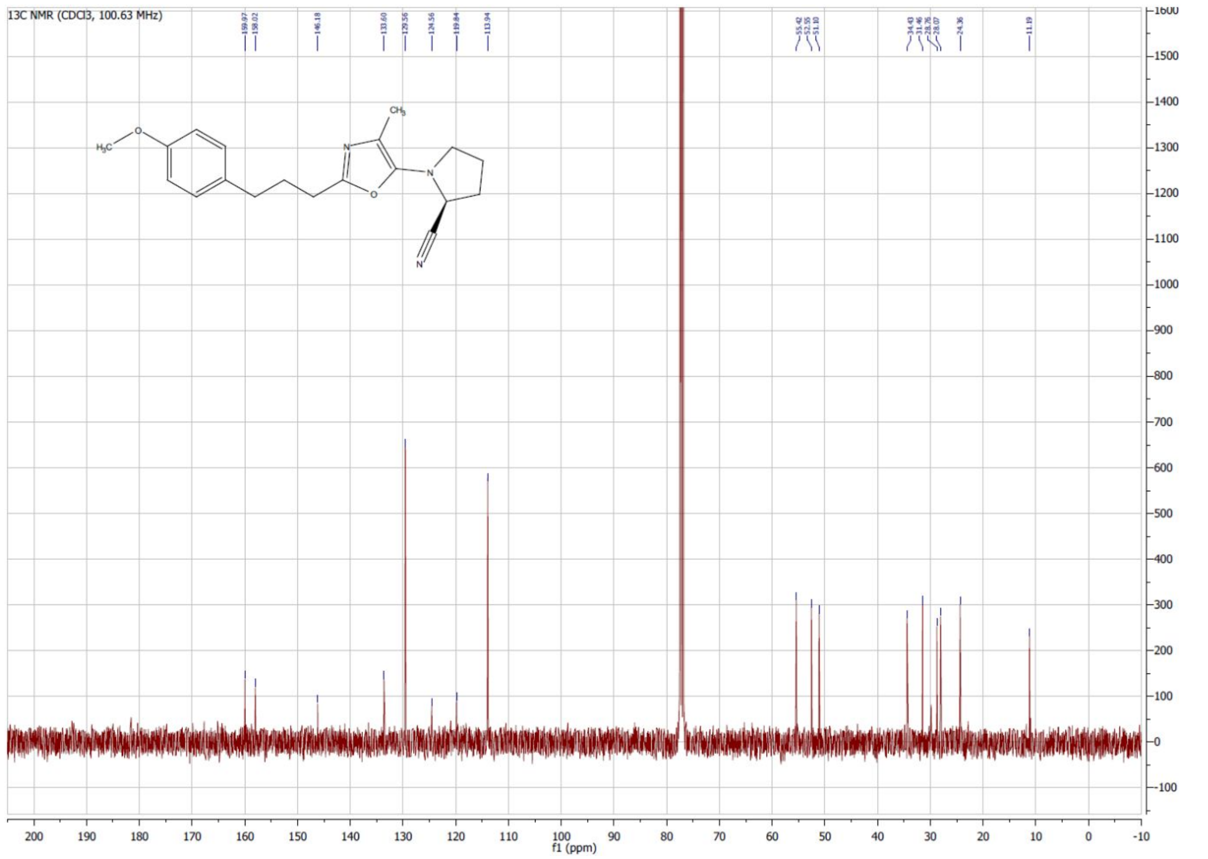

Figure S46. ^{1}H and ^{13}C NMR spectra of compound **18**.

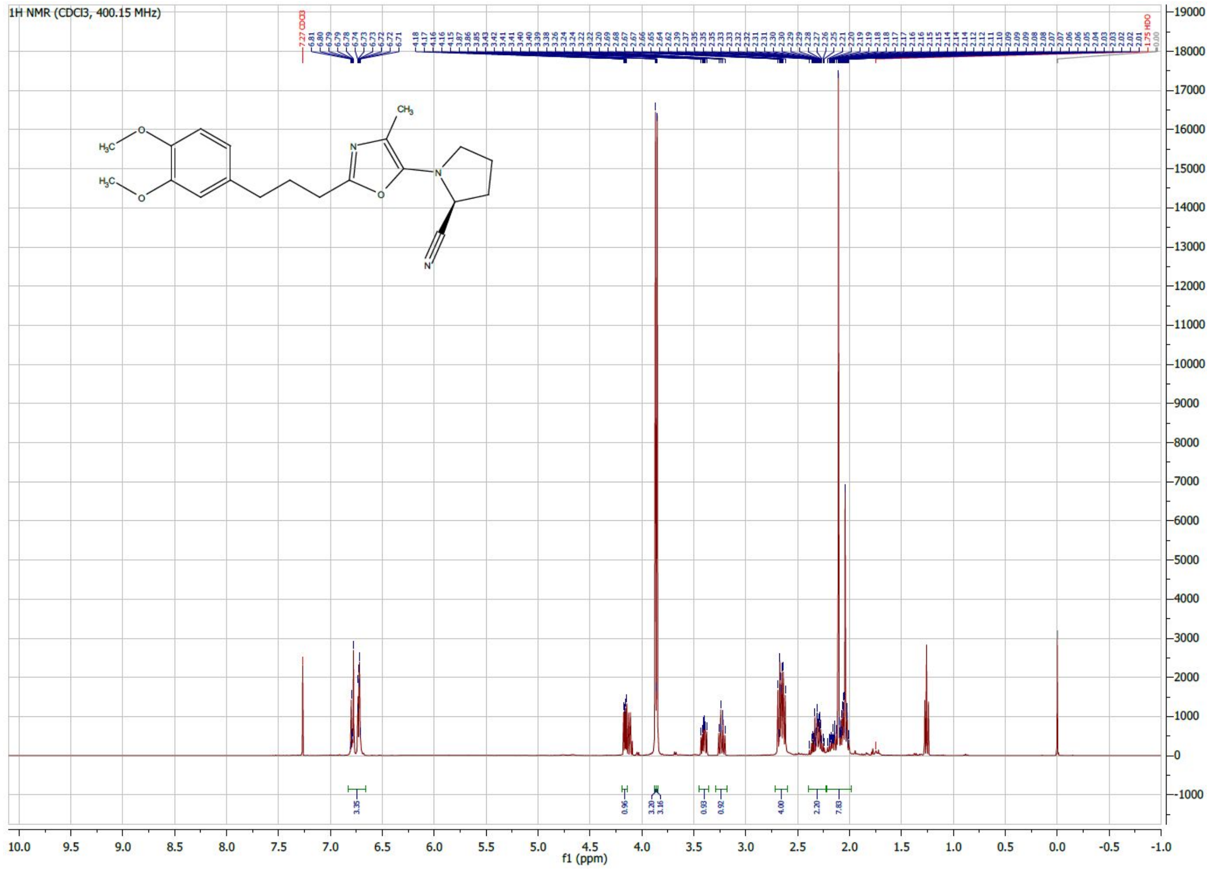

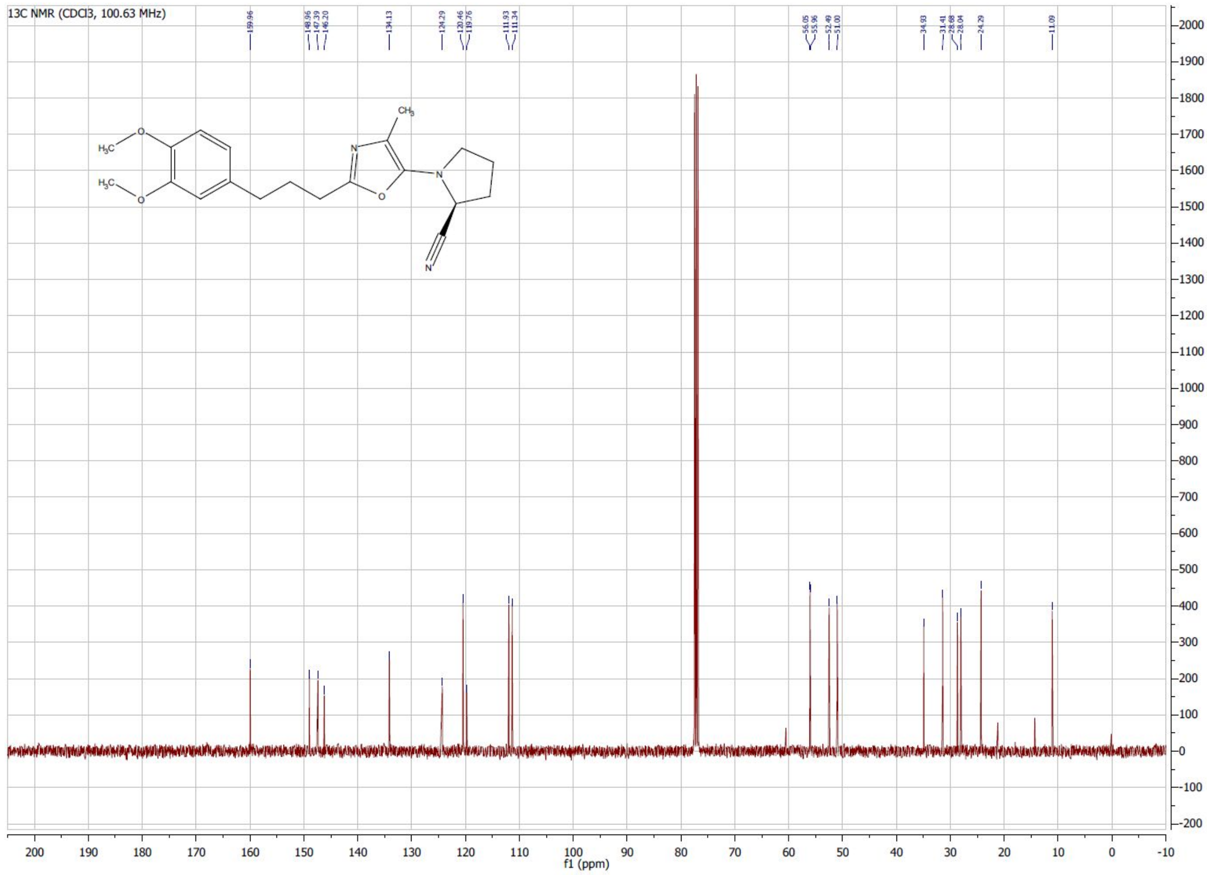

Figure S47. ^{1}H and ^{13}C NMR spectra of compound **19**.

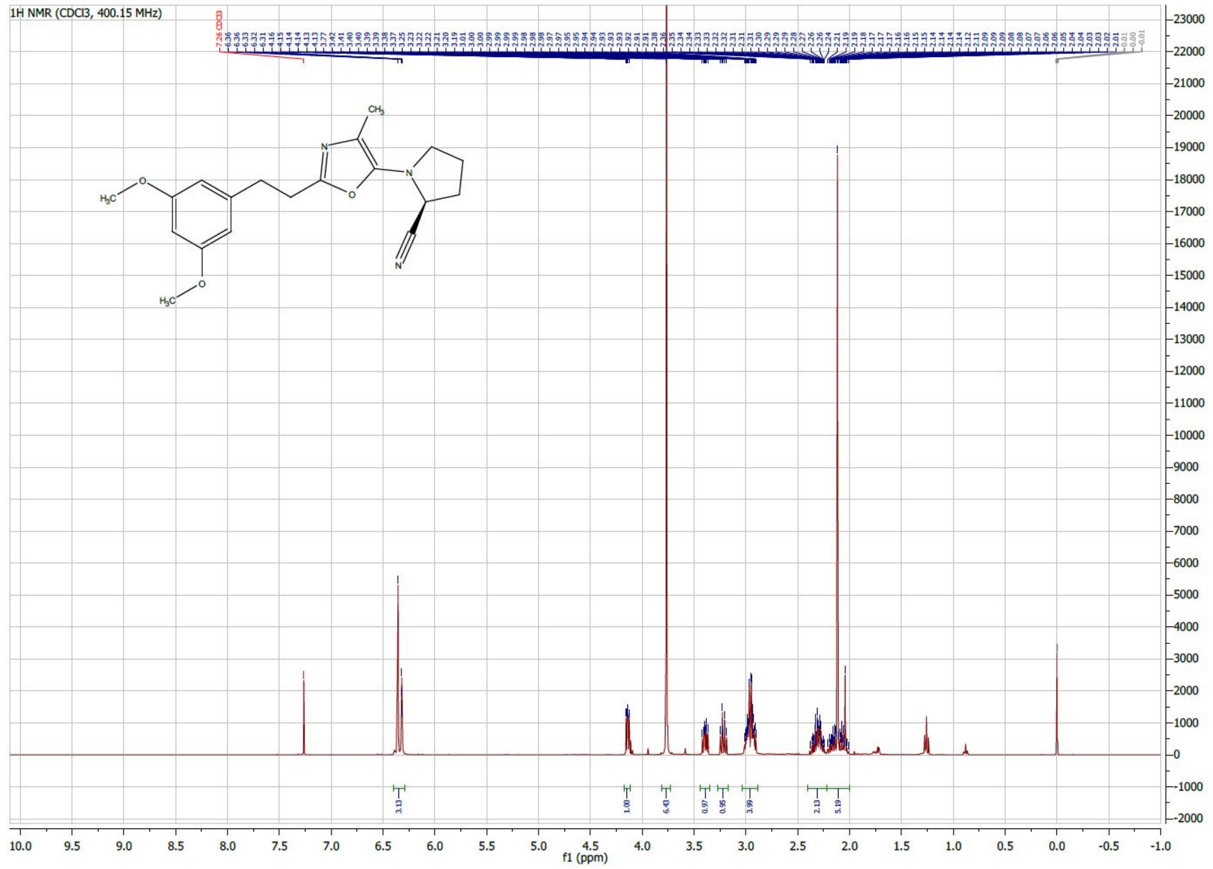

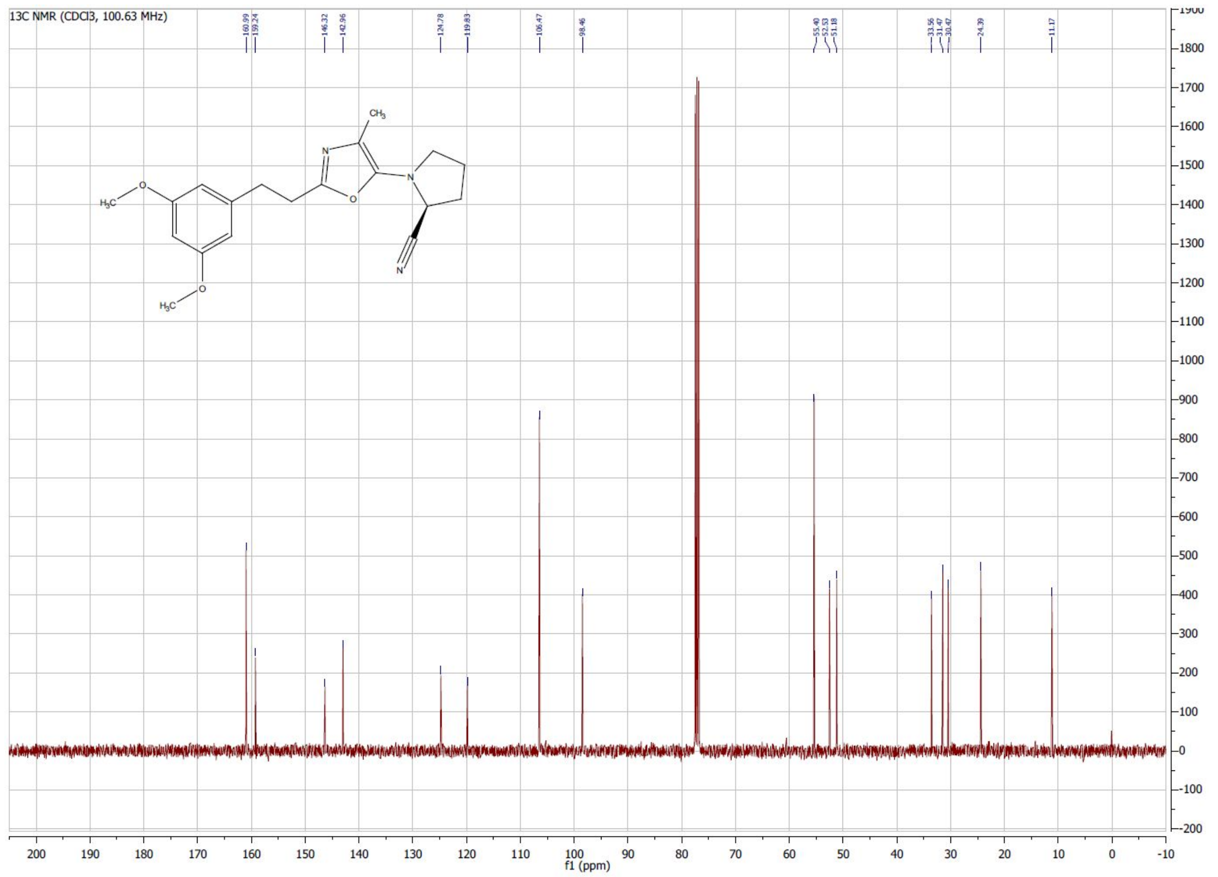

Figure S48. ^{1}H and ^{13}C NMR spectra of compound **20**.

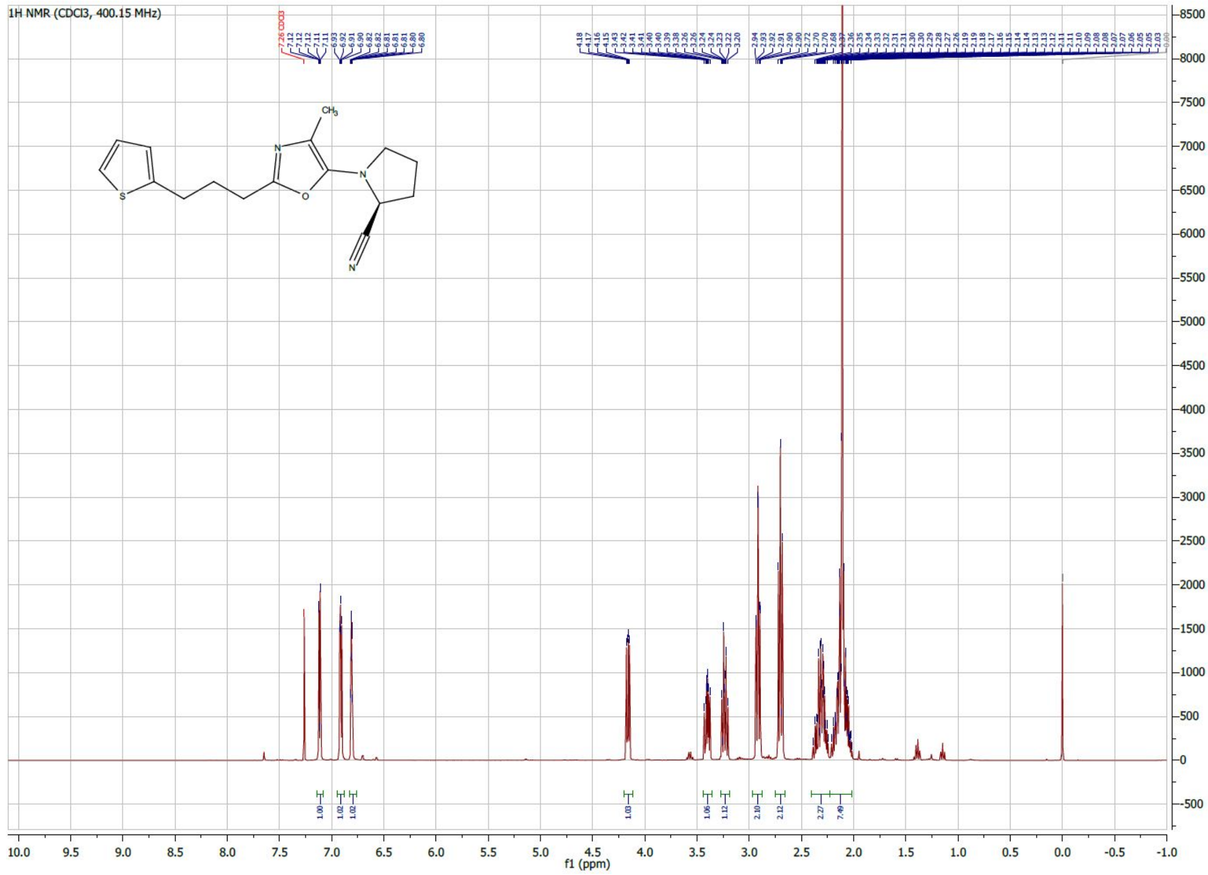

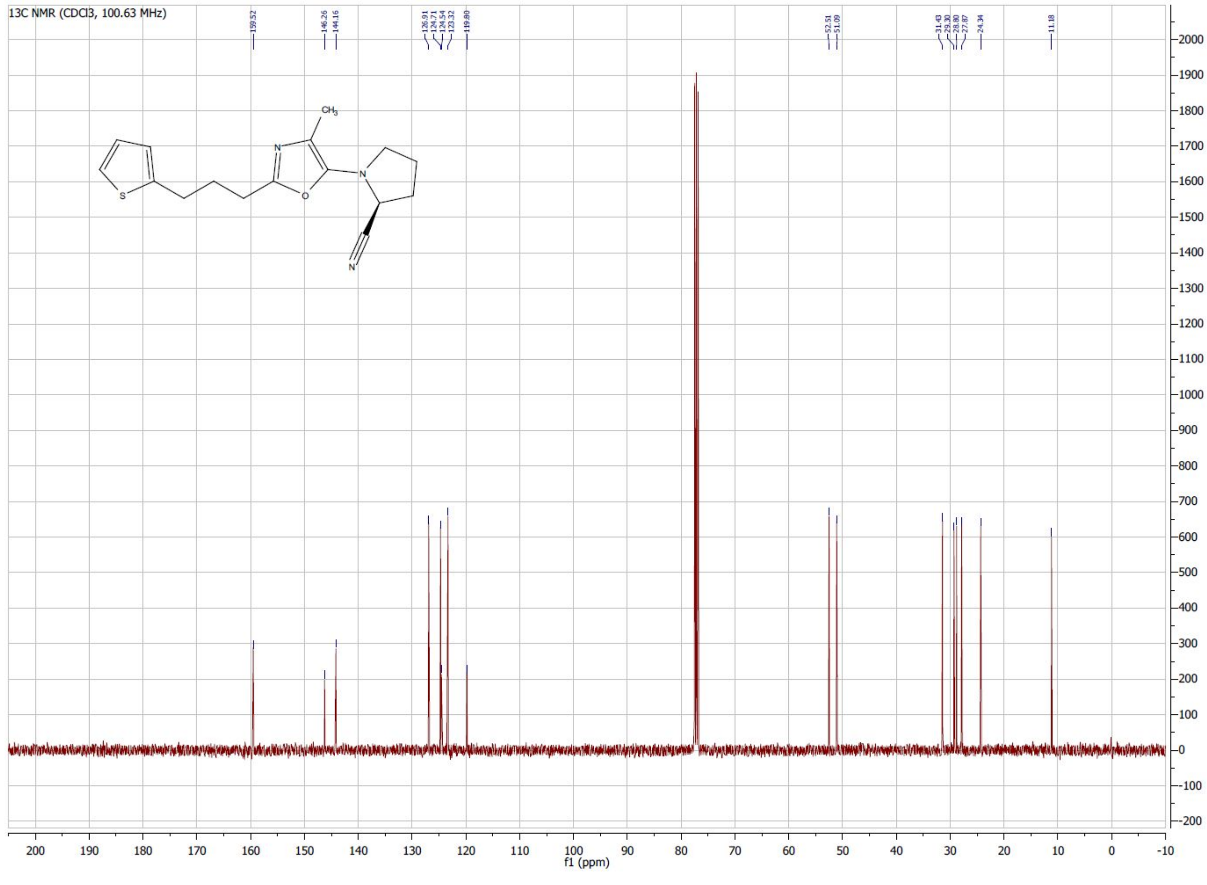

Figure S49. ^{1}H and ^{13}C NMR spectra of compound **21**.

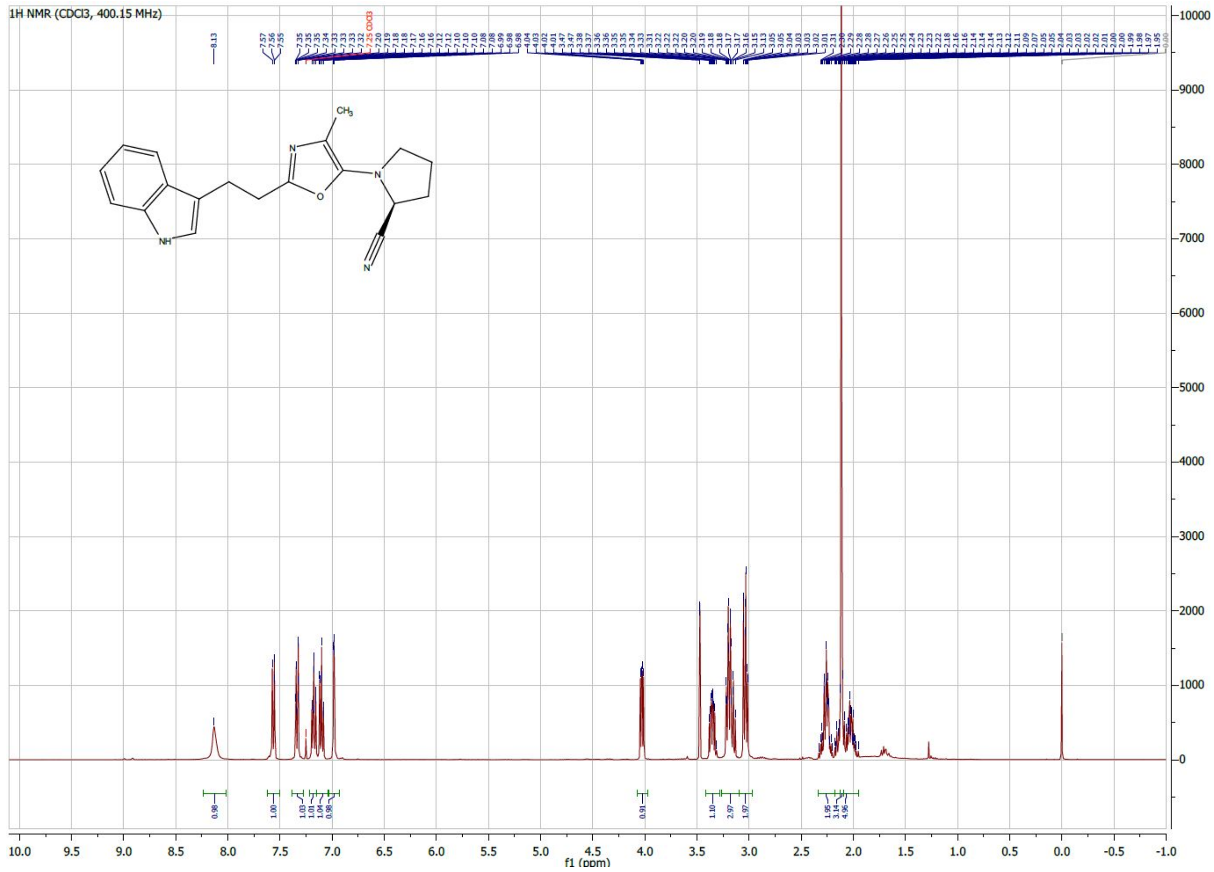

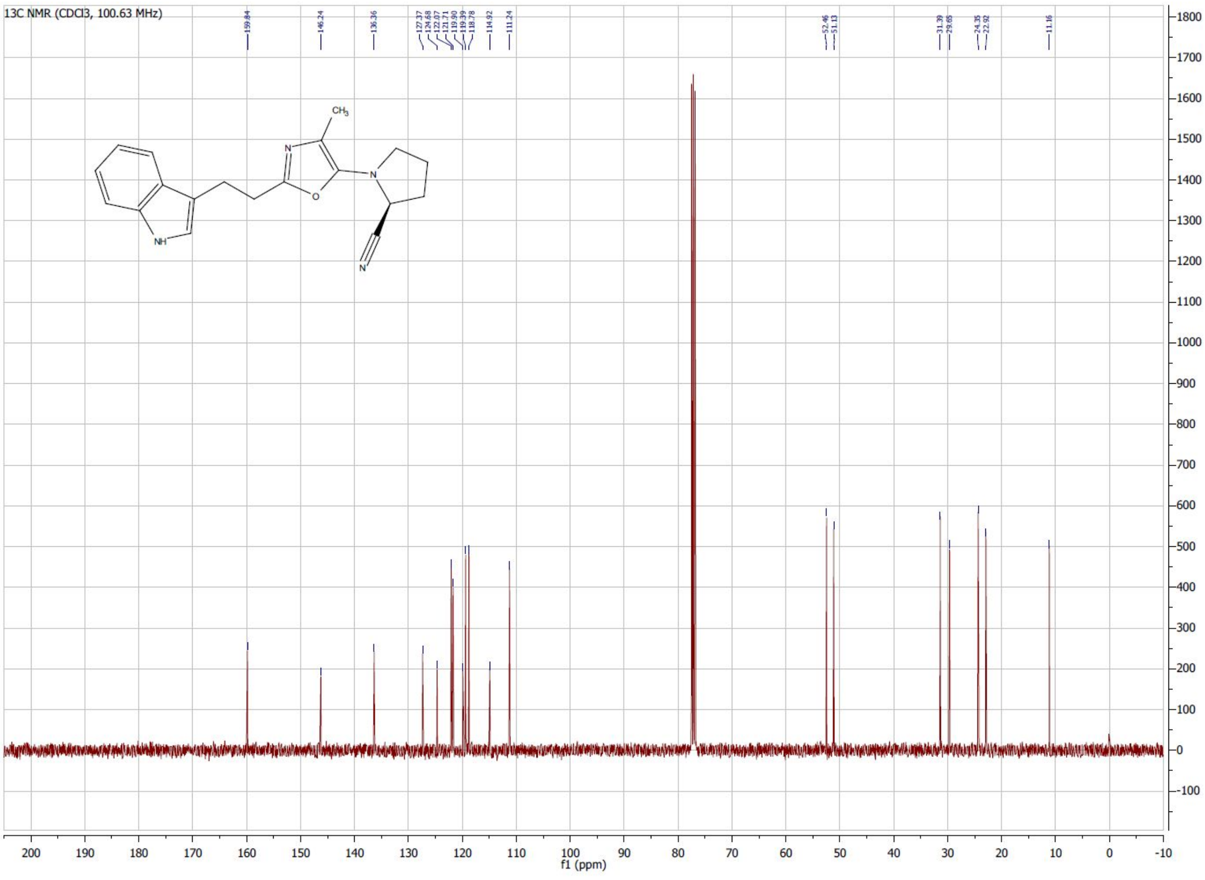

Figure S50. ^{1}H and ^{13}C NMR spectra of compound **22**.

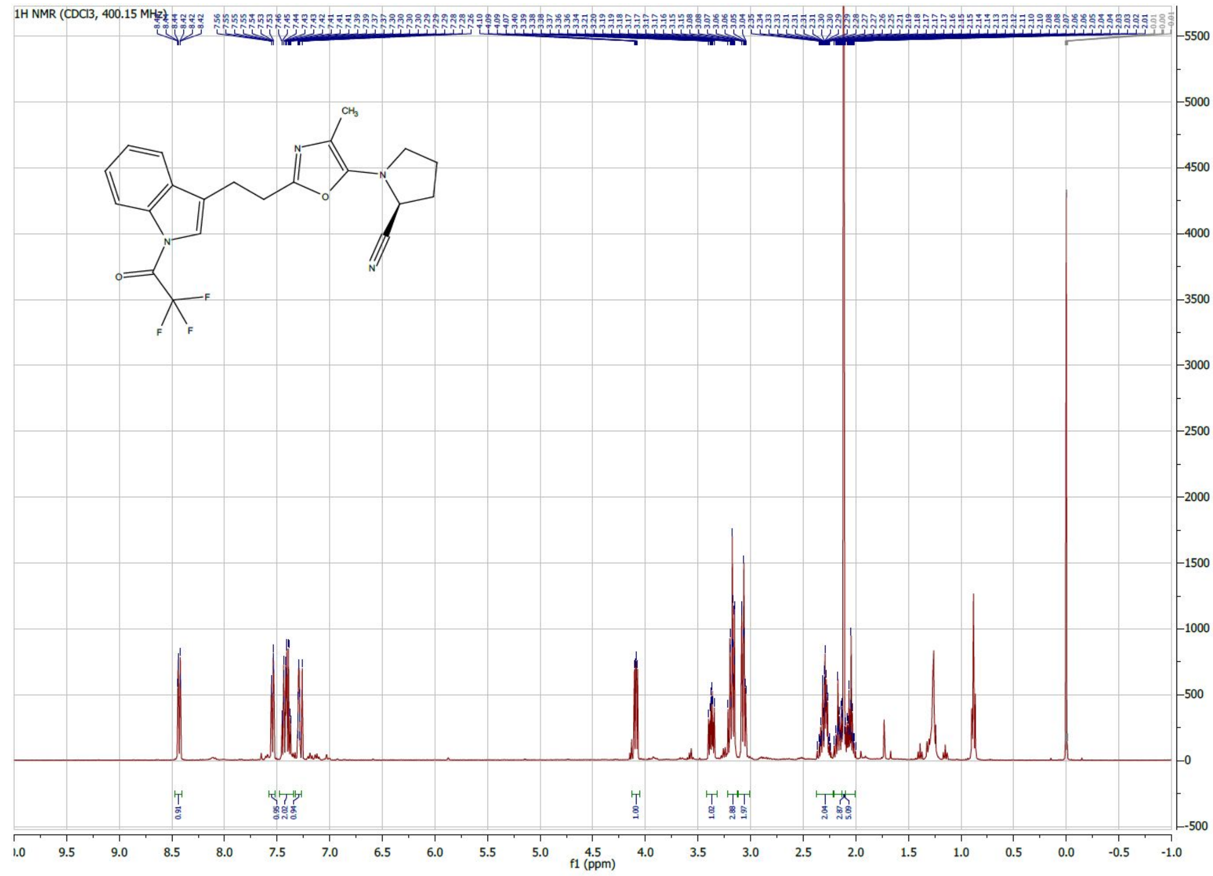

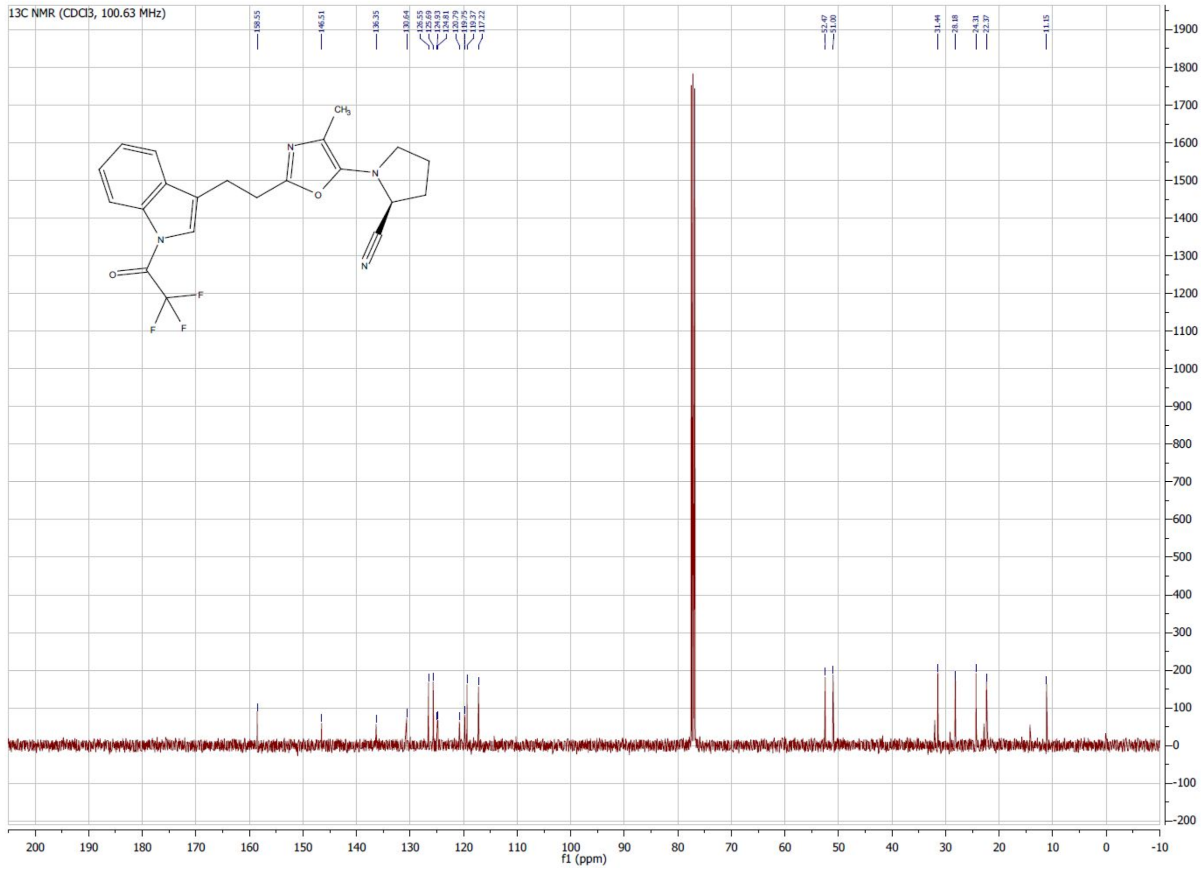

Figure S51. ^{1}H and ^{13}C NMR spectra of compound **23**.

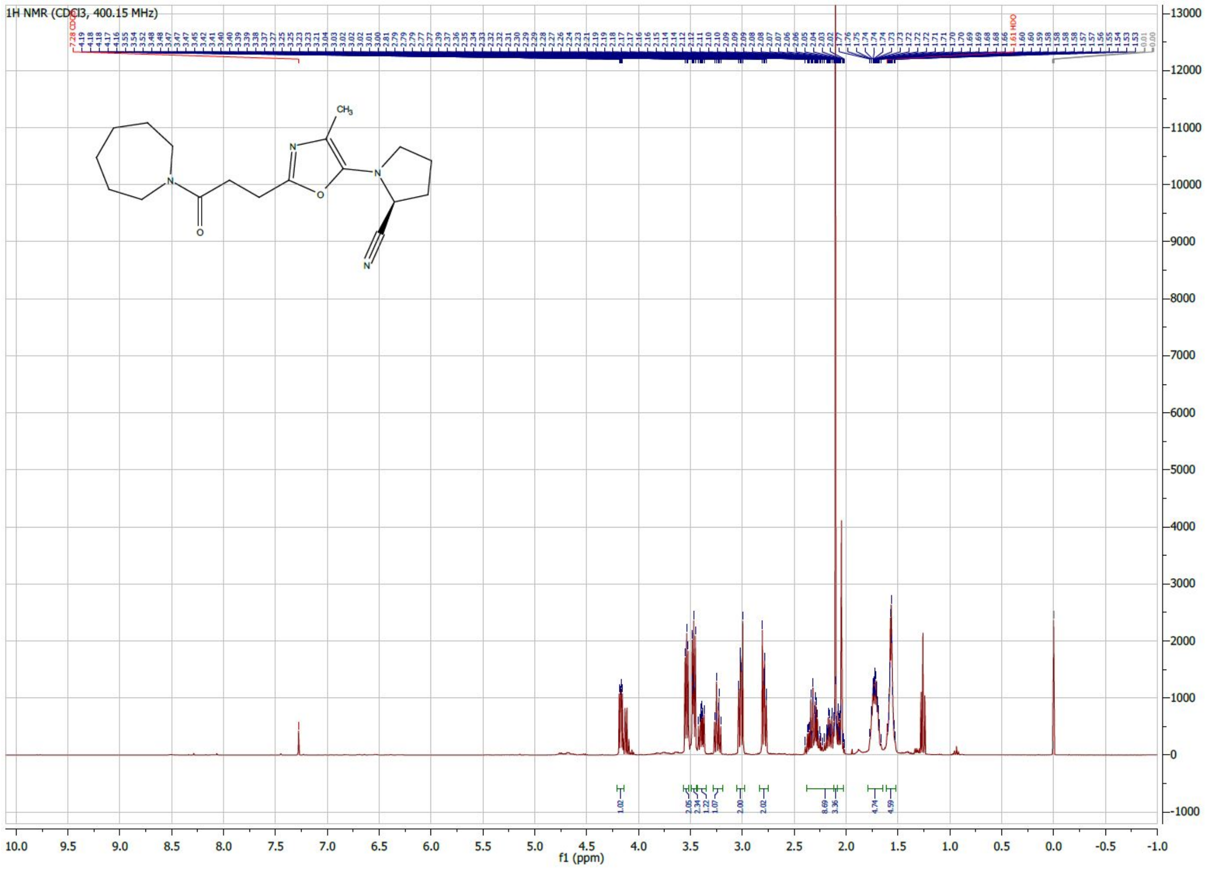

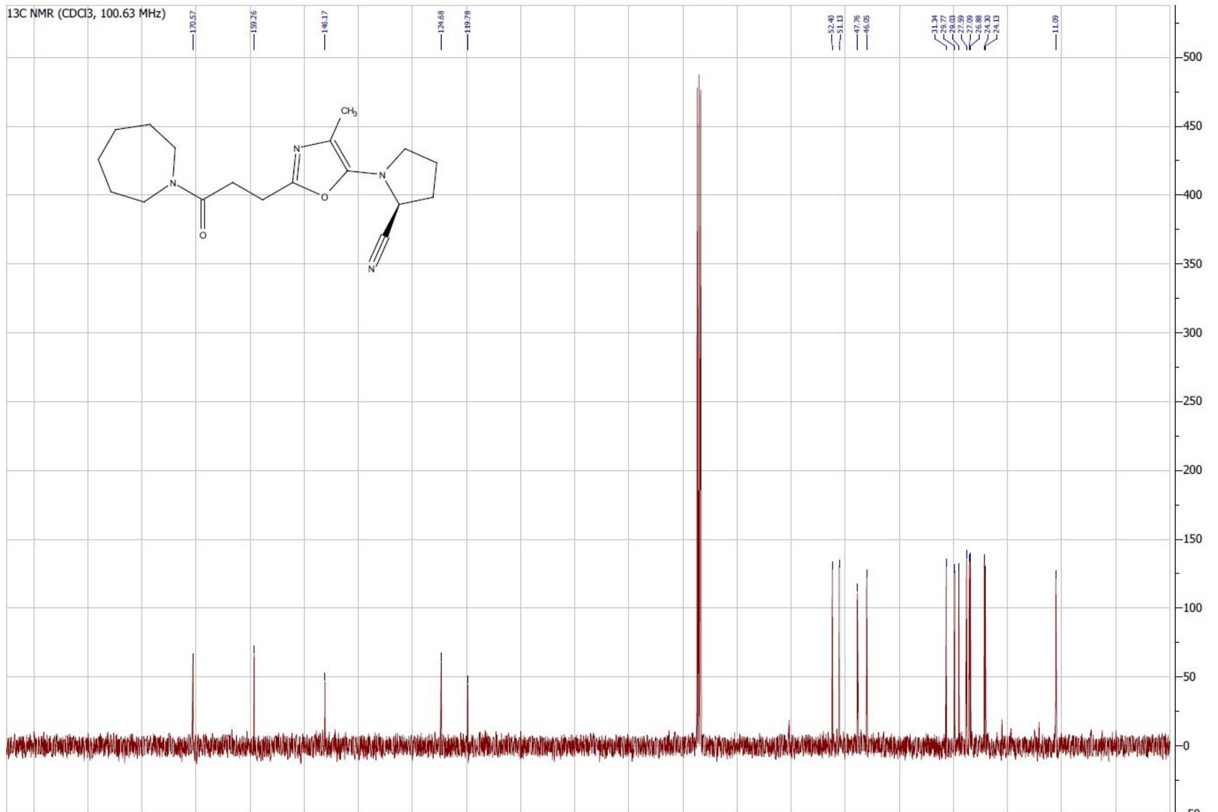

Figure S52. ^{1}H and ^{13}C NMR spectra of compound **24**.

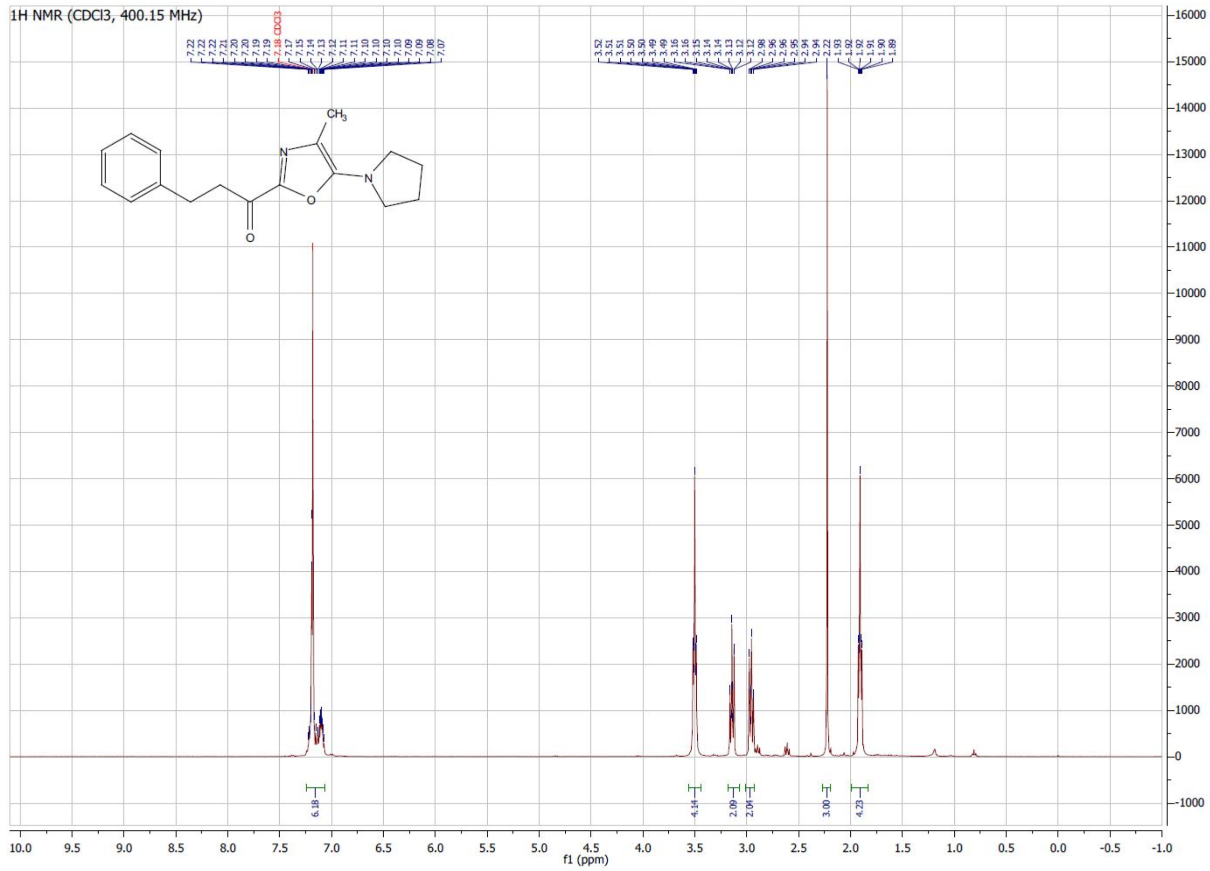

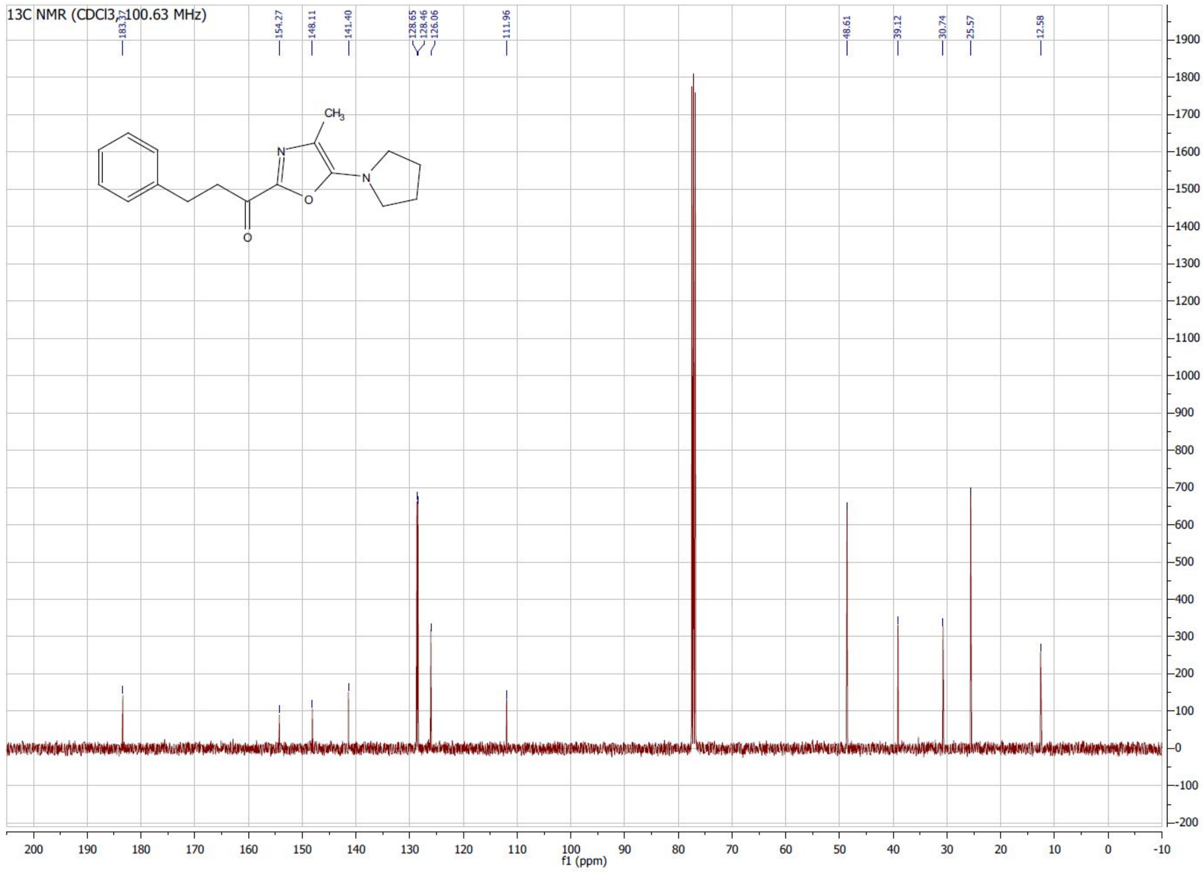

Figure S53. ^{1}H and ^{13}C NMR spectra of compound **25**.

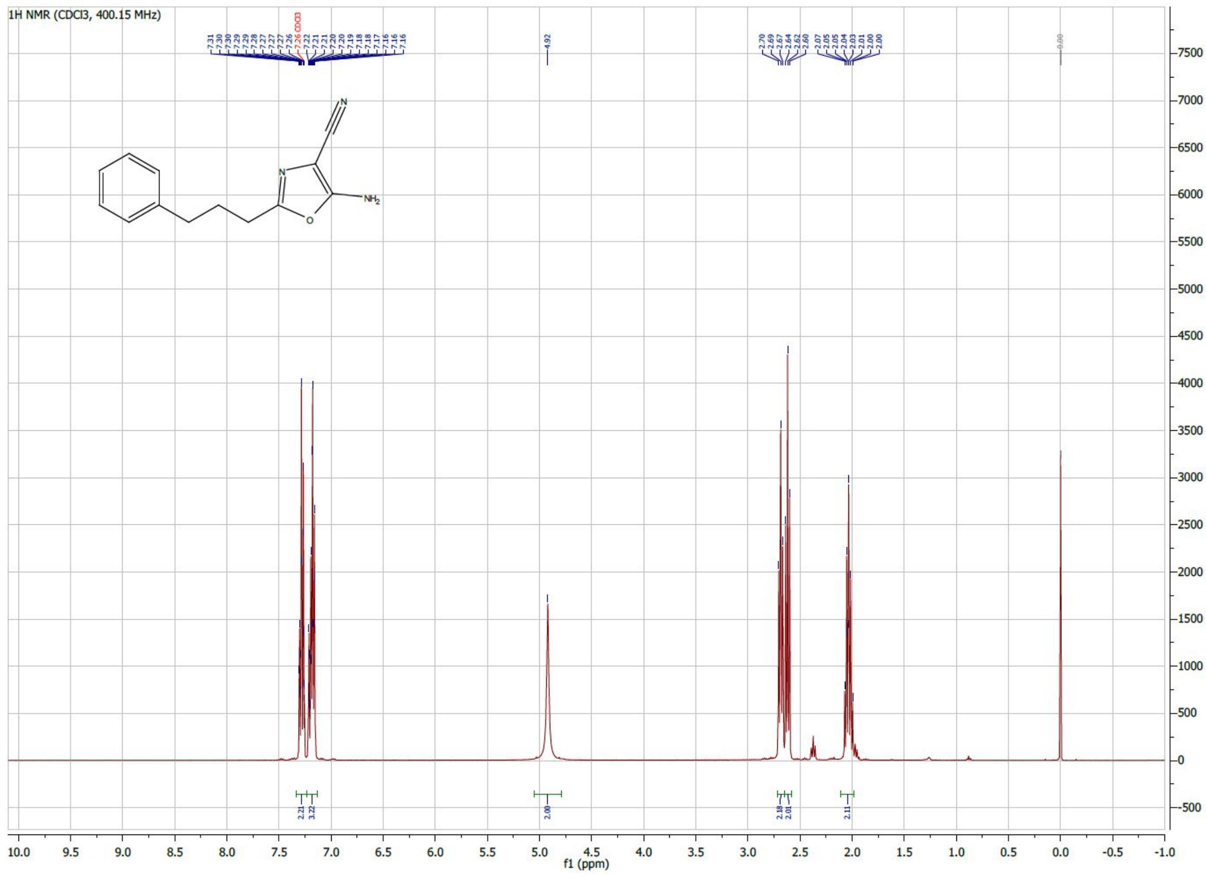

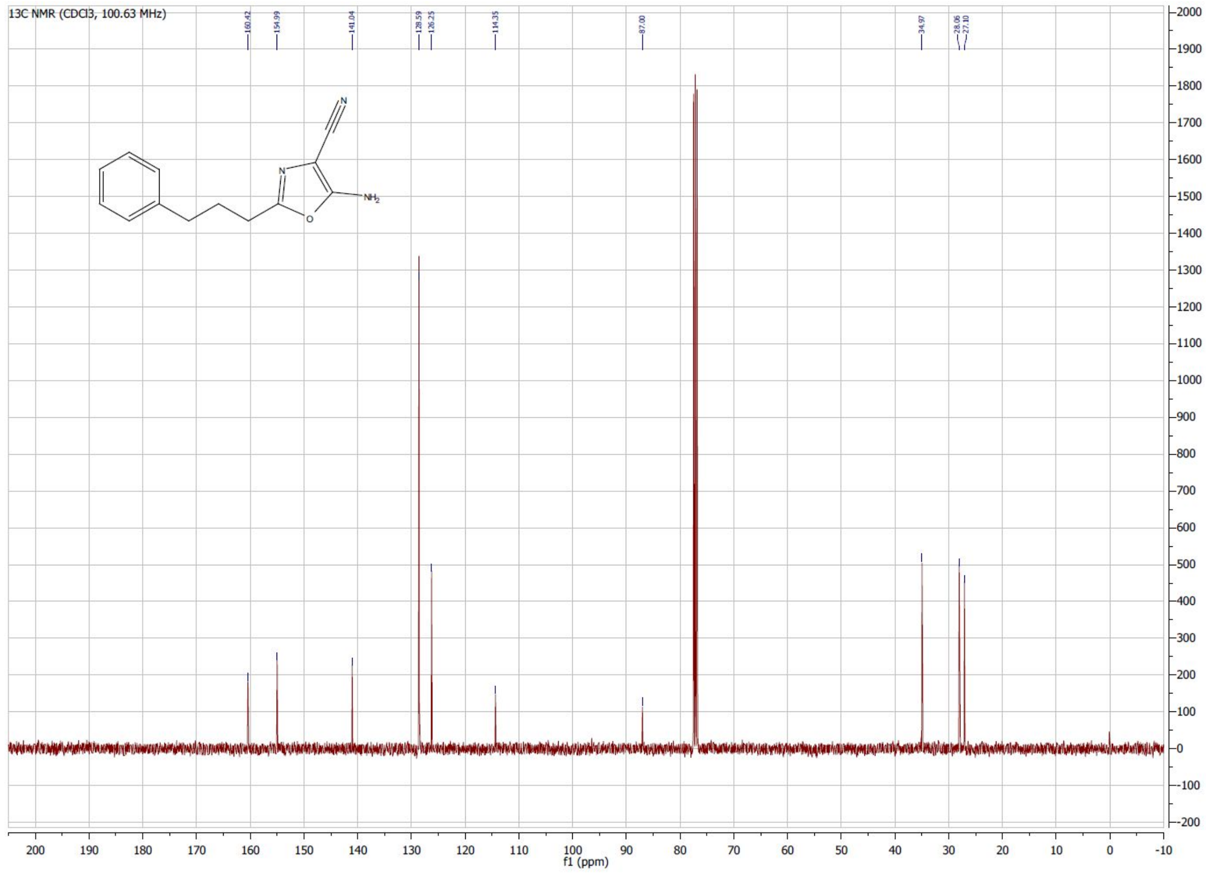

Figure S54. ^{1}H and ^{13}C NMR spectra of compound **26**.

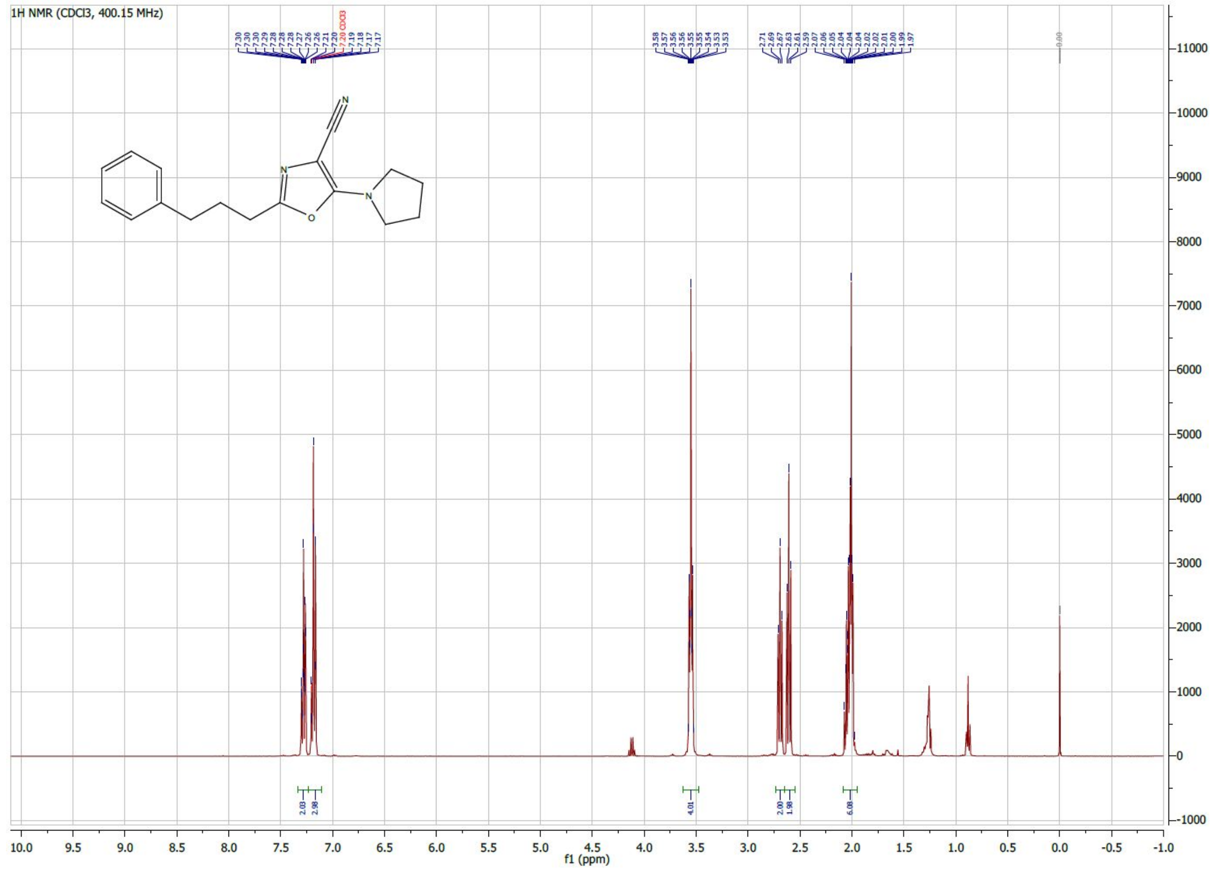

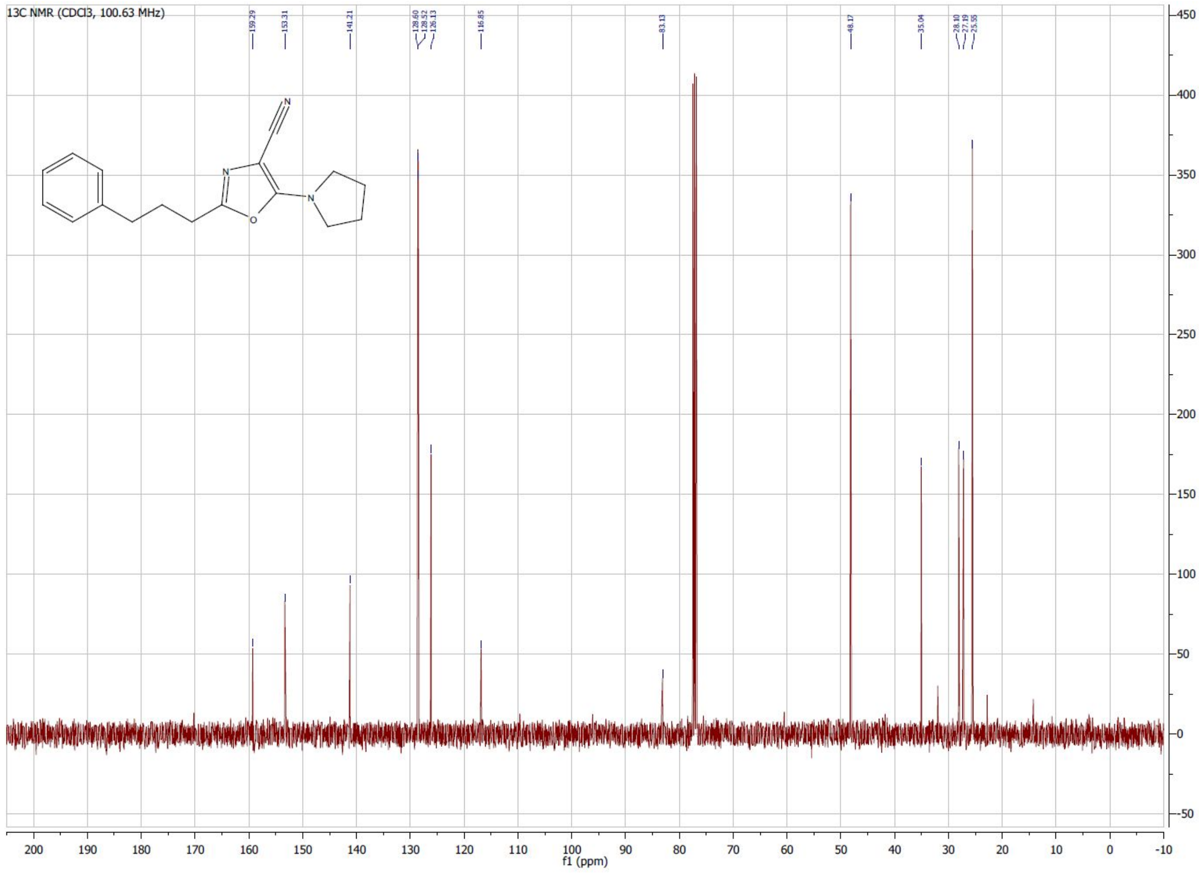

Figure S55. ^{1}H and ^{13}C NMR spectra of compound **27**.

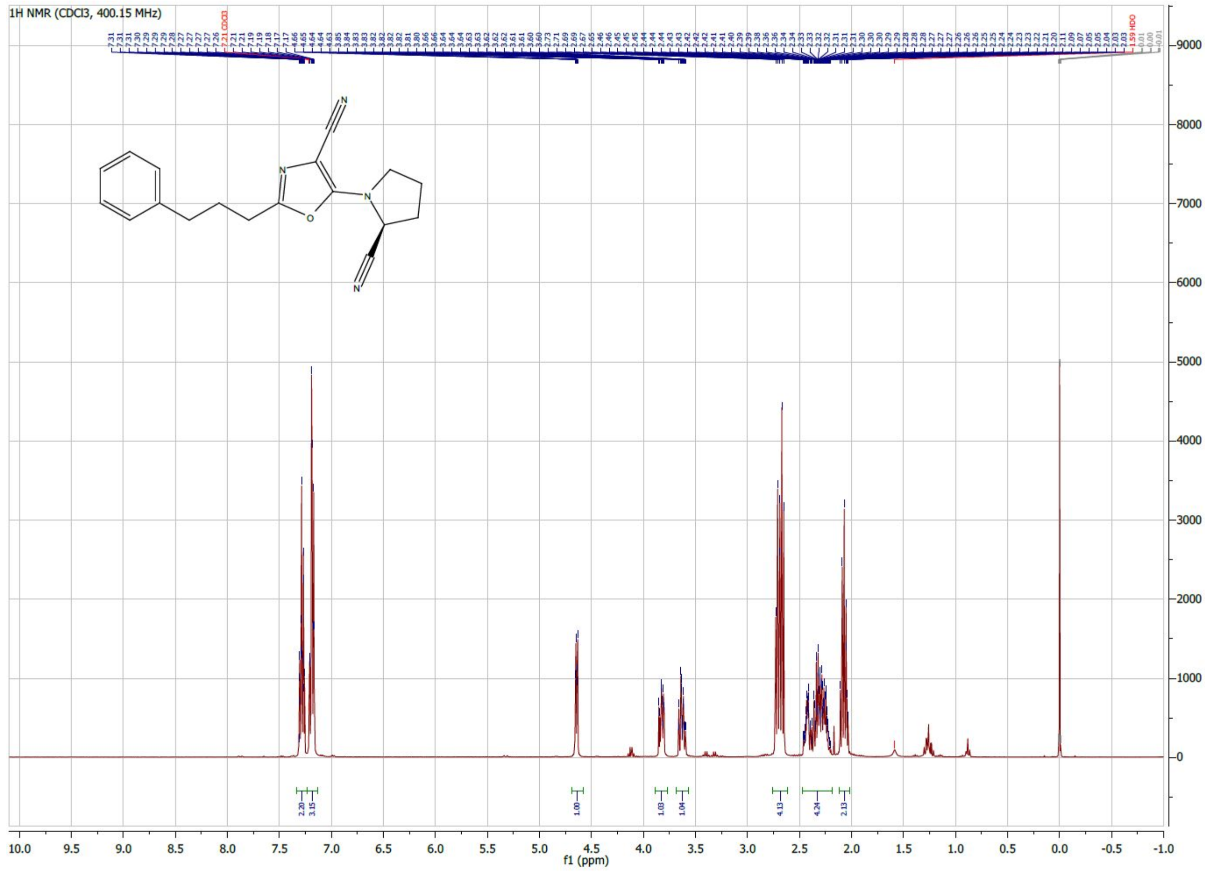

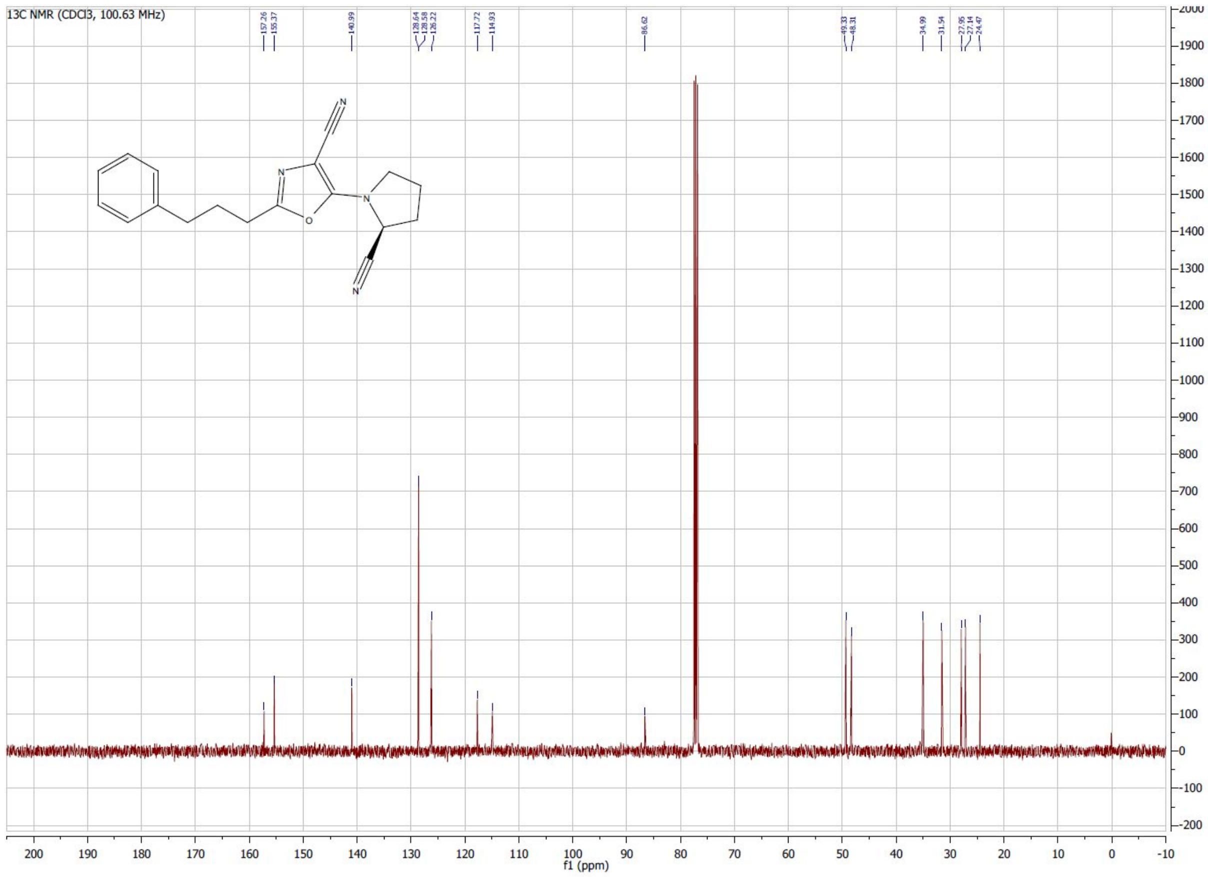

Figure S56. ^{1}H and ^{13}C NMR spectra of compound **28**.

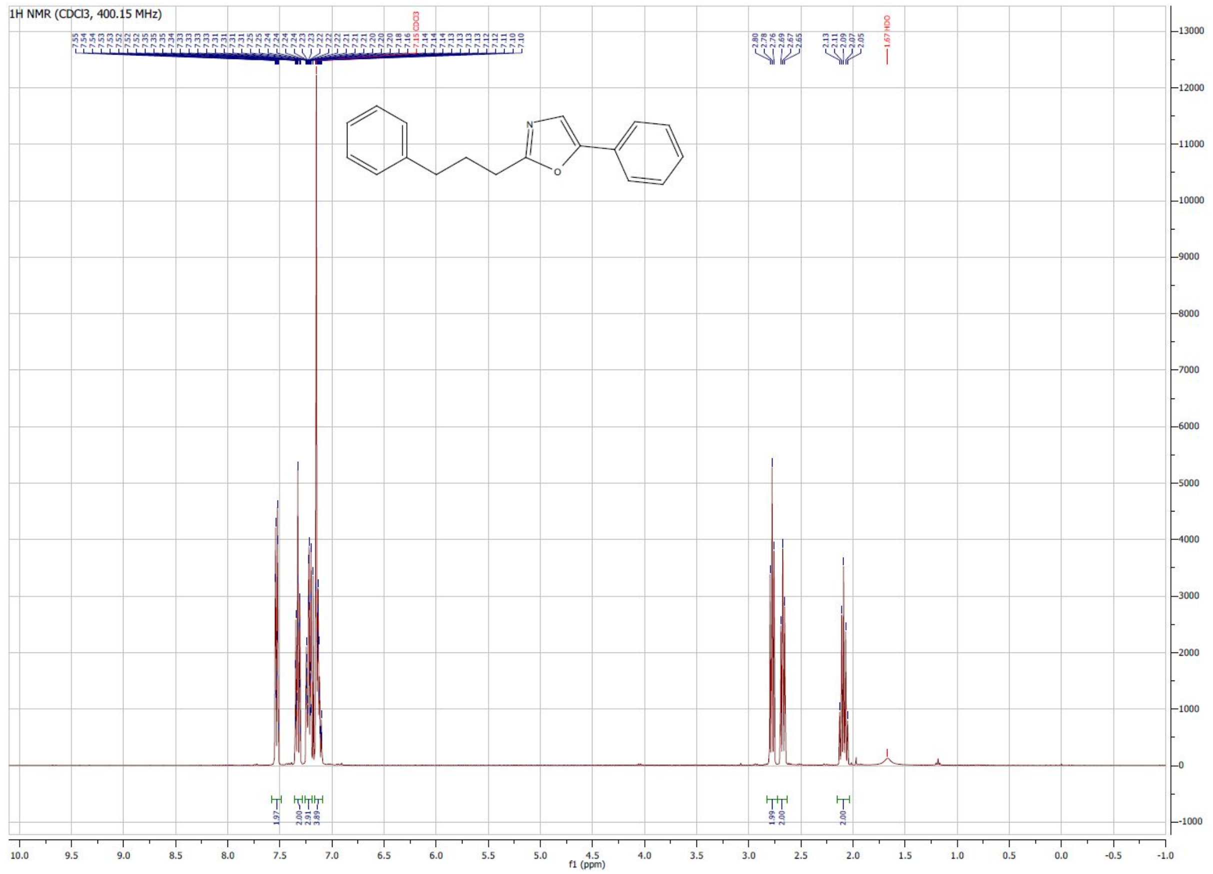

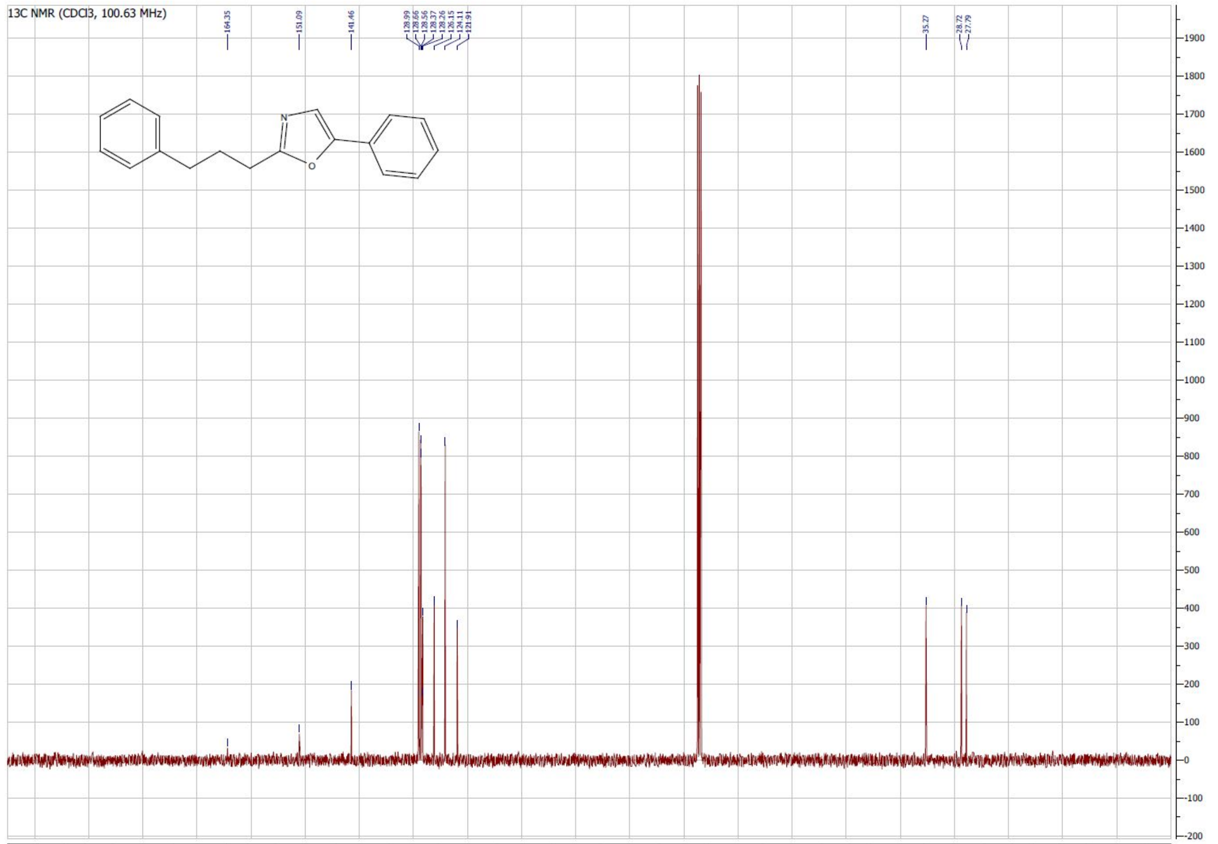

Figure S57. ^{1}H and ^{13}C NMR spectra of compound **29**.

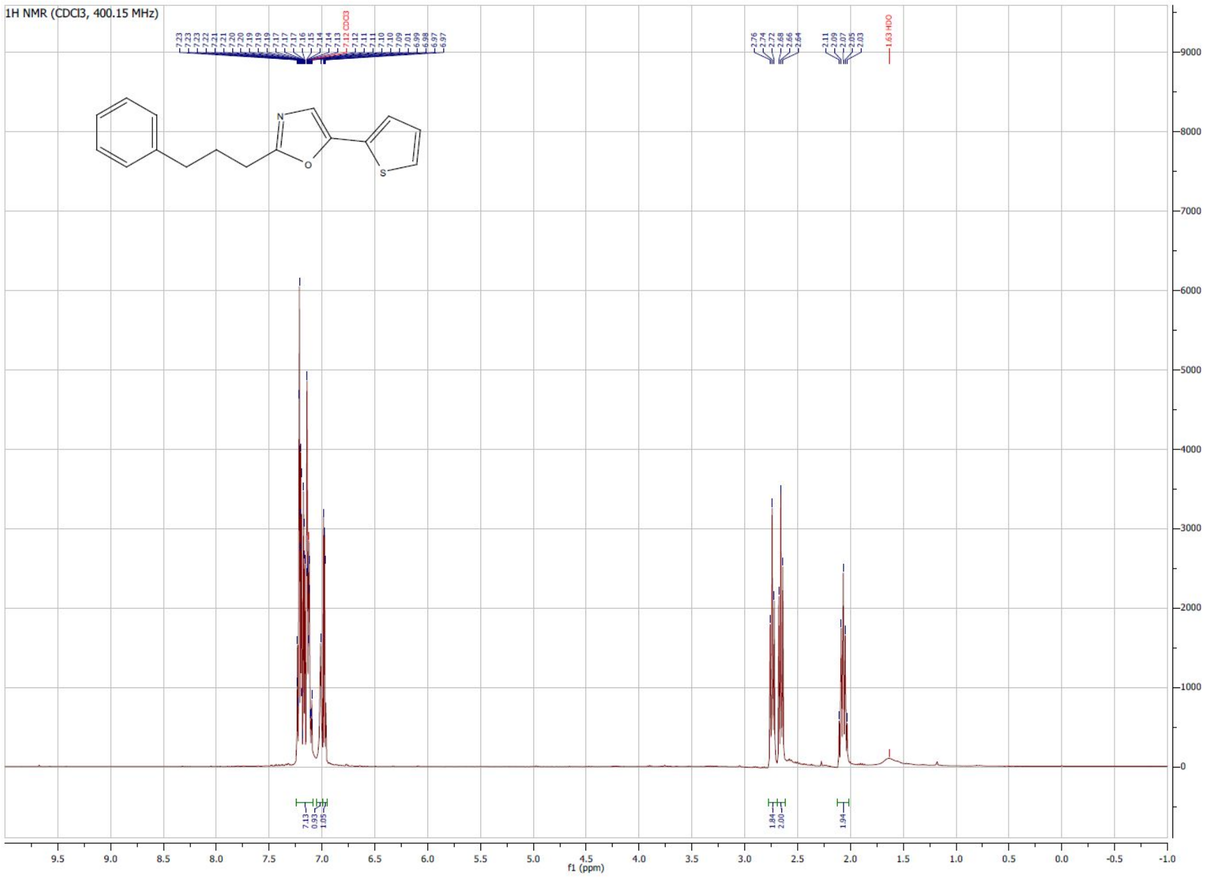

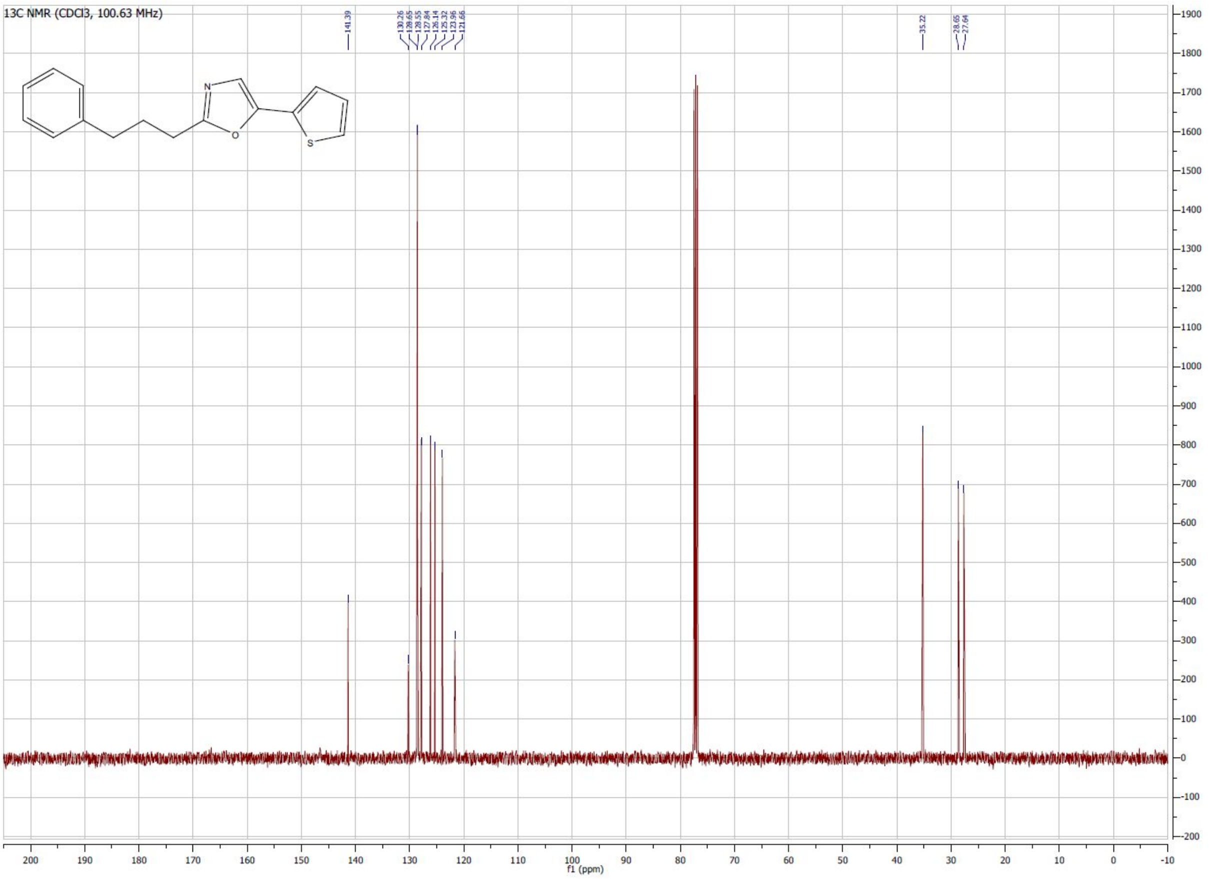

Figure S58. ^{1}H and ^{13}C NMR spectra of compound **30**.

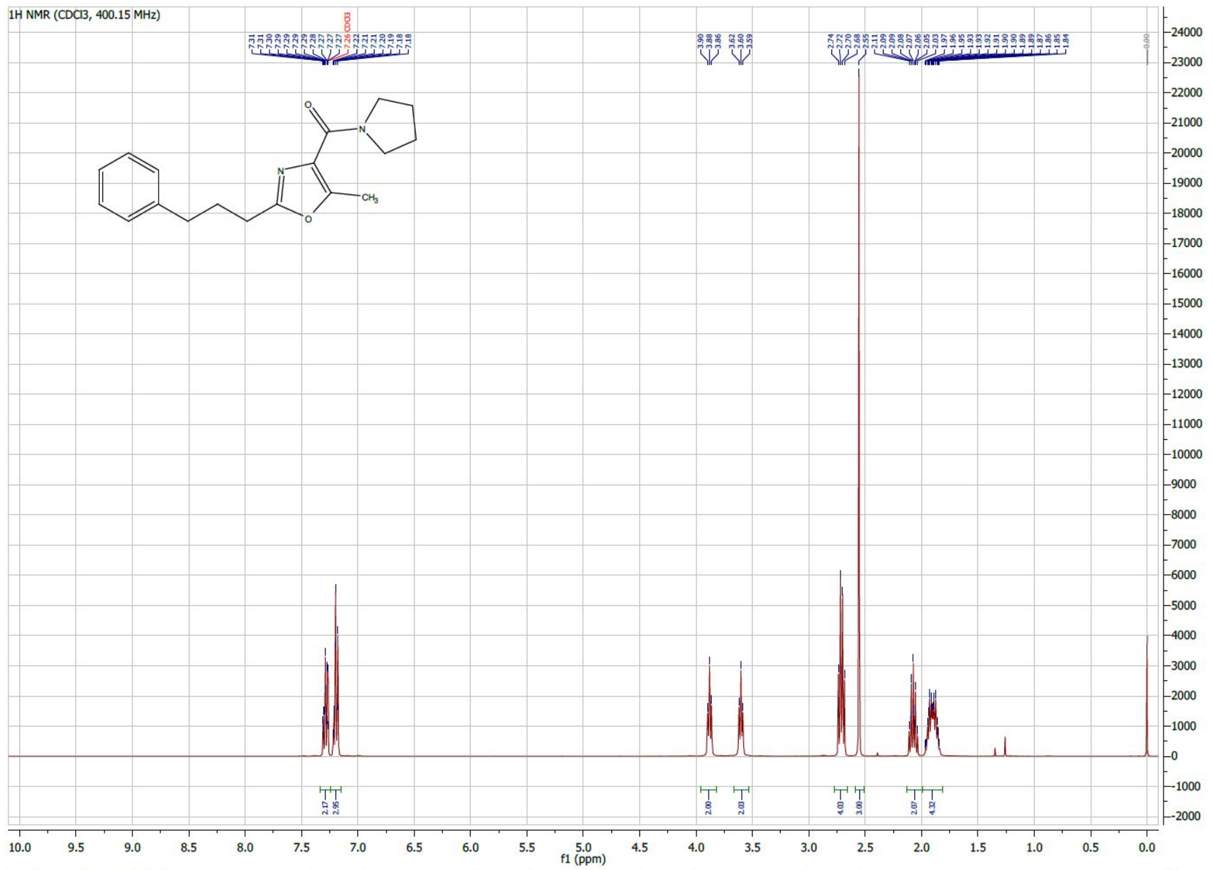

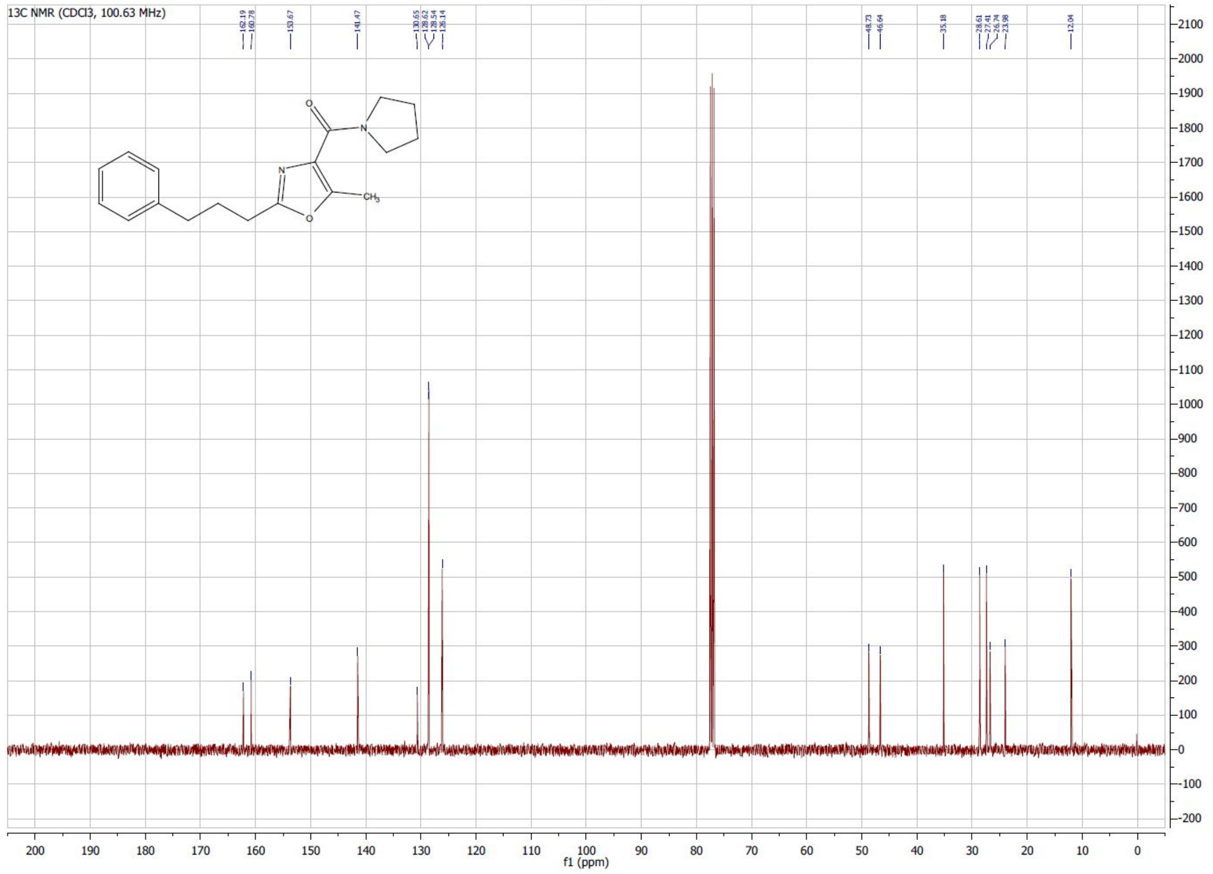

Figure S59. ^{1}H and ^{13}C NMR spectra of compound **31**.

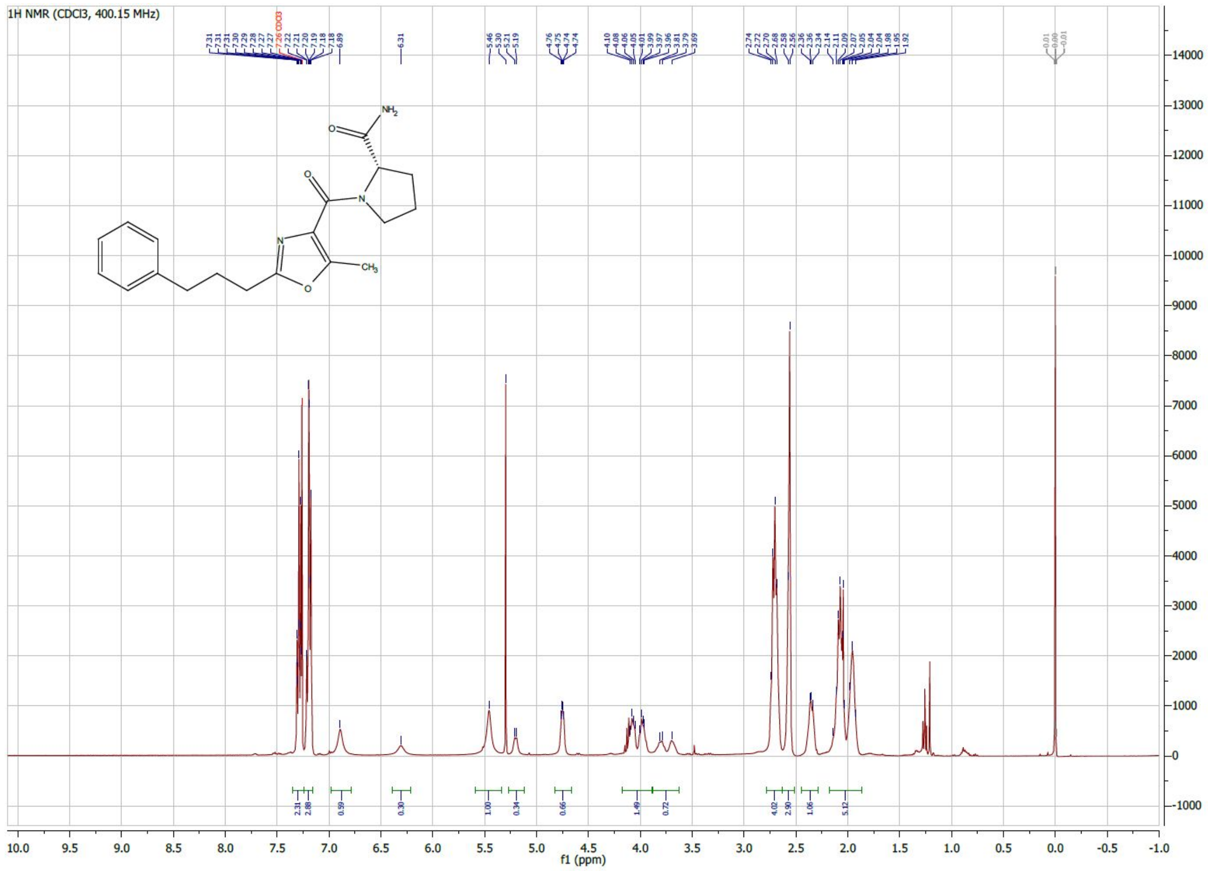

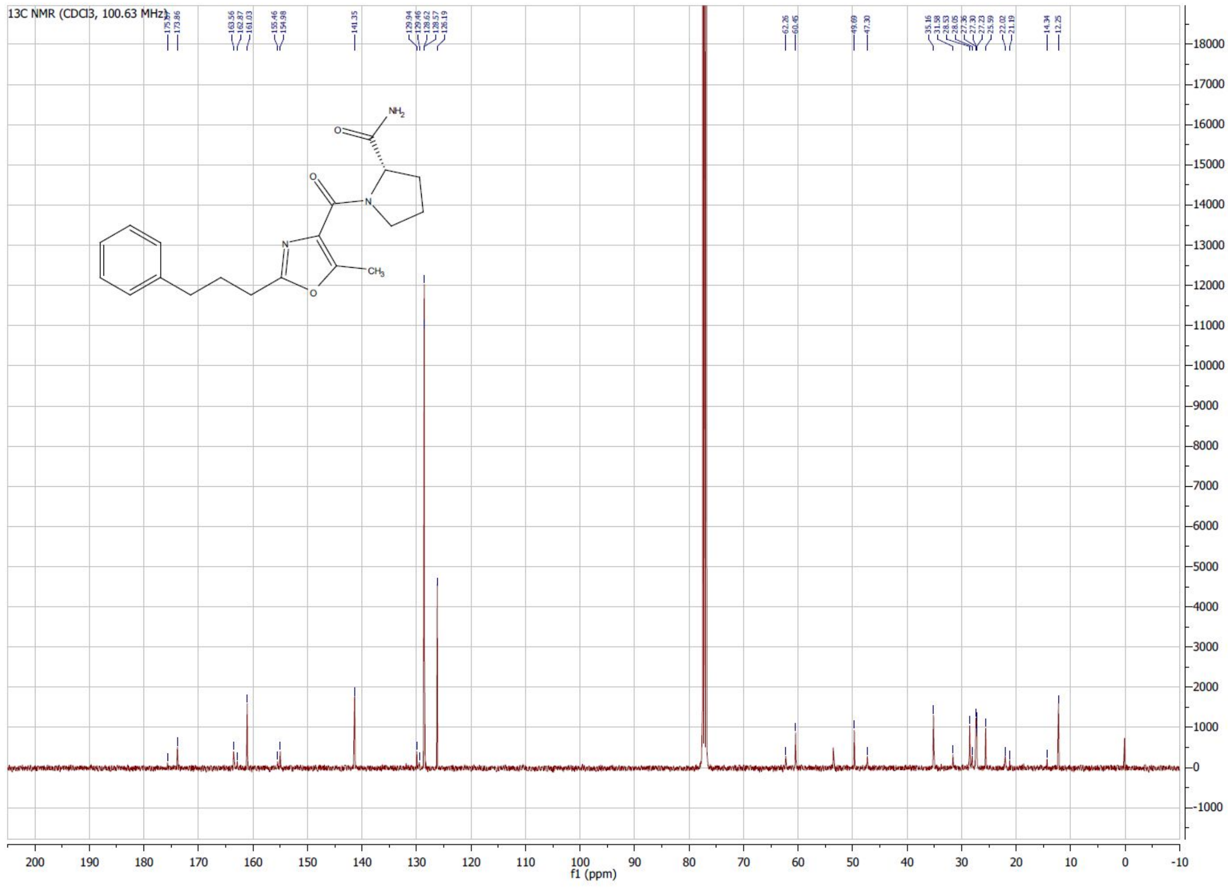

Figure S60. ^{1}H and ^{13}C NMR spectra of compound **32**.

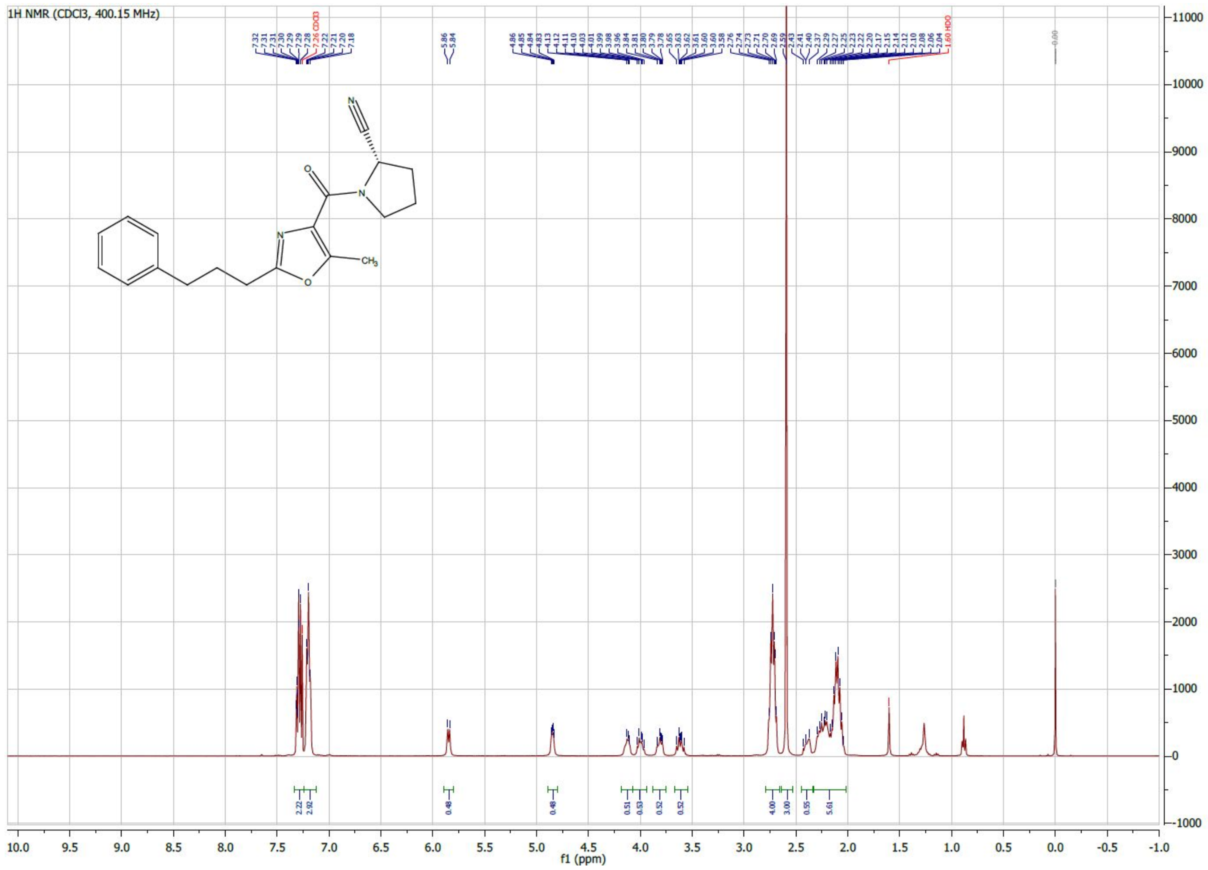

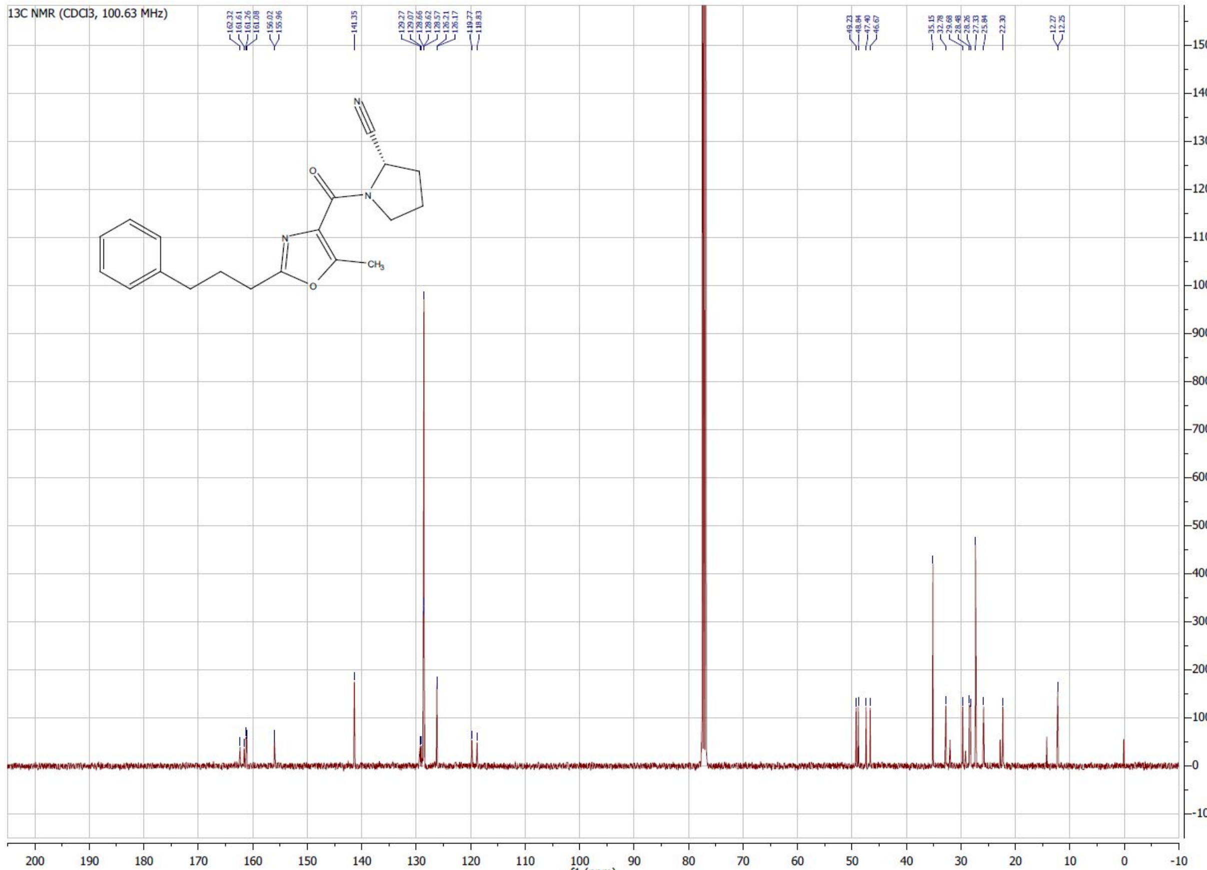

Figure S61. ^{1}H and ^{13}C NMR spectra of compound **33**.

Monitoring stability

Stability was monitored from NMR samples of the compounds in solution, typically in $CDCl_3$ if not otherwise noted, by repeating measurements after storing the samples for 2-4 days at room temperature. Purity analysis using UPLC-MS was, by default, run two or more weeks after isolating the compounds, confirming long-term stability. In this way, we ensured that no compounds with even slight stability issues entered biological assays. These experiments revealed that a methyl ester group as a replacement of the nitrile group in compound **68** does not result in the same stabilizing effect. Furthermore, in compounds **69** and **70**, we noticed that the copresence of a nucleophilic pyridine ring and an electrophilic nitrile group in the same molecule lowered the stability. The 2,5-diaminooxazoles, compounds **66** and **67**, also have limited stability as the isolated derivatives with an attached *N*-trifluoroacetyl group are slightly labile and those without it could not be isolated in the reaction. All isolated compounds with problems in long-term stability are shown in Figure S62. These were not tested in the biological assays.

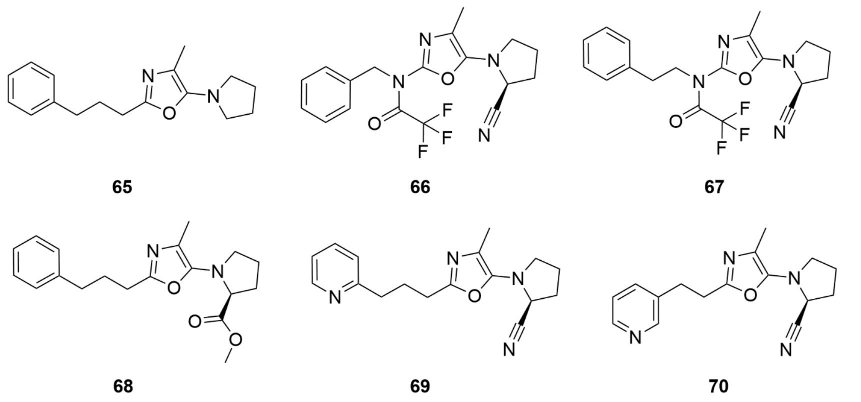

Figure S62. Compounds that were isolated but not evaluated in the biological assays due to problems in long-term stability.

HUP-55 NMR stability

A 50 mM NMR sample of **HUP-55** in DMSO-d_6 was observed over 9 weeks. For the first 4 weeks, the sample was kept refrigerated and NMR measurements were taken after 1 week and 4 weeks. For the next 4 weeks, the sample was kept at room temperature and measurements were taken 1 week, 2 weeks, and 4 weeks after moving to room temperature. Finally, the sample was diluted to 25 mM with D_2O and measurements were taken after 1 day and 1 week. The results are shown in Figure S63.

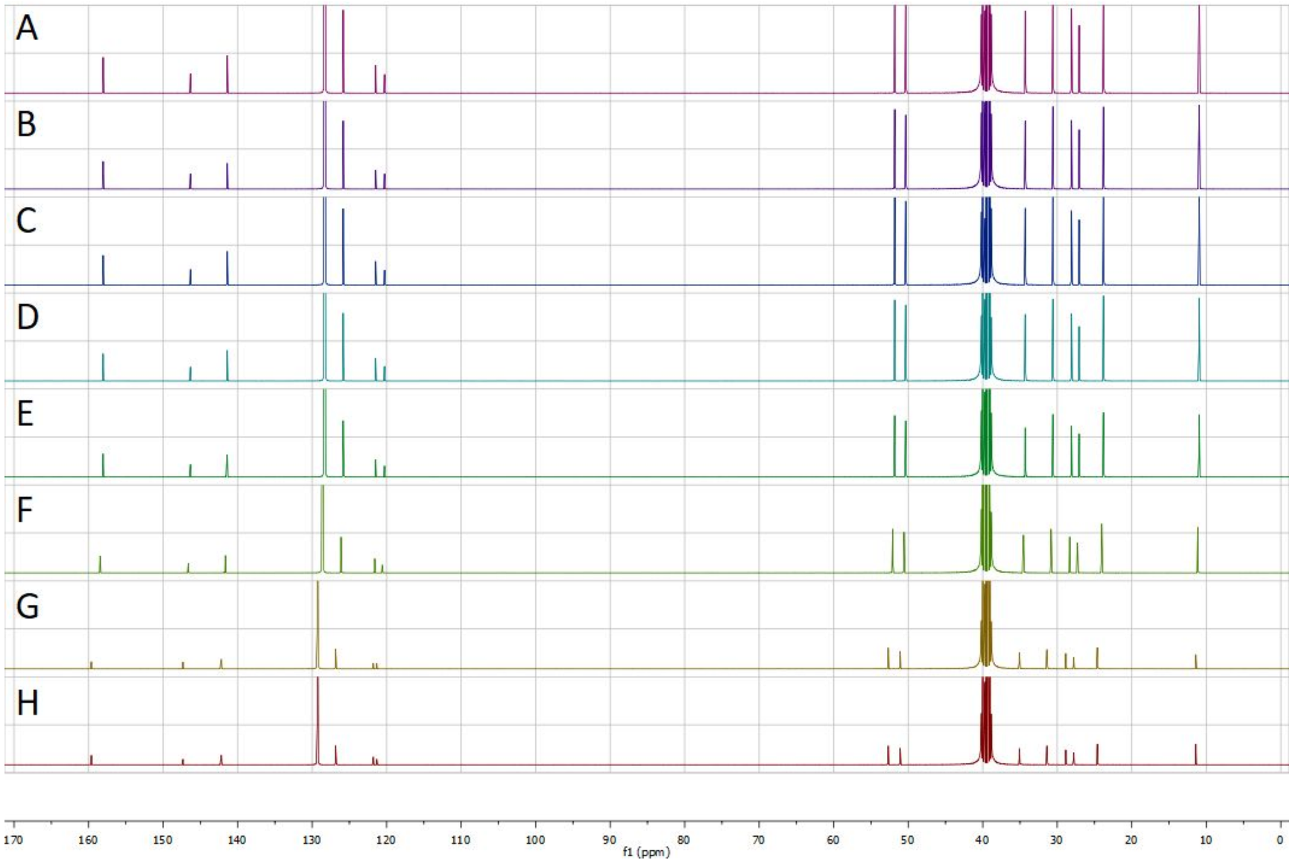

Figure S63. ^{13}C NMR spectra of the **HUP-55** NMR stability study generated using the MestReNova[1] stack function. The sample was prepared in DMSO-d_6 with a concentration of 50 mM and measurements were taken (A) before storage, after storing the sample refrigerated for (B) 1 week and (C) 4 weeks, after further storing the sample at room temperature for (D) 1 week, (E) 2 weeks, and (F) 4 weeks. D_2O was then added, lowering the concentration to 25 mM, and the final measurements were taken after further storing the sample at room temperature for (G) 1 day and (H) 1 week.

HUP-55 MS stability

Samples for MS-assay were prepared following the procedure for PREP activity assay using 100 µM concentration of **HUP-55**, compound **2**, and vehicle. No substrates were used and the enzyme concentration was 3 times higher (6 nmol) than in PREP activity assay. After incubation 4 x volume methanol was added and samples were centrifuged for 10 min (4 °C, 14 000g). Supernatants were collected and analyzed with a Waters Synapt G2 HDMS mass spectrometer using ESI. The purity was determined by UPLC-MS with diode-array detector. Final compound concentrations were 20 µM in 0.2% DMSO 80% MeOH. Buffer and methanol with enzyme was used as a negative control and 50 µM **HUP-55** + compound **2** in methanol was used as a positive control. The results are shown in Figure S64.

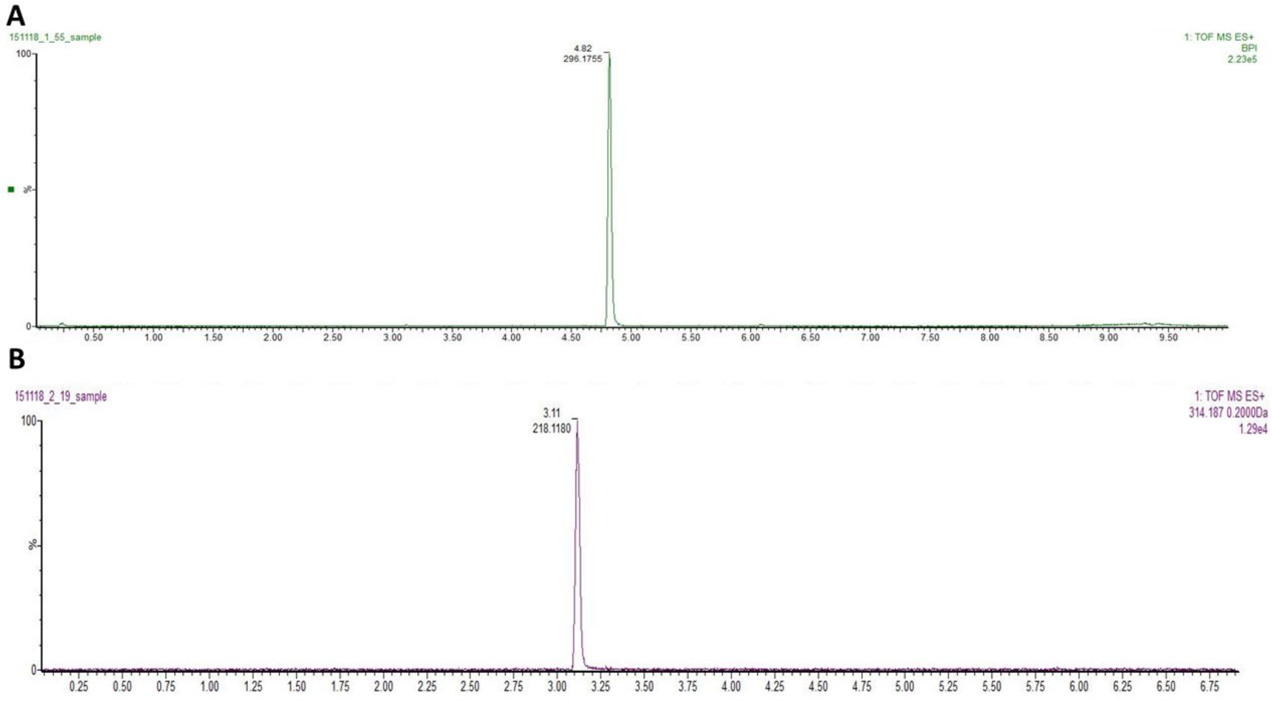

Figure S64. Total ion chromatograms (TIC) from samples of (A) **HUP-55** and (B) compound **2** after 90 min incubation in 0.1 M sodium–potassium phosphate buffer (pH 7.0) with porcine PREP enzyme. No hydrolysis of the oxazole ring was observed in the **HUP-55** sample.

HUP-55 configurational stability

The optical rotation for **HUP-55** was measured when it was first synthesized, and the measurement was repeated after ca 5 years of storage in a freezer at -18°C. No clear racemization of **HUP-55** was observed (Table S1). Optical activity was measured using a Modular Circular Polarimeter 200 (Anton Paar). MeOH was used as the solvent and the measurements were done at 20 °C, using a wavelength of 589 nm and a nominal cell length of 50.00 mm. The specific rotation was calculated from the measured optical rotation.

Table S1. Results of the polarimetry measurements.

Compound	Concentration (mg/ml)	Optical rotation	Specific rotation
HUP-55 (original)	9.8	-0.507°	-103.47
HUP-55 (after storage)	10.35	-0.520°	-100.48

Molecular modelling

Modelling studies were performed using Schrödinger Maestro.[2] The PREP crystal structure (PDB: 3DDU) was chosen since it is the only structure of human PREP, has a relatively good resolution (1.56 Å), and includes a co-crystalized ligand in the active site.[3] The structure was prepared using Protein Preparation Wizard with mostly default settings.[4] During pre-processing, missing side chains (Gln5, Gln56, Glu65, Glu69, Glu107, Glu163, Gln192, Asp256, Lys335, Gln388, Lys428, Asn483, Ile498, Lys546, Glu624 and Arg664) were filled with Prime[5] and heteroatom states were generated and selected at pH 7.4 with Epik.[6] All water, acetate, and glycerol molecules were removed. Hydrogen bond assignment was done using default settings at pH 7.4 using PROPKA.[7] Minimization was performed using default settings with force field OPLS3e. Ligands were prepared from their 2D structures with LigPrep using force field OPLS3e.[8] The grid for glide docking was generated,[9] the center of which was determined based on the co-crystalized ligand. The prepared ligands were docked at XP precision. The co-crystalized ligand was re-docked to ensure the viability of the model.

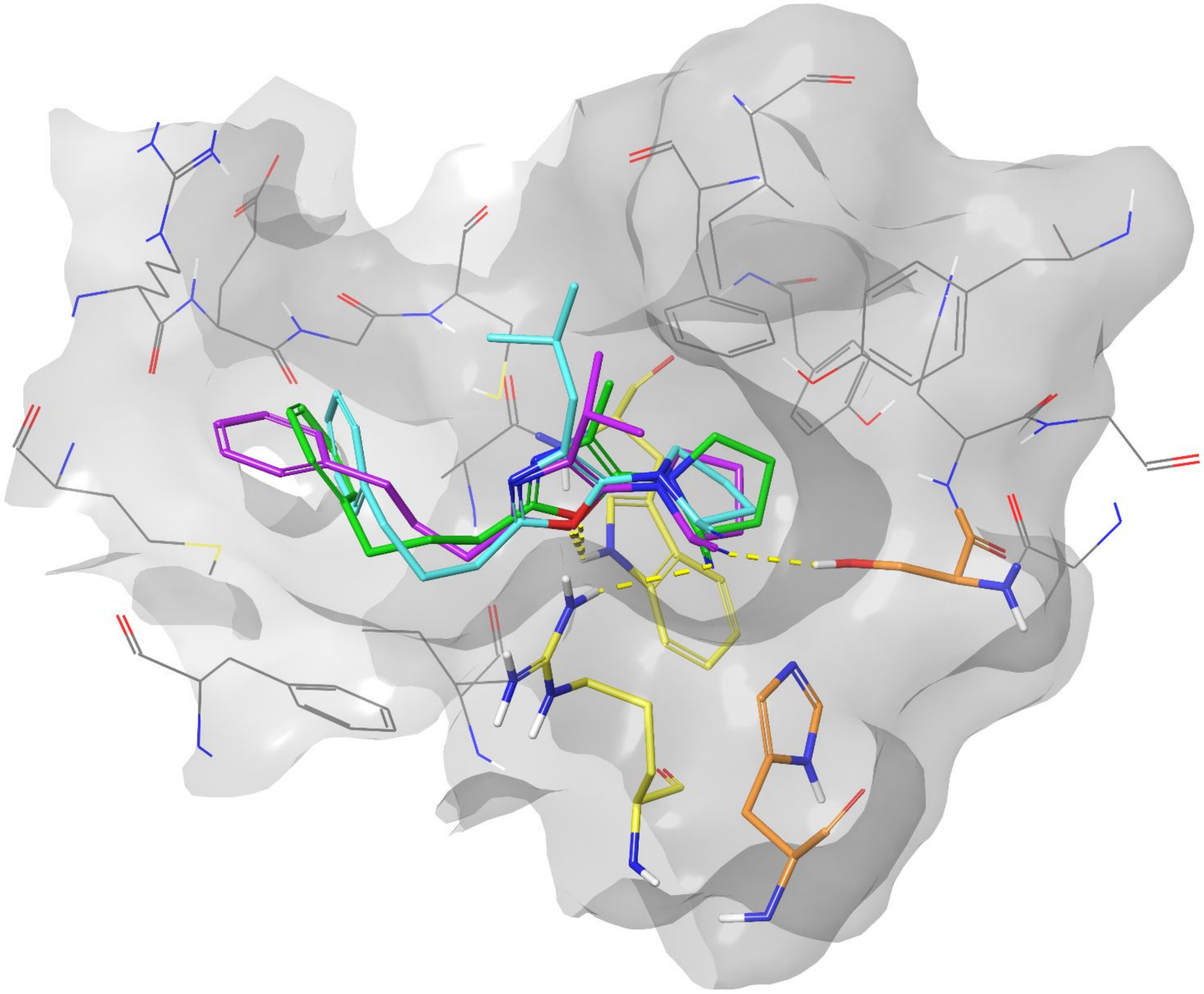

Figure S65. The active site of PREP (PDB ID: 3DDU) with Ser554 and His680 in orange and Trp595 and Arg643 in yellow. Other residues making up the active site are shown in grey. Hydrogen bonds are shown as yellow dashed lines. The docked compounds are **HUP-55** in green, compound **7** in purple, and compound **8** in cyan.

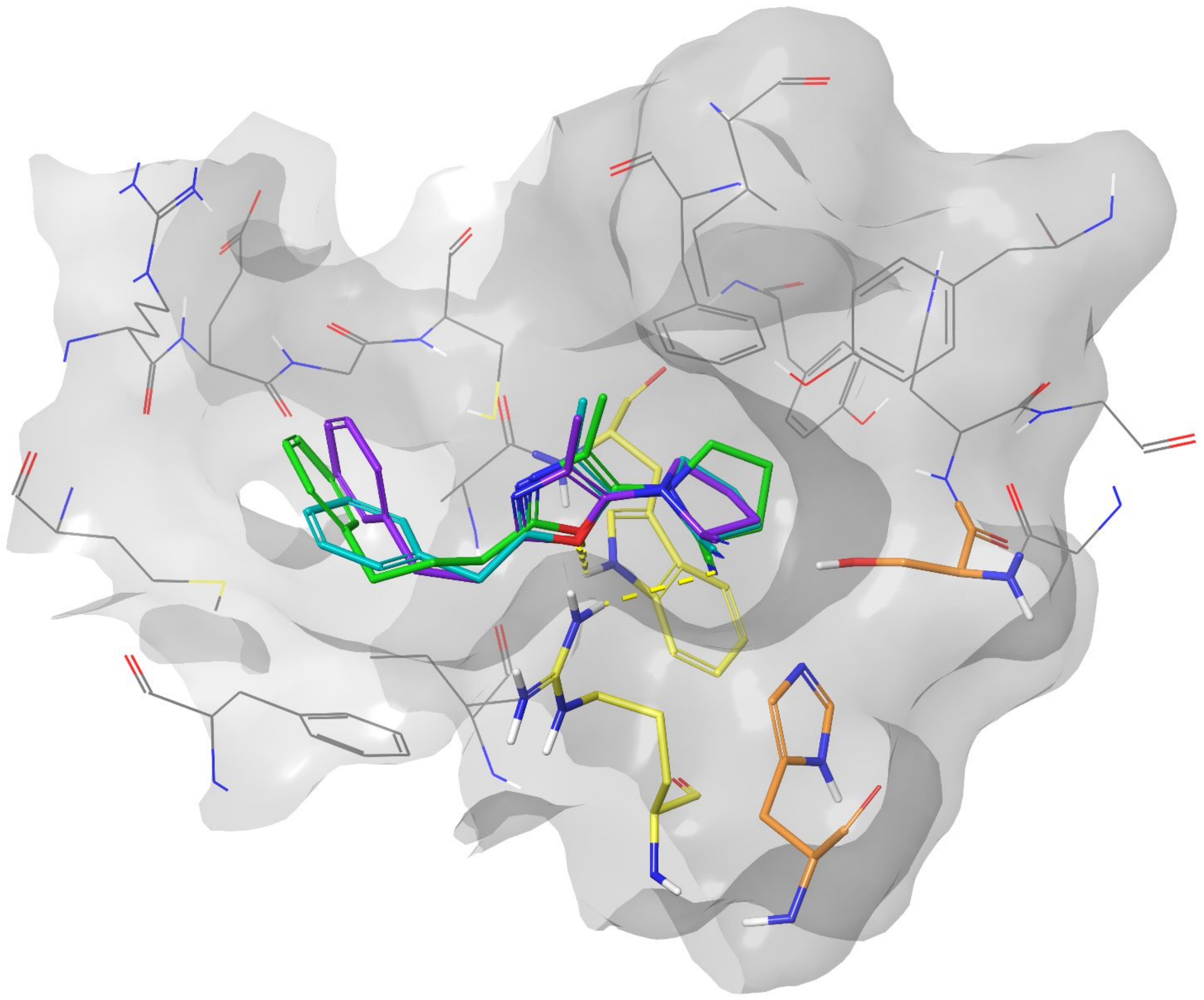

Figure S66. The active site of PREP (PDB ID: 3DDU) with Ser554 and His680 in orange and Trp595 and Arg643 in yellow. Other residues making up the active site are shown in grey. Hydrogen bonds are shown as yellow dashed lines. The docked compounds are **HUP-55** in green, compound **15** in purple, and compound **14** in cyan.

QikProp results for selected compounds

QikProp was run on Schrödinger Maestro for selected compounds using default settings. [2,10] The results are shown in Table S2.

Table S2. Relevant QikProp results for selected compounds.

Compound	QPlogPo/w[a]	QPlogS[b]	QPPCaco[c]	QPlogBB[d]
**HUP-55**	3.998	-6.074	1464.267	-0.612
**7**	4.91	-6.998	2733.632	-0.415
**8**	5.083	-7.112	1685.092	-0.697
**15**	3.52	-5.252	1760.997	-0.409

[a]Predicted octanol/water partition coefficient. [b]Predicted aqueous solubility (mol dm^{-3}). [c]Predicted apparent Caco-2 cell permeability (nm/sec). Less than 25 is considered poor and greater than 500 is considered great. [d]Predicted brain/blood partition coefficient (range from -3.0 to 1.2).

Biological data

Results of the screening assay

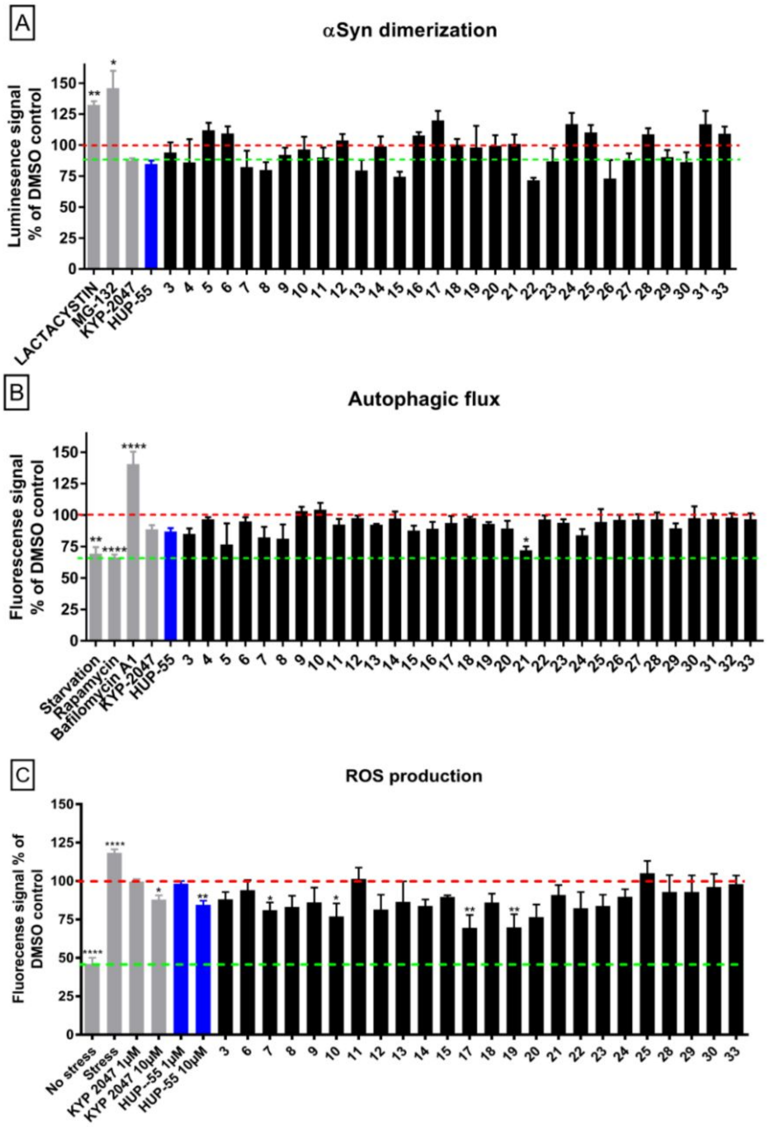

Figure S67. (A) Luminescence signal depicts αSyn-αSyn interaction and dimerization, and a lower signal compared to control indicates decreased dimerization. Test compounds were incubated in N2A cells for 24 h with 10 µM concentration, and 10 µM proteasome inhibitors lactacystin and MG-132 were used as positive controls. The red line indicates the level of DMSO control and the green line indicates the level of reference compound KYP-2047. (B) GFP-LC3-RFP expressing HEK-293 cells were used to measure autophagic flux. Reduced GFP signal indicates increased autophagic flux compared to control. 500 nM rapamycin and 24 h serum starvation were used as positive controls for increased autophagic flux and 20 nM bafilomycin 1A were used as autophagy inhibitor, and the concentration of study compounds was 10 µM for 24 h incubation. Red line indicates the level of DMSO control and green line indicates the level of rapamycin control. (C) Fluorescence signal shows formation of ROS after 3 h treatment in stressed SHSY-cells for selected oxazole compounds. A weaker signal indicates decreased ROS production compared to control. 10 µM concentrations for test compounds were used unless otherwise mentioned. Red line indicates the level of DMSO control and green line indicates the level of rapamycin control. 1-way ANOVA with Dunnet´s multiple comparison, ****<0.0001, ***<0.0005, **<0.005, *<0.05. All results are presented as mean+SEM.

Toxicity testing of selected compounds

The impact of compounds on cell viability was assayed at the end of incubation period using a standard MTT assays as described earlier in Svarcbahs et al.[11] and Myöhänen et al.[12] HEK-293 and SH-SY5Y cells and mouse primary neurons were seeded onto 96-well plates (10,000 cells/well) and incubated overnight. Thereafter, the cells were incubated with 1, 10, and 100 μM of study compounds for 24 h (HEK-293 and SH-SY5Y cells) or 48 h (mouse primary neurons), followed by the measurement of mitochondrial dehydrogenase activity.

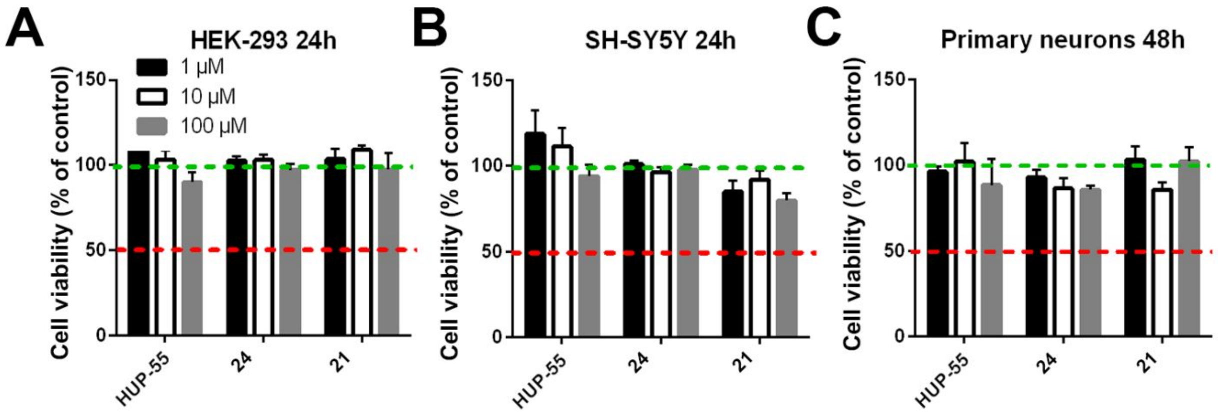

Figure S68. Selected oxazoles were tested for cell viability in an MTT assay after 24 h treatment in (A) HEK-293 cells and (B) SH-SY5Y cells and 48 h treatment in (C) mouse primary neurons. Even with the high concentration (100 uM), mouse primary neurons did not show any toxicity. Data are presented as mean + SEM. **, p<0.01; student's t-test.

LC-MS detection of HUP-55 metabolites in the mouse brain

The same column and gradient was used with UPLC-HDMS analysis of the metabolites of **HUP-55**, as for the analysis of **HUP-55** detailed in the experimental section of the article. The analyses were performed with ESI (+) resolution mode and the exact masses were analysed with UPLC-Synapt G2 QTOF/MS (Waters). The elemental composition for the metabolites of **HUP-55** was calculated with MassLynx v4.2. The results are detailed in table S2.

Table S3. LC-MS results of **HUP-55** and its metabolites in the mouse brain.

Compound	Retention time (min)	m/z $(M+H)^+$	Empirical formula
**HUP-55**	4.83	296.1753	$C_{18}H_{22}N_3O$
Metabolite 1	3.19	301.1552	$C_{17}H_{21}N_2O_3$
Metabolite 2	3.67	312.1712	$C_{18}H_{22}N_3O_2$
Metabolite 3	3.83	285.1603	$C_{17}H_{21}N_2O_2$
Metabolite 4	4.20	326.1505	$C_{18}H_{20}N_3O_3$

Results of the locomotor activity recordings

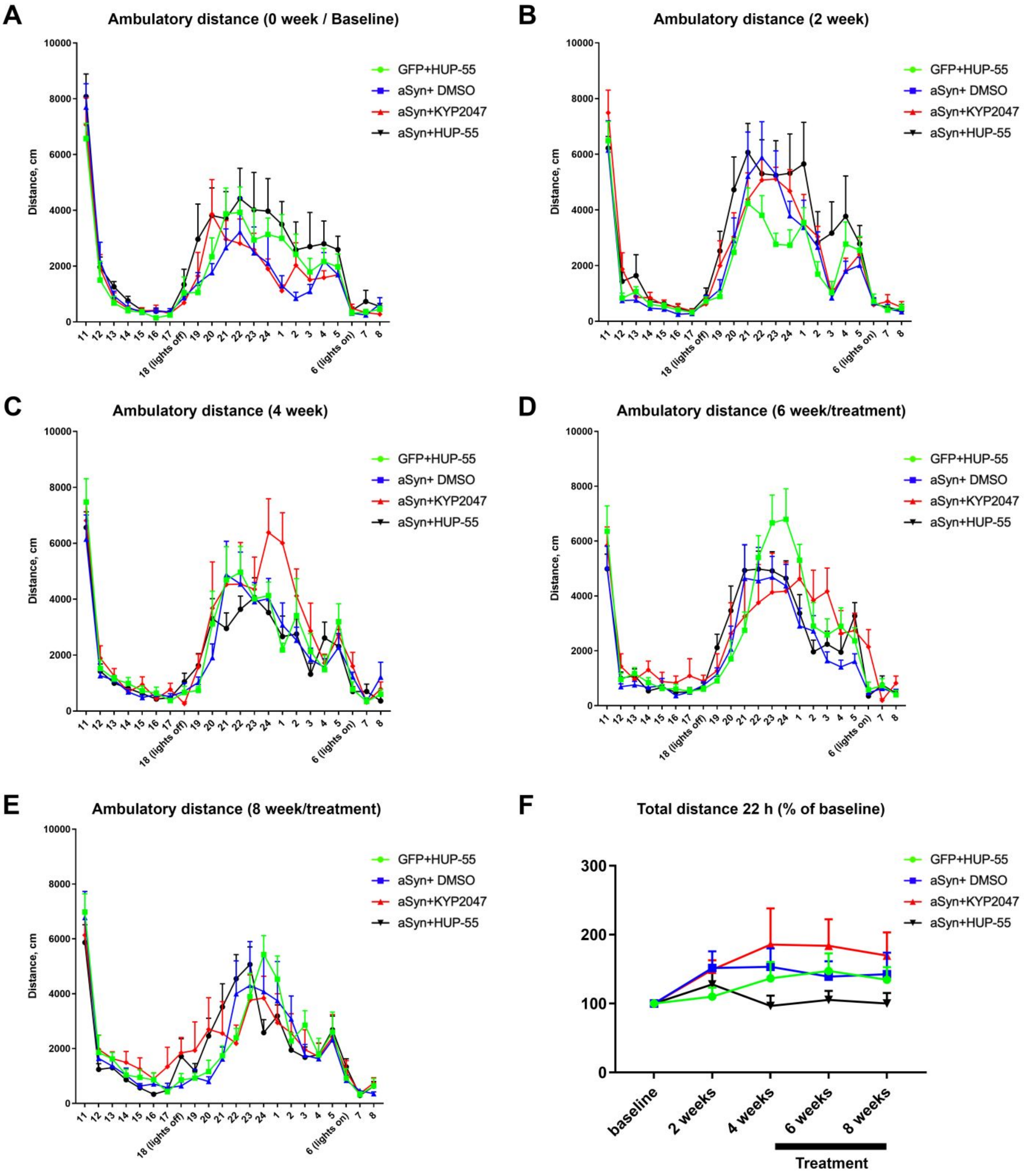

Figure S69. Results of the 22 h locomotor activity recordings from baseline (A) and 2 (B), 4 (C), 6 (D), and 8 (E) weeks post AAV-injection. The total distance change from the baseline is shown in panel F. No clear effects between AAV-injections or the treatments were seen throughout the recording period. Data is expressed as mean ± SEM; n = 9-10/group.

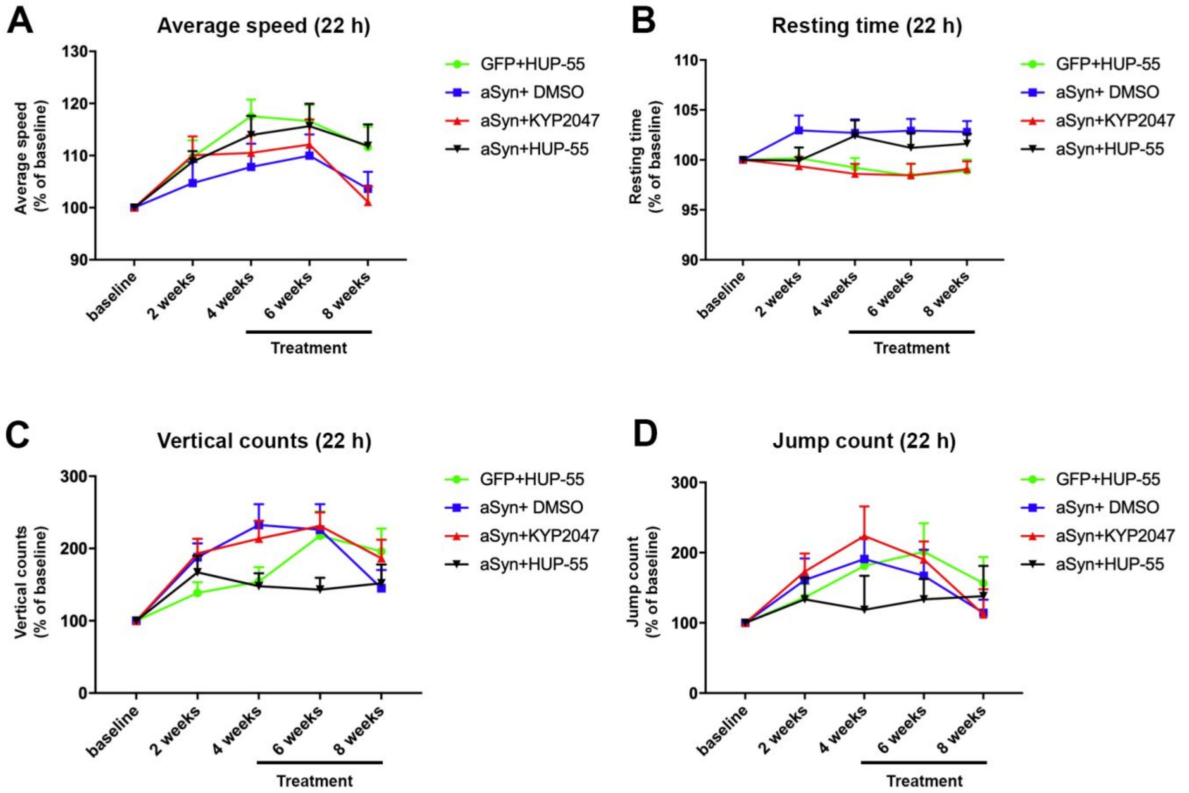

Figure S70. Parameters from 22 h locomotor measurements. Average speed (A), resting time (B), vertical counts (C) and jump count (D) were measured. No clear effects between AAV-injections or the treatments were seen throughout the recording period. Data is expressed as % of the baseline and mean ± SEM; n = 9-10/group.

Results of total aSyn immunoreactivity in striatum

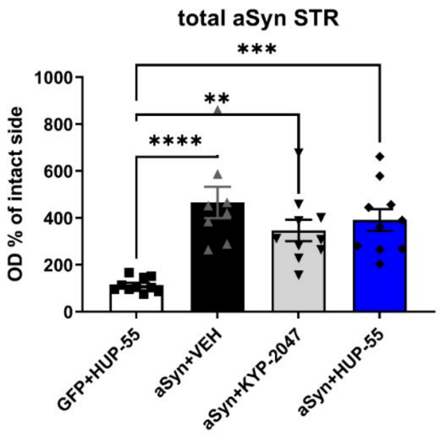

Figure S71. Total aSyn levels from the striatum. AAV-aSyn injected mice had significantly higher aSyn levels in the striatum compared to GFP-injected mice. No statistical difference by the treatment was found. 1-way ANOVA compared was used with Dunnett´s multiple comparison test, **** p<0.0001, *** p=0.0003. Data is presented as means+SEM.

References

1. Mestrelab Research S.L., MestReNova version 12.0.1
2. Schrödinger Release 2020-3: Maestro; Schrödinger, LLC, New York, NY, 2020.
3. Haffner, C. D., Diaz, C. J., Miller, A. B., Reid, R. A., Madauss, K. P., Hassell, A., Hanlon, M. H., Porter, D. J., Becherer, J. D., Carter, L. H. (2008). Pyrrolidinyl pyridone and pyrazinone analogues as potent inhibitors of prolyl oligopeptidase (POP). *Bioorganic & Medicinal Chemistry Letters*, 18, 4360- 4363.
4. Schrödinger Release 2020-3: Protein Preparation Wizard; Schrödinger, LLC, New York, NY, 2020.
5. Schrödinger Release 2020-3: Prime; Schrödinger, LLC, New York, NY, 2020.
6. Schrödinger Release 2020-3: Epik; Schrödinger, LLC, New York, NY, 2020.
7. Olsson, M. H. M., Søndergaard, C. R., Rostkowski, M., Jensen, J. H. (2011). PROPKA3: Consistent Treatment of Internal and Surface Residues in Empirical pKa Predictions. *Journal of Chemical Theory and Computation*, 7, 525-537.
8. Schrödinger Release 2020-3: LigPrep; Schrödinger, LLC, New York, 2020.
9. Schrödinger Release 2020-3: Glide; Schrödinger, LLC, New York, 2020.
10. Schrödinger Release 2022-3: QikProp; Schrödinger, LLC, New York, NY, 2020.
11. Svarcbahs, R., Julku, U. H., Norrbacka, S., & Myöhänen, T. T. (2018). Removal of prolyl oligopeptidase reduces alpha-synuclein toxicity in cells and in vivo . Scientific Reports, 8, Article 1552.
12. Myöhänen, T. T., Hannula, M. J., van Elzen, R., Gerard, M., van der Veken, P., García-Horsman, J. A., Baekelandt, V., Männistö, P. T., & Lambeir, A. M. (2012). A prolyl oligopeptidase inhibitor, KYP-2047, reduces α-synuclein protein levels and aggregates in cellular and animal models of Parkinson's disease. *British Journal of Pharmacology*, *166*, 1097-1113.
